# Economy of Catalyst Synthesis—Convenient Access to Libraries of Di- and Tetranaphtho Azepinium Compounds

**DOI:** 10.3390/molecules23040750

**Published:** 2018-03-24

**Authors:** Sorachat Tharamak, Christian Knittl-Frank, Auraya Manaprasertsak, Anchulee Pengsook, Lydia Suchy, Philipp Schuller, Barbara Happl, Alexander Roller, Michael Widhalm

**Affiliations:** 1 Department of Chemistry, Faculty of Science, Kasetsart University, Bangkok 10900, Thailand; sorachat.th@ku.th (S.T.); auraya.ma@ku.th (A.M.); anchulee.peng@ku.th (A.P.); 2 Institute of Organic Chemistry, University of Vienna, Währinger Straße 38, Wien 1090, Austria; christian.knittl-frank@univie.ac.at (C.K.-F.); a01205005@unet.univie.ac.at (L.S.); philipp.daniel.schuller@gmail.com (P.S.); 3 Institute of Inorganic Chemistry, University of Vienna, Währinger Straße 42, Wien 1090, Austria; barbara.happl@univie.ac.at (B.H.); alexander.roller@univie.ac.at (A.R.); 4 Institute of Chemical Catalysis, University of Vienna, Währinger Straße 38, Wien 1090, Austria

**Keywords:** 1,1′-binaphthyl, biphenyl, Suzuki-Miyaura coupling, phase transfer catalyst

## Abstract

Efficient optimization procedures in chiral catalysis are usually linked to a straightforward strategy to access groups of structurally similar catalysts required for fine-tuning. The ease of building up such ligand libraries can be increased when the structure-modifying step (introduction of a substituent) is done at a later stage of the synthesis. This is demonstrated for the extended family of di- and tetranaphtho azepinium compounds, widely used as chiral phase transfer catalysts (PTC). Using 2,6-diiodo-4,5-dihydro-3*H*-dinaphtho[2,1-c:1′,2′-e]azepine and 4,8-diiodo-6,7-dihydro-5*H*-dibenzo[c,e]azepine, respectively, as key intermediates, 18 *spiro*-azepinium compounds were synthesized in a total yield of 25–42% over 6–7 steps from 1,1′-binaphthyl-2,2′-dicarboxylic acid or diphenic acid, respectively. The replacement of iodo groups with aryl substituents was performed as the last or the penultimate step of the synthesis.

## 1. Introduction

For the practical application of organocatalytic transformations, an economic access to libraries of potentially useful catalysts is essential. As organocatalytic reactions are still optimized empirically, easy access to stepwise structural modification of catalysts is essential for thorough and rapid screening. Synthetic work will be minimized if the modifiers are introduced at the latest stage possible of a synthetic sequence. This necessitates the proper choice of a reactive precursor which can be readily transformed to the chiral catalysts under mild reaction conditions compatible with other functional groups already present.

**Figure 1:**
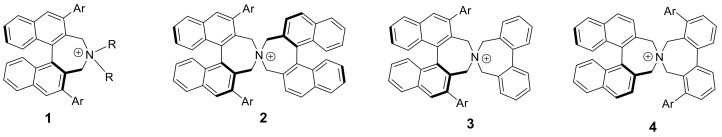
Phase transfer catalysts with dinaphthoazepinium backbone.

A class of versatile organocatalysts like **1**–**4** (Figure 1) is based on the *C*_2_-symmetric dinaphthoazepinium backbone [1]. Such catalysts have been applied as highly efficient PTCs, references [2,3,4,5] most prominently for the allylation and benzylation of *N*-protected glycine esters [6,7,8,9,10]. Variations were particularly made by changing *N*-substituents, as well as 3,3′-aryl groups, with different degrees of bulkiness and additional electron donating or withdrawing substituents [11,12,13,14].

### Comparison of Synthetic Concepts

**Scheme 1:**
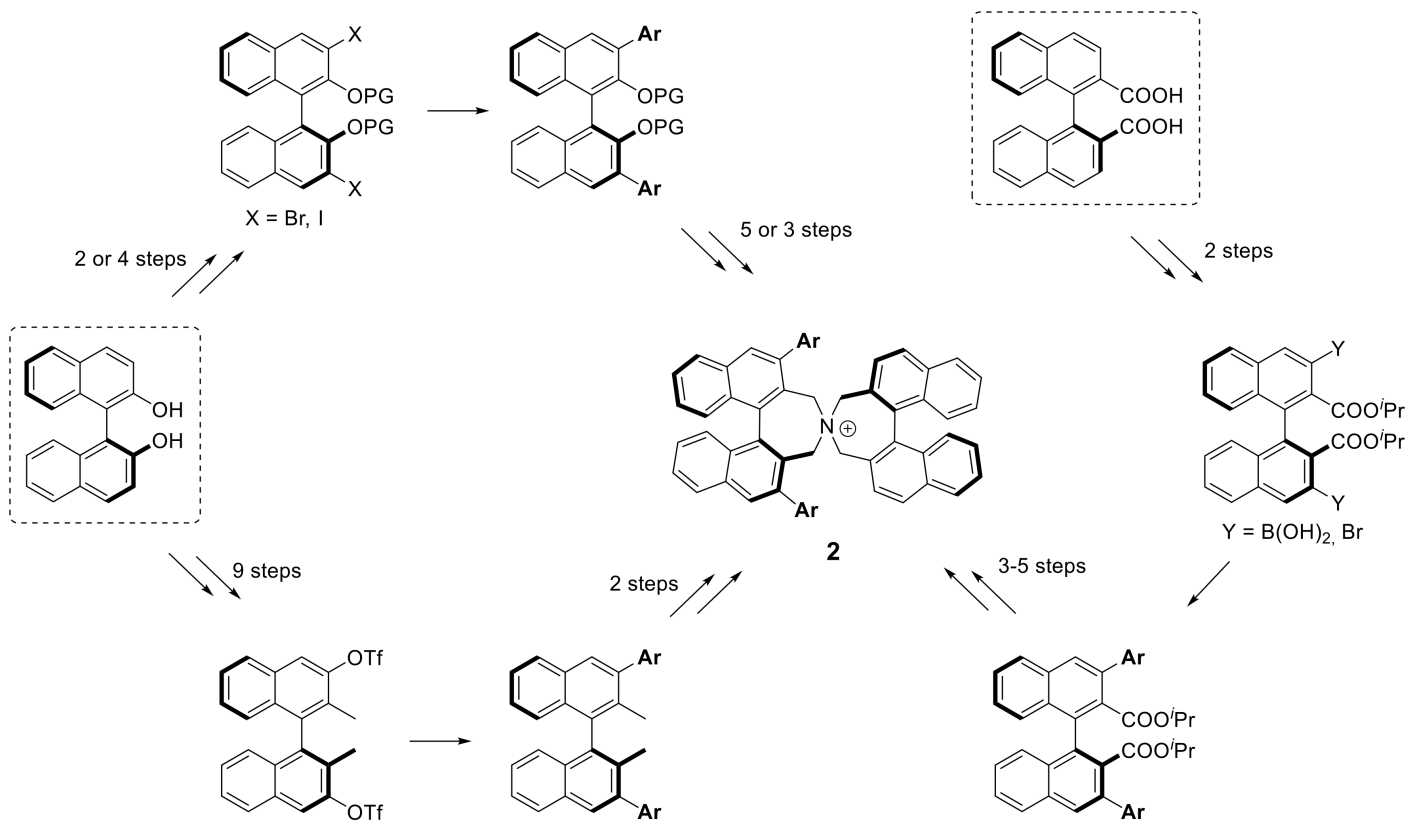
Published syntheses of *N*-*spiro*-azepinium bromides **2**.

Several routes to azepinium-type PTCs have been developed, starting either from commercially available enantiopure 2,2′-dihydroxy-1,1′-binaphthyl or from 1,1′-binaphthalene-2,2′-dicarboxylic acid, to yield a broad range of 3,3′-diaryl-substituted bisazepinium compounds (see Scheme 1 for a summary). The published syntheses from 2,2′-dihydroxy-1,1′-binaphthalene required in total 13 to 17 steps [15] with the introduction of 3,3′-aryl substituents in the third, fifth or tenth step of the linear sequence (8 or 12 steps). This in turn means that for each individual catalyst 2–5 steps had to be performed afterwards to complete the synthesis. An alternative approach used 1,1′-binapthalene-2,2′-dicarboxylic acid as the key intermediate, [16] which was typically prepared in 4 to 6 steps from binaphthol or 2-methylnaphthalene (for details see Supplementary Materials) [17,18]. In the latter case, an optical resolution procedure had to be performed. The corresponding 2,2′-diisopropylester was *ortho*-metallated and converted into 3,3′-diaryl-2,2′-carboxylates (via the diboronic acid). Three more steps led to the desired *spiro*-ammonium catalysts. Some improvement was made by using other *ortho*-directing ester groups [19] and *ortho*-metallation/arylation of free carboxylic acid, [20] which shortened the synthesis in several cases.

**Scheme 2:**
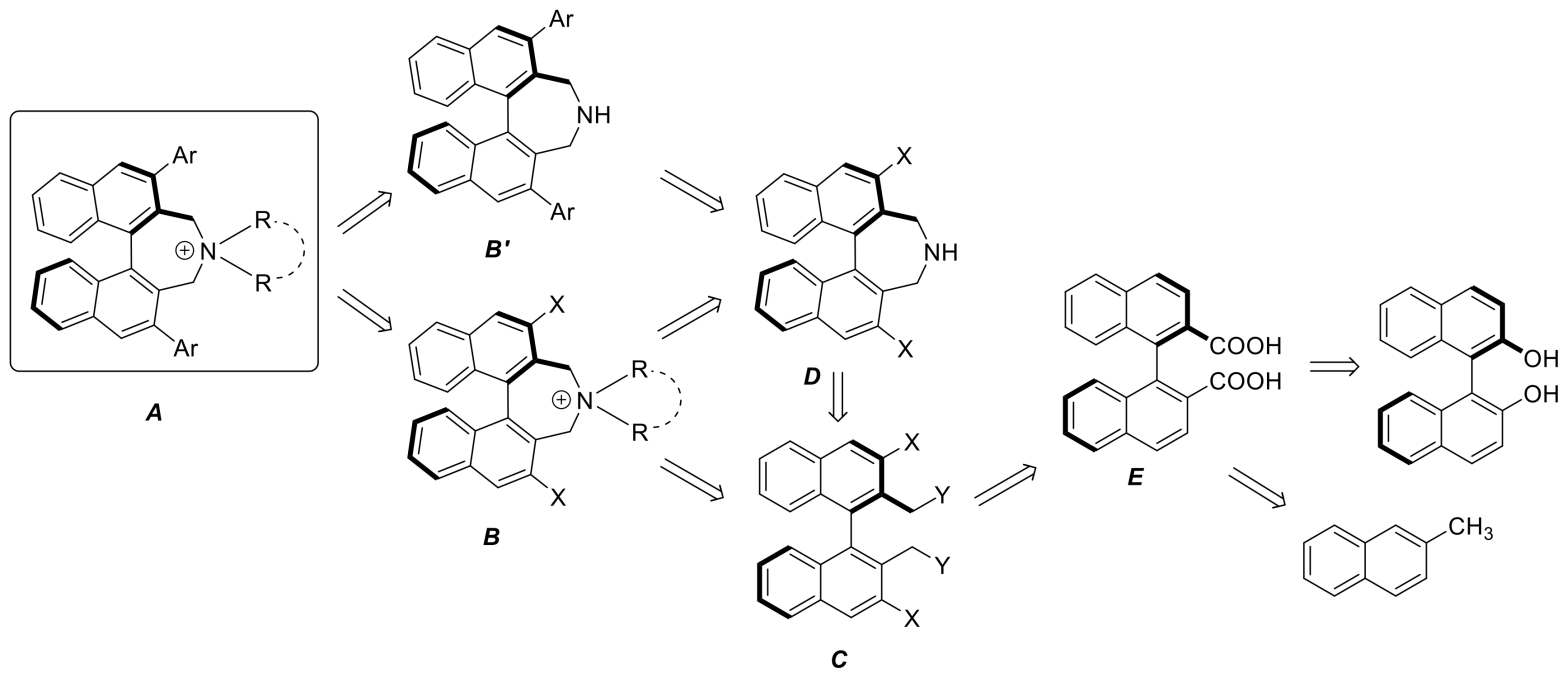
Retrosynthetic approach to biarylazepinium type libraries (X, Y = Br, I).

Retrosynthetic analysis revealed that the economy of accessing azepinium compounds could be improved when structures *C* and *D* with X = I, Br were chosen as key intermediates (Scheme 2). This would allow the introduction of variable *N*-substituents at a late stage, just before attaching aromatic groups at C-3 and C-3′. (path *C-B-A*). Alternatively, azepine *D* can be prepared from *C*, followed by 3,3′-arylation or quaternation of the nitrogen atom (path *D-B-A* vs. *D-B′-A*). Since halide substituents are present in *C* and *D*, this is compatible with carboxylate as an *ortho*-directing group (*E*).

## 2. Results and Discussion

**Scheme 3:**
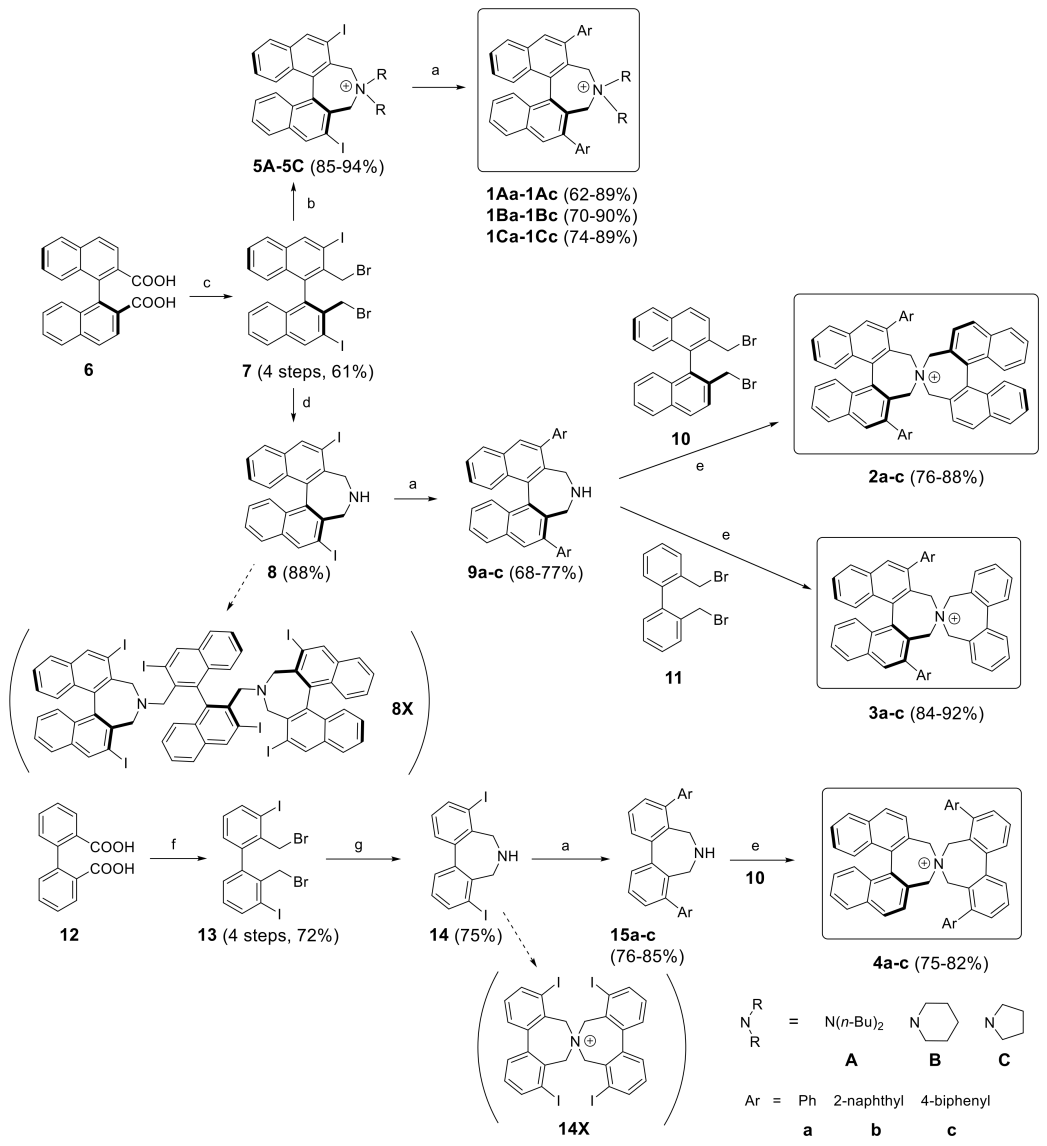
Access to PTC **1**–**4** (counter anions omitted). *Reagents and Conditions*: a: ArB(OH)_2_, Na_2_CO_3_, Pd(Ph_3_P)_4_, toluene, 80 °C, 4–20 h. b: R_2_NH, K_2_CO_3_, CH_3_CN, 80 °C, 22–24 h. c: ref. [21]. d: NH_3_ (25%), CH_3_CN, 60 °C, 24 h. e: K_2_CO_3_, CH_3_CN, 90 °C, 24 h. f: (1) Li-TMP, Me_3_SiCl, THF, −78 °C to r.t. (2) BH_3_·THF, THF, reflux, 24 h. (3) ICl, DCM, −40 °C, 2 h. (4) HBr, HOAc, reflux, 2 h. g: NH_3_ (25%), CH_3_CN, 50 °C, 48 h.

**Scheme 4:**
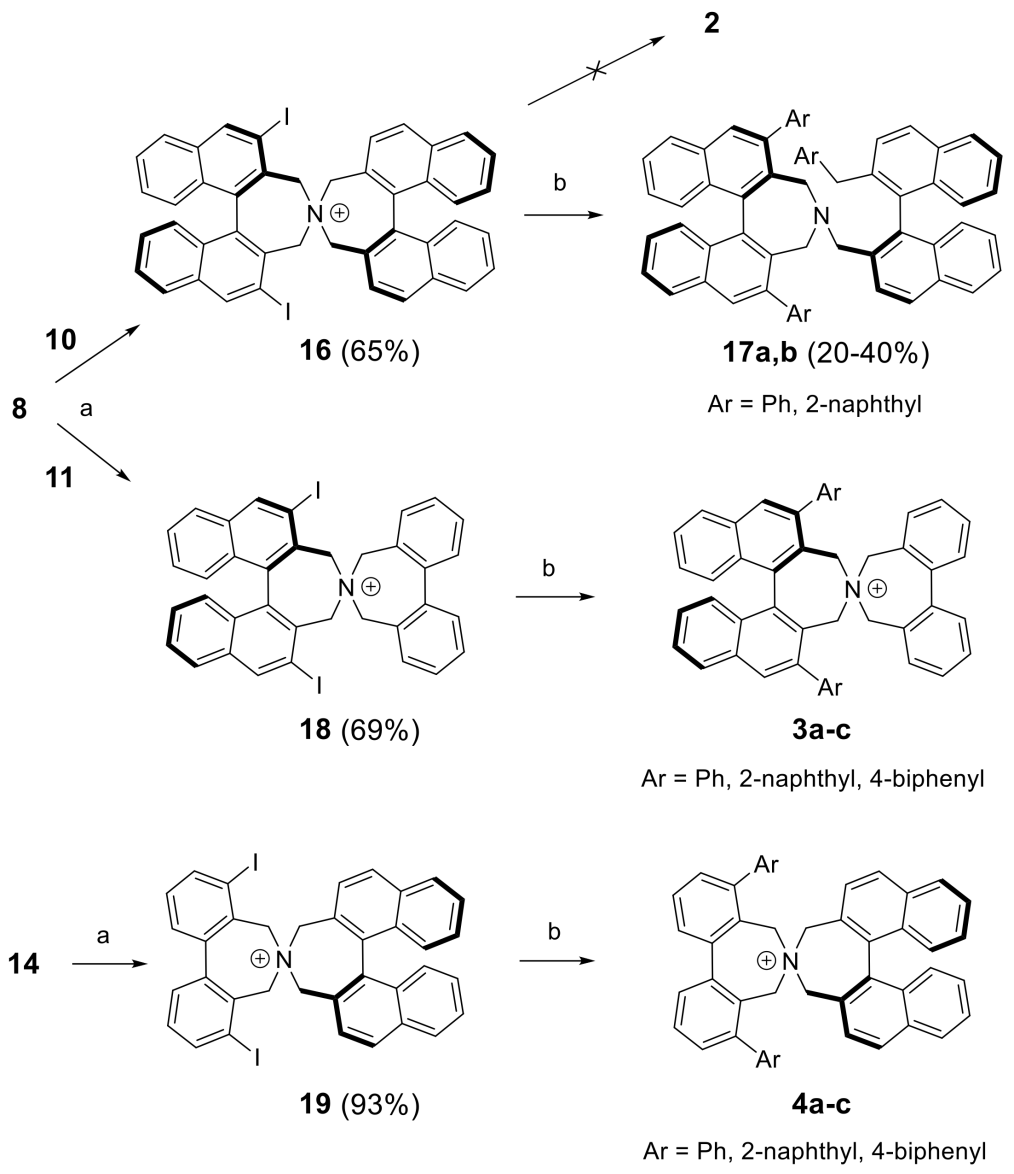
Arylation of diiodo-*N-spiro*-bisazepinium bromides **16**, **18** and **19** under Suzuki conditions (counter anions omitted). *Reagents and Conditions*: a: K_2_CO_3_, CH_3_CN, 80 °C, 12 h. b: ArB(OH)_2_, Na_2_CO_3_, Pd(Ph_3_P)_4_, toluene, 80 °C, 4–24 h.

**Figure 2:**
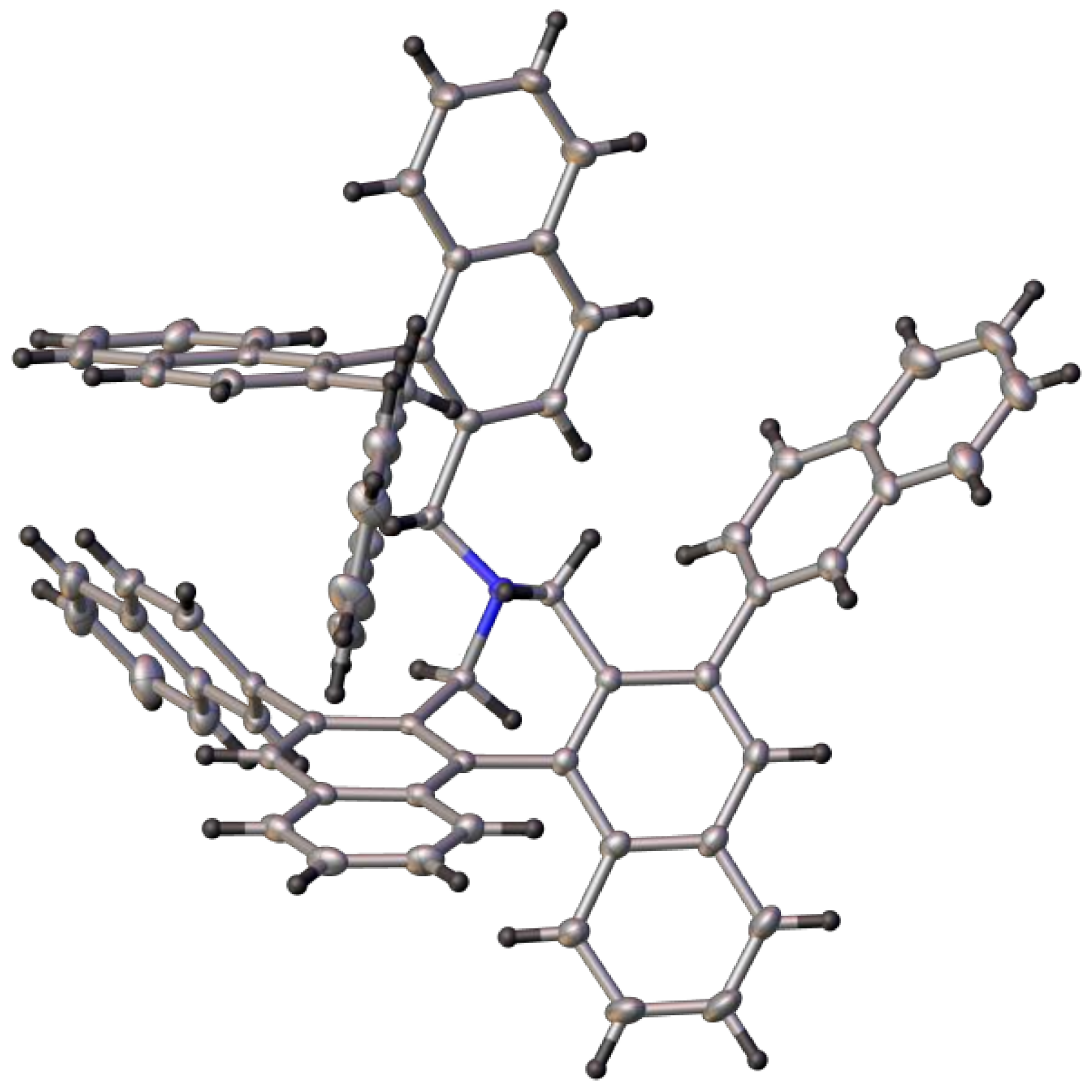
X-ray structure of **17b**.

**Figure 3:**
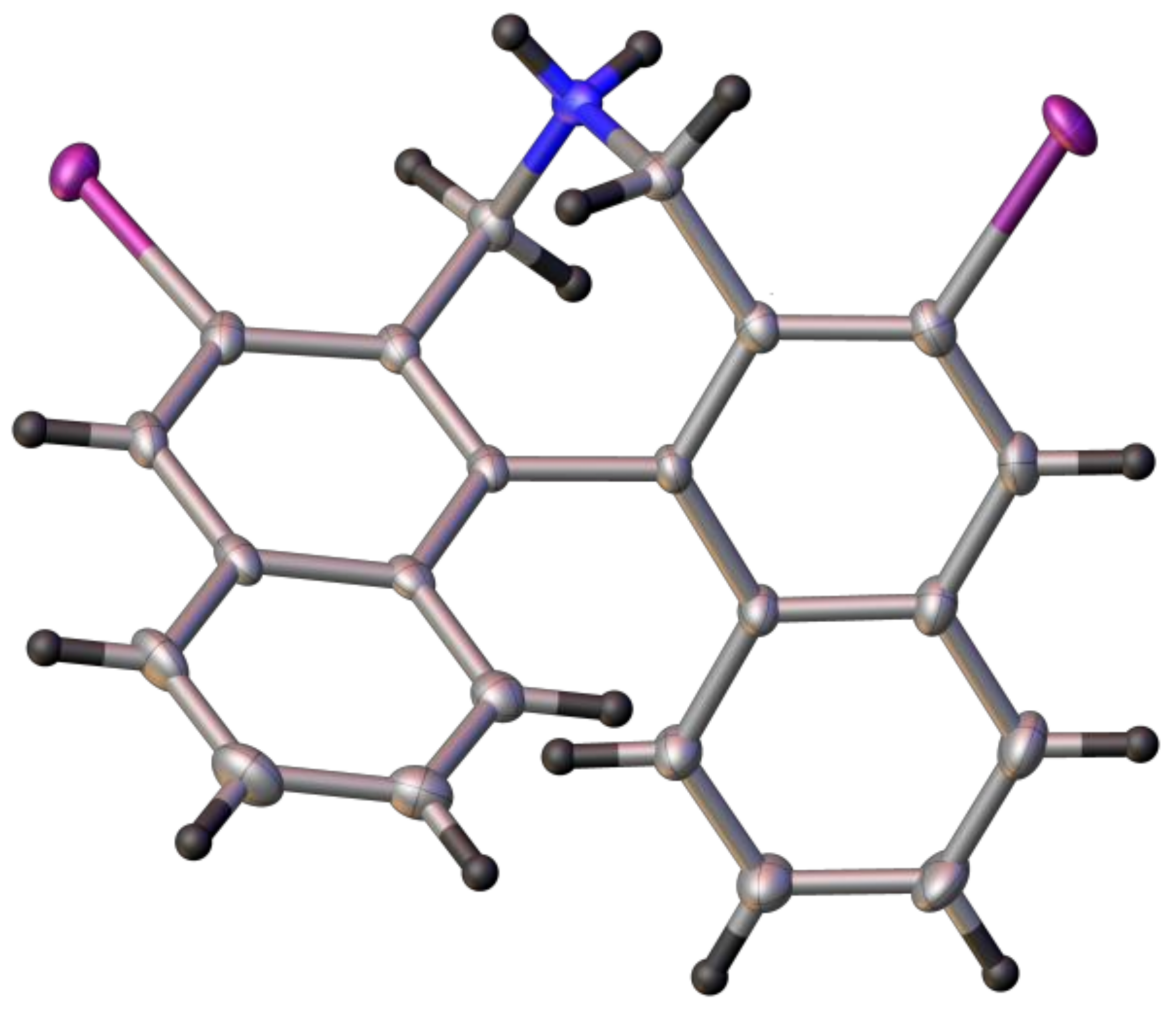
X-ray structure of **8**·HBr (counterion and solvent molecules ommitted).

**Figure 4:**
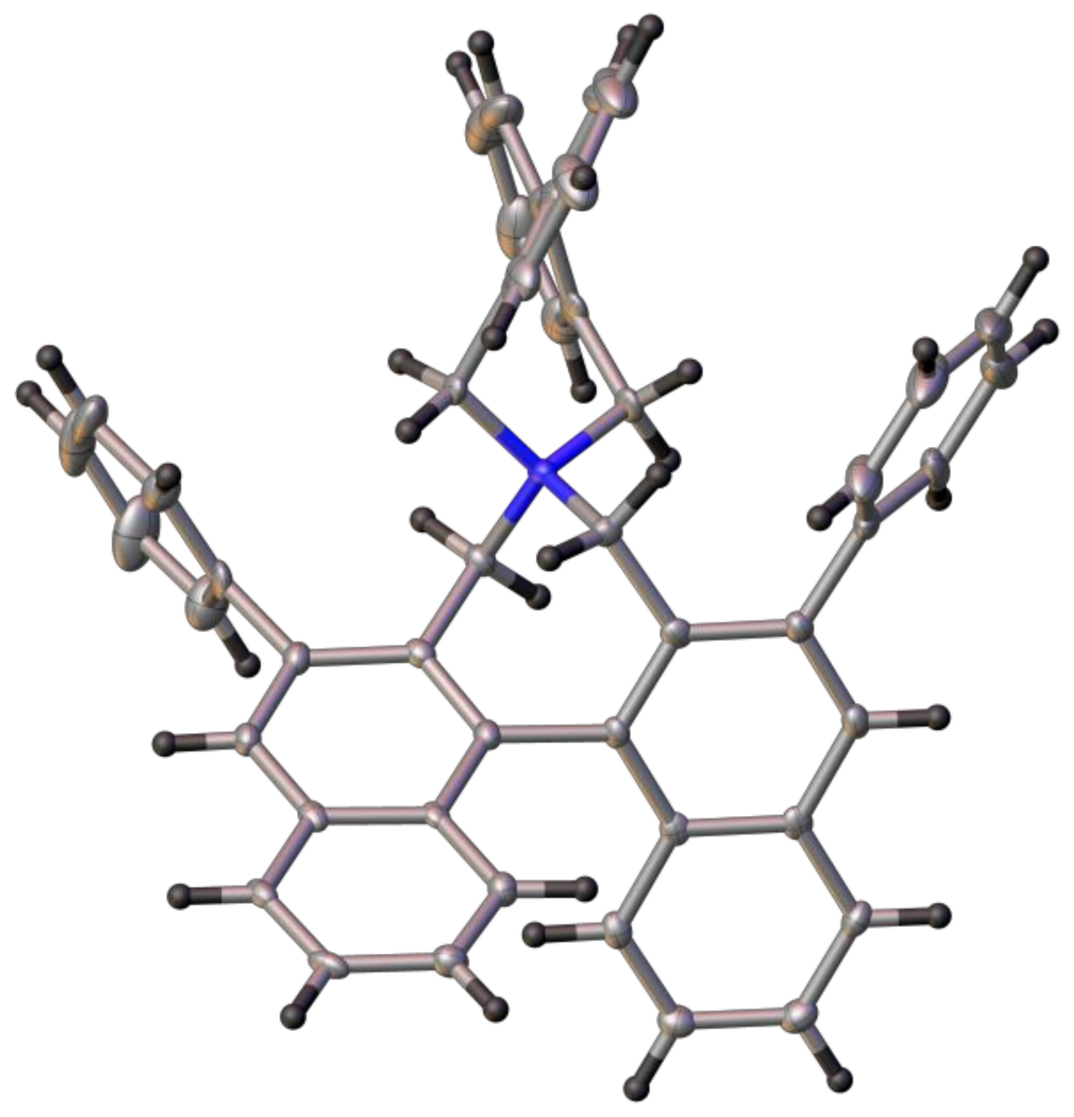
X-ray structure of **3a** (counterion and solvent molecules ommitted).

**Figure 5:**
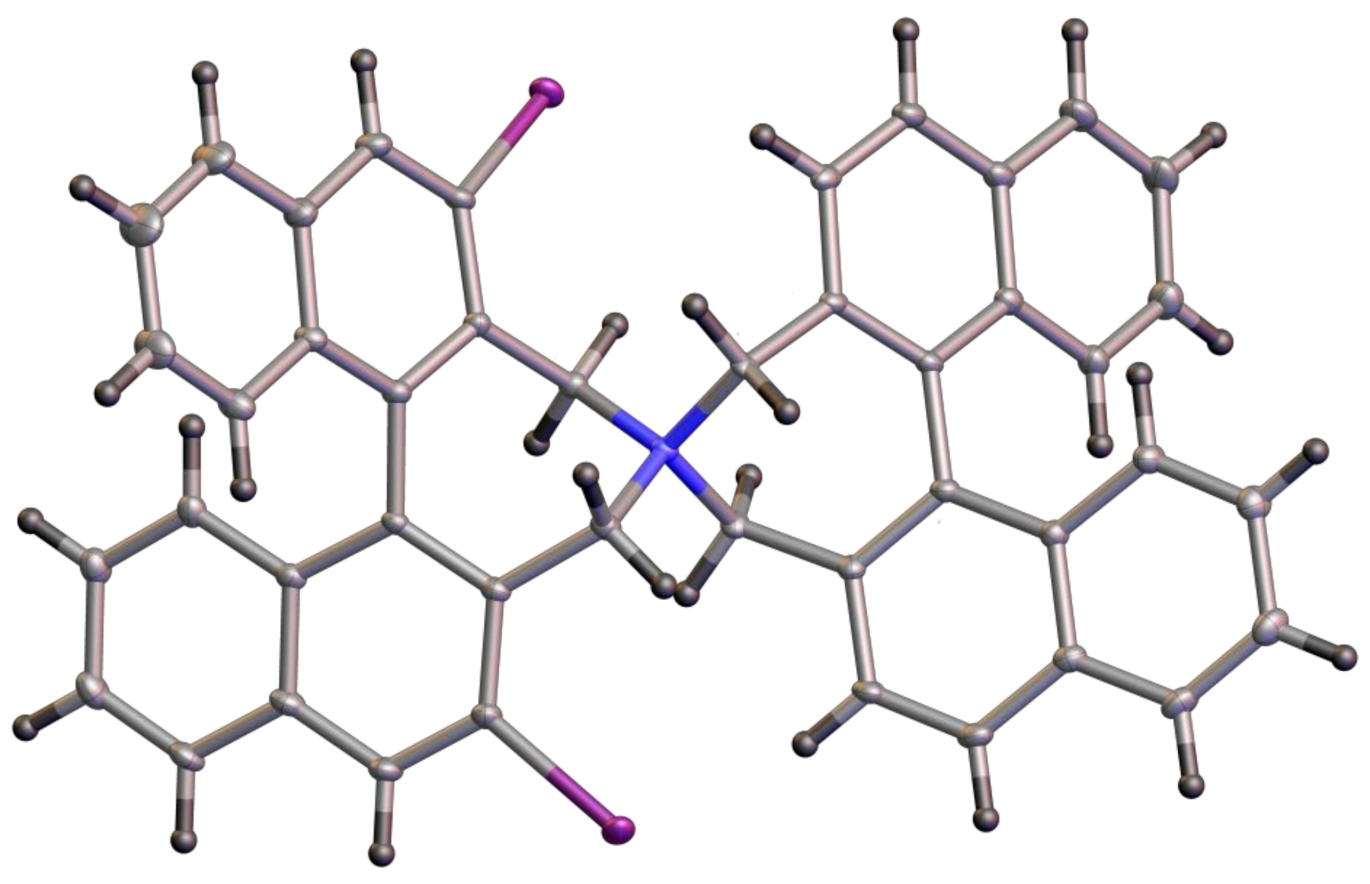
X-ray structure of **16** (counterion and solvent molecules ommitted).

Based on these considerations, an alternative synthetic protocol for **1**–**4** is presented in Scheme 3. Intermediate **7**, which is available in four steps from **6** [21] using an improved procedure for the last step (see the Materials and Methods section), was cyclized to azepinium salts **5A**–**C** or diiodoazepine **8**. In a preliminary feasibility study, it turned out that **7** was a versatile precursor, forming **5** smoothly with various secondary amines (dibutylamine, dimethylamine, pyrrolidine, piperidine, etc.). The subsequent twofold Suzuki-Miyaura coupling of ammonium salts yielded **1Aa**–**1Cc** in good yield (62–90%, not optimized). In contrast, applying the same conditions to **16** (Scheme 3) resulted in a complex mixture from which triarylated ring-opened products **17a** and **17b** could be isolated. The solid state structure of **17b** was determined (Figure 2). Instead, ligands of type **2** and **3** were accessible through dinaphthoazepine **8** (intermediate *D*, X = I in Figure 1, crystal structure of **8**·HBr in Figure 3). High yields were obtained when the temperature was kept at 60 °C, however, if the temperature was raised to 80 °C, considerable amounts of a sparingly soluble “diamino trimer” **8X** were isolated. Suzuki coupling afforded **9a**–**c** in good yield and cyclization with non-racemic **10** or **11** gave **2a**–**c** and **3a**–**c** in 76–92% yield. In the latter case, characterisation of products was hampered due to extreme broadening of NMR signals. In several cases, high temperature NMR (378 K in DMSO-*d_6_*) gave more informative spectra. Proposed structures have been confirmed by HRMS and by X-ray structure analysis in one case (**3a**, Figure 4). Similarly, dinaphthodibenzoazepinium compounds **4a**–**c** were obtained from diphenic acid **12**. During synthesis of **14**, the formation of small amounts (8–13%) of *N-spiro*-bisazepinium bromide **14X** could not be completely suppressed, however, it was easily removed by chromatography. It is worth noting that **3a**–**c** and **4a**–**c** have been alternatively synthesized from diiodo precursors **18** and **19**, respectively in fair to good yields. However, chromatographic purification of the azepinium salts proved difficult and in several cases much product was nonisolable (Scheme 4). The contrasting reactivity of **16** and its failure as a precursor for **2a–c** remains unclear. The crystal structure did not show significant distortions pointing to steric strain in the azepine ring (Figure 5).

The economic benefits of the synthetic concept towards chiral ammonium salts are various. Synthesis of key intermediates **7** and **8** from diacid **6**, and **14** from **12** can be done on a multi-gram scale in 4 or 5 steps without requirement for column chromatography. The cyclization of **7** with aqueous ammonia obviates the previously applied two-step methodology where 3,3′-diaryl substituted dibromides were cyclized with allylamine, benzylamine or hydroxylamine followed by removal of the allyl, benzyl or hydroxy substituent [15,22,23,24,25,26]. Side reactions can be largely suppressed if the temperature is kept low during the cyclization step. The introduction of aryl substituents is now shifted to the last step (**1Aa**–**1Cc**) or the penultimate step (**2a**–**c**, **3a**–**c**, **4a**–**c**).

While **12** is commercially available at a reasonable price, the synthesis of racemic or non-racemic **6** will be often performed by researchers. The costs and ease of accessing this important key intermediate will also influence the decision on the practical application of a class of catalysts. Several routes to **6** have been published through the last decades. Particularly, practical considerations comprising complexity of optical resolution, purification of intermediates, price and availability of reagents, the time needed for preparation, ease of purification and overall yield might help to choose a synthetic path. An overview and evaluation of methods can be found in the Supplementary Material.

Summarizing, we presented a highly flexible route to binaphthyl based azepinium compounds with the aim to facilitate access to libraries of organocatalysts. Reference [27] As key intermediates, 4,8-diiodo-6,7-dihydro-5*H*-dibenzo[c,e]azepine **14** was prepared from diphenic acid **12** (five steps, 59% overall yield) and 2,6-diiodo-4,5-dihydro-3*H*-dinaphtho[2,1-c:1′,2′-e]azepine **8** from 1,1′-binaphthyl-2,2′-dicarboxylic acid **6** (five steps, 42% overall yield). Two more steps (Suzuki-Miyaura coupling with various arylboronic acids followed by *spiro*-cyclization) afforded *C_2_*-symmetrical ammonium salts **2**–**4** in good yield. A similar protocol led to various dinaphthoazepinium bromides **1Aa**–**1Cc** accessible from 3,3′-diiodo-2,2′-bis(bromomethyl)-1,1′-binaphthyl **7** in two steps.

## 3. Materials and Methods

### 3.1. General Considerations

Melting points: Kofler melting point apparatus (Reichert Thermovar, Reichert Technologies, Depew, NY, USA), uncorrected. NMR: recorded at 400.27 MHz, 500.32 MHz, or 600.25 MHz (^1^H) and 100.66 MHz, 125.81 MHz, or 150.95 MHz (^13^C), on AV 400, AV 500, AV 600 (Bruker Biospin, Billerica, MA, USA) respectively. Chemical shifts δ are reported in ppm; for ^1^H rel. to (residuals non-deuterated) solvent signals (chloroform-*d*: 7.26, DMSO-*d*_6_: 2.50 ppm), for ^13^C to CDCl_3_ at 77.00 ppm, or DMSO-*d*_6_ at 39.52 ppm, respectively. Coupling patterns are designated as s(inglet), d(oublet), t(riplet), q(uartet), m(ultiplet), p(seudo), and br(oad). ^13^C{^1^H}-NMR spectra are recorded in a *J*-modulated mode; signals are assigned as C, CH, CH_2_, and CH_3_. MS: ESI or EI (ESI-Qq ao TOF mass spectrometer, 70 eV (Bruker, Bremen, Germany).

Heptane (PE), dichloromethane (DCM), and ethyl acetate (EtOAc) were distilled, absolute THF from sodium benzophenone ketyl, DCM and acetonitrile from CaH_2_; BH_3_·THF was used as a 1.0 molar solution in THF, *n*-BuLi as a 2.5 molar solution in hexanes. All the other chemicals were analytical grade and used as obtained. Purification by chromatography (MPLC) was performed on a Isolera One flash purification system (Biotage, Uppsala, Sweden) with self-packed SiO_2_-cartridges (40–63 µm) and a solvent gradient. Reported procedures have been followed to obtain **7**, [21] **10**, [28,29,30] and **11** [31,32]. (*Note:* For the sake of convenience compounds **1Aa**–**1Cc** as well as precursors **5A**–**5C** have been synthesized in racemic form.)

### 3.2. Synthesis

**1Aa**–**1Cc** *(General Procedure A).* To ammonium salt **5A**–**C** (0.1 mmol) dissolved in toluene (3 mL) in a Schlenk tube was added Na_2_CO_3_ (1 mL of a 2 M solution) and arylboronic acid (0.4 mmol, dissolved in a minimum amount of EtOH). The mixture was degassed before adding Pd(PPh_3_)_4_ (20 mol%, 23 mg) and stirred at 80 °C (bath) under Ar atmosphere. After 4–20 h (TLC) DCM (10 mL) and water (10 mL) was added and the aqueous phase was extracted with DCM (10 mL). The combined organic phases were washed with KOH (20% in water, 10 mL) and sat. KBr solution (2 × 5 mL) and the solvents were evaporated. The residue was subjected to MPLC (aminopropyl-SiO_2_, MeOH(0→8%)/DCM) to give products **1Aa**–**1Cc** as pale yellow solids.

*4,4-Dibutyl-2,6-diphenyl-4,5-dihydro-3H-dinaphtho[2,1-c:1′,2′-e]azepin-4-ium bromide* (**1Aa**) [27]: Yield: 62%; m.p.: 182–184 °C. ^1^H-NMR (CDCl_3_) δ: 8.09 (s, 2H); 8.06 (br.d, *J* = 8.3 Hz, 2H); 7.64 (ddd, *J* = 8.0, 5.8, 2.2 Hz, 2H); 7.52–7.65 (br.m, ~8H); 7.46–7.52 (br.m, 2H); 7.35–7.41 (m, 4H); 5.11 (d, *J* = 14.0 Hz, 2H); 3.65 (br.dd, *J* = 14.0, 1.4 Hz, 2H); 3.17 (br.t, *J* = 13.1 Hz, 2H); 2.56 (br.td, *J* = 12.3, 4.7 Hz, 2H); 0.80–1.05 (m, 6H); 0.62 (t, *J* = 7.0 Hz, 6H); 0.12 (m, 2H) ppm. ^13^C-NMR (CDCl_3_) δ: 140.17 (C); 138.69 (C); 138.41 (C); 133.95 (C); 131.11 (CH); 130.72 (C); 129.65 (2CH); 128.53 (CH); 128.32 (CH); 128.25 (CH); 127.61 (CH); 127.40 (CH); 123.92 (C); 57.50 (CH_2_); 57.21 (CH_2_); 24.14 (CH_2_); 19.53 (CH_2_); 13.30 (CH_3_) ppm. HRMS (ESI) *m*/*z*: calcd. for C_42_H_42_N [M − Br]^+^: 560.3312, found: 560.3313.

*4,4-Dibutyl-2,6-di(naphthalen-2-yl)-4,5-dihydro-3H-dinaphtho[2,1-c:1′,2′-e]azepin-4-ium bromide* (**1Ab**): Yield: 89%; m.p.: 255–260 °C. ^1^H-NMR (CDCl_3_) δ: 8.21 (br.s, 2H); 8.03 (br.d, *J* = 8.3 Hz, 2H); 7.67 (ddd, *J* = 8.1, 5.2, 2.7 Hz, 2H); 7.50–7.61 (m, 4H); 7.37–7.48 (m, 4H); 5.10 (br.m, 2H); 3.75 (d, *J* = 13.6 Hz, 2H); 3.05 (br.t, *J* = 12.6 Hz, 2H); 2.61 (m, 2H); −0.4–0.9 (br.m, ~14H) ppm. In addition a broad band was observed (7.6–8.2 ppm) corresponding to ~10H. ^13^C-NMR (CDCl_3_) δ: 140.15 (C); 138.46 (C); 134.03 (C); 132.62 (C); 131.43 (br.CH); 130.81 (C); 129.41 (br.CH); 128.57 (CH); 128.32 (CH); 127.71 (CH); 127.46 (CH); 127.18 (CH); 127.01 (CH); 124.15 (br.C); 57.50 (br.2CH_2_); 24.19 (CH_2_); 19.42 (CH_2_); ~12.5 (br.CH_3_) ppm (4CH and 2C not observed). HRMS (ESI) *m*/*z*: calcd. for C_50_H_46_N [M − Br]^+^: 660.3625, found 660.3626.

*2,6-Di([1,1′-biphenyl]-4-yl)-4,4-dibutyl-4,5-dihydro-3H-dinaphtho[2,1-c:1′,2′-e]azepin-4-ium bromide* (**1Ac**) [27]: Yield: 82%; m.p.: 184–189 °C. ^1^H-NMR (CDCl_3_) δ: 8.13 (s, 2H); 8.05 (d, *J* = 8.3 Hz, 2H); 7.83 (br.d, *J* = 7.6 Hz, 4H); 7.61–7.73 (m, 10H); 7.42–7.49 (m, 4H); 7.34–7.42 (m, 6H); 5.19 (br.d, *J* = 13.3 Hz, 2H); 3.70 (br.d, *J* = 13.4 Hz, 2H); 3.20 (br.m, 2H); 2.61 (br.m, 2H); 0.8–1.2 (br.m, 6H); 0.55 (t, *J* = 6.6 Hz, 6H); 0.29 (br.m, 2H) ppm. ^13^C-NMR (CDCl_3_) δ: 141.08 (C); 139.73 (C); 139.68 (C); 138.44 (C); 137.45 (C); 133.94 (C); 131.10 (CH); 130.68 (C); 128.91 (CH); 128.51 (CH); 128.28 (CH); 128.16 (CH); 127.78 (CH); 127.62 (CH); 127.43 (CH); 127.07 (CH); 123.84 (C); 57.41 (CH_2_); 57.10 (CH_2_); 24.36 (CH_2_); 19.60 (CH_2_); 13.39 (CH_3_) ppm. HRMS (ESI) *m*/*z*: calcd. for C_54_H_50_N [M − Br]^+^: 712.3938, found 712.3936.

*2,6-Diphenyl-3,5-dihydrospiro[dinaphtho[2,1-c:1′,2′-e]azepine-4,1′-piperidin]-4-ium bromide* (**1Ba**): Yield: 88%; m.p.: 246–250 °C. ^1^H-NMR (CDCl_3_) δ: 8.06 (s, 2H); 8.02 (d, *J* = 8.5 Hz, 2H); 7.63 (ddd, *J* = 8.1, 6.5, 1.4 Hz, 2H); 7.56 (m, 2H); 7.55 (br.d, *J* = 7.0 Hz, 2H); 7.44–7.51 (m, 8H); 7.39 (ddd, *J* = 8.6, 6.5, 1.3 Hz, 2H); 5.06 (d, *J* = 13.3 Hz); 3.61 (d, *J* = 13.3 Hz, 2H); 3.41 (m, 2H); 2.72 (m, 2H); 1.34 (br.m, 4H); 0.73 (br.m, 2H) ppm. ^13^C-NMR (CDCl_3_) δ: 140.20 (C); 138.73 (C); 138.11 (C); 133.90 (C); 130.88 (CH); 130.61 (C); 130.15 (CH); 129.51 (CH); 128.56 (CH); 128.41 (CH); 128.22 (CH); 127.51 (CH); 127.38 (CH); 123.94 (C); 59.12 (CH_2_); 57.85 (CH_2_); 20.27 (CH_2_); 19.76 (CH_2_) ppm. HRMS (ESI) *m*/*z*: calcd. for C_39_H_34_N [M − Br]^+^: 516.2686, found: 516.2699.

*2,6-Di(naphthalen-2-yl)-3,5-dihydrospiro[dinaphtho[2,1-c:1′,2′-e]azepine-4,1′-piperidin]-4-ium bromide* (**1Bb**): Yield: 90%; m.p.: 250–256 °C (dec.). ^1^H-NMR (CDCl_3_) δ: 8.16 (s, 2H); 7.86–8.14 (br.m, 10H); 7.36–7.75 (br.m, 12H); 5.16 (d, *J* = 13.1 Hz, 2H); 3.78 (d, *J* = 13.1 Hz, 2H); 3.39 (m, 2H); 2.69 (m, 2H); 1.19 (m, 2H); 1.04 (m, 2H); 0.57 (m, 2H) ppm. ^13^C-NMR (CDCl_3_) δ: 140.18 (C); 138.24 (C); 136.20 (br.C); 134.00 (C); 133.42 (br.C); 132.61 (C); 131.34 (CH); 130.73 (C); 129.38 (br.CH); 129.34 (CH); 128.63 (CH); 128.37 (br.CH); 128.31 (CH); 127.87 (CH); 127.67 (CH); 127.62 (CH); 127.47 (CH); 127.10 (CH); 126.95 (CH); 124.14 (C); 59.40 (CH_2_); 58.37 (br.CH_2_); 20.21 (CH_2_); 19.74 (CH_2_) ppm. HRMS (ESI) *m*/*z*: calcd. for C_47_H_38_N [M − Br]^+^: 616.2999, found: 616.2994.

*2,6-Di([1,1′-biphenyl]-4-yl)-3,5-dihydrospiro[dinaphtho[2,1-c:1′,2′-e]azepine-4,1′-piperidin]-4-ium bromide* (**1Bc**): Yield: 70%; m.p.: 230–240 °C (slow dec. > 210 °C). ^1^H-NMR (CDCl_3_) δ: 8.12 (s, 2H); 8.05 (d, *J* = 8.2 Hz, 2H); 7.82 (d, *J* = 8.5 Hz, 2H); 7.82 (br.d, *J* = 6.5 Hz, 2H); 7.68–7.71 (m, 4H); 7.66 (ddd, *J* = 8.1, 6.6, 1.3 Hz, 2H); 7.59 (d, *J* = 8.2 Hz, 2H); 7.59 (m, 2H); 7.44–7.50 (m, 6H); 7.42 (ddd, *J* = 8.6, 6.7, 1.3 Hz, 2H); 7.37 (ddt, *J* = 7.8, 7.0, 1.3 Hz, 2H); 5.17 (d, *J* = 13.5 Hz, 2H); 3.69 (d, *J* = 13.4 Hz, 2H); 3.49 (m, 2H); 2.83 (m, 2H); 1.42 (m, 2H); 1.34 (m, 2H); 0.84 (m, 2H) ppm. ^13^C-NMR (CDCl_3_) δ: 141.21 (C); 139.92 (C); 138.25 (C); 137.61 (C); 133.99 (C); 130.95 (CH); 130.70 (C); 130.66 (CH); 128.91 (CH); 128.62 (CH); 129.29 (CH); 128.15 (CH); 127.78 (CH); 127.59 (CH); 127.45 (CH); 127.22 (CH); 124.08 (C); 59.25 (CH_2_); 58.00 (br.CH_2_); 20.33 (CH_2_); 19.85 (CH_2_) ppm. HRMS (ESI) *m*/*z*: calcd. for C_51_H_42_N [M − Br]^+^: 668.3312, found 668.3307.

*2,6-Diphenyl-3,5-dihydrospiro[dinaphtho[2,1-c:1′,2′-e]azepine-4,1′-pyrrolidin]-4-ium bromide* (**1Ca**): Yield: 74%; m.p.: 224–228 °C. ^1^H-NMR (CDCl_3_) δ: 8.08 (s, 2H); 8.04 (d, *J* = 8.2 Hz, 2H); 7.65 (ddd, *J* = 8.1, 6.7, 1.4 Hz, 2H); 7.56 (m, 2H); 7.54 (br.d, *J* = 6.8 Hz, 2H); 7.44–7.50 (m, 8H); 7.41 (ddd, *J* = 8.5, 6.6, 1.2 Hz, 2H); 4.85 (d, *J* = 13.5 Hz, 2H); 3.81 (d, *J* = 13.5 Hz, 2H); 3.46 (m, 2H); 2.96 (m, 2H); 1.81 (m, 2H); 1.24 (m, 2H) ppm. ^13^C-NMR (CDCl_3_) δ: 139.67 (C); 138.61 (C); 137.75 (C); 133.90 (C); 130.87 (CH); 130.68 (C); 130.10 (CH); 129.31 (CH); 128.57 (CH); 128.37 (CH); 128.13 (CH); 127.47 (CH); 127.33 (CH); 124.83 (C); 61.84 (CH_2_); 57.30 (CH_2_); 20.51 (CH_2_) ppm. HRMS (ESI) *m*/*z*: calcd. for C_38_H_32_N [M − Br]^+^: 502.2529, found: 502.2543.

*2,6-Di(naphthalen-2-yl)-3,5-dihydrospiro[dinaphtho[2,1-c:1′,2′-e]azepine-4,1′-pyrrolidin]-4-ium bromide* (**1Cb**): Yield: 81%; m.p.: 275–280 °C (dec.). ^1^H-NMR (CDCl_3_) δ: 8.17 (s, 2H); 8.07 (d, *J* = 8.4 Hz, 2H); 8.02 (d, *J* = 8.8 Hz, 2H); 8.01 (s, 2H); 7.96 (m, 2H); 7.90 (m, 2H); 7.67 (ddd, *J* = 8.1, 6.7, 1.2 Hz, 2H); 7.60 (br.d, *J* = 6.4 Hz, 2H); 7.51–7.57 (m, 6H); 7.45 (ddd, *J* = 8.4, 6.7, 1.2 Hz, 2H); 4.96 (d, *J* = 13.5 Hz, 2H); 3.95 (d, *J* = 13.4 Hz, 2H); 3.47 (m, 2H); 2.92 (m, 2H); 1.61 (m, 2H); 0.97 (m, 2H) ppm. ^13^C-NMR (CDCl_3_) δ: 139.68 (C); 137.93 (C); 136.07 (br.C); 134.03 (C); 133.26 (br.C); 132.61 (C); 131.33 (CH); 130.81 (C); 129.34 (CH); 129.24 (CH); 128.68 (CH); 128.32 (CH); 128.25 (CH); 127.87 (CH); 127.64 (CH); 127.59 (CH); 127.47 (CH); 127.14 (CH); 126.98 (CH); 125.05 (C); 61.93 (CH_2_); 57.83 (CH_2_); 20.56 (CH_2_) ppm. HRMS (ESI) *m*/*z*: calcd. for C_46_H_36_N [M − Br]^+^: 602.2842, found 602.2835. 

*2,6-Di([1,1′-biphenyl]-4-yl)-3,5-dihydrospiro[dinaphtho[2,1-c:1′,2′-e]azepine-4,1′-pyrrolidin]-4-ium bromide* (**1Cc**): Yield: 89%; glassy material. ^1^H-NMR (600 MHz, CDCl_3_) δ: 8.13 (s, 2H); 8.06 (d, *J* = 8.3 Hz, 2H); 7.80 (d, *J* = 8.2 Hz, 4H); 7.68 (br.d, *J* = 7.3 Hz, 4H); 7.66 (m, 2H); 7.57 (br.d, *J* = 7.9 Hz, 4H); 7.50 (d, *J* = 8.4 Hz, 2H); 7.44–7.47 (m, 4H); 7.43 (m, 2H); 7.37 (m, 2H); 4.96 (d, *J* = 13.5 Hz, 2H); 3.87 (d, *J* = 13.5 Hz, 2H); 3.57 (m, 2H); 3.00 (m, 2H); 1.84 (m, 2H); 1.27 (m, 2H) ppm. ^13^C-NMR (151 MHz, CDCl_3_) δ: 141.14 (C); 139.83 (C); 139.28 (C); 137.86 (C); 137.39 (C); 133.97 (C); 130.97 (CH); 130.69 (C); 130.62 (CH); 128.88 (CH); 128.64 (CH); 128.24 (CH); 128.01 (CH); 127.76 (CH); 127.54 (CH); 127.45 (CH); 127.19 (CH); 124.84 (C); 61.97 (CH_2_); 57.48 (CH_2_); 20.60 (CH_2_) ppm. HRMS (ESI) *m*/*z*: calcd. for C_50_H_40_N [M − Br]^+^: 654.3155, found: 654.3134.

*Spiro* cyclization of (*S*)-**9a**–**c** with (*S*)-2,2′-bisbromomethyl-1,1′-binaphthyl **10** yielding (*S*,*S*)-**2a**–**c**, respectively. (*General Procedure B):* A Schlenk tube, equipped with magnetic stirring bar and glass stopper, was charged with a solution of substrate (0.3 mmol) in MeCN (6 mL) and K_2_CO_3_ (83 mg, 2 eq) followed by (*S*)-2,2′-bis(bromomethyl)-1,1′-binaphthyl **10** (132 mg, 0.3 mmol) and the mixture was degassed. The reaction was left stirring at 85–90 °C (bath) for 24 h. After cooling to r.t., DCM (30 mL) and H_2_O (30 mL) were added and the phases were separated. The aqueous one was extracted with DCM (3 × 15 mL) and the combined organic extracts were evaporated under reduced pressure. The crude material was purified by MPLC using a solvent gradient (MeOH(0→8%)/DCM).

*(S,S)-2,6-Diphenyl-3,3′,5,5′-tetrahydro-4,4′-spirobi[dinaphtho[2,1-c:1′,2′-e]azepin]-4-ium bromide* (**2a**) [8]: Yield: 76%. ^1^H-NMR (CDCl_3_) δ: 8.33 (s, 2H); 8.10 (br.d, *J* = 8.2 Hz, 2H); 7.83 (d, *J* = 8.1 Hz, 2H); 7.74 (m, 2H); 7.62 (ddd, *J* = 7.9, 6.5, 1.1 Hz, 2H); 7.48 (ddd, *J* = 7.9, 6.6, 1.1 Hz, 2H); 7.32 (ddd, *J* = 8.3, 6.8, 1.1 Hz, 2H); 7.32 (d, *J* = 8.5 Hz, 2H); 7.18 (ddd, *J* = 8.1, 6.8, 1.2 Hz, 2H); 7.12 (d, *J* = 8.6 Hz, 2H); 7.09 (d, *J* = 8.4 Hz, 2H); 6.32 (d, *J* = 8.4 Hz, 2H); 5.00 (d, *J* = 13.8 Hz, 2H); 4.38 (d, *J* = 13.7 Hz, 2H); 4.21 (d, *J* = 13.4 Hz, 2H); 3.69 (d, *J* = 13.3 Hz) ppm. In addition a broad band was observed (7.4–8.2 ppm) corresponding to ~8H. ^13^C-NMR (CDCl_3_) δ: 139.27 (C); 139.13 (C); 136.15 (C); 133.95 (C); 133.85 (C); 132.69 (CH); 131.02 (C); 130.96 (C); 130.89 (br. CH); 130.15 (br. CH); 129.04 (CH); 128.74 (CH); 128.52 (CH); 128.33 (CH); 128.18 (CH); 127.51 (2CH); 127.42 (CH); 127.34 (CH); 126.87 (CH); 126.70 (CH); 124.81 (C); 122.36 (C); 62.33 (CH_2_); 57.41 (CH_2_) ppm (1C not observed). HRMS (ESI) calcd. for C_56_H_40_N [M − Br]^+^: 726.3161, found: 726.3171.

*(S,S)-2,6-Di(naphthalen-2-yl)-3,3′,5,5′-tetrahydro-4,4′-spirobi[dinaphtho[2,1-c:1′,2′-e]azepin]-4-ium bromide* (**2b**) [8]: Yield: 88%; m.p.: 255–258 °C (dec.); ${\left[\alpha \right]}_{D}^{20}$ = +55 (c: 0.70, DCM). ^1^H-NMR (CDCl_3_) δ: 8.47 (s, 2H); 8.15 (d, *J* = 8.1 Hz, 2H); 8.13 (br.m, 2H); 7.77 (br.m, 2H); 7.65 (ddd, *J* = 8.1, 6.8, 1.1 Hz, 2H); 7.30–7.42 (m, 6H); 7.19 (br.d, *J* = 8.7 Hz, 2H); 7.07 (ddd, *J* = 8.3, 6.7, 1.4 Hz, 2H); 6.94 (d, *J* = 8.7 Hz, 2H); 6.06 (br.m, 4H); 5.06 (br.m, 2H); 4.49 (d, *J* = 13.8 Hz, 2H); 4.20 (d, *J* = 13.2 Hz, 2H); 3.65 (d, *J* = 13.2 Hz, 2H) ppm. In addition a broad band was observed (7.5–9.1 ppm); integration corresponding to ~10H. ^13^C-NMR (CDCl_3_) δ: 139.43 (C); 139.28 (C); 135.92 (C); 134.08 (C); 133.68 (C); 133.19 (C); 132.79 (br.CH); 131.27 (C); 130.75 (C); 129.97 (br.CH); 128.86 (br.CH); 128.57 (2CH); 128.40 (CH); 128.31 (br.CH); 127.95 (CH); 127.75 (br.CH); 127.59 (CH); 127.56 (CH); 127.30 (3CH); 127.19 (CH); 126.78 (br.CH); 126.56 (CH); 124.78 (C); 122.84 (C); 62.40 (CH_2_); 57.61 (br.CH_2_) ppm (1CH and 2C not observed). HRMS (ESI) calcd. for C_64_H_44_N [M − Br]^+^: 826.3474, found: 826.3471.

*(S,S)-2,6-Di([1,1′-biphenyl]-4-yl)-3,3′,5,5′-tetrahydro-4,4′-spirobi[dinaph-tho[2,1-c:1′,2′-e]azepin]-4-ium bromide* (**2c**): Yield: 81%; m.p.: 252–255 °C (dec.); ${\left[\alpha \right]}_{D}^{20}$ = +140 (c: 0.81, DCM). ^1^H-NMR (CDCl_3_) δ: 8.40 (s, 2H); 8.12 (d, *J* = 7.9 Hz, 2H); 7.84 (m, 4H); 7.57–7.67 (m, 8H); 7.53 (m, 2H); 7.43 (ddd, *J* = 8.1, 6.7, 1.1 Hz, 2H); 7.32 (ddd, *J* = 8.2, 6.8, 1.1 Hz, 2H); 7.13–7.20 (m, 6H); 7.08 (d, *J* = 8.4 Hz, 2H); 6.41 (d, *J* = 8.4 Hz, 2H); 5.08 (d, *J* = 13.8 Hz, 2H); 4.47 (d, *J* = 13.8 Hz, 2H); 4.27 (d, *J* = 13.4 Hz, 2H); 3.76 (d, *J* = 13.4 Hz, 2H) ppm; in addition a broad band was observed (7.4–8.5 ppm); integration corresponding to ~8H. ^13^C-NMR (CDCl_3_) δ: 141.76 (C); 140.18 (C); 139.40 (C); 138.77 (C); 138.18 (C); 136.24 (C); 134.05 (C); 133.84 (C); 132.42 (CH); 131.48 (br. CH); 131.22 (C); 131.03 (C); 129.27 (CH); 128.82 (CH); 128.77 (CH); 128.58 (CH); 128.34 (CH); 128.11 (CH); 128.00 (CH); 127.54 (2CH); 127.41 (CH); 127.32 (CH); 126.83 (CH); 126.71 (CH); 125.05 (C); 122.67 (C); 62.51 (CH_2_); 57.43 (CH_2_) ppm (1CH not observed). HRMS (ESI) calcd. for C_68_H_48_N [M − Br]^+^: 878.3781, found: 878.3781.

*Spiro* cyclization of (*S*)-**9a**–**c** with 2,2′-bisbromomethyl-1,1′-biphenyl **10** yielding (*S*)-**3a**–**c**; a similar procedure as for the synthesis of (*S*,*S*)-**2a**–**c** was applied.

*(S)-2′,6′-Diphenyl-3′,5,5′,7-tetrahydrospiro[dibenzo[c,e]azepine-6,4′-dinaphtho[2,1-c:1′,2′-e]azepin]-6-ium bromide* (**3a**): Yield: 89%; m.p.: 275–286 °C (dec.); ${\left[\alpha \right]}_{D}^{20}$ = +12 (c: 0.51, DCM). ^1^H-NMR (500 MHz, DMSO-*d*_6_, 378 K) δ: 8.28 (s, 2H); 8.25 (d, *J* = 8.1 Hz, 2H); 7.72 (br.ddd, *J* = 8.0, 6.7, 1.0 Hz, 2H); 7.1–7.6 (br.m, 22H); 4.66 (br.d, *J* = 13.2 Hz, 2H); 4.52 (d, *J* = 13.3 Hz, 2H); 4.16 (br.s, 2H); 2.87 (br.s, 2H) ppm. ^13^C-NMR (125.8 MHz, DMSO-*d*_6_, 378 K) δ: 139.67 (C); 138.96 (C); 138.41 (C); 137.58 (C); 133.32 (C); 130.44 (CH); 130.31 (C); 130.20 (CH); 129.57 (CH); 128.25 (CH); 128.08 (CH); 127.83 (CH); 127.49 (CH); 127.39 (CH); 127.31 (CH); 126.61 (CH); 126.57 (CH); 126.27 (C); 123.85 (br.C); 61.30 (CH_2_); 57.74 (CH_2_) ppm. HRMS (ESI) calcd. for C_48_H_36_N [M − Br]^+^: 626.2842, found: 626.2839.

*(S)-2′,6′-Di(naphthalen-2-yl)-3′,5,5′,7-tetrahydrospiro[dibenzo[c,e]azepine-6,4′-dinaphtho[2,1-c:1′,2′-e]-azepin]-6-ium bromide* (**3b**): Yield: 92%; m.p.: 264–267 °C (dec.); ${\left[\alpha \right]}_{D}^{20}$ = −21 (c: 0.35, DCM). ^1^H-NMR (500 MHz, DMSO-*d*_6_, 378 K) δ: 8.46 (s, 2H); 8.30 (d, *J* = 8.3 Hz, 2H); 8.18 (br.s, 2H); 8.00 (br.d, *J* = 8.2 Hz, 2H); 7.96 (br.d, *J* = 7.5 Hz, 2H); 7.92 (br.d, *J* = 7.3 Hz, 2H); 7.75 (ddd, *J* = 8.0, 7.0, 1.0 Hz, 2H); 7.73 (br.s, 2H); 7.58 (m, 4H); 7.51 (ddd, *J* = 8.2, 7.0, 1.1 Hz, 2H); 7.36 (br.d, *J* = 8.3 Hz, 2H); 7.23 (m, 4H); 6.76 (br.s, 2H); 6.57 (br.s, 2H); 4.85 (d, *J* = 13.9 Hz, 2H); 4.53 (d, *J* = 13.8 Hz, 2H); 3.97 (br.s, 2H); 3.13 (br.s, 2H) ppm. ^13^C-NMR (125.8 MHz, DMSO-*d*_6_, 378 K) δ: 139.48 (C); 138.86 (C); 137.88 (br.C); 135.79 (C); 133.34 (C); 132.46 (br.C); 131.82 (C); 130.81 (CH); 130.37 (C); 130.14 (CH); 128.92 (CH); 128.24 (CH); 127.99 (CH); 127.50 (CH); 127.42 (CH); 127.35 (CH); 126.96 (CH); 126.74 (CH); 126.59 (CH); 126.24 (2CH); 125.98 (C); 123.70 (br.C); 61.10 (CH_2_); 57.56 (CH_2_) ppm (3CH not observed). HRMS (ESI) calcd. for C_56_H_40_N [M − Br]^+^: 726.3155, found: 726.3145.

*(S)-2′,6′-Di([1,1′-biphenyl]-4-yl)-3′,5,5′,7-tetrahydrospiro[dibenzo[c,e]aze-pine-6,4′-dinaphtho[2,1-c:1′,2′-e]azepin]-6-ium bromide* (**3c**): Yield: 84%; glassy material. ${\left[\alpha \right]}_{D}^{20}$ = +9 (c: 0.61, DCM). ^1^H-NMR (500 MHz, DMSO-*d*_6_, 378 K) δ: 8.37 (s, 2H); 8.28 (d, *J* = 8.2 Hz, 2H); 7.55–7.80 (m, 14H); 7.31–7.54 (m, 14H); 7.17 (br.s, 4H); 4.79 (br.d, *J* = 13.0 Hz, 2H); 4.51 (d, *J* = 13.8 Hz, 2H); 4.10 (br.s, 2H); 3.08 (br.s, 2H) ppm. ^13^C-NMR (125.8 MHz, DMSO-*d*_6_, 378 K) δ: 139.75 (C); 139.73 (C); 138.89 (C); 138.53 (C); 137.72 (C); 137.38 (C); 133.33 (C); 130.34 (C); 130.32 (CH); 130.27 (CH); 128.39 (CH); 128.25 (CH); 127.59 (br.CH); 127.51 (CH); 127.36 (CH); 127.17 (CH); 126.67 (CH); 126.57 (CH); 126.48 (CH); 126.29 (C); 126.11 (2CH); 123.79 (br.C); 61.21 (CH_2_); 57.49 (CH_2_) ppm (1CH not observed). HRMS (ESI) calcd for C_60_H_44_N [M − Br]^+^: 778.3468, found: 778.3441.

*(S)-4,8-Diphenyl-3′,5,5′,7-tetrahydrospiro[dibenzo[c,e]azepine-6,4′-dinaphtho[2,1-c:1′,2′-e]azepin]-6-ium bromide* (**4a**) [14]: Yield: 82%; m.p.: 225–228 °C; ${\left[\alpha \right]}_{D}^{20}$ = +225 (c: 0.49, DCM). ^1^H-NMR (CDCl_3_) δ: 6.66–8.10 (several broad m, ~24H), 7.92 (br.d, *J* = 8.3 Hz, ~2H); 7.18 (br.m, *J* = 8.8 Hz, ~2H); 4.60–4.75 (m, 4H); 4.43 (d, *J* = 13.3 Hz, 2H); 2.46–3.10 (br.m, 2H) ppm. ^13^C-NMR (CDCl_3_) δ: 143.52 (C); 142.57 (br.C); 138.92 (C); 136.27 (C); 134.00 (C); 130.95 (CH); 130.65 (C); 129.60 (br.CH); 129.53 (CH); 129.11 (CH); 128.33 (CH), 127.57 (br.CH); 127.21 (CH); 127.11 (2CH); 126.74 (CH); 126.09 (br.C); 124.16 (br.C); 63.17 (CH_2_); 57.86 (CH_2_) ppm; (2CH not observed). HRMS (ESI) calcd. for C_48_H_36_N [M − Br]^+^: 626.2842, found: 626.2828.

*(S)-4,8-Di(naphthalen-2-yl)-3′,5,5′,7-tetrahydrospiro[dibenzo[c,e]azepine-6,4′-dinaphtho[2,1-c:1′,2′-e]azepin]-6-ium bromide* (**4b**) [14]: Yield: 80%; m.p.: 238–241 °C (dec.); ${\left[\alpha \right]}_{D}^{20}$ = +228 (c: 0.54, DCM). ^1^H-NMR (CDCl_3_) δ: 7.29–8.23 (several broad m, ~26H), 7.35 (m, 2H); 7.05 (br.ddd, *J* = 8.5, 6.9, 1.0 Hz, 2H); 6.90 (d, *J* = 8.5 Hz, 2H); 4.76–4.92 (br.m, 2H); 4.48–4.65 (br.m, 4H); 2.73–3.25 (br.m, 2H) ppm. ^13^C-NMR (CDCl_3_) δ: 143.52 (C); 142.87 (br.C); 136.31 (br.C); 135.95 (C); 133.62 (C); 132.96 (br.C); 132.37 (br.C); 131.05 (CH); 130.40 (C); 129.39 (CH); 128.95 (several br.CH); 128.07 (CH); 127.64 (br.CH); 127.35 (CH); 126.97 (CH); 126.88 (CH); 126.78 (CH); 126.70 (CH); 126.59 (CH); 126.48 (CH); 125.48 (br.C); 124.11 (br.C); 62.96 (CH_2_); 57.55 (br.CH_2_) ppm. HRMS (ESI) calcd. for C_56_H_40_N [M − Br]^+^: 726.3155, found: 726.3145.

*(S)-4,8-Di([1,1′-biphenyl]-4-yl)-3′,5,5′,7-tetrahydrospiro[dibenzo[c,e]azepine-6,4′-dinaphtho[2,1-c:1′,2′-e]azepin]-6-ium bromide* (**4c**): Yield: 75%; m.p.: 236–241 °C; ${\left[\alpha \right]}_{D}^{20}$ = +211 (c: 0.62, DCM). ^1^H-NMR (500 MHz, DMSO-*d*_6_, 378 K) δ: 7.98 (br.m, 2H); 7.93 (br.d, *J* = 8.3 Hz, 2H); 7.88 (s, 2H); 7.85–7.90 (m, 2H); 7.62–7.66 (m, 2H); 7.58 (br.m, 2H); 7.46 (ddd, *J* = 8.0, 6.8, 1.0 Hz, 2H); 7.23–7.45 (br.m, ~14H); 7.21 (ddd, *J* = 8.2, 6.8, 1.1 Hz, 2H); 7.06 (d, *J* = 8.5 Hz, 2H); 7.04 (br.m, 4H); 4.49 (d, *J* = 13.3 Hz, 2H); 4.48 (br.m, 2H); 4.35 (d, *J* = 13.3 Hz, 2H); 2.65 (br.m, 2H) ppm. ^13^C-NMR (125.8 MHz, DMSO-*d*_6_, 378 K) δ: 142.60 (C); 141.81 (C); 139.77 (C); 138.81 (C); 137.08 (C); 135.38 (C); 133.17 (C); 130.31 (CH); 129.75 (br.CH); 129.64 (C); 129.43 (CH); 128.64 (CH); 128.37 (CH); 128.03 (CH); 127.70 (CH); 126.83 (CH); 126.70 (CH); 126.44 (CH); 126.24 (CH); 125.97 (2CH); 125.87 (C); 125.69 (CH); 124.33 (C); 62.16 (CH_2_); 56.78 (CH_2_) ppm. HRMS (ESI) calcd for C_60_H_44_N [M − Br]^+^: 778.3468, found: 778.3451.

**5A**–**C** *(General Procedure C)* A round-bottomed flask (10 mL) was charged with racemic dibromide **7** (0.2 mmol) and secondary amine (0.8 mmol for **5A** and 0.4 mmol for **5B** and **5C**, respectively) in MeCN (4 mL). To this was added K_2_CO_3_ (110 mg, 0.8 mmol) and the reaction was stirred for 22–24 h at 80 °C. Volatiles were removed under vaccum and the residue was suspended in water (2 mL). The precipitate was separated, washed with water (2 mL), Et_2_O (2 × 2 mL) and air-dried to give a pale yellow solid which was found to be pure by ^1^H-NMR.

*4,4-Dibutyl-2,6-diiodo-4,5-dihydro-3H-dinaphtho[2,1-c:1′,2′-e]azepin-4-ium bromide* (**5A**): Yield: 86%; m.p.: 220–225 °C. ^1^H-NMR (CDCl_3_) δ: 8.70 (s, 2H); 7.89 (d, *J* = 8.2 Hz, 2H); 7.59 (ddd, *J* = 8.0, 6.9, 1.0 Hz, 2H); 7.32 (ddd, *J* = 8.2, 6.9, 1.2 Hz, 2H); 7.09 (d, *J* = 8.7 Hz, 2H); 5.12 (d, *J* = 13.9 Hz, 2H); 3.97 (d, *J* = 13.7 Hz, 2H); 3.52 (m, 4H); 2.18 (m, 2H); 1.83 (m. 2H); 1.51 (m, 4H); 1.02 (t, *J* = 7.4 Hz, 6H) ppm. ^13^C-NMR (CDCl_3_) δ: 141.36 (CH); 137.89 (C); 135.31 (C); 130.88 (C); 128.87 (CH); 128.16 (CH); 127.71 (C); 127.47 (CH); 127.44 (CH); 97.56 (C); 66.01 (CH_2_); 59.60 (CH_2_); 25.44 (CH_2_); 19.97 (CH_2_); 13.69 (CH_3_) ppm. HRMS (ESI) *m*/*z*: Calcd. for C_30_H_32_I_2_N [M − Br]^+^: 660.0619, found: 660.0610.

*2,6-Diiodo-3,5-dihydrospiro[dinaphtho[2,1-c:1′,2′-e]azepine-4,1′-piperidin]-4-ium bromide* (**5B**): Yield: 85%; m.p.: ~290 °C (dec.). ^1^H-NMR (CDCl_3_) δ: 8.71 (s, 2H); 7.89 (d, *J* = 8.3 Hz, 2H); 7.60 (ddd, *J* = 8.0, 6.9, 1.1 Hz, 2H); 7.33 (ddd, *J* = 8.2, 6.9, 1.2 Hz, 2H); 7.10 (dm, *J* = 8.6 Hz, 2H); 5.42 (d, *J* = 13.9 Hz); 4.11 (m, 2H); 3.88 (m, 2H); 3.76 (d, *J* = 13.8 Hz, 2H); 2.38 (m, 2H); 2.10 (m, 2H); 2.02 (m, 2H) ppm. ^13^C-NMR (CDCl_3_) δ: 141.42 (CH); 137.97 (C); 135.27 (C); 130.69 (C); 128.84 (CH); 128.14 (CH); 128.06 (C); 127.40 (CH); 96.99 (C); 65.34 (CH_2_); 59.23 (CH_2_); 21.80 (CH_2_); 20.42 (CH_2_) ppm. HRMS (ESI) *m*/*z*: Calcd. for C_27_H_24_I_2_N [M − Br]^+^: 615.9993, found: 616.0016.

*2,6-Diiodo-3,5-dihydrospiro[dinaphtho[2,1-c:1′,2′-e]azepine-4,1′-pyrrolidin]-4-ium bromide* (**5C**): Yield: 94%; m.p.: 234–238 °C. ^1^H-NMR (CDCl_3_) δ: 8.71 (s, 2H); 7.90 (ddd, *J* = 8.0, 6.8, 1.0 Hz, 2H); 7.35 (ddd, *J* = 8.2, 6.9, 1.3 Hz, 2H); 7.14 (br.d, *J* = 8.5 Hz, 2H); 5.25 (d, *J* = 13.8 Hz, 2H); 4.24 (m, 2H); 4.09 (m, 2H); 3.92 (d, *J* = 13.7 Hz, 2H); 2.62 (m, 2H); 2.51 (m, 2H) ppm. ^13^C-NMR (CDCl_3_) δ: 141.46 (CH); 137.65 (C); 135.34 (C); 130.75 (C); 128.80 (CH); 128.66 (C); 128.17 (CH); 127.49 (CH); 127.39 (CH); 96.56 (C); 65.03 (CH_2_); 62.55 (CH_2_); 22.16 (CH_2_) ppm. HRMS (ESI) *m*/*z*: Calcd. for C_26_H_22_I_2_N [M − Br]^+^: 601.9836, found 601.9861.

*(S)-2,2′-Bis(bromomethyl)-3,3′-diiodo-1,1′-binaphthalene* (**7**) [21]: (*S*)-2,2′-Bis(hydroxymethyl)-3,3′-diiodo-1,1′-binaphthalene (2.971 g, 5.247 mmol) was suspended in HBr (30% in HOAc, 50 mL) and heated to 90 °C for 1.5 h. The cooled reaction mixture was poured into water (500 mL) and sufficient DCM was added to dissolve the precipitate (usually 350–400 mL). The aqueous layer was extracted with DCM (2 × 50 mL) and the combined organic phases washed with water, sat. NaHCO_3_, and sat. NaCl and dried (MgSO_4_). Evaporation afforded crude **7** as a pale yellow crystalline precipitate; yield: 3.531 g (96%, 99% purity by NMR). The material was pure enough for the next step.

*(S)-2,6-Diiodo-4,5-dihydro-3H-dinaphtho[2,1-c:1′,2′-e]azepine* (**8**): In a pressure tube with stirring bar dibromide **7** (1 mmol, 692 mg) was suspended in aqueous NH_3_ (25%, 15 mL) and acetonitrile (25 mL) and the mixture was heated to 60 °C (oil bath) with stirring for 24 h. The pressure tube was opened at r.t. and the slurry was transferred to a 250 mL flask and ammonia and in part acetonitrile was evaporated. To the residue was added KOH (50 mL, 5% in water) and DCM (50 mL) to obtain a clear 2-phase system. The alkaline phase was extracted with DCM (2 × 20 mL) and the combined organic phase was washed neutral, dried (K_2_CO_3_) and evaporated to yield 495–520 mg (86–90%) of crude **8** (95% purity by NMR) which was pure enough for subsequent reactions. Further purification by chromatography (EtOAc/heptane, 50:50) yielded a crystalline product, m.p.: 239–241 °C; ${\left[\alpha \right]}_{D}^{20}$ = +231 (c: 1.0, EtOH). ^1^H-NMR (CDCl_3_) δ: 8.58 (s, 2H); 7.81 (d, *J* = 8.3 Hz, 2H); 7.45 (m, 2H); 7.26 (m, 4H); 4.35 (d, *J* = 12.8 Hz, 2H); 3.34 (d, *J* = 12.8 Hz, 2H); ~2.10 (br.s, 1H) ppm. ^13^C-NMR (CDCl_3_) δ: 139.78 (CH); 136.05 (C); 135.63 (C); 134.17 (C); 130.80 (C); 127.38 (CH); 127.14 (CH); 126.61 (CH); 126.42 (CH); 97.74 (C); 52.57 (CH_2_) ppm. HRMS (ESI) calcd. for C_22_H_16_I_2_N [M + H]^+^: 547.9372, found: 547.9367.

*(11bS,11b′S)-4,4′-(((S)-3,3′-Diiodo-[1,1′-binaphthalene]-2,2′-diyl)bis(methylene))bis(2,6-diiodo-4,5-dihydro-3H-dinaphtho[2,1-c:1′,2′-e]azepine)* (**8X**) (by-product). m.p.: 233–236 °C (dec.). ^1^H-NMR (CDCl_3_, 600 MHz) δ: 8.54 (s, 2H); 8.43 (s, 4H); 7.74 (d, *J* = 8.2 Hz, 4H); 7.49 (m, 2H); 7.37–7.42 (m, 6H); 7.16 (m, 4H); 7.01–7.10 (m, 8H); 4.37 (d, *J* = 13.7 Hz, 2H); 3.79 (d, *J* = 13.6 Hz) ppm; In addition a broad band was observed (2.3–3.5 ppm); integration corresponding to ~4H. ^13^C-NMR (CDCl_3_, 151 MHz) δ: 139.81 (C); 139.00 (CH); 138.92 (CH); 136.22 (C); 134.06 (C); 134.02 (C); 133.88 (C); 130.73 (C); 129.16 (CH); 127.30 (CH); 127.03 (CH); 126.28 (CH); 126.10 (2CH); 125.76 (CH); 125.36 (CH); 99.50 (C); 98.74 (C); 62.68 (CH_2_) ppm (1CH_2_ not observed). HRMS (ESI) calcd. for C_66_H_43_I_6_N_2_ [M + H]^+^: 1624.7694, found: 1624.7684.

Suzuki-Miyaura coupling of (*S*)-**8** yielding (*S*)-**9a**–**9c** *(General Procedure D):* A Schlenk tube, equipped with magnetic stirring bar and glass stopper, was charged with a solution of diiodoazepine (*S*)-**8** (274 mg, 0.50 mmol) in toluene (10 mL) and a Na_2_CO_3_-solution (2 M in H_2_O, 5.0 mL). Then arylboronic acid (2.0 mmol, 4 eq) in a minimum amount of EtOH (~2 mL) was added and the mixture was degassed. After the addition of Pd(PPh_3_)_4_ (115 mg, 20 mol %), the reaction was left stirring at 80 °C for 48 h. The reaction was monitored by TLC (EtOAc/heptane, 30/70). After cooling to r.t., DCM (50 mL) and H_2_O (30 mL) were added and the phases were separated. The aqueous phase was extracted with DCM (3 × 20 mL). The combined organic phases were washed with KOH solution (10%, 20 mL) and sat. NaCl solution and dried (K_2_CO_3_). After evaporation of solvents the crude material was purified by MPLC using a solvent gradient (EtOAc + 5% triethylamine(0→30%)/heptane).

*(S)-2,6-Diphenyl-4,5-dihydro-3H-dinaphtho[2,1-c:1′,2′-e]azepine* (**9a**) [33]: Yield: 77%; glassy material; ${\left[\alpha \right]}_{D}^{20}$ = +294 (c: 0.56, DCM). ^1^H-NMR (CDCl_3_) δ: 7.95 (s, 2H); 7.94 (br.d, *J* = 8.2 Hz, 2H); 7.60 (m, 4H); 7.38–7.50 (m, 10H); 7.27 (ddd, *J* = 8.5, 6.7, 1.2 Hz, 2H); 4.00 (d, *J* = 12.5 Hz, 2H); 3.36 (d, *J* = 12.5 Hz, 2H) ppm. ^13^C-NMR (CDCl_3_) δ: 141.35 (C); 139.75 (C); 136.00 (C); 133.37 (C); 132.44 (C); 130.80(C); 129.65 (CH); 129.60 (CH); 128.26 (CH); 128.19 (CH); 127.53 (CH); 127.16 (CH); 125.76 (CH); 125.69 (CH); 44.58 (CH_2_) ppm. HRMS (ESI) calcd for C_34_H_26_N [M + H]^+^: 448.2065, found: 448.2054.

*(S)-2,6-Di(naphthalen-2-yl)-4,5-dihydro-3H-dinaphtho[2,1-c:1′,2′-e]azepine* (**9b**): Yield: 68%; glassy material; ${\left[\alpha \right]}_{D}^{20}$ = +221 (c: 0.48, DCM). ^1^H-NMR (CDCl_3_) δ: 8.08 (br.d, *J* = 1.2 Hz, 2H); 8.06 (s, 2H); 7.89–8.00 (m, 8H); 7.75 (dd, *J* = 8.3, 1.5 Hz, 2H); 7.48–7.56 (m, 8H); 7.31 (m, 2H); 4.08 (d, *J* = 12.6 Hz, 2H); 3.44 (d, *J* = 12.6 Hz, 2H) ppm. ^13^C-NMR (CDCl_3_) δ: 139.70 (C); 138.93 (C); 136.10 (C); 133.53 (C); 133.35 (C); 132.53 (C); 132.50 (C); 130.93 (C); 129.92 (CH); 128.32 (2CH); 128.15 (CH); 128.09 (CH); 127.72 (CH); 127.62 (CH); 127.57 (CH); 126.32 (CH); 126.01 (CH); 125.83 (CH); 125.79 (CH); 44.74 (CH_2_) ppm. HRMS (ESI) calcd. for C_42_H_30_N [M + H]^+^: 548.2378, found: 548.2383.

*(S)-2,6-Di([1,1′-biphenyl]-4-yl)-4,5-dihydro-3H-dinaphtho[2,1-c:1′,2′-e]azepine* (**9c**): Yield: 68%; m.p.: 185–190 °C; ${\left[\alpha \right]}_{D}^{20}$ = +274 (c: 0.49, DCM). ^1^H-NMR (CDCl_3_) δ: 8.01 (s, 2H); 7.97 (br.d, *J* = 8.2 Hz, 2H); 7.66–7.73 (m, 12H); 7.45–7.52 (m, 8H); 7.38 (m, 2H); 7.29 (ddd, *J* = 8.6, 6.8, 1.4 Hz, 2H); 4.10 (d, *J* = 12.5 Hz, 2H); 3.41 (d, *J* = 12.5 Hz, 2H) ppm. ^13^C-NMR (CDCl_3_) δ: 140.77 (C); 140.30 (C); 140.03 (C); 139.32 (C); 136.06 (C); 133.33 (C); 132.46 (C); 130.82 (C); 130.10 (CH); 129.65 (CH); 128.83 (CH); 128.29 (CH); 127.54 (CH); 127.35 (CH); 127.12 (CH); 126.94 (CH); 125.81 (CH); 125.76 (CH); 44.65 (CH_2_) ppm. HRMS (ESI) calcd. for C_46_H_34_N [M + H]^+^: 600.2686, found: 600.2704.

*3,3′-Bis(trimethylsilyl)-[1,1′-biphenyl]-2,2′-dicarboxylic acid:* A solution of 2,2,6,6-teramethylpiperidine (1.730 g, 12 mmol, 2.08 mL) in THF (20 mL) was degassed and cooled to 0 °C. To this was added *n*-BuLi (4.80 mL of a 2.5 molar solution, 12 mmol) and stirring was continued for 20 min. The reaction was cooled to −78 °C and Me_3_SiCl (2.53 mL, 20 mmol) was added followed by dropwise addition of diphenic acid (484 mg, 2 mmol) in degassed THF (10 mL) during 30 min. The mixture was allowed to reach r.t. overnight. For work-up HCl (4 M, 20 mL) was carefully added with stirring followed by Et_2_O (40 mL). The aqueous phase was extracted with Et_2_O (20 mL) and the combined organic phases stirred with NaOH (1 molar, 40 mL) for 15 min. The organic phases was washed another time with NaOH and the alkaline extracts washed with ether (20 mL) and acidified (HCl, 6 M). The mixture was extracted with Et_2_O (2 × 100 mL) and the combined organic phase was washed with brine and dried (MgSO_4_). Evaporation of solvent left 704 mg (90%) of 3,3′-bis(trimethylsilyl)-[1,1′-biphenyl]-2,2′-dicarboxylic acid as an off-white powder which was pure enough for the next step (99% by NMR). M.p.: 199–202 °C. ^1^H-NMR (CDCl_3_) δ: 7.62 (dd, *J* = 7.5, 1.2 Hz, 2H); 7.39 (t, *J* = 7.5 Hz, 2H); 7.20 (dd, *J* = 7.6, 1.2 Hz, 2H); 0.32 (s, 18H) ppm. ^13^C-NMR (CDCl_3_) δ: 173.71 (C); 138.73 (C); 138.08 (C); 137.47 (C); 134.42 (CH); 130.15 (CH); 129.22 (CH); 0.24 (CH_3_) ppm. HRMS (ESI) calcd. for C_20_H_25_O_4_Si_2_ [M − H]^−^: 385.1297, found 385.1298.

*(3,3′-Bis(trimethylsilyl)-[1,1′-biphenyl]-2,2′-diyl)dimethanol:* To a degassed solution of 3,3′-bis(trimethylsilyl)-[1,1′-biphenyl]-2,2′-dicarboxylic acid (3.67 g, 9.50 mmol) in THF (180 mL) was added BH_3_·THF complex (38 mL of a 1 M solution in THF, 38 mmol, 4 eq.) by syringe and the reaction was refluxed under Ar for 20–24 h (TLC control). Diluted HCl (2 M) was carefully added at 0 °C to decompose excess of BH_3_. After removing bulk of THF the residue was partioned between HCl (2 M, 200 mL) and DCM (400 mL). The aqueous phase was extracted with more DCM (100 mL and 50 mL) and the combined organic phase was washed with water and brine and dried (MgSO_4_). Evaporation of solvents and drying under vacuum overnight left 3.299 g (95% purity by ^1^H-NMR, 92% yield) of (3,3′-bis(trimethylsilyl)-[1,1′-biphenyl]-2,2′-diyl)dimethanol which was pure enough for the next step. M.p.: 171–175 °C. ^1^H-NMR (CDCl_3_) δ: 7.59 (dd, *J* = 7.5, 1.5 Hz, 2H); 7.32 (t, *J* = 7.5 Hz, 2H); 7.17 (dd, *J* = 7.5, 1.4 Hz, 2H); 4.54 (d, *J* = 11.5 Hz, 2H); 4.50 (d, *J* = 11.5 Hz, 2H); 2.56 (br.s, 2H); 0.40 (s, 18H) ppm. ^13^C-NMR (CDCl_3_) δ: 143.43 (C); 141.77 (C); 141.00 (C); 134.44 (CH); 130.94 (CH); 126.83 (CH); 61.83 (CH_2_); 0.83 (CH_3_) ppm. HRMS (ESI) calcd. for C_20_H_31_O_2_Si_2_ [M + Na]^+^: 381.1682, found: 381.1677.

*(3,3′-Diiodo-[1,1′-biphenyl]-2,2′-diyl)dimethanol:* A solution of ICl (4.14 g, 25.5 mmol, 3 eq.) in DCM (100 mL) was dropwise added to (3,3′-bis(trimethylsilyl)-[1,1′-biphenyl]-2,2′-diyl)dimethanol (3.21 g, 8.5 mmol, 95% purity from the previous step) in DCM (140 mL) at −40 °C and the reaction was stirred at the same temperature for 2 h. A solution of NaHSO_3_ (100 mL, 10%) was added with vigorous stirring and after formation of a semifrozen slury the mixture was warmed up to r.t. The crystalline product was separated, washed with some water and Et_2_O and dried under vacuum to give 3.435 g of product. The organic phase was separated, washed with water and dried (MgSO_4_) and evaporated. The residue was treated with DCM/heptane (1:1, 10 mL) to leave a white precipitate which was pure product as well, giving a total yield of 3.763 g (95%) of (3,3′-diiodo-[1,1′-biphenyl]-2,2′-diyl)dimethanol. M.p.: 230–233 °C. ^1^H-NMR (CDCl_3_) δ: 7.94 (dd, *J* = 7.9, 1.3 Hz, 2H); 7.11 (dd, *J* = 7.6, 1.3 Hz, 2H); 7.02 (t, *J* = 7.7 Hz, 2H); 4.52 (d, *J* = 12.2 Hz, 2H); 4.36 (d, *J* = 12.3 Hz, 2H); 3.32 (br.s, 2H) ppm. ^13^C-NMR (CDCl_3_) δ: 141.84 (C); 140.62 (C); 139.88 (CH); 129.87 (CH); 129.29 (CH); 102.04 (C); 65.75 (CH_2_) ppm. HRMS (ESI) calcd. for C_14_H_12_I_2_NaO_2_ [M + Na]^+^: 488.8824, found 488.8821.

*2,2′-Bis(bromomethyl)-3,3′-diiodo-1,1′-biphenyl* (**13**): To a mixture of HBr (100 mL, 30% in HOAc) and HOAc (100 mL) was added (3,3′-diiodo-[1,1′-biphenyl]-2,2′-diyl)dimethanol (3.593 g, 7.71 mmol) and the mixture was refluxed for 2 h. Upon cooling the first crop of product crystallized which was separated and dried under vacuum (3.776 g, 83%). To the clear filtrate was added ice water (300 mL) and DCM (200 mL). The aqueous phase was separated and extracted with DCM (2 × 50 mL). The combined organic phase was washed with water, sat. NaHCO_3_ solution and brine and dried (MgSO_4_). Evaporation gave a slightly less pure second crop of product (>90% NMR, 412 mg, 8%); total yield of **13**: 91%; m.p.: 173–175 °C. ^1^H-NMR (CDCl_3_) δ: 7.96 (dd, *J* = 8.0, 1.3 Hz, 2H); 7.26 (dd, *J* = 7.6, 1.2 Hz, 2H); 7.06 (t, *J* = 7.8 Hz, 2H); 4.43 (d, *J* = 10.2 Hz, 2H); 4.29 (d, *J* = 10.2 Hz, 2H) ppm. ^13^C-NMR (CDCl_3_) δ: 141.10 (C); 140.62 (CH); 137.51 (C); 130.08 (CH); 129.69 (CH); 102.02 (C); 37.64 (CH_2_) ppm. HRMS (EI) calcd. for C_14_H_10_^79^Br^81^BrI_2_: 591.7218, found: 591.7278.

*4,8-Diiodo-6,7-dihydro-5H-dibenzo[c,e]azepine* (**14**): A similar procedure as in the synthesis of **8** was applied with following modifications: The reaction was conducted at 50 °C for 48 h and the crude product was purified by MPLC in a solvent gradient (MeOH(0→10)/DCM) to yield **14** and minor amounts of *N-spiro* compound **14X**.

**14**: Yield: 73–77%; m.p.: 164–167 °C. ^1^H-NMR (CDCl_3_) δ: 7.91 (dd, *J* = 7.9, 1.2 Hz, 2H); 7.39 (dd, *J* = 7.6, 1.2 Hz, 2H); 7.09 (t, *J* = 7.8 Hz, 2H); 3.79 (br.m, 4H) ppm. ^13^C-NMR (CDCl_3_) δ: 142.33 (C); 139.51 (CH); 138.94 (C); 129.21 (CH); 127.93 (CH); 100.56 (C); 53.20 (CH_2_) ppm. HRMS (ESI) calcd. for C_14_H_12_I_2_N [M + H]^+^: 447.9054, found: 447.9050.

**14X**: Yield: 8–13%; m.p.: 295–298 °C. ^1^H-NMR (CDCl_3_) δ: 8.13 (dd, *J* = 8.0, 1.1 Hz, 4H); 7.70 (dd, *J* = 7.8, 1.1Hz, 4H); 7.47 (t, *J* = 7.9 Hz, 4H); 4.88 (d, *J* = 13.7 Hz, 4H); 4.59 (d, *J* = 13.7 Hz, 4H) ppm. ^13^C-NMR (CDCl_3_) δ: 142.75 (C); 141.31 (CH); 133.56 (CH); 130.27 (CH); 128.90 (C); 103.79 (C); 67.36 (CH_2_) ppm. HRMS (ESI) calcd for C_28_H_20_I_4_N [M − Br]^+^: 877.7769, found: 877.7763.

Suzuki-Miyaura coupling of **14** yielding **15a**–**15c**: *General Procedure D* was applied with exception of condition for the MPLC separations; a gradient EtOAc(20→50%)/heptane was used.

*4,8-Diphenyl-6,7-dihydro-5H-dibenzo[c,e]azepine* (**15a**): Yield: 78%; colorless foam. ^1^H-NMR (CDCl_3_) δ: 7.35–7.55 (m, 16H); 3.65 (br.m, 4H) ppm. ^13^C-NMR (CDCl_3_) δ: 142.55 (C); 142.02 (C); 141.28 (C); 134.24 (C); 129.74 (CH); 129.46 (CH); 128.17 (CH); 127.38 (CH); 127.18 (CH); 127.13 (CH); 45.20 (CH_2_) ppm. HRMS (ESI) calcd. for C_26_H_22_N [M + H]^+^: 348.1752, found 348.1740.

*4,8-Di(naphthalen-2-yl)-6,7-dihydro-5H-dibenzo[c,e]azepine* (**15b**): Yield: 76%; colorless foam. ^1^H-NMR (CDCl_3_) δ: 7.98 (br.s, 2H); 7.87–7.93 (m, 6H); 7.68 (dd, *J* = 8.1, 1.5 Hz, 2H); 7.59 (dd, *J* = 6.4, 2.6 Hz, 2H); 7.49–7.55 (m, 8H); 3.73 (br.m, 4H) ppm. ^13^C-NMR (CDCl_3_) δ: 142.61 (C); 141.94 (C); 138.83 (C); 134.45 (C); 133.29 (C); 132.47 (C); 130.02 (CH); 128.14 (CH); 128.10 (CH); 128.00 (CH); 127.68 (CH); 127.65 (CH); 127.52 (CH); 127.27 (CH); 126.27 (CH); 125.97 (CH); 45.32 (CH_2_) ppm. HRMS (ESI) calcd. for C_34_H_26_N [M + H]^+^: 448.2065, found 448.2057.

*4,8-Di([1,1′-biphenyl]-4-yl)-6,7-dihydro-5H-dibenzo[c,e]azepine* (**15c**): After extractive work-up large amount of product crystallized upon addition of diethylether of a concentrated solution in DCM; from the mother liquor more product was obtained by chromatography; total yield: 85%; m.p.: 250–255 °C. ^1^H-NMR (CDCl_3_) δ: 7.63–7.70 (m, 8H); 7.59–7.63 (m, 4H); 7.57 (dd, *J* = 7.6, 1.5 Hz, 2H); 7.51 (t, *J* = 7.4 Hz, 2H); 7.44–7.49 (m, 6H); 7.34–7.40 (m, 2H); 3.75 (br.m, 4H) ppm. ^13^C-NMR (CDCl_3_) δ: 142.62 (C); 141.63 (C); 140.79 (C); 140.25 (C); 140.04 (C); 134.28 (C); 129.92 (CH); 129.75 (CH); 128.81 (CH); 127.46 (CH); 127.35 (CH); 127.29 (CH); 127.10 (CH); 126.93 (CH); 45.29 (CH_2_) ppm. HRMS (ESI) calcd. for C_38_H_30_N [M + H]^+^: 500.2378, found: 500.2368. 

Synthesis of diiodo bisazepinium compounds **16**, **18**, and **19** (*Typical procedure*): To diiodoazepine (*S*)-**8** or **14** (0.5 mmol) and dibromide (*S*)-**10** or **11** (0.5 mmol), respectively in acetonitrile (5 mL) was added dry K_2_CO_3_ (414 mg, 3 mmol) and the suspension was degassed and stirred at 80 °C under argon overnight. After cooling to room temperature DCM (50 mL) and water (20 mL) was added. The aqueous phase was separated and extracted with DCM (3 × 10 mL). The combined organic layers were evaporated and the crude products purified by MPLC in MeOH (0→10%)/DCM.

*(S,S)-2,6-Diiodo-3,3′,5,5′-tetrahydro-4,4′-spirobi[dinaphtho[2,1-c:1′,2′-e]azepin]-4-ium bromide* (**16**): Yield: 65%; m.p.: 216–219 °C (dec.); ${\left[\alpha \right]}_{D}^{20}$ = +261 (c: 0.46, DCM). ^1^H-NMR (DMSO-*d*_6_) δ: 9.08 (s, 2H); 8.52 (d, *J* = 8.5 Hz, 2H); 8.31 (d, *J* = 8.6 Hz, 2H); 8.18 (d, *J* = 8.2 Hz, 2H); 8.15 (d, *J* = 8.3 Hz, 2H); 7.65 (ddd, *J* = 6.8, 2.9, 1.0 Hz, 2H); 7.63 (ddd, *J* = 6.8, 3.0, 1.2 Hz, 2H); 7.36 (m, 4H); 7.13 (br.d, *J* = 8.7 Hz, 2H); 6.80 (br.d, *J* = 8.6 Hz, 2H); 4.79 (d, *J* = 14.8 Hz, 2H); 4.43 (d, *J* = 14.2 Hz, 2H); 4.35 (d, *J* = 14.2 Hz, 2H); 4.33 (d, *J* = 14.8 Hz, 2H) ppm. ^13^C-NMR (DMSO-*d*_6_) δ: 141.06 (CH); 137.28 (C); 135.98 (C); 134.93 (C); 134.08 (C); 131.93 (CH); 130.99 (C); 130.93 (C); 128.86 (CH);128.44 (CH); 128.30 (CH); 127.76 (CH); 127.35 (C); 127.33 (CH); 127.29 (CH); 127.17 (CH); 126.80 (CH); 126.68 (CH); 125.69 (C); 97.09 (C); 64.94 (CH_2_); 62.29 (CH_2_) ppm. HRMS (ESI) calcd. for C_44_H_30_I_2_N [M − Br]^+^: 826.0462, found: 826.0480.

*(S)-2′,6′-Diiodo-3′,5,5′,7-tetrahydrospiro[dibenzo[c,e]azepine-6,4′-dinaphtho[2,1-c:1′,2′-e]azepin]-6-ium bromide* (**18**): Yield: 69%; m.p.: 255–259 °C (dec.); ${\left[\alpha \right]}_{D}^{20}$ = +74 (c: 0.81, DCM) ^1^H-NMR (CDCl_3_) δ: 8.69 (s, 2H); 8.04 (d, *J* = 8.0 Hz, 2H); 7.92 (d, *J* = 8.2 Hz, 2H); 7.68–7.73 (m, 4H); 7.62 (t, *J* = 7.5 Hz, 2H); 7.57 (m, 2H); 7.36 (t, *J* = 8.0 Hz, 2H); 7.24 (d, *J* = 8.7 Hz, 2H); 5.12 (d, *J* = 13.5 Hz, 2H); 5.02 (d, *J* = 13.5 Hz, 2H); 4.88 (d, *J* = 12.6 Hz, 2H); 4.38 (d, *J* = 12.6 Hz, 2H) ppm. ^13^C-NMR (CDCl_3_) δ: 141.33 (CH); 141.08 (C); 138.55 (C); 135.73 (C); 132.02 (CH); 131.61 (C); 131.55 (CH); 129.50 (CH); 128.69 (CH); 128.66 (CH); 128.01 (CH); 127.69 (CH); 127.60 (CH); 127.60 (C); 127.15 (C); 96.80 (C); 66.78 (CH_2_); 63.26 (CH_2_) ppm. HRMS (ESI) calcd. for C_36_H_26_I_2_N [M − Br]^+^: 726.0149, found: 726.0152.

*(S)-4,8-Diiodo-3′,5,5′,7-tetrahydrospiro[dibenzo[c,e]azepine-6,4′-dinaphtho[2,1-c:1′,2′-e]azepin]-6-ium bromide* (**19**): Yield: 93%; m.p.: 279–281 °C (dec.); ${\left[\alpha \right]}_{D}^{20}$ = +86 (c: 0.62, DCM). ^1^H-NMR (CDCl_3_) δ: 8.27 (d, *J* = 8.4 Hz, 2H); 8.15 (d, *J* = 8.4 Hz, 2H); 8.06 (dm, *J* = 8.4 Hz, 2H); 8.01 (dd, *J* = 8.1, 1.2 Hz, 2H); 7.63 (ddd, *J* = 8.1, 6.8, 1.1 Hz, 2H); 7.61 (dd, *J* = 7.7, 1.2 Hz, 2H); 7.54 (dm, *J* = 8.6 Hz, 2H); 7.41 (ddd, *J* = 8.5, 6.8, 1.3 Hz, 2H); 7.36 (t, *J* = 7.9 Hz, 2H); 5.26 (d, *J* = 12.7 Hz, 2H); 5.16 (d, *J* = 13.8 Hz, 2H); 4.70 (d, *J* = 13.6 Hz, 2H); 4.46 (d, *J* = 12.7 Hz, 2H) ppm. ^13^C-NMR (CDCl_3_) δ: 143.36 (C); 140.73 (CH); 136.92 (C); 134.51 (C); 132.78 (CH); 131.12 (C); 130.39 (CH); 130.24 (CH); 130.01 (C); 128.85 (CH); 127.78 (CH); 127.68 (CH); 127.53 (CH); 127.09 (CH); 126.94 (C); 102.69 (C); 66.43 (CH_2_); 64.32 (CH_2_) ppm. HRMS (ESI) calcd. for C_36_H_26_I_2_N [M − Br]^+^: 726.0149, found: 726.0138. 

*(R)-4-(((S)-2′-Benzyl-[1,1′-binaphthalen]-2-yl)methyl)-2,6-diphenyl-4,5-dihydro-3H-dinaphtho[2,1-c:1′,2′-e]azepine* (**17a**): Yield: 20–40%. ^1^H-NMR (CDCl_3_) δ: 8.03 (d, *J* = 8.0 Hz, 1H); 7.96 (d, *J* = 8.0 Hz, 2H); 7.89 (d, *J* = 8.5 Hz, 1H); 7.80 (d, *J* = 8.3 Hz, 1H); 7.77 (s, 2H); 5.58 (s, 2H); 7.54 (m, 3H); 7.50 (d, *J* = 8.7 Hz, 2H); 7.33 (m, 3H); 7.23 (m, 2H); 6.97–7.18 (m, 10H); 7.95 (d, *J* = 8.5 Hz, 1H); 6.86 (d, *J* = 8.7 Hz, 1H); 6.81 (t, *J* = 7.3 Hz, 1H); 6.73 (d, *J* = 8.5 Hz, 1H); 6.63 (t, *J* = 7.6 Hz, 2H); 6.29 (d, *J* = 7.6 Hz, 2H); 3.77 (d, *J* = 12.8 Hz, 2H); 3.16 (d, *J* = 16.0 Hz, 1H); 2.99 (d, *J* = 14.9 Hz, 1H); 2.84–2.94 (br.m, 4H) ppm. ^13^C-NMR (CDCl_3_) δ: 140.90 (C); 140.45 (C); 139.65 (C); 136.22 (C); 136.04 (C); 135.00 (C); 134.12 (C); 133.85 (C); 133.22 (C); 132.52 (C); 132.43 (C); 132.42 (C); 132.32 (C); 132.06 (C); 130.71 (C); 129.70 (CH); 129.10 (CH); 128.55 (CH); 128.29 (CH); 127.99 (CH); 127.91 (CH); 127.84 (CH); 127.79 (CH); 127.59 (CH); 127.53 (CH); 127.15 (CH); 126.73 (CH); 126.26 (CH); 126.21 (CH); 125.79 (CH); 125.76 (CH); 125.74 (CH); 125.65 (CH); 125.61 (CH); 125.57 (CH); 125.37 (CH); 125.14 (CH); 57.49 (CH_2_); 51.72 (CH_2_); 39.31 (CH_2_) ppm. HRMS (ESI) calcd. for C_62_H_46_N [M + H]^+^: 804.3625, found: 804.3640.

*(R)-2,6-Di(naphthalen-2-yl)-4-(((S)-2′-(naphthalen-2-ylmethyl)-[1,1′-binaphthalen]-2-yl)methyl)-4,5-dihydro-3H-dinaphtho[2,1-c:1′,2′-e]azepine* (**17b**): Yield: 20–30%. ^1^H-NMR (CDCl_3_) δ: 8.04 (d, *J* = 8.0 Hz, 1H); 8.01 (d, *J* = 8.2 Hz, 2H); 7.89 (s, 2H); 7.81 (d, *J* = 8.6 Hz, 1H); 7.79 (br.d, *J* = 7.8 Hz, 2H); 7.72 (br.s, 2H); 7.69 (br.d, *J* = 8.0 Hz, 2H); 7.45–7.61 (m, ~11H); 7.28–7.42 (m, 8H); 7.22 (ddd, *J* = 8.0, 6.8, 1.1 Hz, 1H); 7.08 (ddd, *J* = 8.3, 6.8, 1.2 Hz, 1H); ~7.0 (br.m, 1H); 7.00 (ddd, *J* = 8.3, 6.8, 1.3 Hz, 1H); 6.94 (d, *J* = 8.6 Hz, 1H); 6.90 (br.d, *J* = 8.4 Hz, 1H); 6.83 (s, 1H); 6.79 (d, *J* = 8.4 Hz, 1H); ~6.7 (br.m, 1H); 6.69 (d, *J* = 8.6 Hz, 1H); 6.36 (dd, *J* = 8.5, 1.6 Hz, 1H); 3.84 (d, *J* = 12.9 Hz, 2H); 3.24 (d, *J* = 15.5 Hz, 1H); ~3.1 (br.m, 2H); 3.01 (d, *J* = 15.6 Hz, 1H); 3.00 (d, *J* = 14.6 Hz, 1H); 2.97 (d, *J* = 14.8 Hz, 1H) ppm. ^13^C-NMR (CDCl_3_) δ: 140.39 (C); 138.47 (C); 137.39 (C); 136.29 (C); 136.15 (C); 135.07 (C); 134.25 (C); 133.79 (C); 133.31 (C); 133.13 (C); 132.94 (C); 132.54 (C); 132.53 (C); 132.32 (C); 132.31 (C); 131.71 (C); 130.88 (C); 128.87 (CH); 128.42 (CH); 128.39 (CH); 128.11 (CH); 128.07 (CH); 128.01 (CH); 127.77 (CH); 127.71 (CH); 127.68 (CH); 127.62 (CH); 127.60 (CH); 127.52 (CH); 127.45 (CH); 127.37 (CH); 127.27 (CH); 127.24 (CH); 126.46 (CH); 126.21 (CH); 126.10 (CH); 125.88 (CH); 125.82 (CH); 125.80 (CH); 125.77 (CH); 125.60 (CH); 125.56 (CH); 125.43 (CH); 125.00 (CH); 124.93 (CH); 57.67 (CH_2_); 51.90 (CH_2_); 39.59 (CH_2_) ppm (2C, 1CH not observed). HRMS (ESI) calcd. for C_74_H_52_N [M − Br]^+^: 954.4094, found: 954.4095.

### 3.3. X-ray Analysis

The X-ray intensity data were measured on Bruker D8 Venture diffractometer (Bruker, Billerica, MA, USA) equipped with multilayer monochromator, Mo K/a and Cu K/a INCOATEC micro focus sealed tubes and Kryoflex II or Oxford 800 cooling devices. The structures were solved by direct methods and refined by full-matrix least-squares techniques. Non-hydrogen atoms were refined with anisotropic displacement parameters. Hydrogen atoms were inserted at calculated positions and refined with a riding model. The following software was used: Bruker SAINT software package [34] sing a narrow-frame algorithm for frame integration, SADABS [35] for absorption correction, OLEX2 (version 1.2.9, OlexSys Ltd, Durham, UK) [36] for structure solution, refinement, molecular diagrams and graphical user-interface, Shelxle (Rev 833, University of Goettingen, Goettingen, Germany) [37] for refinement and graphical user-interface SHELXS-2013 [38] for structure solution, SHELXL-2013 [39] for refinement, Platon (version 270106, Utrecht University, Utrecht, Netherlands) [40] for symmetry check. Crystal data for structures of **3a**, **8**, **16**, and **17b** are collected in Table 1. Experimental data and CCDC-Codes can be found in the Supplementary Materials.

## Figures and Tables

**Table 1:** Crystal data for **3a**, **8**, **16**, and **17b**.

	3a	8	16	17b
M [g/mol]	945.42	866.80	1161.18	1208.93
Space group	*C2/c*	*P2_1_*	*P2_1_2_1_2_1_*	*P-1*
a [Å]	32.9747(11)	11.8178(7)	8.9851(5)	12.3238(10)
b [Å]	33.7652(11)	17.2708(10)	11.0959(7)	13.5521(11)
c [Å]	19.2774(7)	14.3841(8)	44.154(3)	19.1746(19)
α [°]	90	90	90	71.502(2)
β [°]	119.5218(14)	94.2551(19)	90	86.172(4)
γ [°]	90	90	90	84.204(3)
V [Å^3^]	18676.7(11)	2927.7(3)	4402.1(5)	3019.4(5)
Z	16	4	4	2
Dcalc [g/cm^3^]	1.345	1.967	1.752	1.330
R_int_	0.1057	0.0567	0.0471	0.0295
R_sigma_	0.0620	0.0214	0.0263	0.0361
R1 (I > 2σ(I))	0.0529	0.0215	0.0318	0.0707
wR2 (all data)	0.1394	0.0496	0.0736	0.1912

## Supplementary Materials

The following are available on http://www.mdpi.com/1420-3049/23/4/750/s1, containing ^1^H- and ^13^C-NMR charts, details of crystal structure determinations and preparation of precursor **6**.
