# Supplementary material for: Economy of Catalyst Synthesis—Convenient Access to Libraries of Di- and Tetranaphtho Azepinium Compounds
Source: Molecules. 2018 Mar 24;23(4):750. doi: 10.3390/molecules23040750 (PMC6017052; doi:10.3390/molecules23040750)
Supplement: Supplementary file 1 [file molecules-23-00750-s001.pdf]

Supplementary Material

Economy of Catalyst Synthesis—Convenient Access to Libraries of Di- and Tetranaphtho Azepinium Compounds

Sorachat Tharamak [1], Christian Knittl-Frank [2], Auraya Manaprasertsak [1], Anchulee Pengsook [1], Lydia Suchy [2], Philipp Schuller [2], Barbara Happl [3], Alexander Roller [3] and Michael Widhalm [4,*]

[1] Department of Chemistry, Faculty of Science, Kasetsart University, Bangkok 10900, Thailand; sorachat.th@ku.th (S.T.); auraya.ma@ku.th (A.M.); anchulee.peng@ku.th (A.P.)
[2] Institute of Organic Chemistry, University of Vienna, Währinger Straße 38, 1090 Wien, Austria; christian.knittl-frank@univie.ac.at (C. K.-F.); a01205005@unet.univie.ac.at (L.S.); philipp.daniel.schuller@gmail.com (P.S.)
[3] Institute of Inorganic Chemistry, University of Vienna, Währinger Straße 42, 1090 Wien, Austria; barbara.happl@univie.ac.at (B.H.); alexander.roller@univie.ac.at (A.R.)
[4] Institute of Chemical Catalysis, University of Vienna, Währinger Straße 38, 1090 Wien, Austria
* Correspondence: m.widhalm@univie.ac.at; Tel.: +43-01-4277-70305

Contents

Large Scale Synthesis of Non-Racemic 1,1′-Binaphthyl-2,2′-dicarboxylic acid **6** and Sequence Products **7** and **8**. (Comparison of methods from the literature) S2

^{1}H-NMR and ^{13}C-NMR Spectra S8

Crystal Structure Analysis of **3a**, **8**, **16**, and **17b** S91

Large Scale Synthesis of Non-Racemic 1,1′-Binaphthyl-2,2′-dicarboxylic acid 6 and Sequence Products 7 and 8. (Comparison of methods reported in the literature)

The discussion of preparative scale synthesis yielding enantiopure dihydroazepine **8** via diacid **6** from commercially available starting material will focus on practical aspects (Scheme S1 and Table S1).
The synthesis of enantiomers of **8** requires twelve steps when starting from 2-methylnaphthalene (**20**) and might include one optical resolution procedure (Scheme S1). With this sequence a total yield of 30% can be expected based on substrate quantities reported in the literature (Table S1). In early steps the reactions were run on typically 40-400 mmol scale with the exception of step *d* where a 12.7 mmol scale was reported. For the late steps *h-k* up to 15 mmol of substrate could be reacted with usual laboratory equipment without problems.

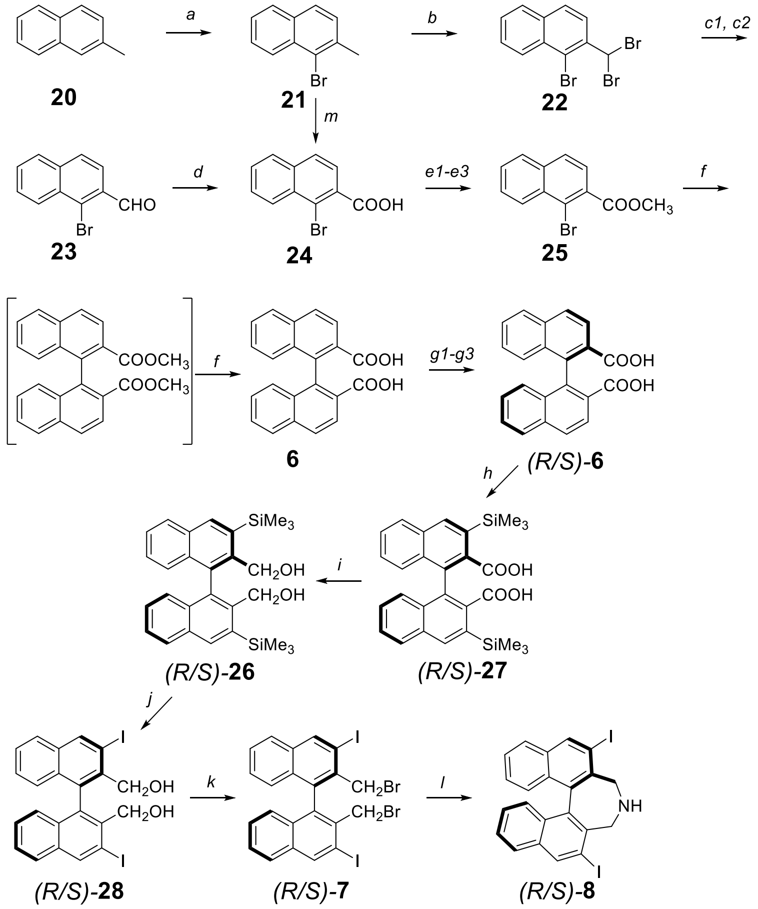

Scheme S1: Synthesis of non-racemic **7** and **8** via **6** from **20** (*Route A*)

Enantiomers: Non-racemic material was commonly obtained by classical optical resolution of diastereomeric compounds / salts at the stage of the diacid **6**. Three practicable methods *g1*-*g3* should be considered. The use of brucine as a resolving agent (*g1*) is hampered due to high price and toxicity.[1] Method *g2* uses the less expensive non-racemic 1-phenylethylamine but requires additional steps to

cleave diastereomeric amides.[2] In method *g3*, finally, the preferred formed 1:2 salt crystallizes and was separated from the mother liquor. The resolving agent was recovered in good yield but following this protocol only one enantiomer of **6** was obtained.[3] The yields of all methods are typically in a range of 40% for each enantiomer.

An interesting report was published which significantly shortens the synthesis. The oxidation of 1-bromo-2-methylnaphthalene (**21**) with O_2 catalysed by $Co(OAc)_2$ giving **24** (step *m*) is conducted in a steel autoclave[3] and substitutes three steps *b-d*. The apparently easy operation without purification and good yield (87%) on a large scale (482 mmol) makes this protocol very attractive saving time and man power (same overall yield as *b-d* within 1%). Merely, the requirement of a 1L-autoclave which might be not generally available is unfavourable.

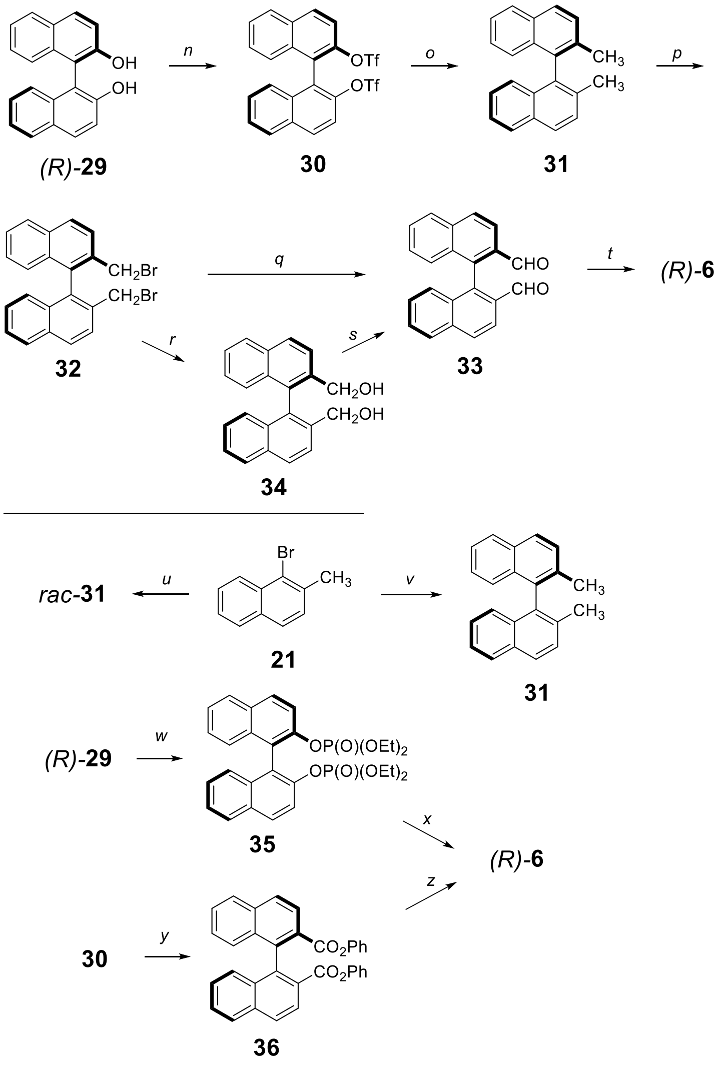

Scheme S2: Synthesis of **6** from **29** (*Route B*)

An alternative route to non-racemic **6** starts from (*R*)- or (*S*)-2,2′-dihydroxy-1,1′-binaphthyl (**29**) which can be obtained by optical resolution using fractional crystallisation of *N*-benzylcinchonidinium

clathrate complexes[4,5,6] on a 100 g scale but is also commercially available at a reasonable price. For the preparation of 2,2′-dimethyl-1,1′-binaphthyl (**31**) a Kumada coupling of bistriflate **30** with MeMgCl, MeMgBr or MeMgI and Ni(dppp)Cl_2 as catalysts worked well.[29] The reaction proceeded on a 10-23 mmol scale without racemisation and was frequently reported.[7,8 ,9,10,11]Both, **30** and **31** were isolated in pure form after simple filtration over silica in >99% and 95-99% yield, respectively.[29]

Dimethylbinaphthyl **31** was also obtained from **21** as a racemate or enantioselectively using chiral catalysts. The Kumada type biaryl coupling was performed with aryl-Grignard reagents and aryl bromides catalysed by Ni complexes to yield racemic **31** (61%, 12.6 mmol scale, 1 mol% Ni(PPh_3)$_2$$Cl_2$)[12] or enantioenriched **31** (69%, 13 mmol scale, 1 mol% (*S*)(*R*)-PPFOMe, 95%e.e. (*R*).configuration).[13] Other protocols (including Suzuki-Miyaura coupling) requiring expensive chiral catalysts and/or starting materials, seem less appropriate for multigram preparation.[14,15,16,17]

For the stepwise oxidation of **31** to **6** NBS bromination was applied followed by hydrolysis/oxidation either *via* diol **34** or directly from **32** to dialdehyde **33** which was finally treated with $KMnO_4$ in acetone/water[34] or H_2O_2, $NaClO_2$, NaH_2PO_4 in MeCN/water[33] to afford **6**. Yields are good to fair (*n-o-p-q-s-t*: 63% overall yield or *v-p-r-s-t*: 47% overall yield). When comparing *Route A* with *Route B* the latter one will be preferable if non-racemic **6** is desired and enantiopure binaphthol **29** is available. Disadvantageous is the need of expensive triflic anhydride in step *n*.

Finally, a two step sequences from **29** to **6** might be considered as well. In an early report the bistriflate **30** was methoxycarbonylated under Pd(II)/dppp catalysis to afford the dimethylester of **6** in 83% yield.[18] The use of CO and requirement of noble metal catalysis obviously hampered upscaling and broad use of this protocol. Two other processes working on gram scale were recently reported. After transformation of **29** to 2,2′-diethylphosphate **35** (quant. yield) this was treated with Li-naphthalenide at -78 °C to give the di-lithio compound which reacted with CO_2 to afford **6** in up to 89% yield (3.6 mmol scale). The need of a column chromatography to purify **6** makes up-scaling more difficult (5 mmol scale reported).[19] In an other report triflate **30** was converted to diphenylester **36** using phenylformiate as CO source and Pd(OAc)$_2$/DPPP as catalyst which was followed by hydrolysis to afford **6**.[20] It is worth noting that both processes can be performed stereoconservative, *i.e.* without racemisation.

Summarizing, for multigram synthesis of 1,1′-binaphthyl-2,2′-dicarboxylic acid (**6**) two comparable routes are available, starting from either 2-methylnaphthalene (**20**) (*Route A*, Scheme S1) or 2,2′-dihydroxy-1,1′-binaphthyl (**29**) (*route B*, Scheme S2). Preference will be given depending on the need of racemic or non-racemic material. In the first case *Route A* is more convenient and requires 4 steps (if reaction *m* can be performed) or 6 steps with an overall yield of 66-67%. If at this stage an optical resolution is performed the yield will drop to 25-26% for each enantiomer of **6**. In this case *Route B* is superior yielding 63% of non-racemic **6**. In contrast, the asymmetric biaryl coupling (Scheme S2, *v*) requiring expensive catalysts and long reaction time is less appropriate particularly for large scale

preparations. An evaluation of both routes based on time and manpower requirement is rather difficult as the reported time for each step in Table S1 is a rough estimate on the published procedures and do not include preparation/drying/evaporation of solvents. Nevertheless, for the preparation of 5-10 g of **6** an approximate time frame with 10-12 days for *Route A* and 7-9 days more for optical resolution (*g*2), and 10-11 days for *route B* will be a valid approximation.

Comments on Table S1

a: While the bromination of 2-methylnaphthalene (**20**) with Br_2 in CS_2 yields up to 91% of **21** after distillation, we found the use of HBr/H_2O_2 a more convenient method which could be upscaled to 0.5 mol yielding 95% of the desired product without purification (> 98%, NMR) and sufficiently pure for the next step.

b: Treatment with excess NBS / AIBN in benzene or CCl_4 gave the tribromide **22** in excellent yield.

c: The conversion to aldehyde **23** proceeds smoothly and should also work on multigram scale.

d: Although $KMnO_4$ oxidation performs satisfying the absence of heavy metal residues with the system $NaClO_2/KH_2PO_4$ makes it more appropriate.

e: For esterification of **24** several protocols can be applied. Due to price and toxicity of MeI *e2* is limited to small scale preparations. The cheapest one is obviously the combination $SOCl_2$/MeOH. No chromatography is needed.

f: Many binaphthyl coupling methods are known but from the practical point of view the classical Ullmann coupling in DMF is still attractive due to simplicity of the procedure, easy work-up and good yields. Copper powder was activated by treatment with EDTA solution.[21] The crude dimethyl 1,1′-binaphthalene-2,2′-dicarboxylate was immediately hydrolysed and after extractive purification is sufficiently pure.

g: At this stage an optical resolution may be performed.

h-k: These steps were already published for enantiomerically pure substrates and largely omit chromatographic purification. Only for step *k* the mother liquor from the crystallisation was chromatographed. Repetition with racemic substrate gave comparable yields (±2%).

i: Reaction with aqueous ammonia yielded exclusively the secondary amine **8**, provided the reaction temperature was kept at 60 °C. No tertiary amine or *spiro*-ammonium compound was detected.

Table S1. Synthesis of Diiodoazepine **8** from 2-Methylnaphthalene **22** (Overview)

Step	Reagent/conditions	Scale (Mmol)	Purification	Time	Yield	Notes
a[22]	HBr/H_2O_2	1	no	2 d	95%	a
b1[23]	NBS/ABIN	42.5	chrom.	2 d	97%	
b2[24]	NBS/ABIN	10	chrom.	2 d	95%	
c1[23]	$CaCO_3$/water	41	cryst.	1 d	95%	
c2[24]	AgOAc/acetone-water	10	chrom.	2 d	95%	
d[25]	$NaClO_2$/KH_2PO_4	12.7	no	1 d	94%	
e1[26]	H_2SO_4/MeOH	1	no	1 d	72%	
e2[27]	K_2CO_3/MeI	100	chrom.	1 d	95%	
e3[3]	$SOCl_2$/MeOH	419	no	8 h	96%	
f[3]	1. Cu/DMF, 2. KOH/ MeOH	~401	extract.	3 d	84%	b
g1[1]	Brucine	88	cryst.	4-5 d	40(*R*)/45(*S*)%	c
g2[2]	1. (*S*)-1-phenylethyl-amine, DCC/THF, MeCN<br>2. $SOCl_2$, MeOH, KOH	43.8	cryst.	~7 d	39(*S*)/38(*R*)%	c, d
g3[3]	(*R*)-CHEA,Me_2NH/MeOH	30	cryst.	2 d	38(*R*)%	c, e
h[28]	*n*-BuLi, TMP, Me_3SiCl, THF	15	precip.	2 d	84%	f, g
i[28]	BH_3/THF	15	no	2 d	84%	f, g
j[28]	ICl/DCM	15	no	1 d	90%	f, g
k[28]	PBr_3/DCM, THF	15	cryst.	2 d	78%	f, g, h
	HBr/HOAc	5	no	4 h	96%	this paper
l	NH_3/CH_3CN		precip.	2 d	80-90%	this paper [i]
m[3]	O_2, $Co(OAc)_2$/butanone, HOAc	482	no	1 d	87%	
n[29]	Tf_2O, 2,6-dimethylpyridine/DCM	24	chrom., cryst.	1 d	99%	
o[29]	MeMgBr, DPPP/$NiCl_2$/cyclohexane	23.6	chrom., cryst.	~3 d	90-92%	
p[29]	NBS, AIBN, hν/cyclohexane	14.2	chrom., cryst.	1 d	88%	
q[30]	1. $NaHCO_3$/DMSO, 2. PDC/DCM	2.5	chrom.	2 d	70%	
r[31]	1. KOAc, Bu_4NBr/DMF, 2. KOH/dioxane-H_2O	8	cryst.	4 d	88%	
s[32]	MnO_2/toluene	1.6	no	1 d	99%	
t[33]	H_2O_2, $NaClO_2$, NaH_2PO_4/H_2O, MeCN	30	no	2 h	91%	

u[12]	$Ni(PPh_3)_2Cl_2$, **21**-Mg/benzene/Et_2O	12.6	distil.	2 d	61%	[k]
v[13]	$NiBr_2$, (*S*)(*R*)-PPFOMe, **21**-Mg/toluene/Et_2O	10	chrom.	5 d	68%	95%e.e.[l]
w[19]	$ClP(O)(OEt)_2$, NaH/THF	3.5	chrom.	4 h	quant.	[m]
x[19]	Li-naphthalene/THF then CO_2	3.5	chrom.	6 h	89%	[m]
y[20]	phenyl formiate, $Pd(OAc)_2$/DPPP, $^{i}Pr_2EtN$/neat	4.0	chrom.	3 d	63%	[m]
z[20]	KOH/MeOH, water	0.4[n]	chrom.	2 d	89%	[m]

Legend: [a] Pure by NMR (>98%). [b] Two steps, no purification of intermediate. [c] Optical resolution. [d] Two steps. [e] Only one enantiomer isolated. [f] Yields reported for enantiomerically pure material. [g] Synthesis was conducted on a 15 mmol scale with unchanged yield.[34] [h] Mother liquor was chromatographed. [i] Alternatively purified by crystallisation. [j] After two crystallisations. [k] Excess of Grignard reagent of **21** used. [l] Excess of **21** used; 99%e.e. after one cryst. [m] Reported for enantiopure starting material, no racemisation was observed. [n] In the paper the hydrolysis step is reported only on a 0.4 mmol scale but might be upscaled without problems.

¹H- and ¹³C-NMR spectra (If not otherwise noted spectra are recorded at room temperature in $CDCl_3$)

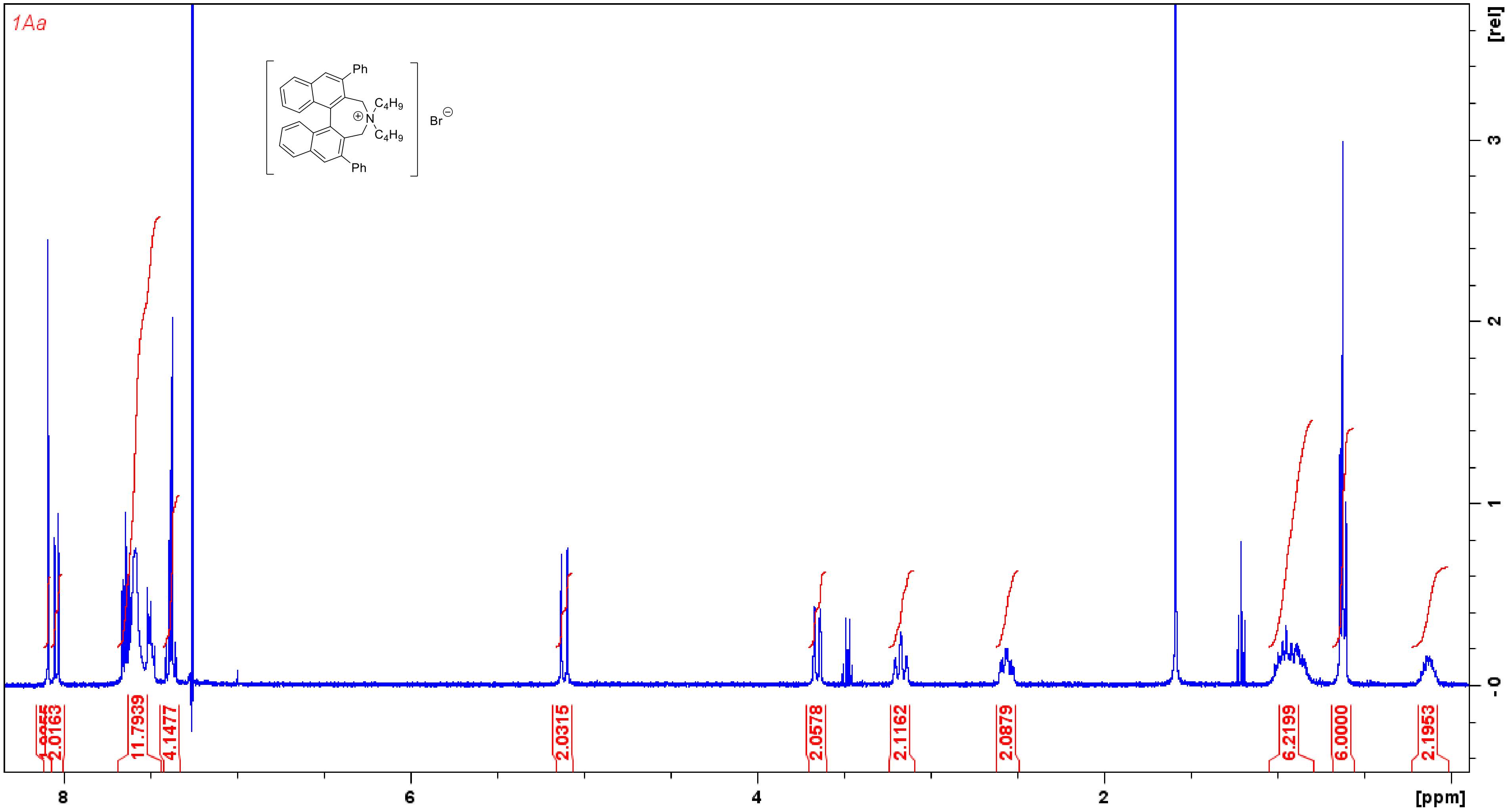

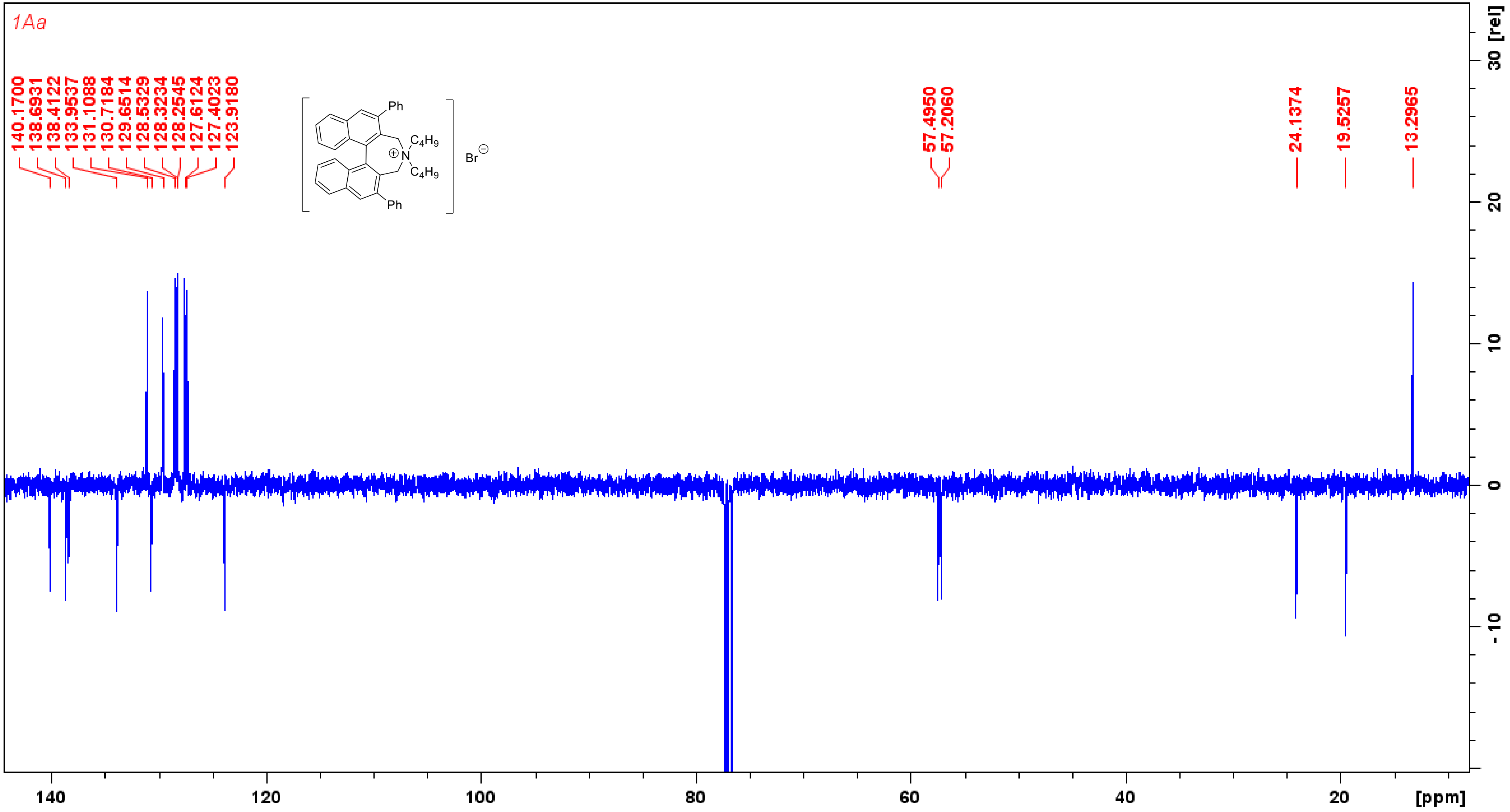
1Aa
140.1700
138.6931
138.4122
133.9537
131.1088
130.7184
129.6514
128.5329
128.3234
128.2545
127.6124
127.4023
123.9180
Ph
C_4H_9
N
C_4H_9
Ph
Br
57.4950
57.2060
24.1374
19.5257
13.2965
30 [rel]
20
10
0
-10
140
120
100
80
60
40
20
[ppm]

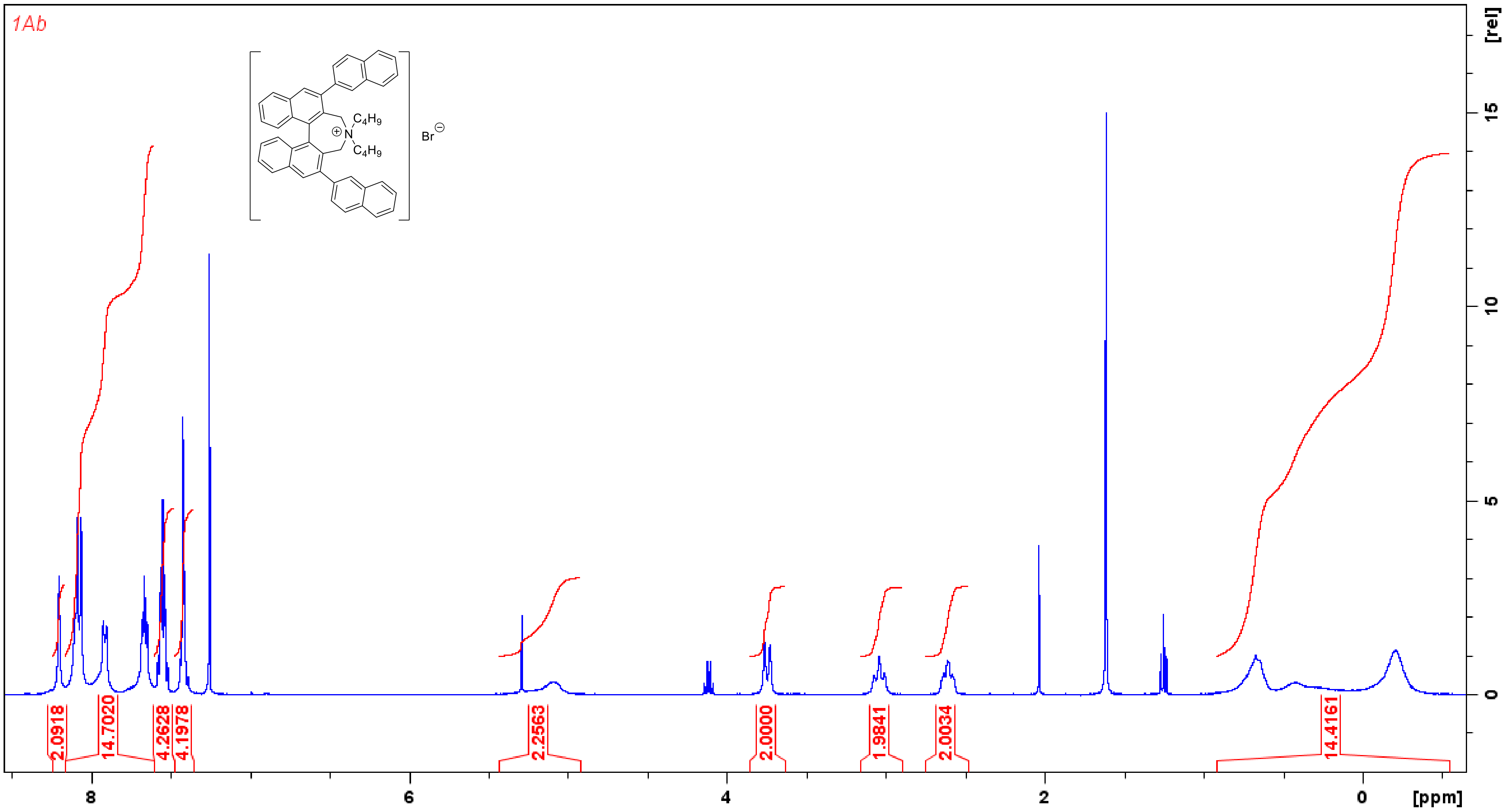
1Ab
C4H9
C4H9
N
Br
2.0918
14.7020
4.2628
4.1978
2.2563
2.0000
1.9841
2.0034
14.4161
8
6
4
2
0
[ppm]
15
10
5
0
[rel]

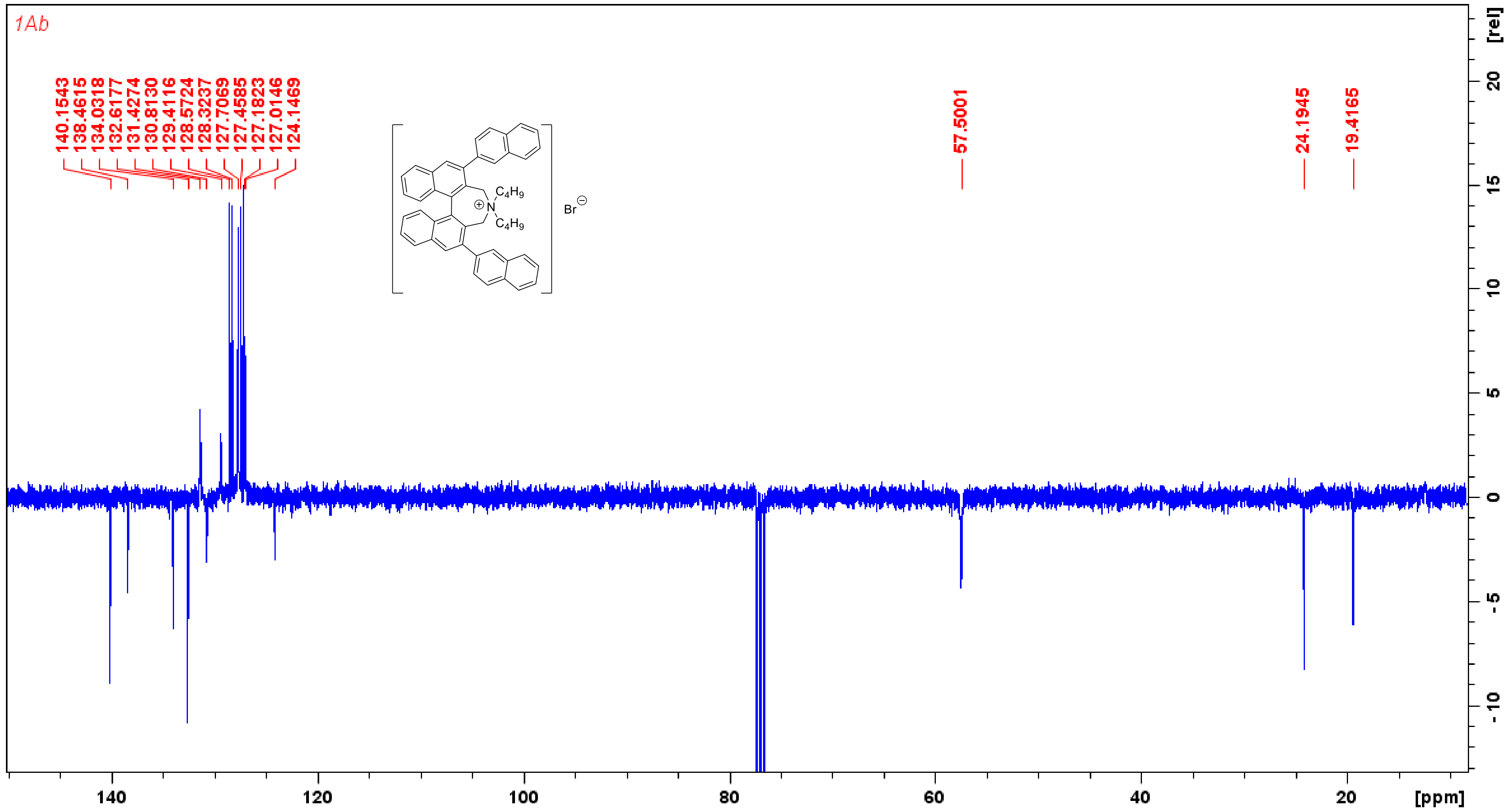
1Ab
140.1543
138.4615
134.0318
132.6177
131.4274
130.8130
129.4116
128.5724
128.3237
127.7069
127.4585
127.1823
127.0146
124.1469
57.5001
24.1945
19.4165
Br
[rel]
[ppm]

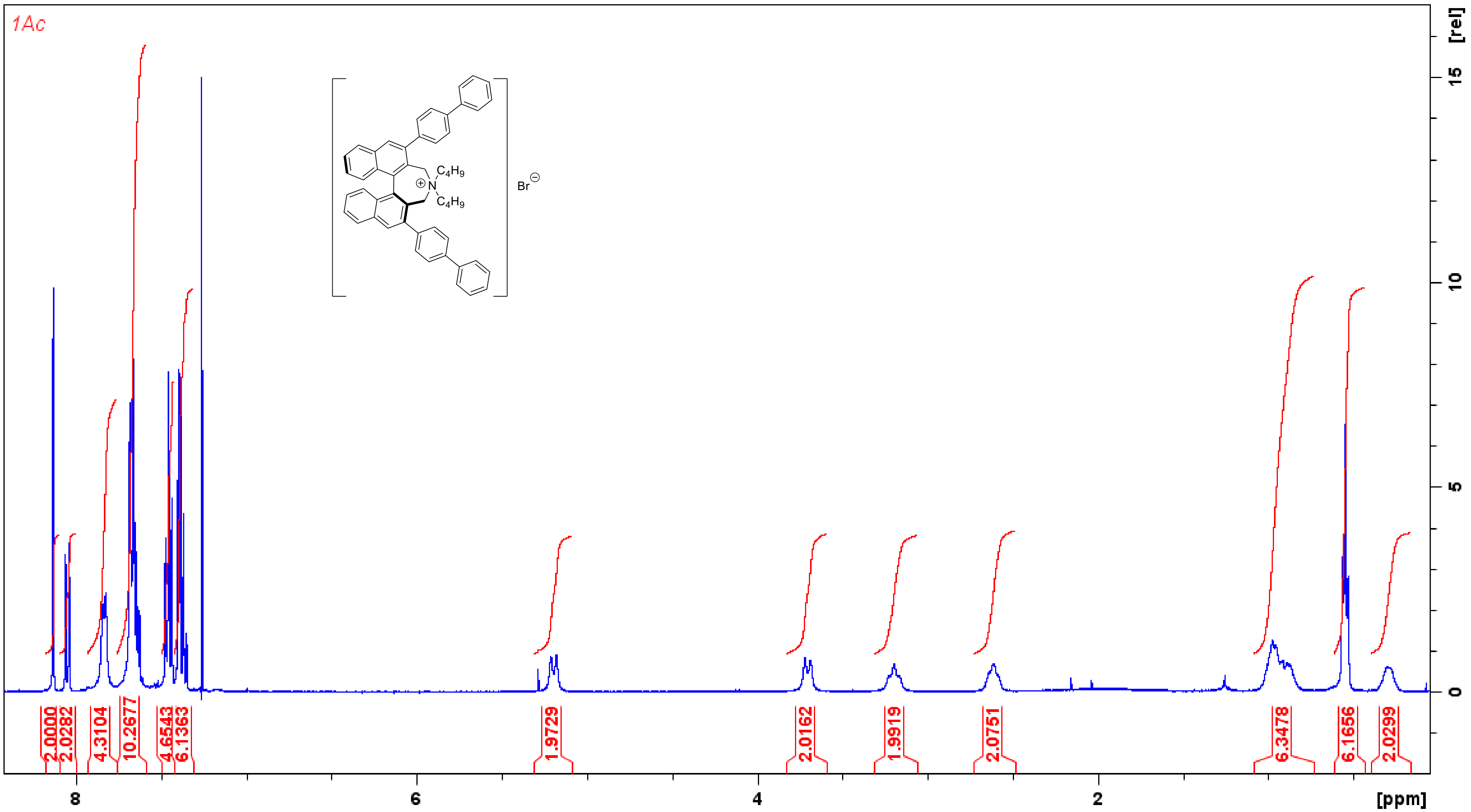
1Ac
C_4H_9
C_4H_9
N
$Br^{\ominus}$
2.0000
2.0282
4.3104
10.2677
4.6543
6.1363
1.9729
2.0162
1.9919
2.0751
6.3478
6.1656
2.0299
8
6
4
2
[ppm]
[rel]
15
10
5
0

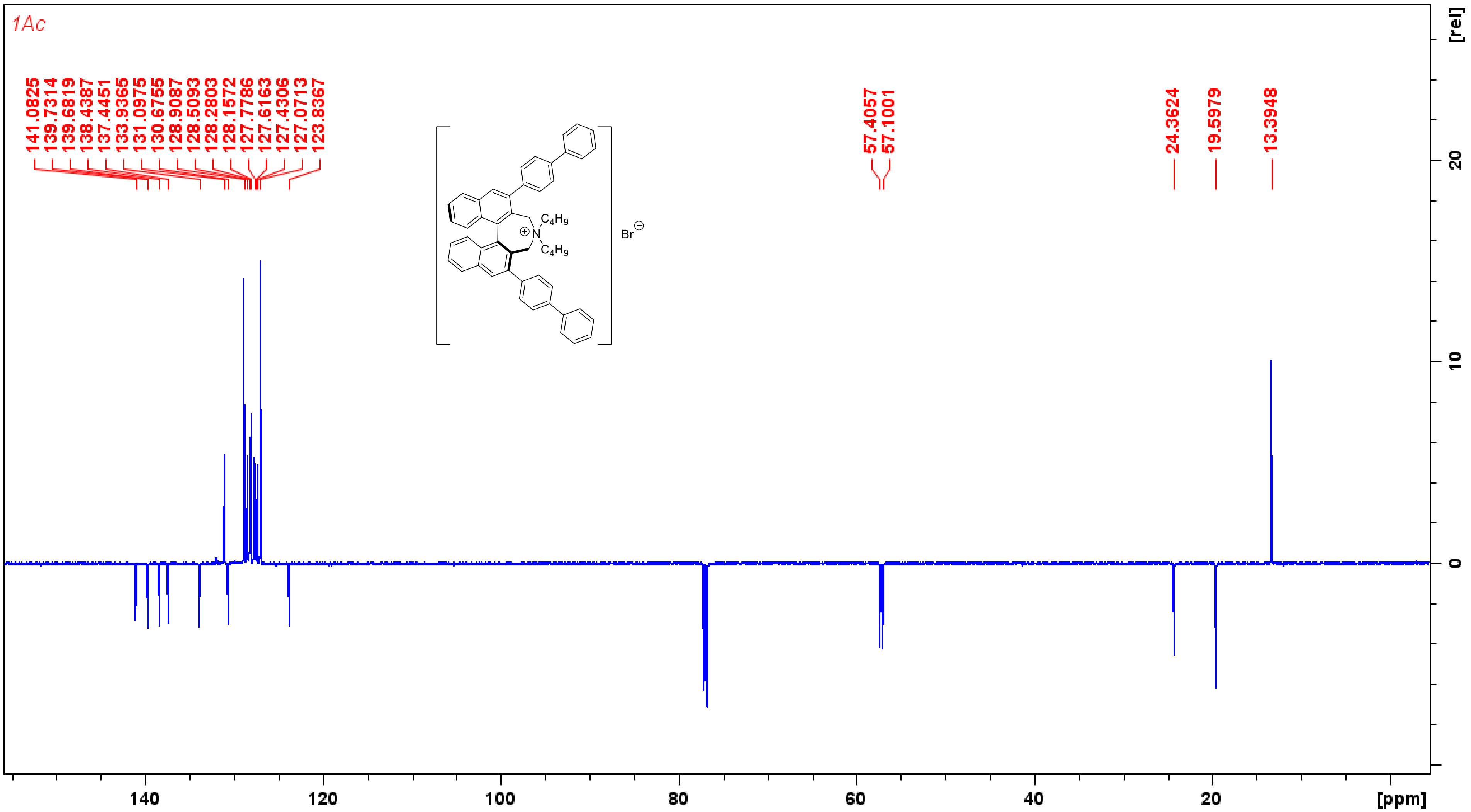

1Ac
141.0825
139.7314
139.6819
138.4387
137.4451
133.9365
131.0975
130.6755
128.9087
128.5093
128.2803
128.1572
127.7786
127.6163
127.4306
127.0713
123.8367
57.4057
57.1001
24.3624
19.5979
13.3948
C4H9
N
C4H9
Br
[rel]
20
10
0
140
120
100
80
60
40
20
[ppm]

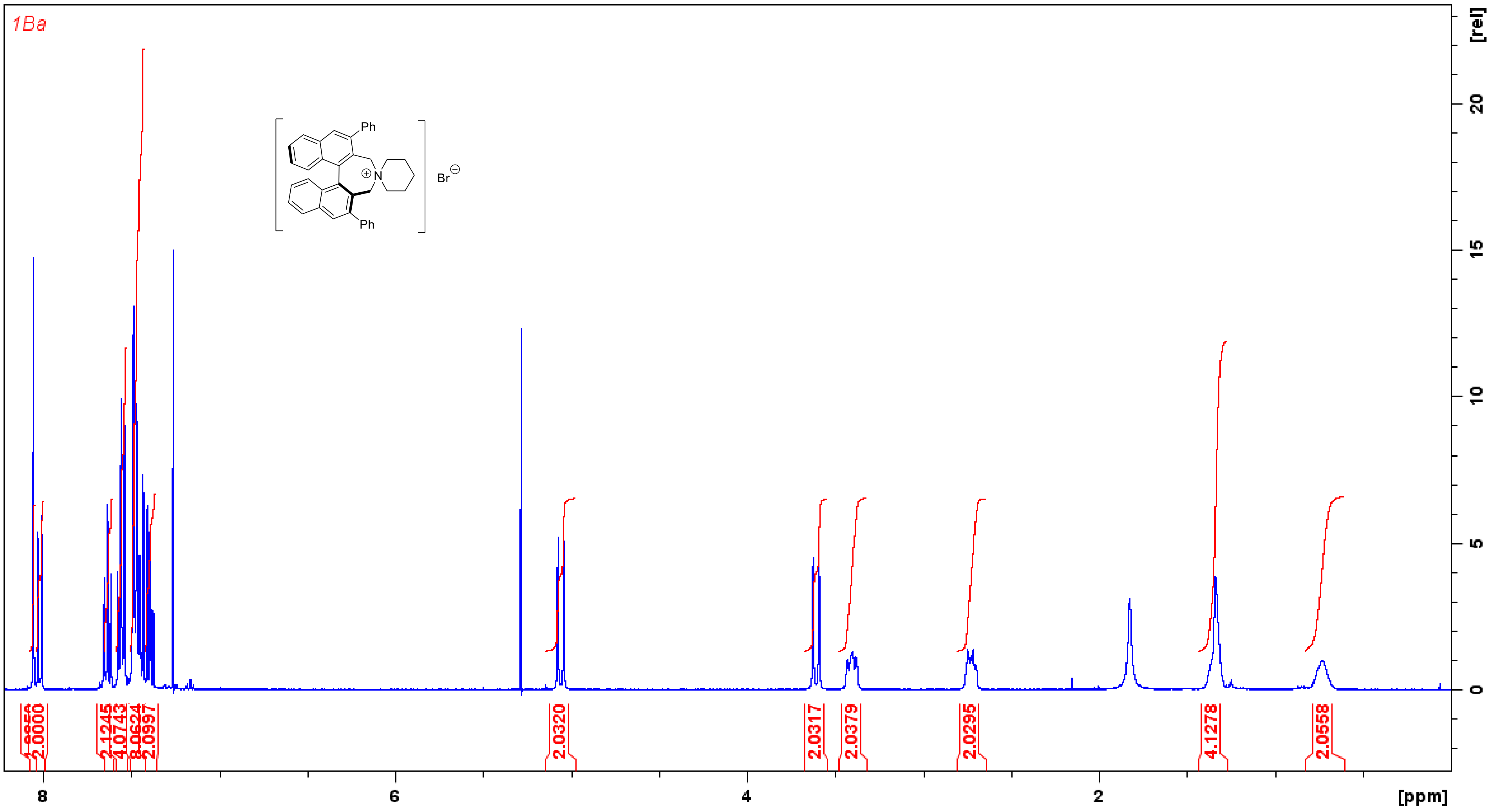
1Ba
Ph
N
Br
Ph
2.0000
2.1245
4.0743
8.0624
2.0997
2.0320
2.0317
2.0379
2.0295
4.1278
2.0558
8
6
4
2
[ppm]
20
15
10
5
0
[rel]

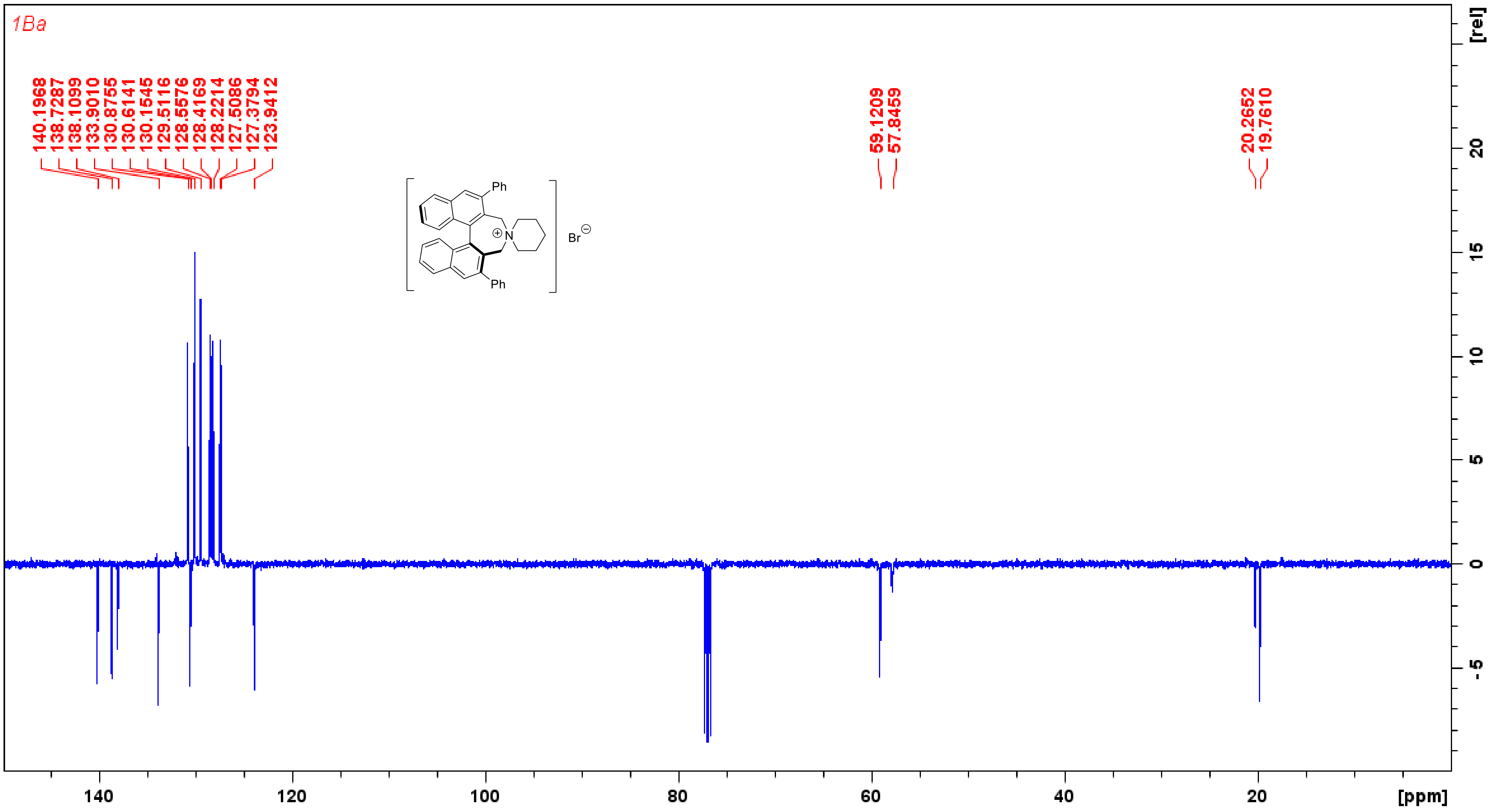
1Ba
140.1968
138.7287
138.1099
133.9010
130.8755
130.6141
130.1545
129.5116
128.5576
128.4169
128.2214
127.5086
127.3794
123.9412
59.1209
57.8459
20.2652
19.7610
Ph
N
Ph
Br
[rel]
20
15
10
5
0
-5
140
120
100
80
60
40
20
[ppm]

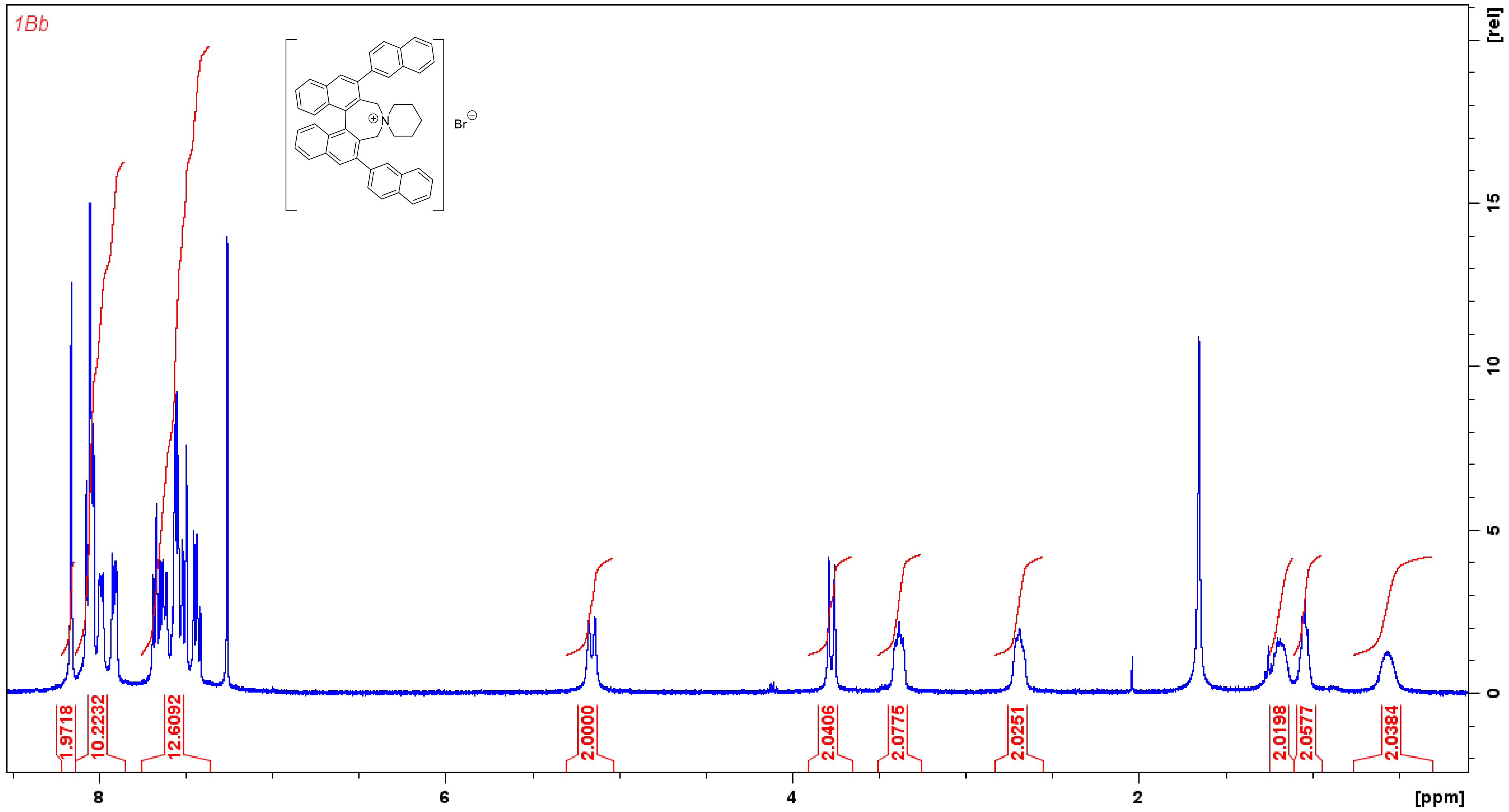
1Bb
Br
1.9718
10.2232
12.6092
2.0000
2.0406
2.0775
2.0251
2.0198
2.0577
2.0384
8
6
4
2
[ppm]
[rel]
15
10
5
0

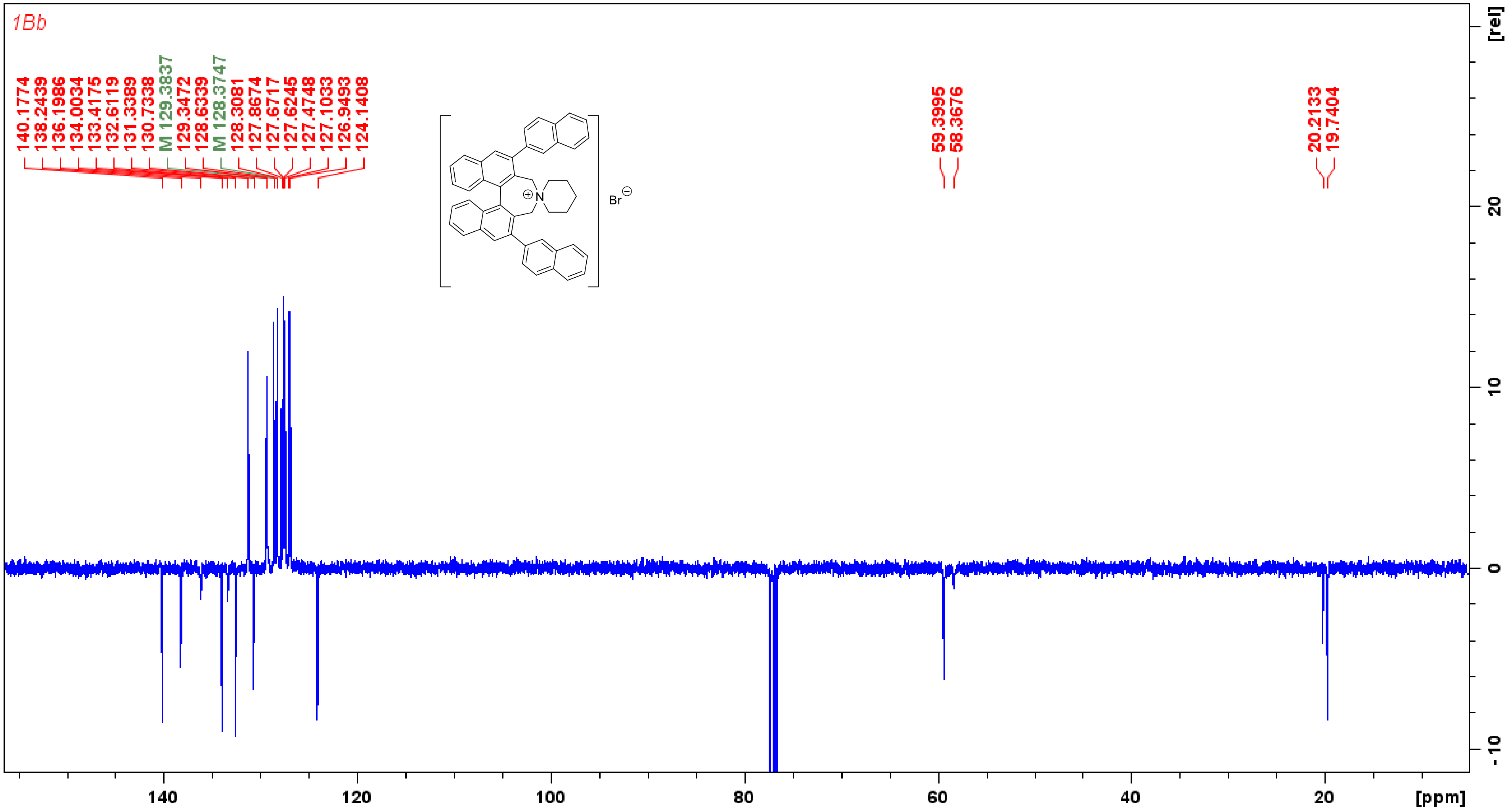
1Bb
140.1774
138.2439
136.1986
134.0034
133.4175
132.6119
131.3389
130.7338
M 129.3837
129.3472
128.6339
M 128.3747
128.3081
127.8674
127.6717
127.6245
127.4748
127.1033
126.9493
124.1408
59.3995
58.3676
20.2133
19.7404
Br
N
[rel]
20
10
0
-10
140
120
100
80
60
40
20
[ppm]

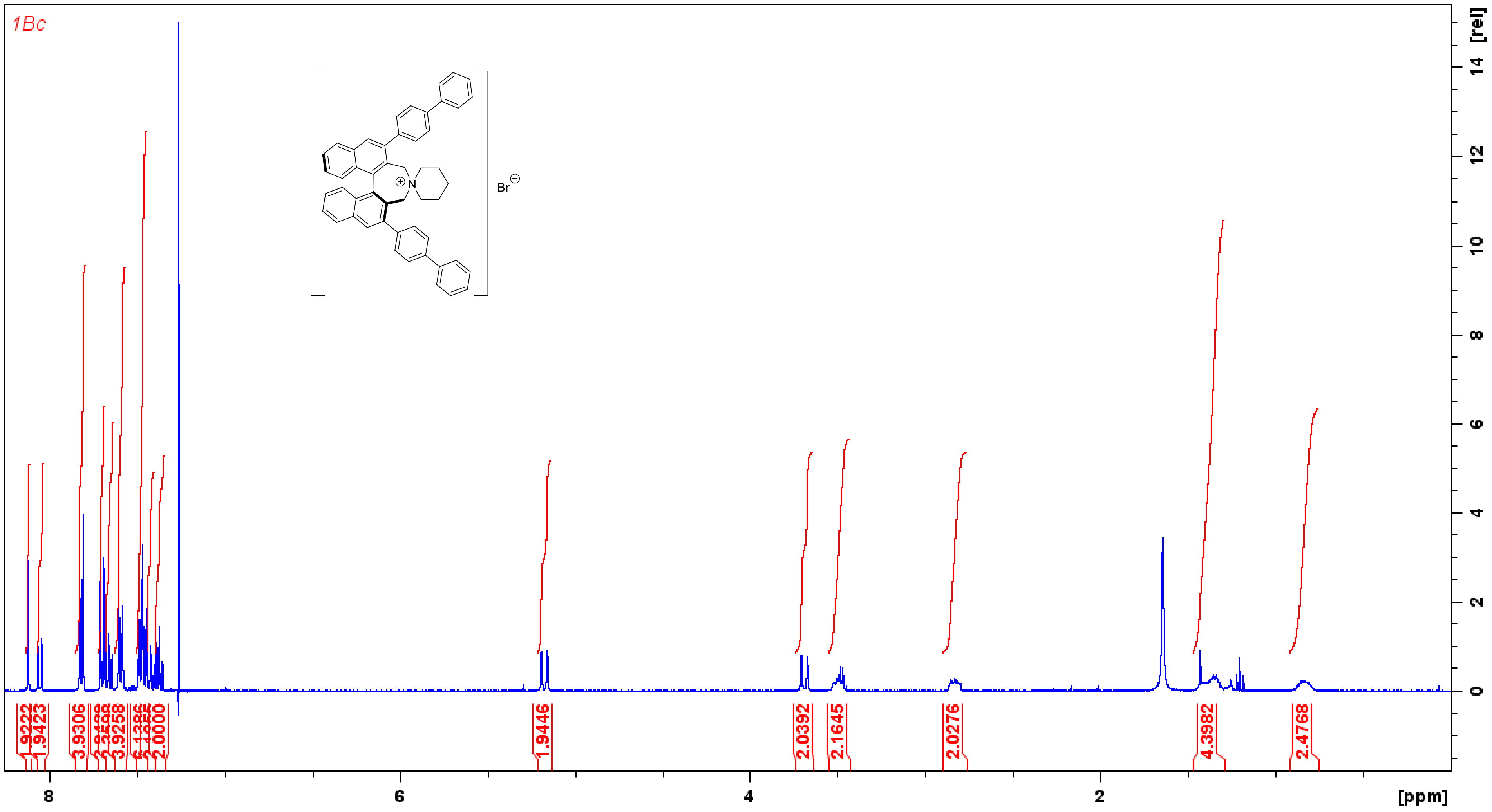
1Bc
Br
[rel]
[ppm]
8
6
4
2
14
12
10
8
6
4
2
0
1.9446
2.0392
2.1645
2.0276
4.3982
2.4768
3.9306
3.9258
2.0000

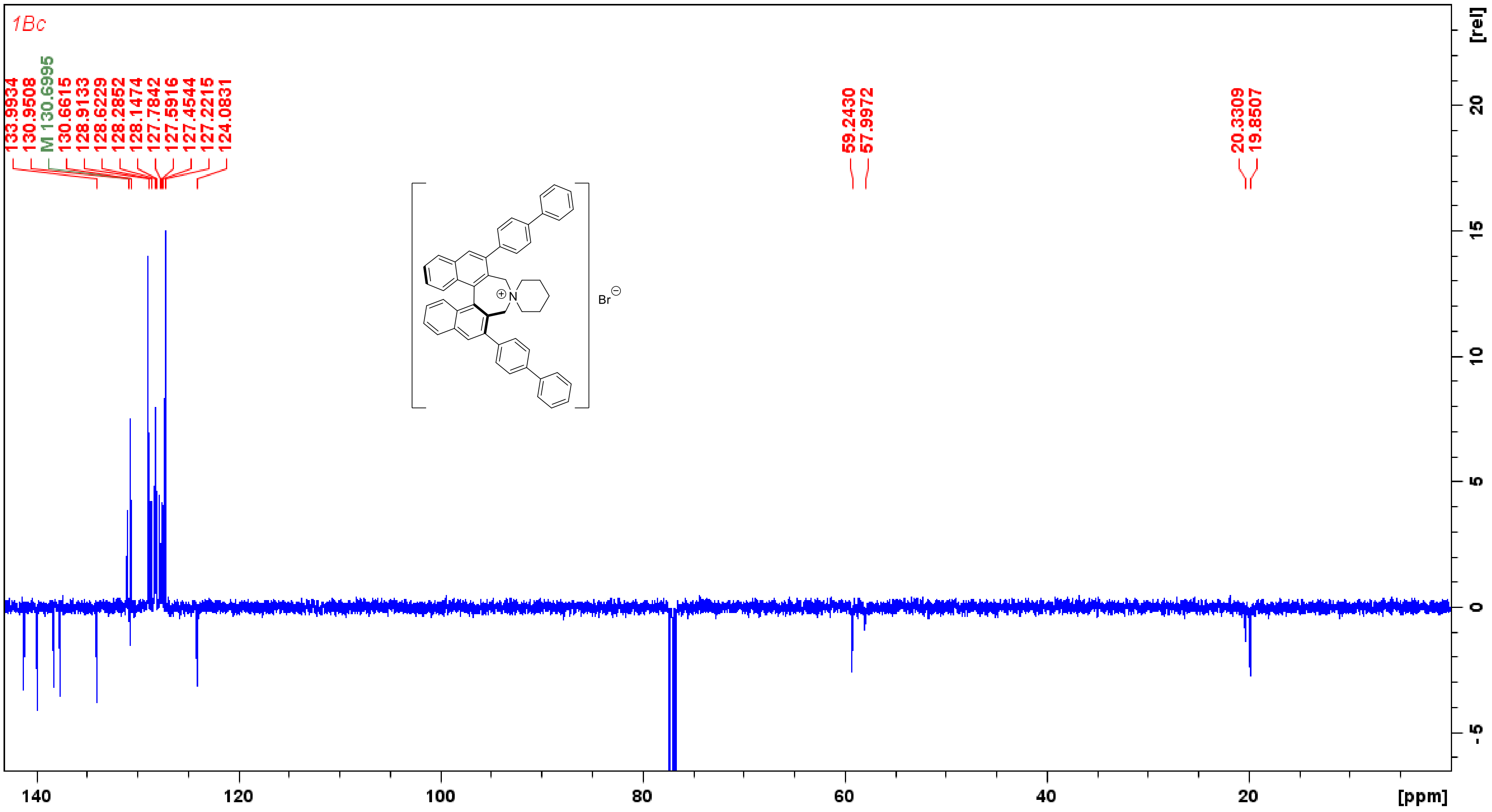
1Bc
133.9934
130.9508
M 130.6995
130.6615
128.9133
128.6229
128.2852
128.1474
127.7842
127.5916
127.4544
127.2215
124.0831
59.2430
57.9972
20.3309
19.8507
Br
N
[rel]
20
15
10
5
0
-5
140
120
100
80
60
40
20
[ppm]

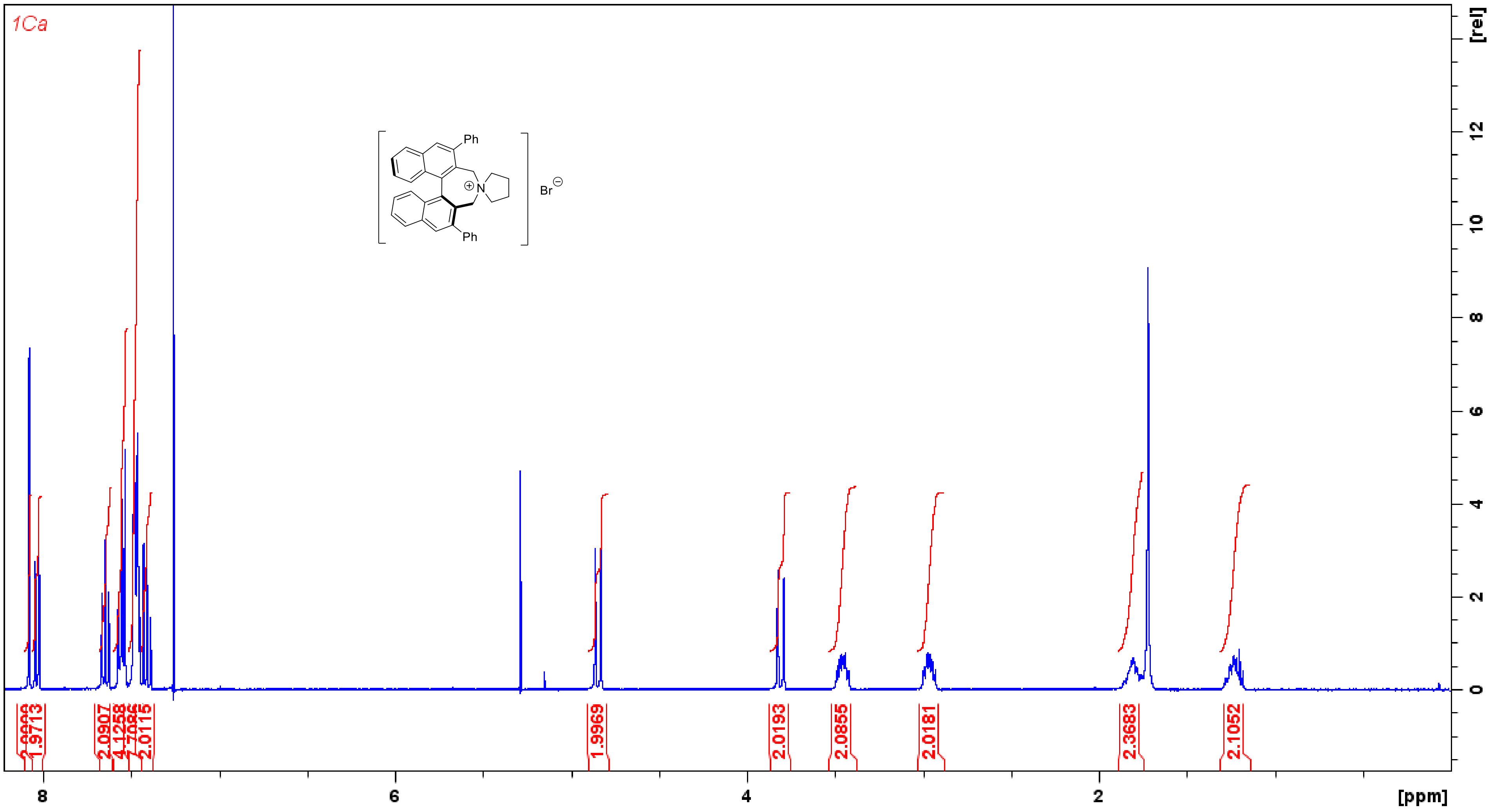
1Ca
Ph
N
Ph
Br
1.9713
2.0907
4.1258
2.0115
1.9969
2.0193
2.0855
2.0181
2.3683
2.1052
8
6
4
2
[ppm]
0
2
4
6
8
10
12
[rel]

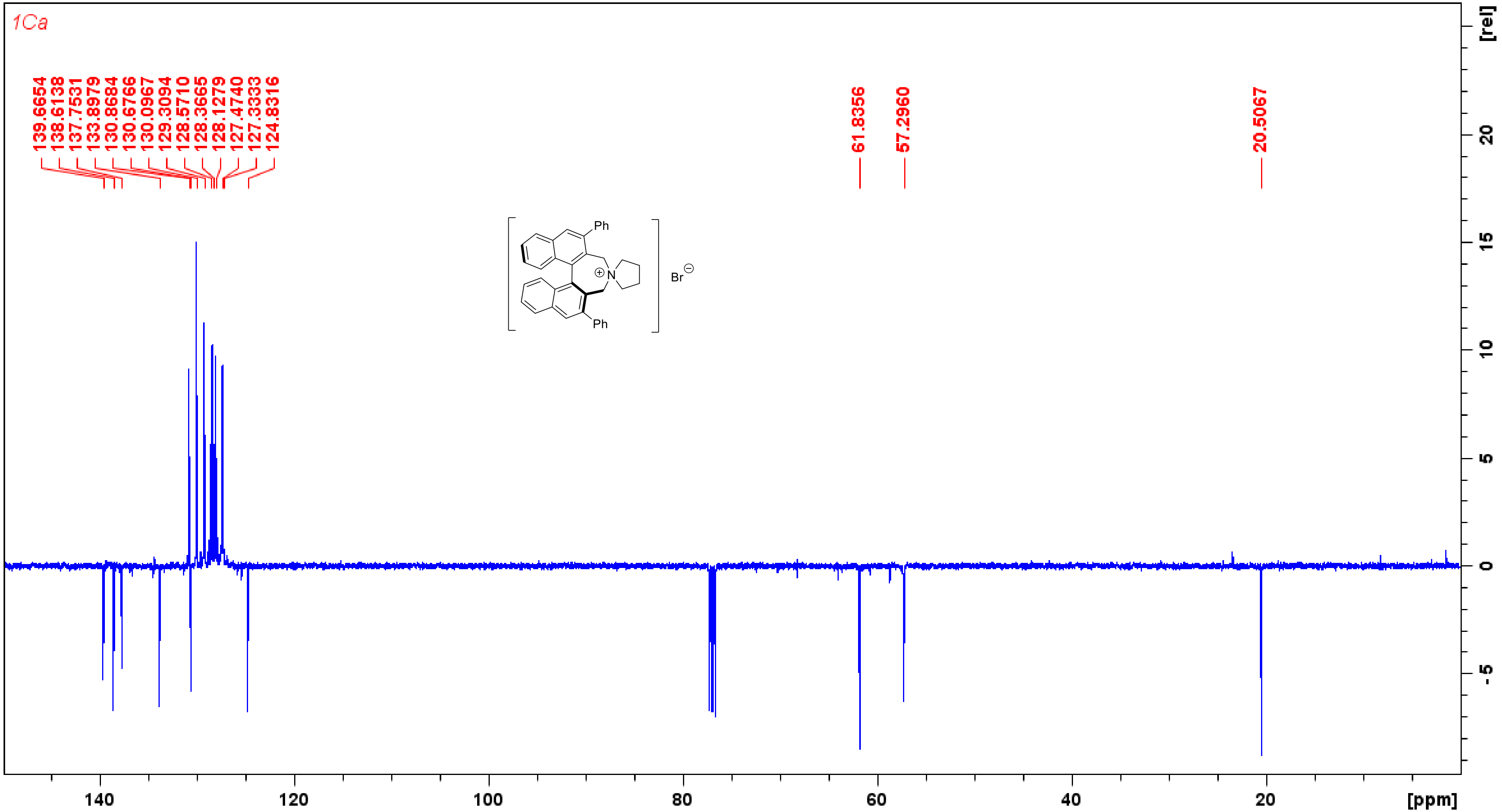
1Ca
139.6654
138.6138
137.7531
133.8979
130.8684
130.6766
130.0967
129.3094
128.5710
128.3665
128.1279
127.4740
127.3333
124.8316
61.8356
57.2960
20.5067
Ph
N
Br
Ph
[rel]
20
15
10
5
0
-5
140
120
100
80
60
40
20
[ppm]

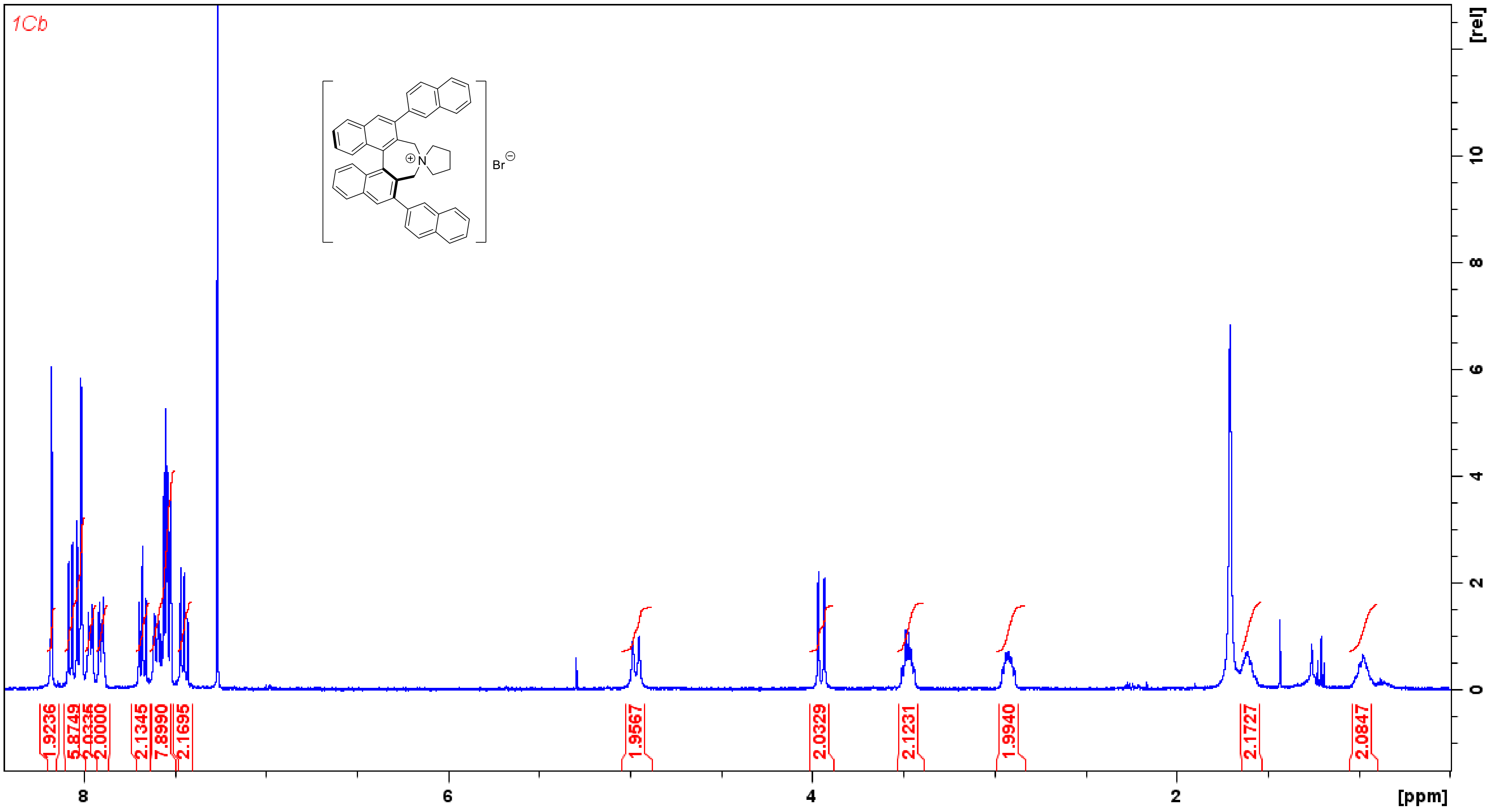
1Cb
Br⊖
1.9236
5.8749
2.0336
2.0000
2.1345
7.8990
2.1695
1.9567
2.0329
2.1231
1.9940
2.1727
2.0847
8
6
4
2
[ppm]
[rel]
0
2
4
6
8
10

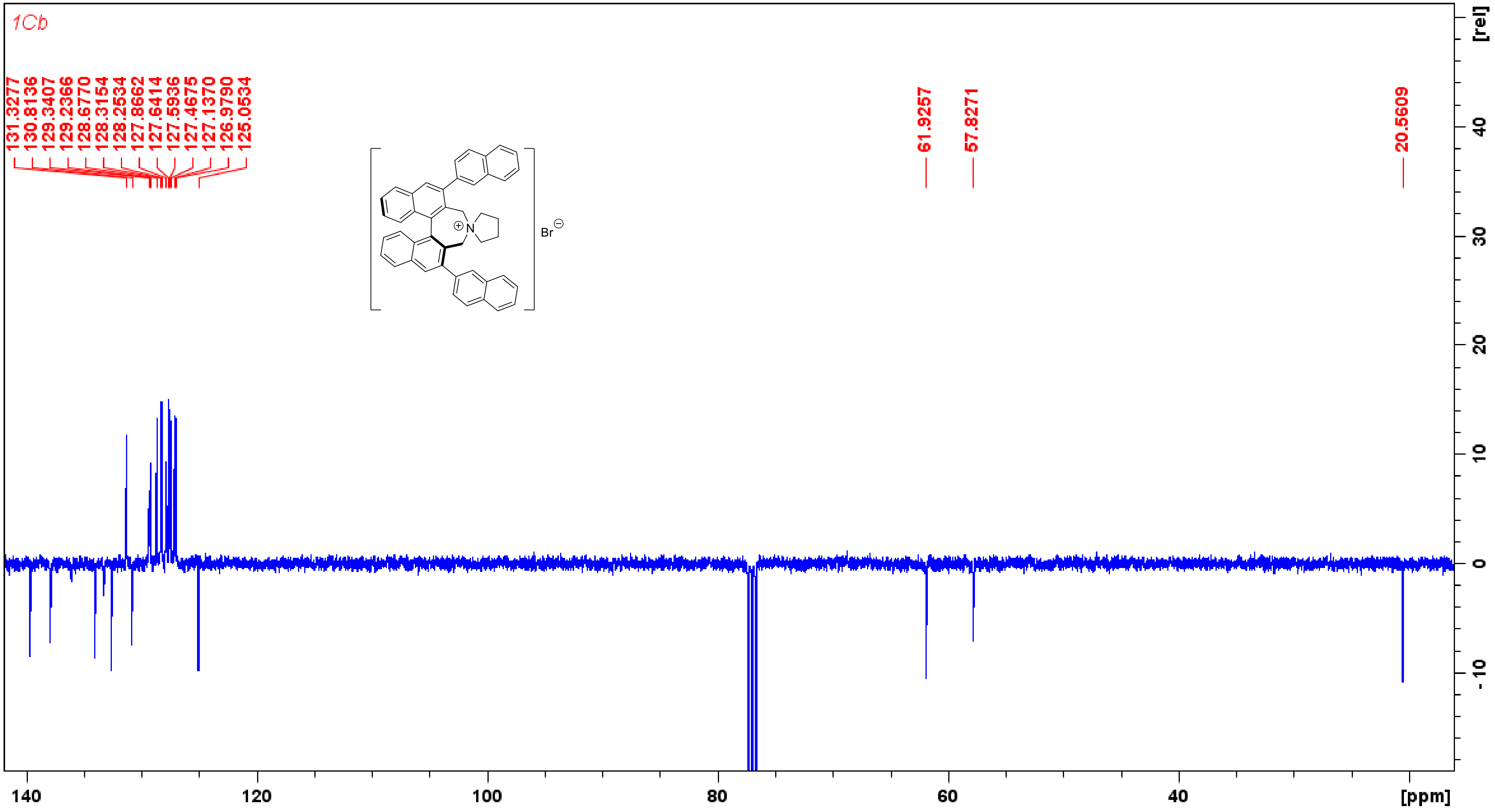
1Cb
131.3277
130.8136
129.3407
129.2366
128.6770
128.3154
128.2534
127.8662
127.6414
127.5936
127.4675
127.1370
126.9790
125.0534
61.9257
57.8271
20.5609
Br
N
[rel]
40
30
20
10
0
-10
140
120
100
80
60
40
[ppm]

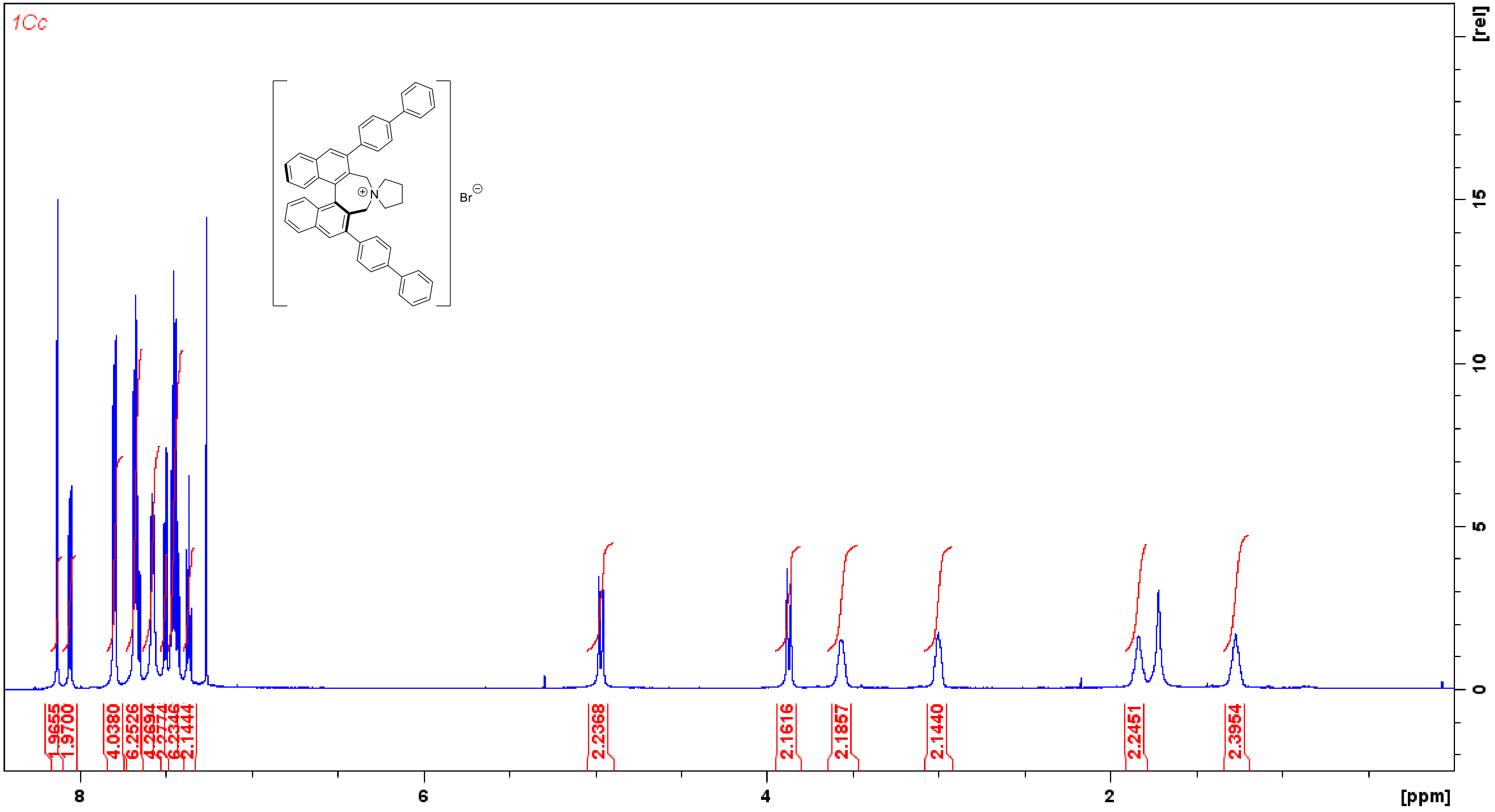
1Cc
Br
[rel]
[ppm]
1.9655
1.9700
4.0380
6.2526
4.2694
2.2774
6.2346
2.1444
2.2368
2.1616
2.1857
2.1440
2.2451
2.3954

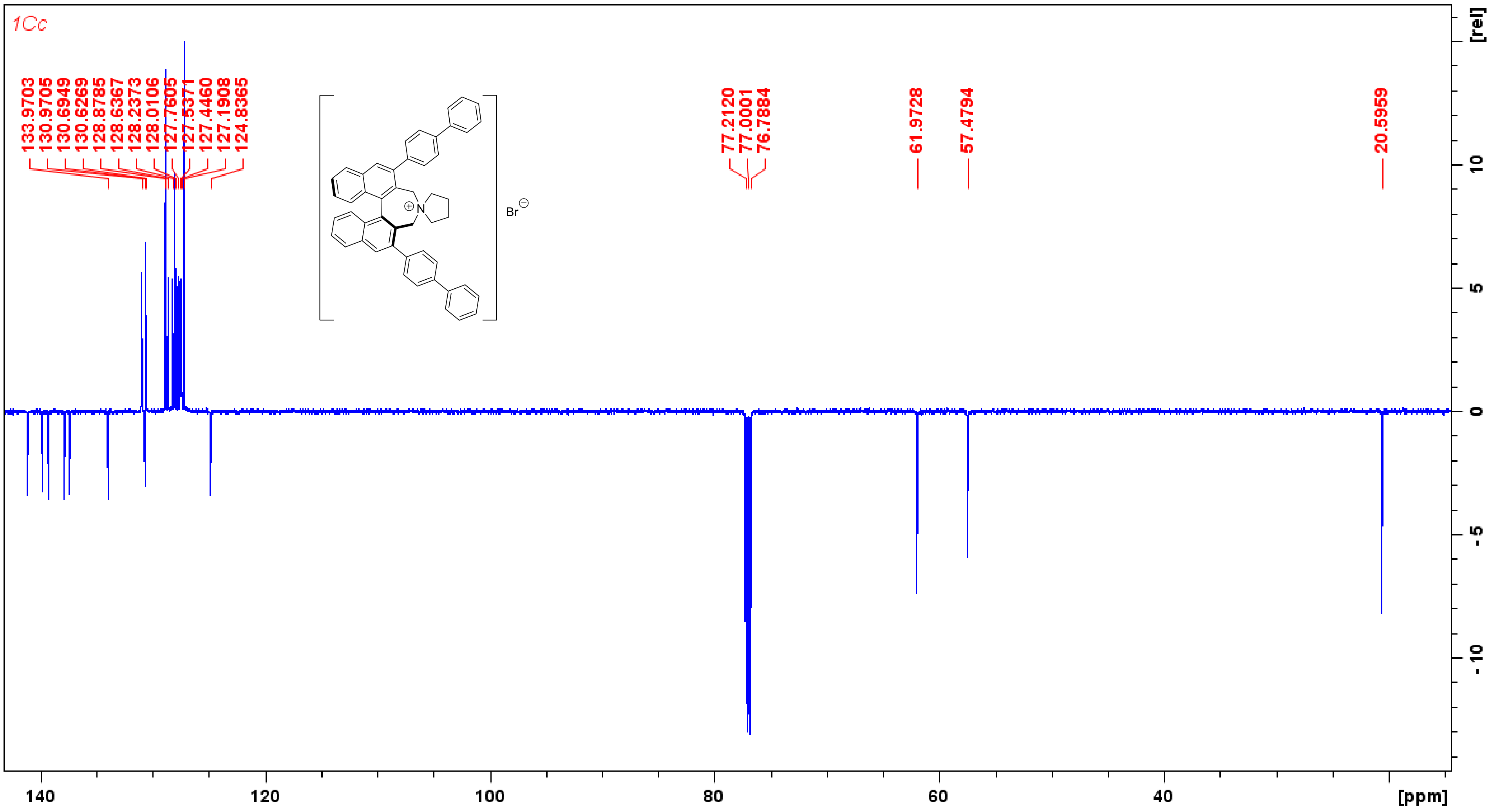
1Cc
133.9703
130.9705
130.6949
130.6269
128.8785
128.6367
128.2373
128.0106
127.7605
127.5371
127.4460
127.1908
124.8365
77.2120
77.0001
76.7884
61.9728
57.4794
20.5959
Br
N
[rel]
10
5
0
-5
-10
140
120
100
80
60
40
[ppm]

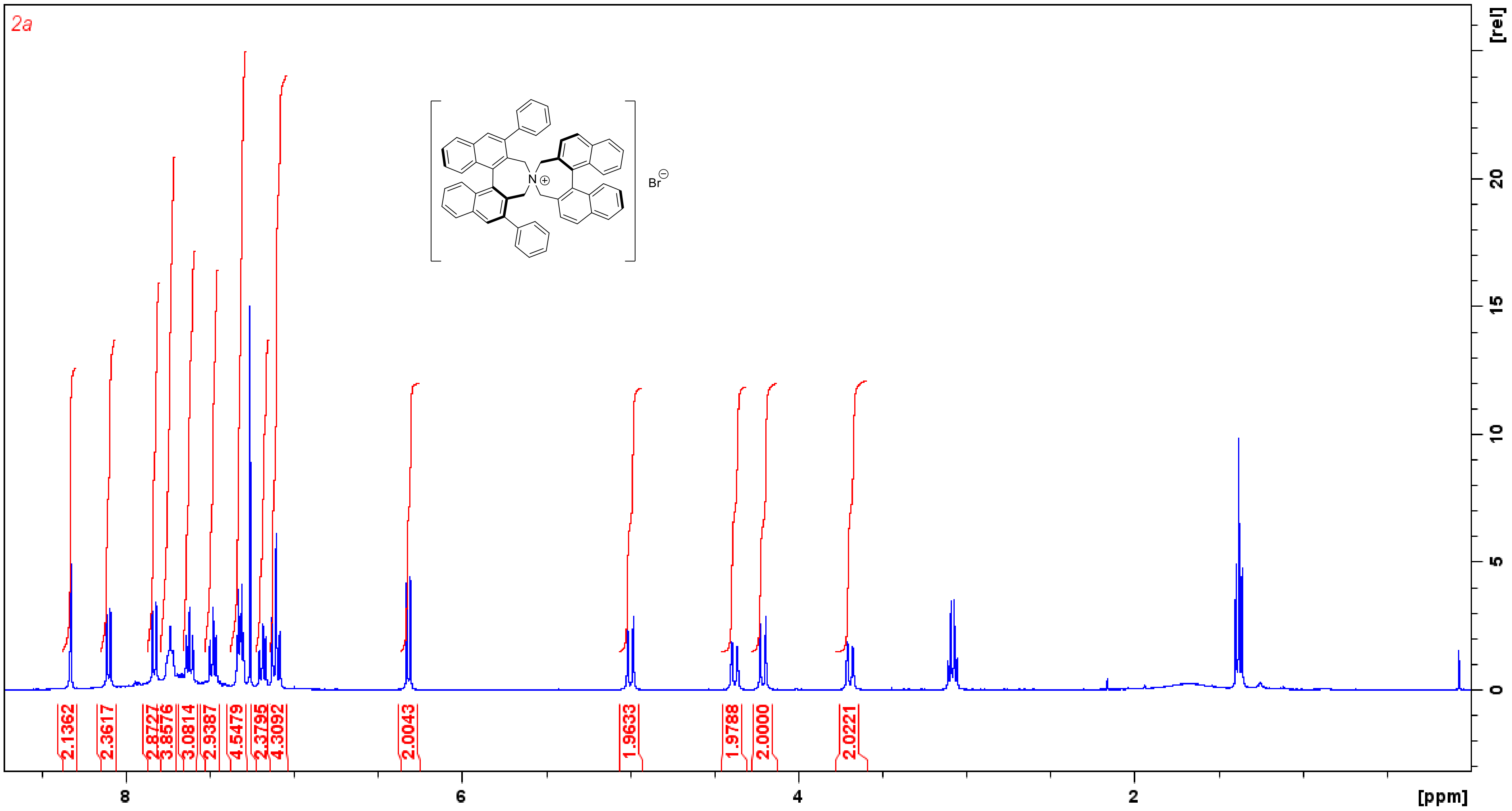

2a
Br
2.1362
2.3617
2.8727
3.8576
3.0814
2.9387
4.5479
2.3795
4.3092
2.0043
1.9633
1.9788
2.0000
2.0221
8
6
4
2
[ppm]
[rel]
20
15
10
5
0

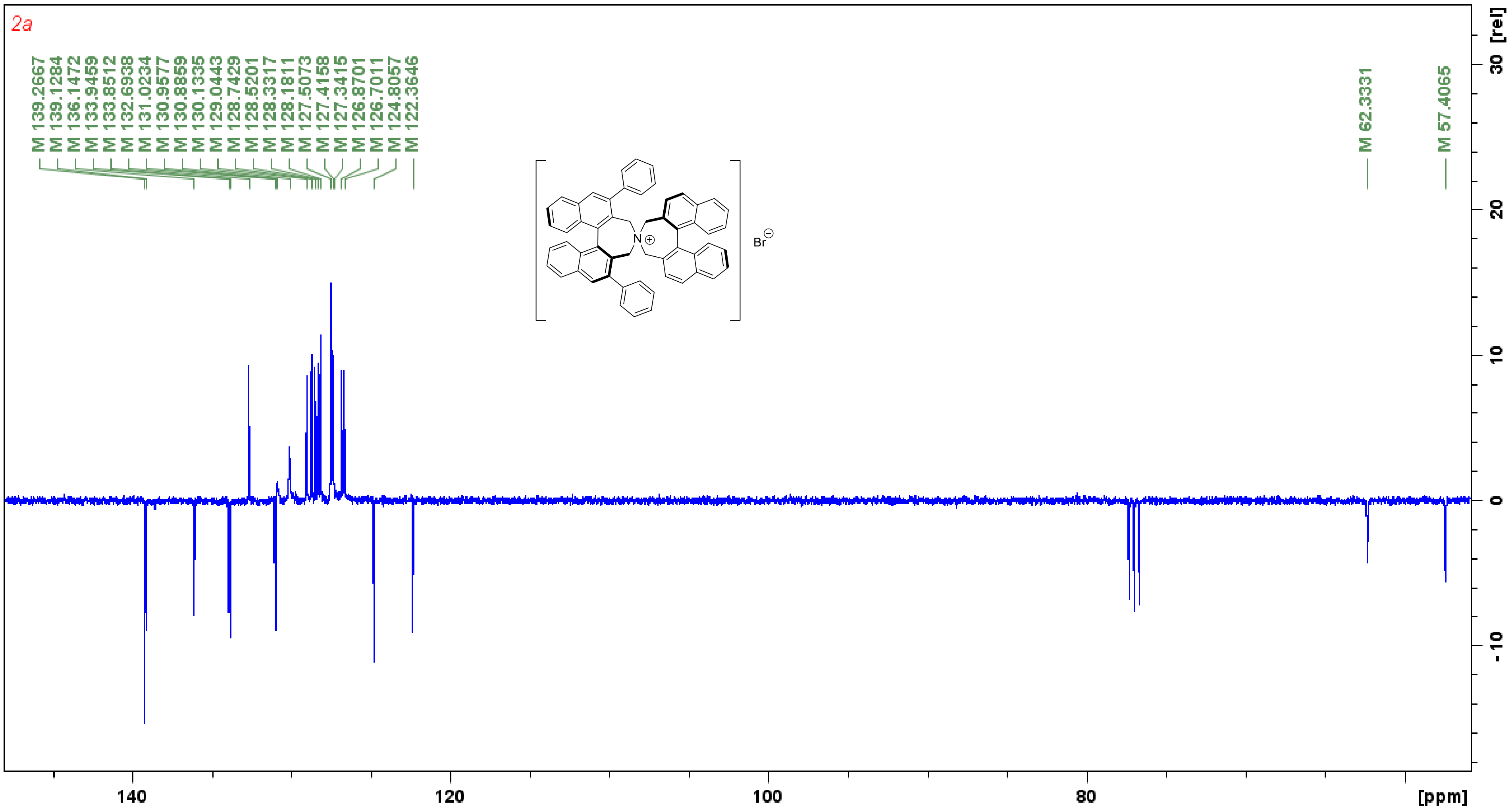
2a
M 139.2667
M 139.1284
M 136.1472
M 133.9459
M 133.8512
M 132.6938
M 131.0234
M 130.9577
M 130.8859
M 130.1335
M 129.0443
M 128.7429
M 128.5201
M 128.3317
M 128.1811
M 127.5073
M 127.4158
M 127.3415
M 126.8701
M 126.7011
M 124.8057
M 122.3646
M 62.3331
M 57.4065
N
Br
140
120
100
80
[ppm]
30
20
10
0
-10
[rel]

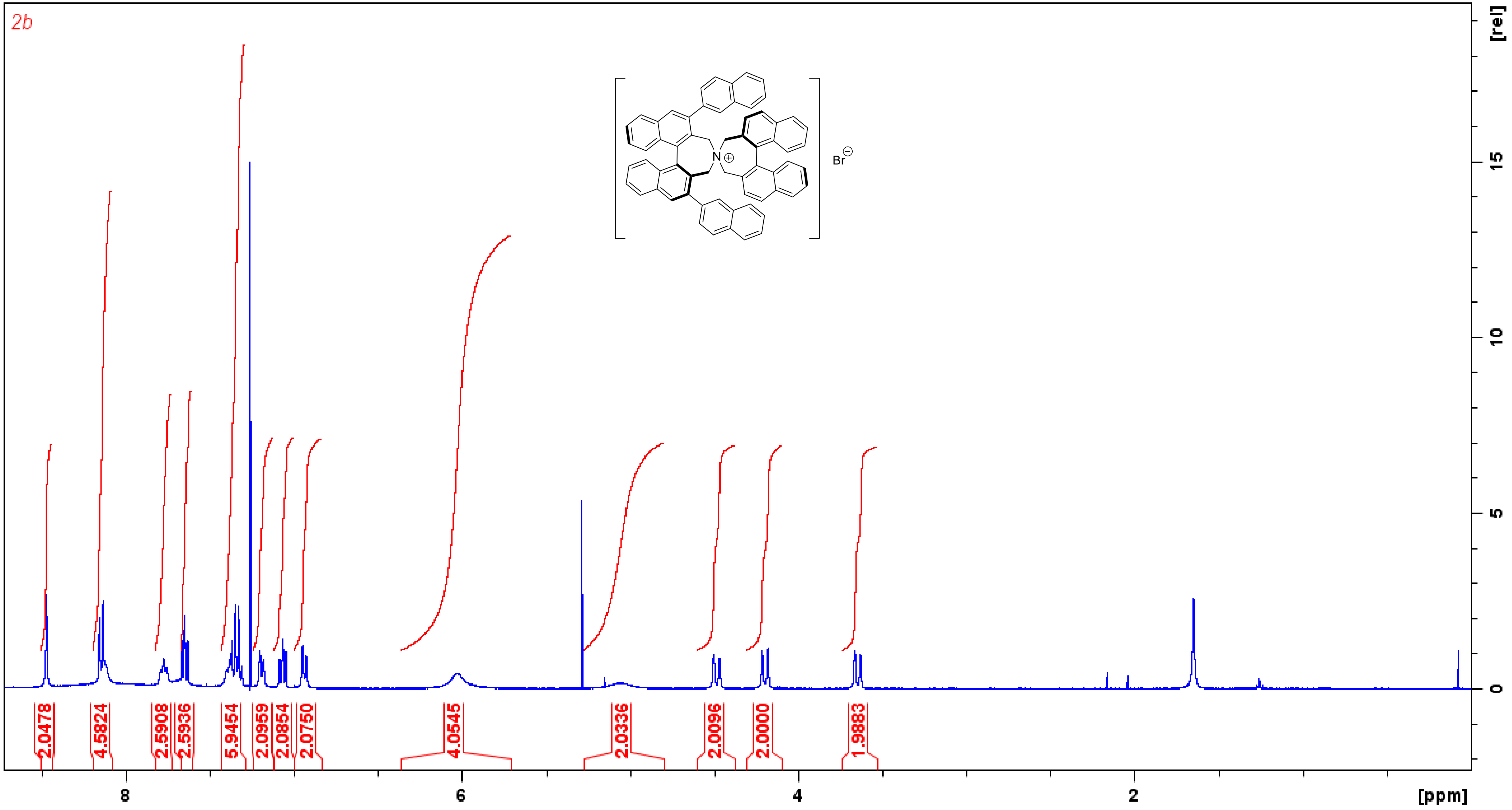
2b
Br
[rel]
15
10
5
0
2.0478
4.5824
2.5908
2.5936
5.9454
2.0959
2.0854
2.0750
4.0545
2.0336
2.0096
2.0000
1.9883
8
6
4
2
[ppm]

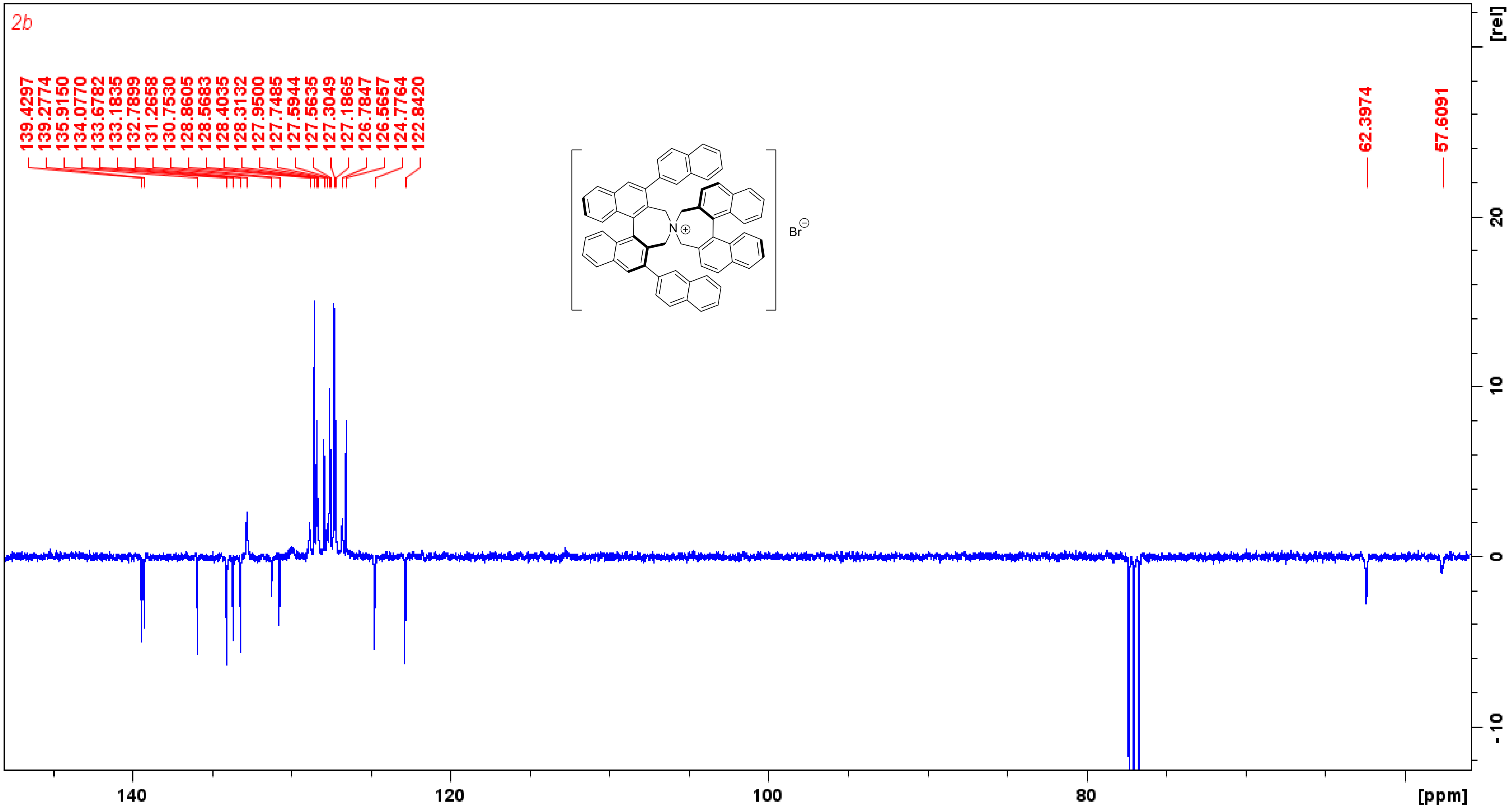
2b
139.4297
139.2774
135.9150
134.0770
133.6782
133.1835
132.7899
131.2658
130.7530
128.8605
128.5683
128.4035
128.3132
127.9500
127.7485
127.5944
127.5635
127.3049
127.1865
126.7847
126.5657
124.7764
122.8420
62.3974
57.6091
N⊕
Br⊖
[rel]
20
10
0
-10
140
120
100
80
[ppm]

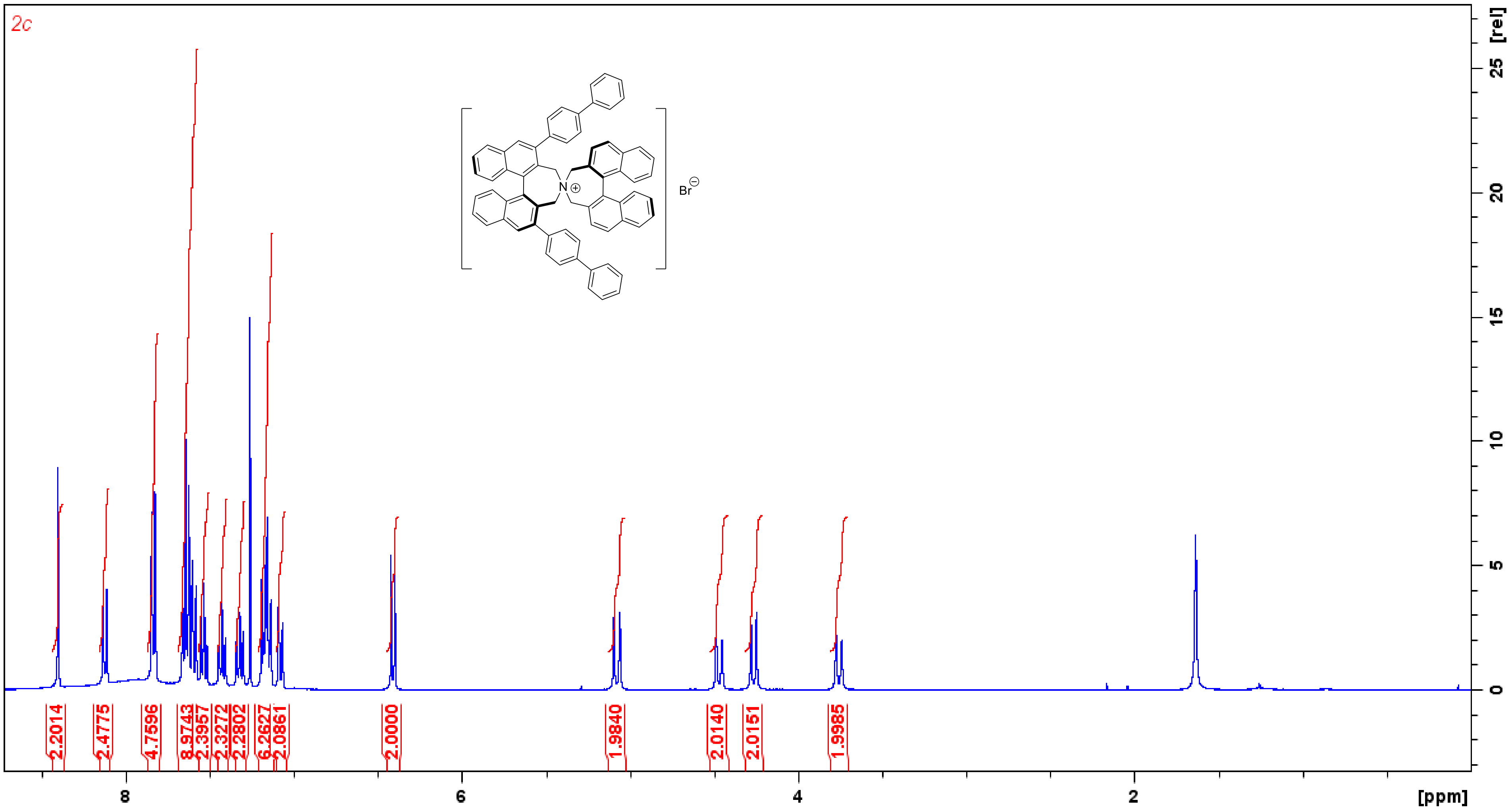

2c
Br
N
2.2014
2.4775
4.7596
8.9743
2.3957
2.3272
2.2802
6.2627
2.0861
2.0000
1.9840
2.0140
2.0151
1.9985
8
6
4
2
[ppm]
25
20
15
10
5
0
[rel]

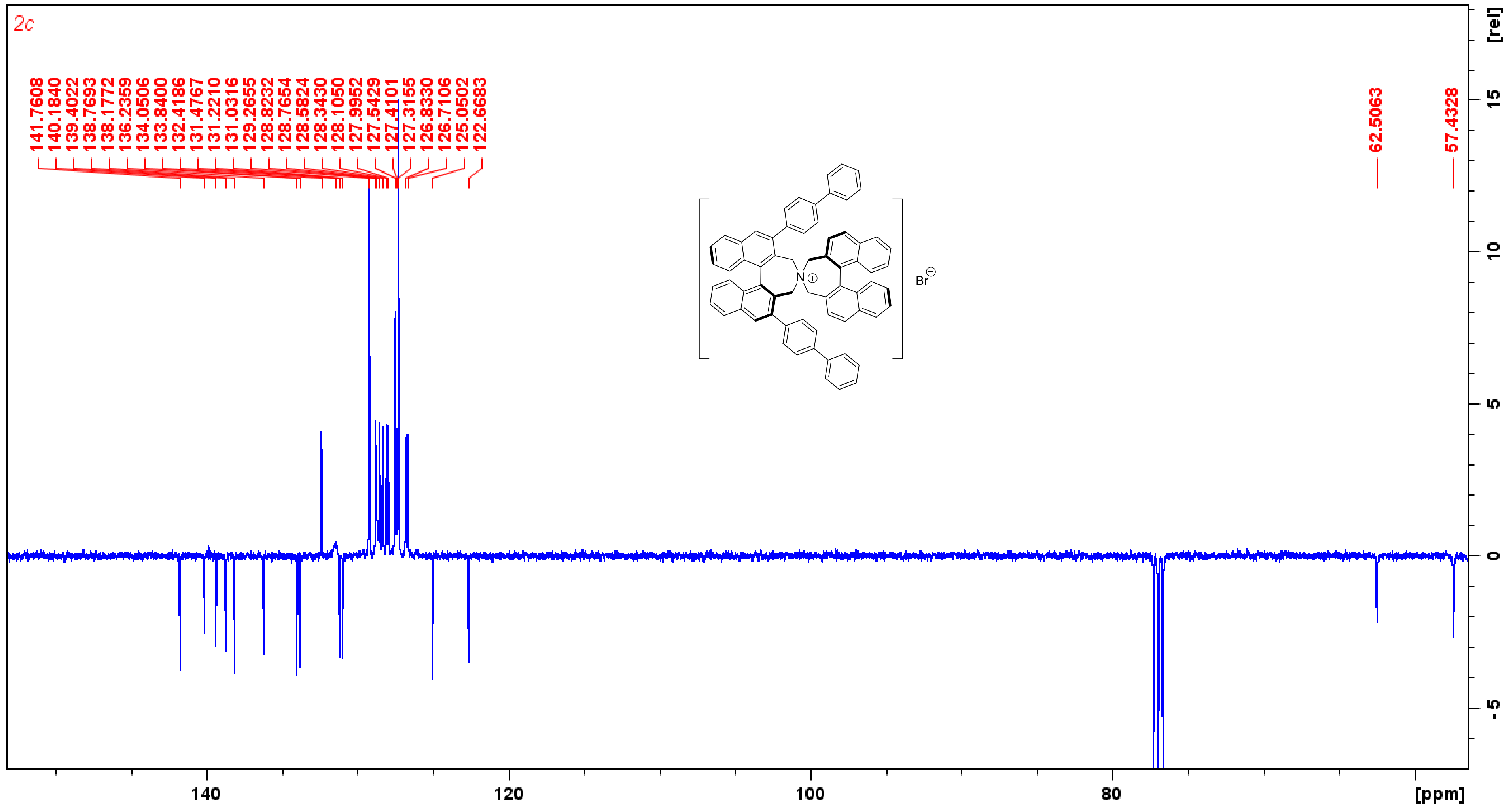
2c
141.7608
140.1840
139.4022
138.7693
138.1772
136.2359
134.0506
133.8400
132.4186
131.4767
131.2210
131.0316
129.2655
128.8232
128.7654
128.5824
128.3430
128.1050
127.9952
127.5429
127.4101
127.3155
126.8330
126.7106
125.0502
122.6683
62.5063
57.4328
N⊕
Br⊖
[rel]
15
10
5
0
-5
140
120
100
80
[ppm]

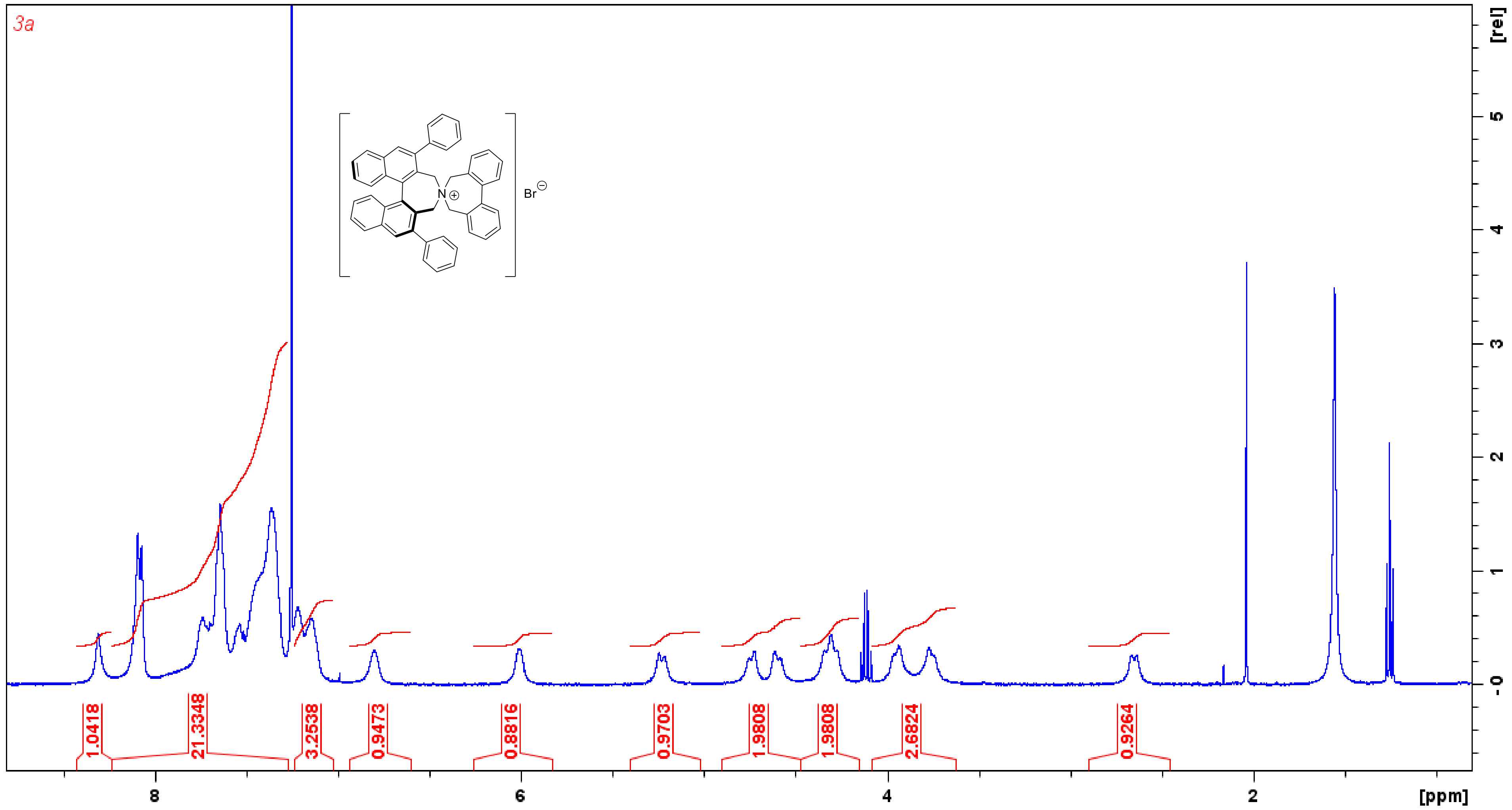
3a
Br⊖
N⊕
1.0418
21.3348
3.2538
0.9473
0.8816
0.9703
1.9808
1.9808
2.6824
0.9264
8
6
4
2
[ppm]
[rel]
5
4
3
2
1
0

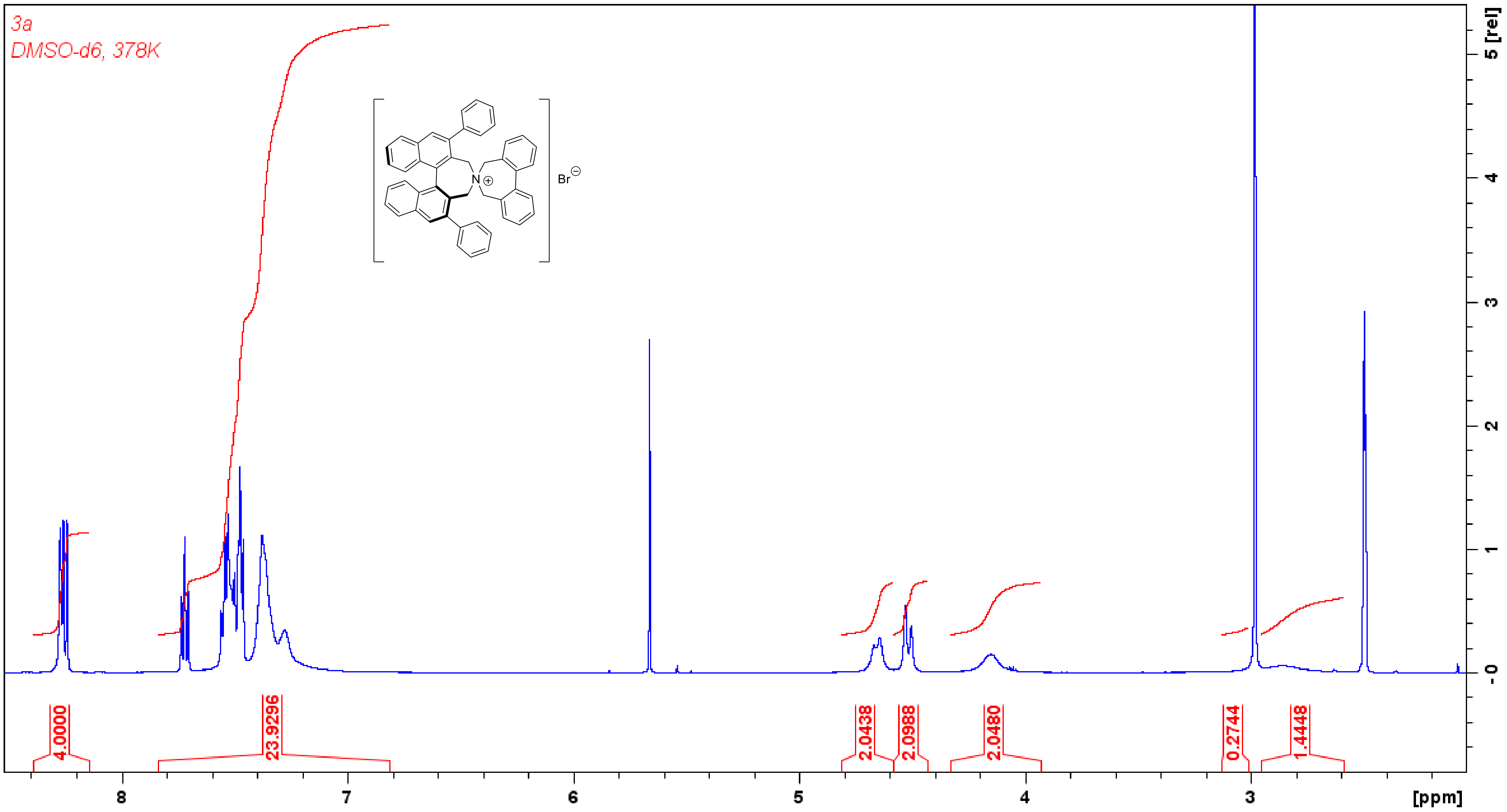
3a
DMSO-d6, 378K
Br
4.0000
23.9296
2.0438
2.0988
2.0480
0.2744
1.4448
8
7
6
5
4
3
[ppm]
5 [rel]
4
3
2
1
0

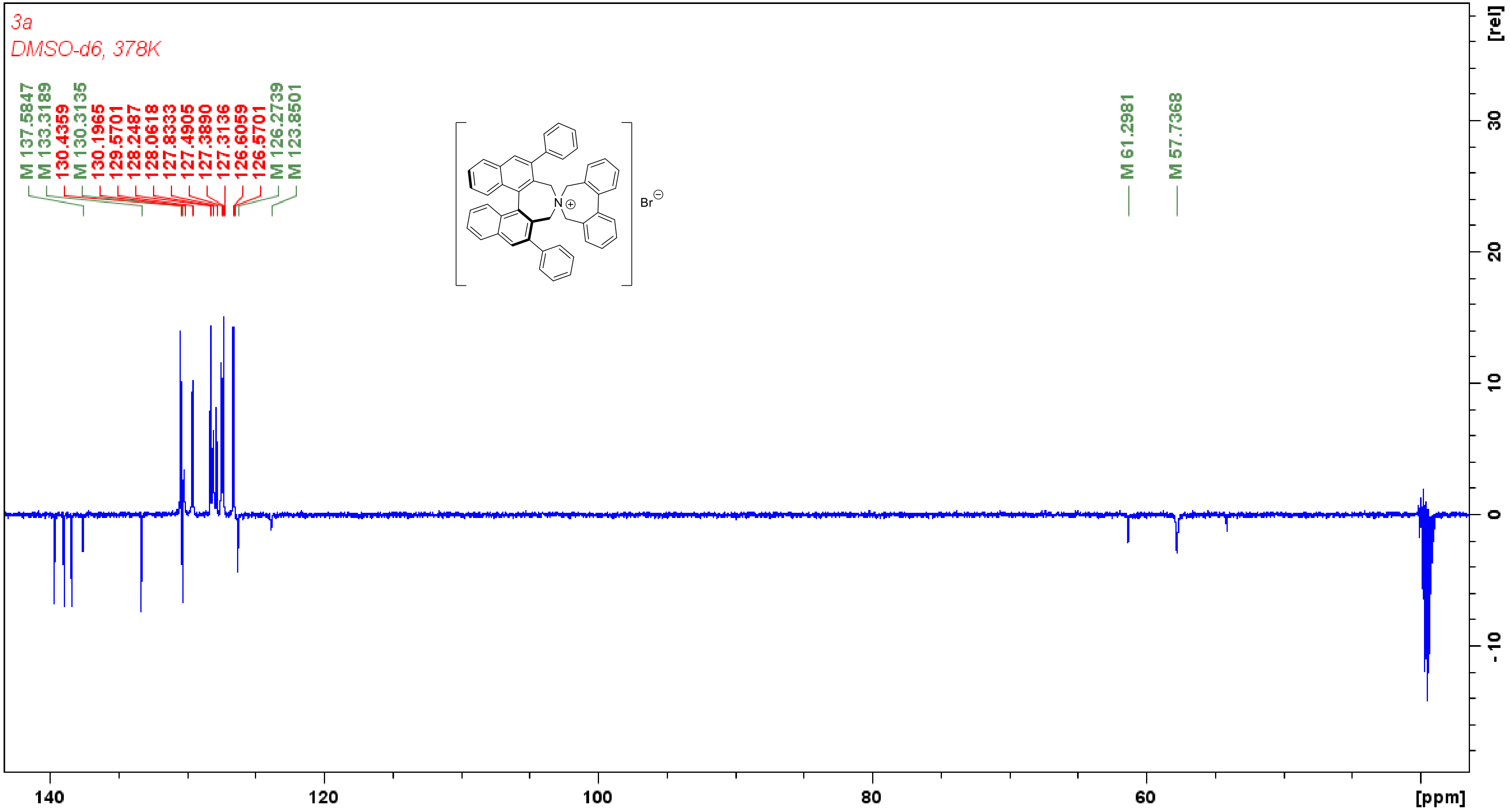
3a
DMSO-d6, 378K
M 137.5847
M 133.3189
130.4359
M 130.3135
130.1965
129.5701
128.2487
128.0618
127.8333
127.4905
127.3890
127.3136
126.6059
126.5701
M 126.2739
M 123.8501
M 61.2981
M 57.7368
Br
N
[rel]
[ppm]
140
120
100
80
60
30
20
10
0
-10

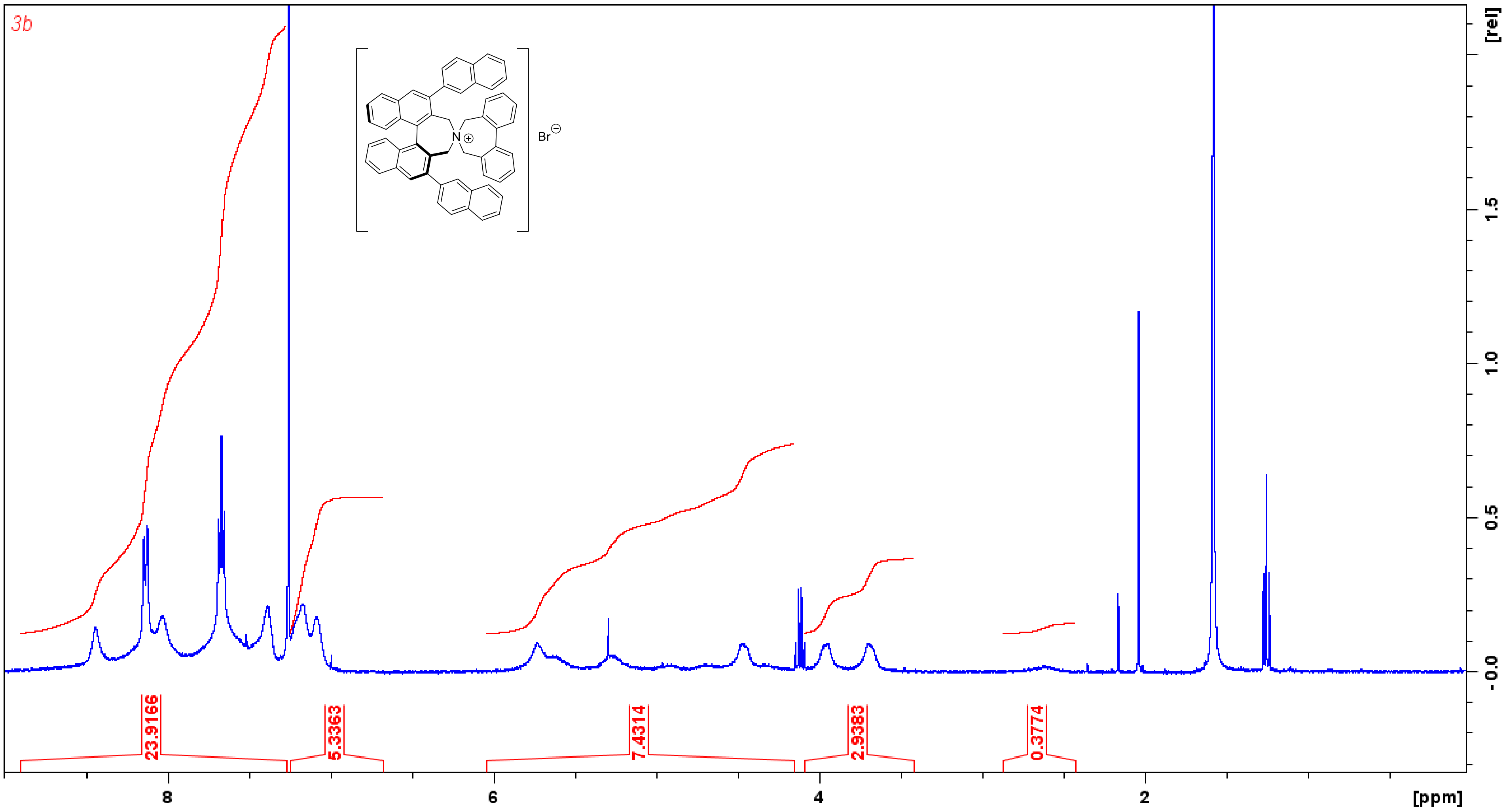
3b
Br
[rel]
1.5
1.0
0.5
-0.0
23.9166
5.3363
7.4314
2.9383
0.3774
8
6
4
2
[ppm]

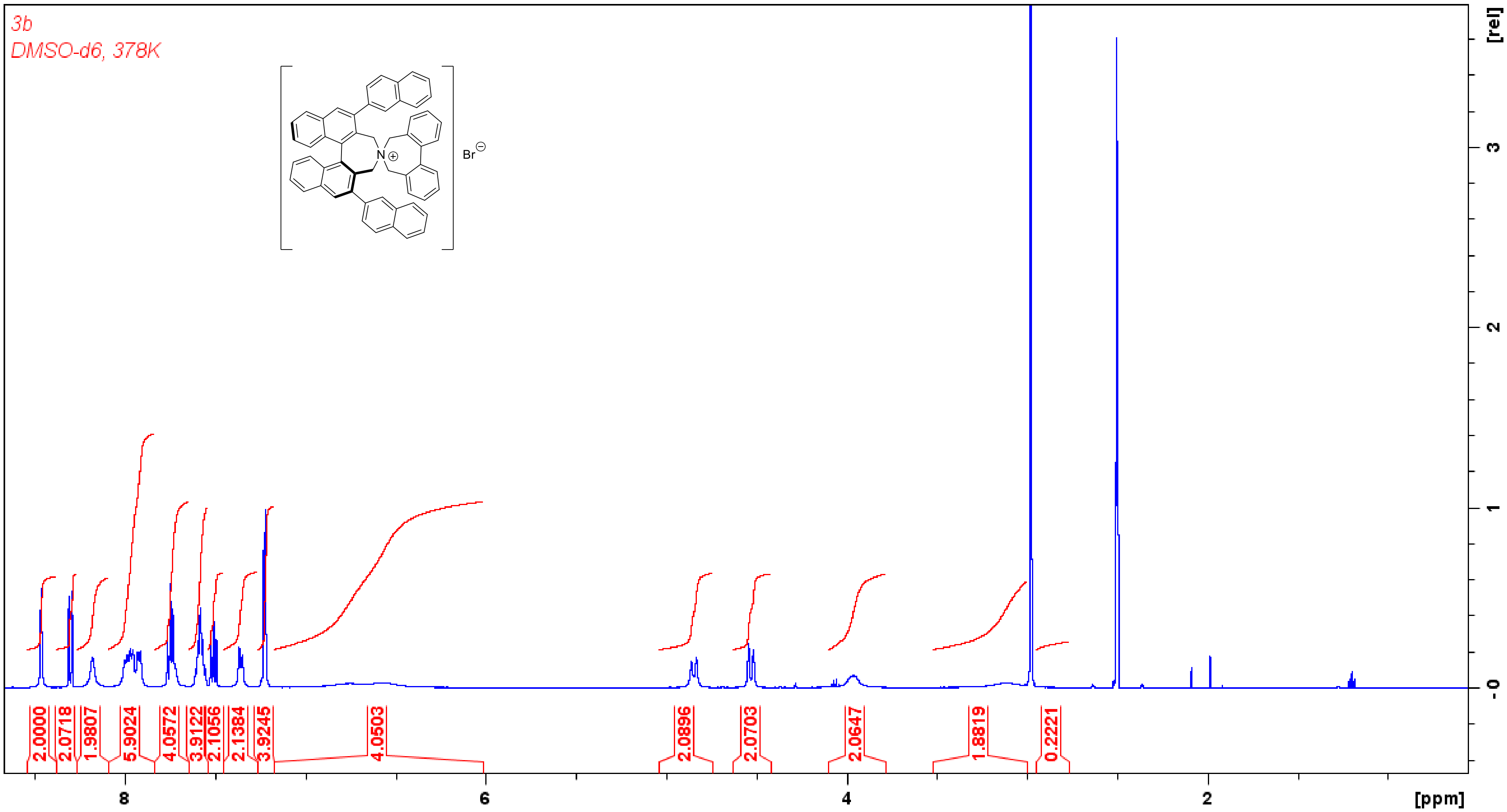
3b
DMSO-d6, 378K
Br
N
2.0000
2.0718
1.9807
5.9024
4.0572
3.9122
2.1056
2.1384
3.9245
4.0503
2.0896
2.0703
2.0647
1.8819
0.2221
8
6
4
2
[ppm]
[rel]
3
2
1
0

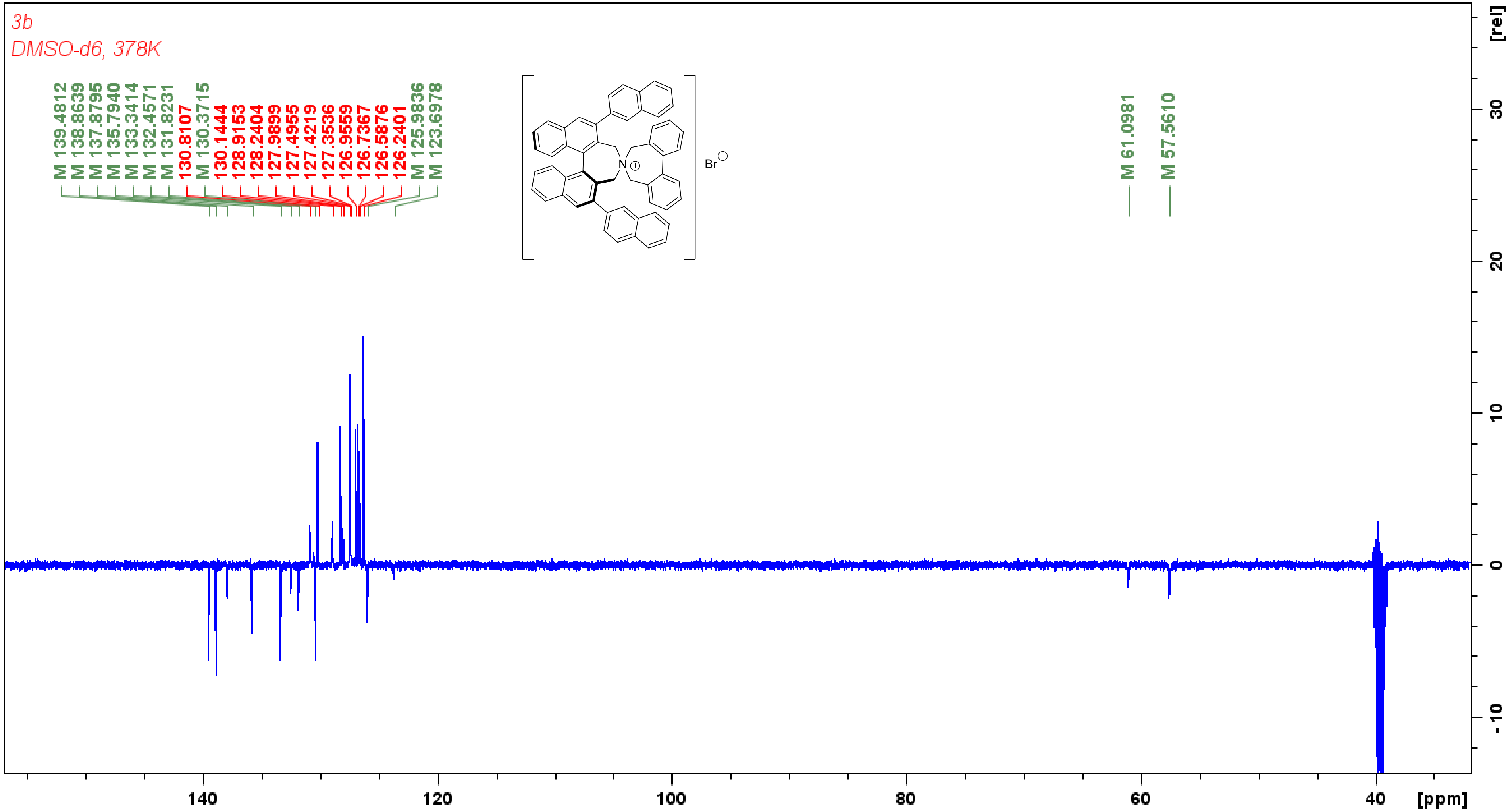
3b
DMSO-d6, 378K
M 139.4812
M 138.8639
M 137.8795
M 135.7940
M 133.3414
M 132.4571
M 131.8231
130.8107
M 130.3715
130.1444
128.9153
128.2404
127.9899
127.4955
127.4219
127.3536
126.9559
126.7367
126.5876
126.2401
M 125.9836
M 123.6978
Br
M 61.0981
M 57.5610
[rel]
140
120
100
80
60
40
[ppm]

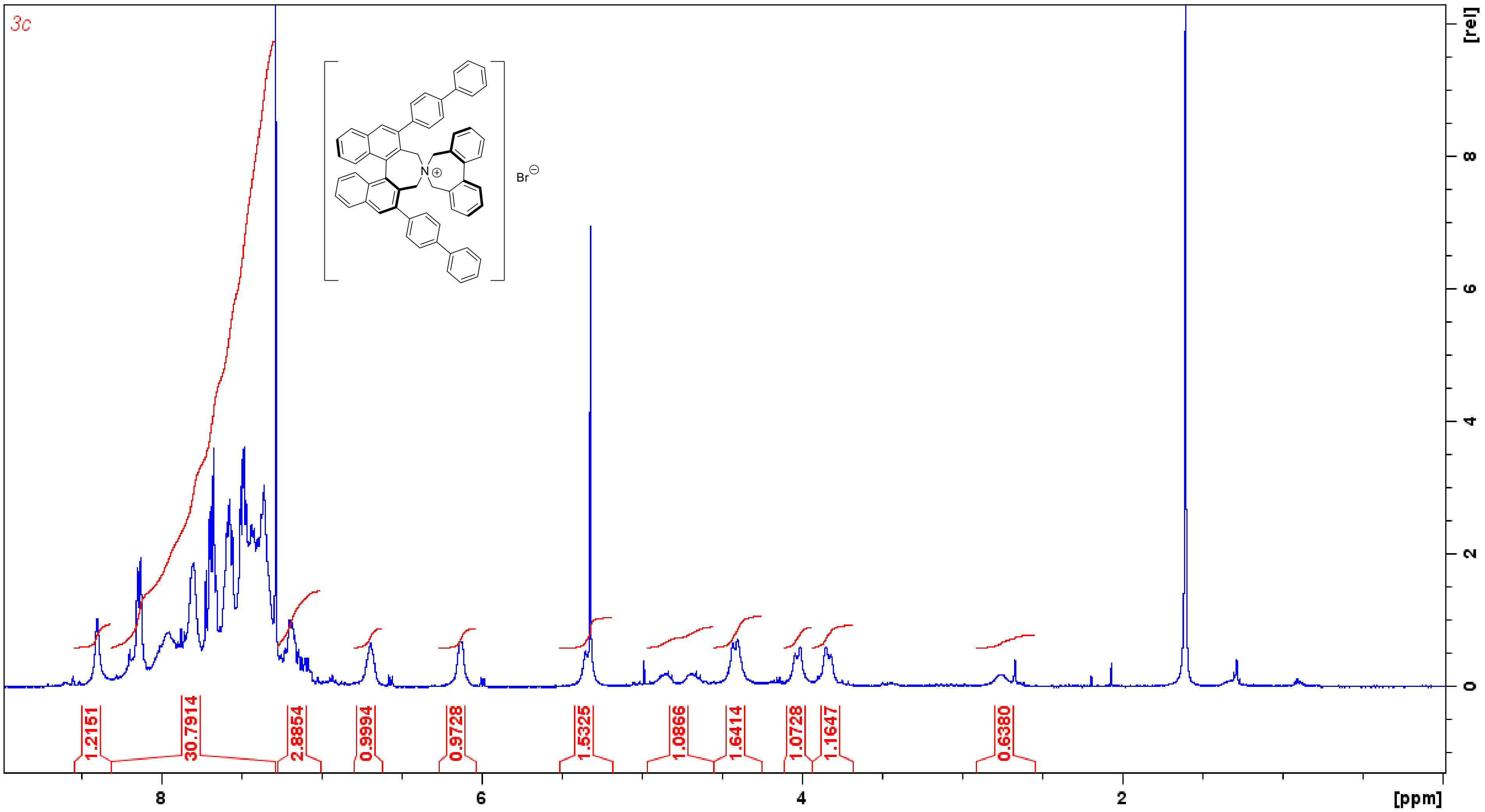
3c
Br
[rel]
[ppm]
1.2151
30.7914
2.8854
0.9994
0.9728
1.5325
1.0866
1.6414
1.0728
1.1647
0.6380

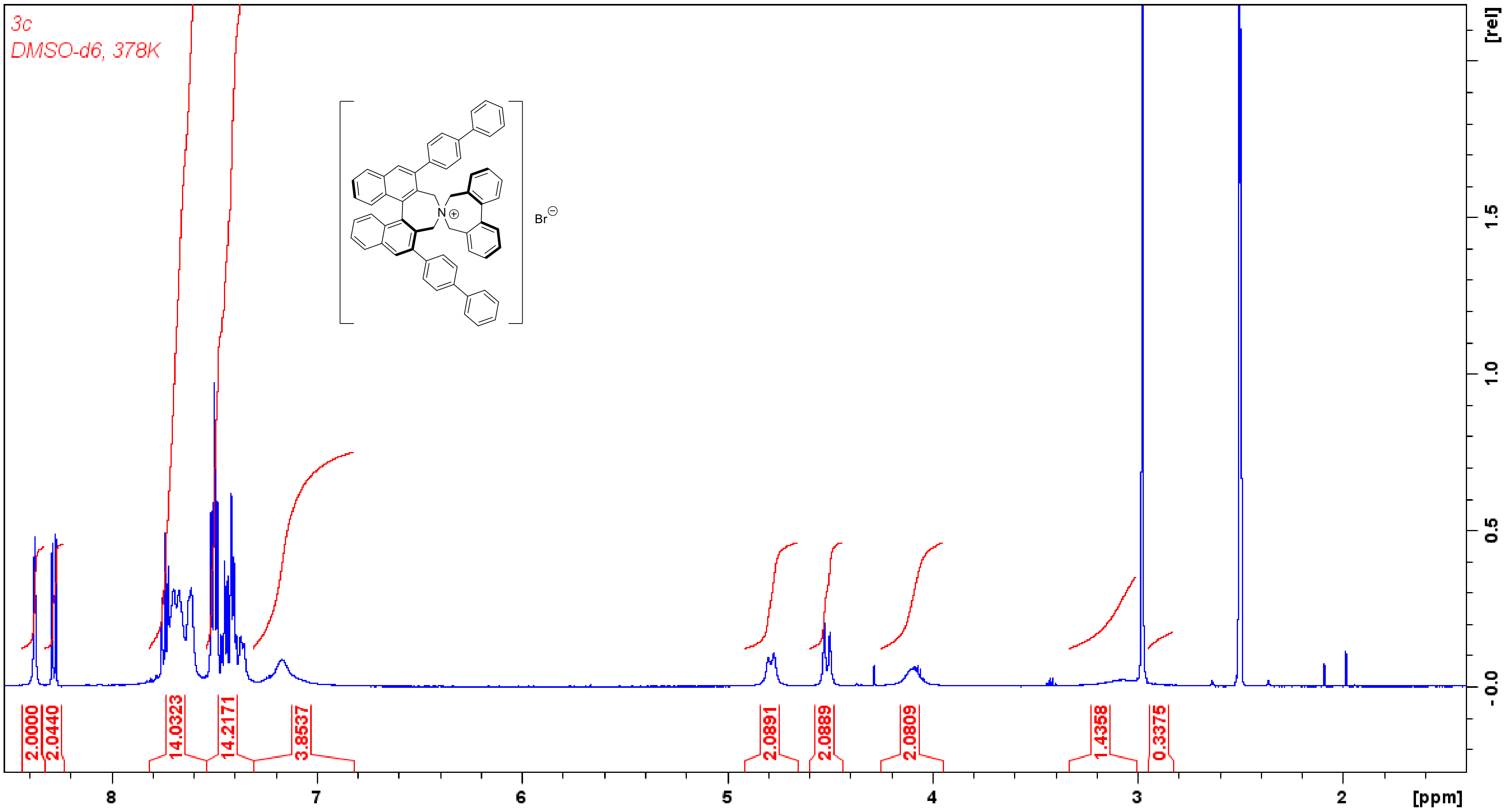
3c
DMSO-d6, 378K
Br⊖
N⊕
2.0000
2.0440
14.0323
14.2171
3.8537
2.0891
2.0889
2.0809
1.4358
0.3375
[rel]
[ppm]

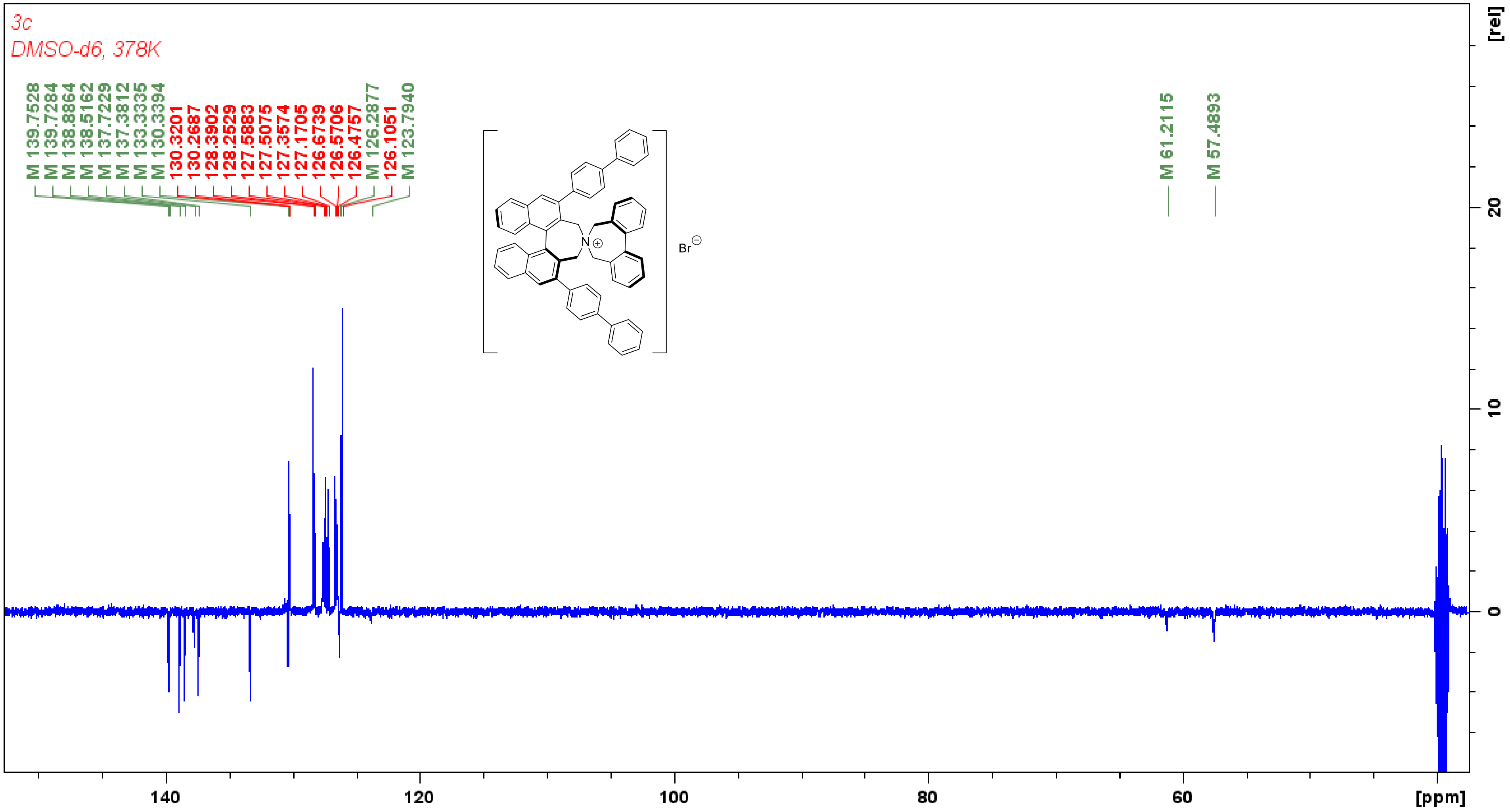
3c
DMSO-d6, 378K
M 139.7528
M 139.7284
M 138.8864
M 138.5162
M 137.7229
M 137.3812
M 133.3335
M 130.3394
130.3201
130.2687
128.3902
128.2529
127.5883
127.5075
127.3574
127.1705
126.6739
126.5706
126.4757
M 126.2877
126.1051
M 123.7940
M 61.2115
M 57.4893
N
Br
[rel]
20
10
0
140
120
100
80
60
[ppm]

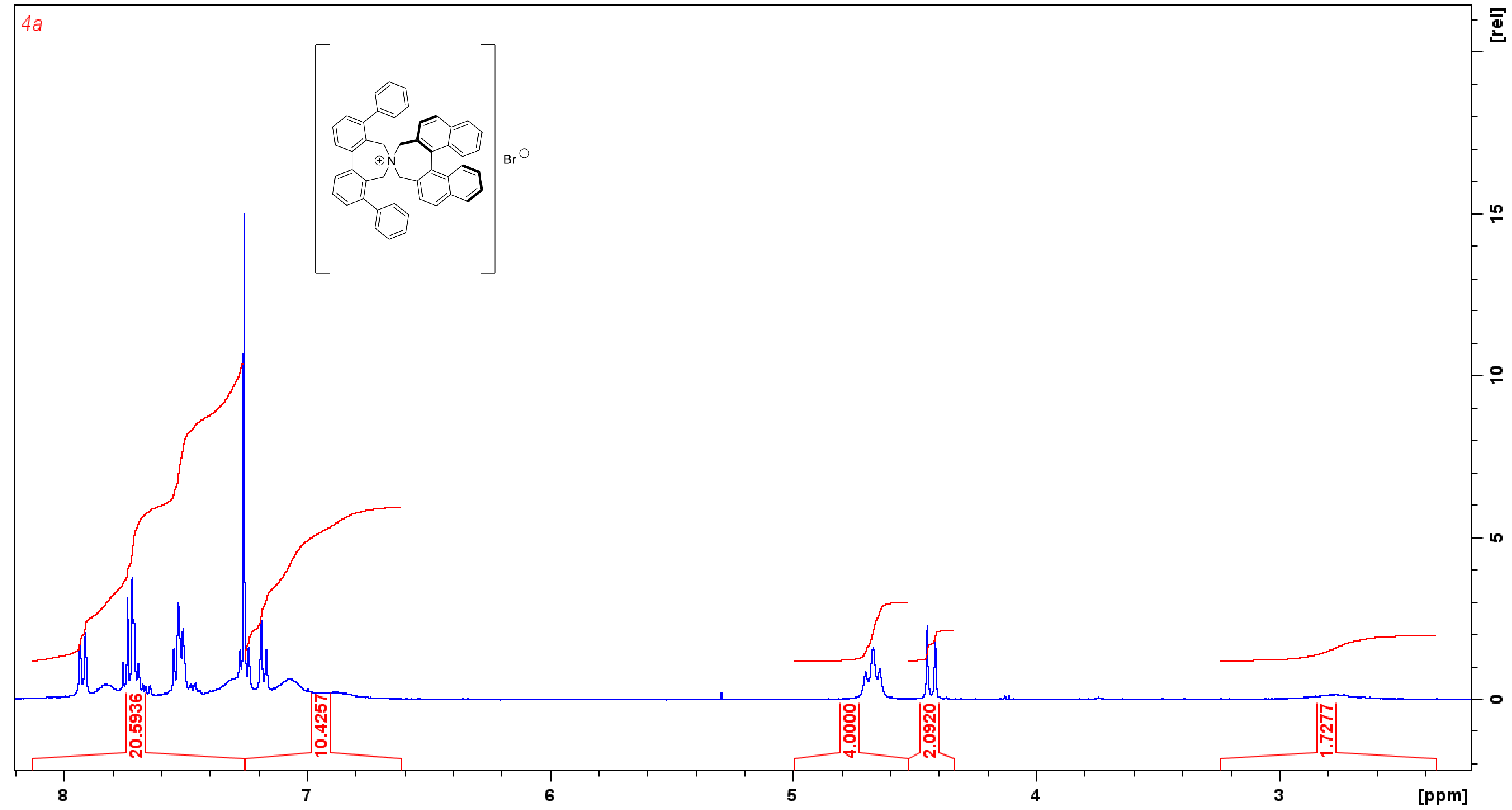
4a
Br
[rel]
15
10
5
0
20.5936
10.4257
4.0000
2.0920
1.7277
8
7
6
5
4
3
[ppm]

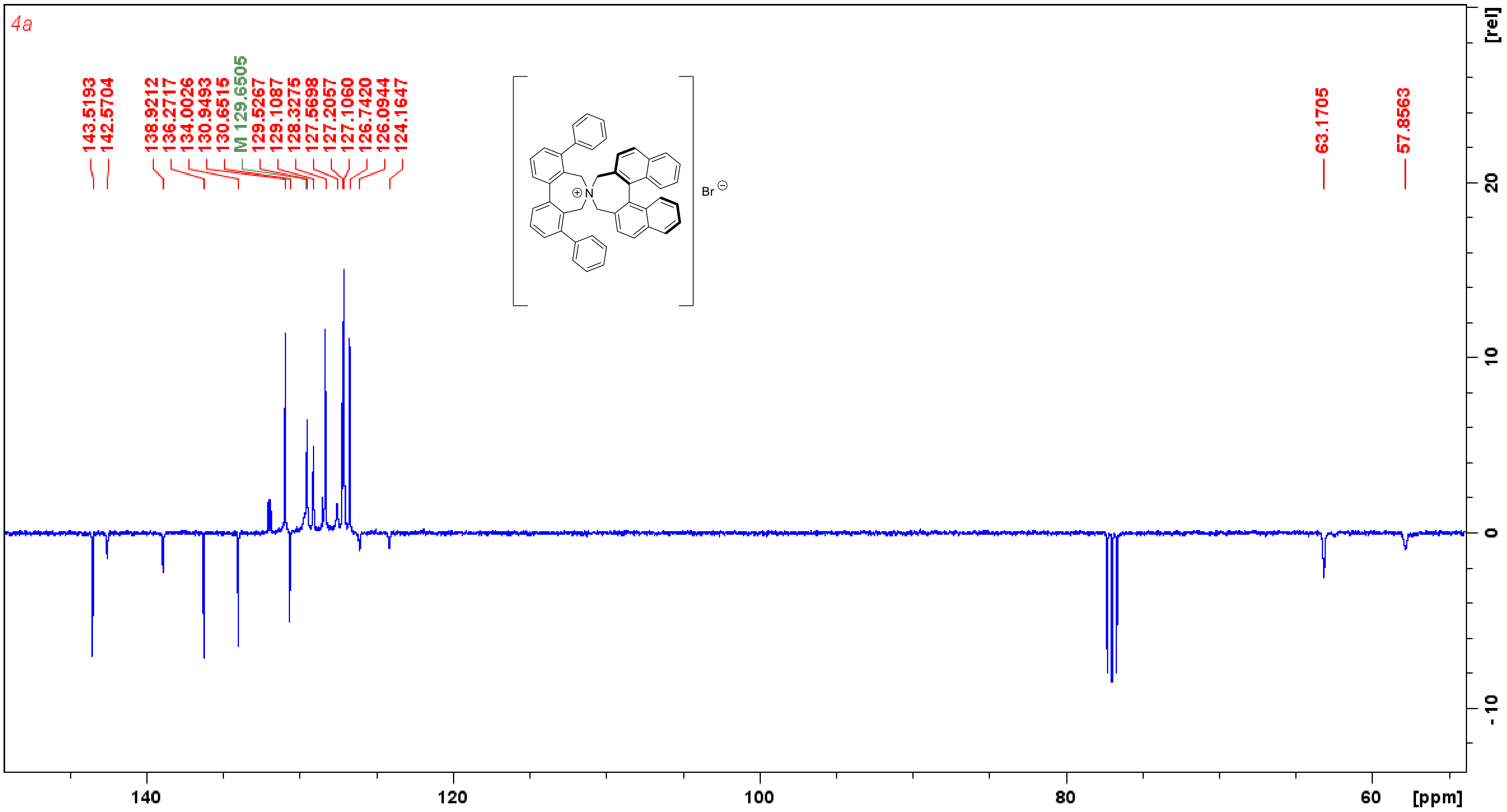
4a
143.5193
142.5704
138.9212
136.2717
134.0026
130.9493
130.6515
M 129.6505
129.5267
129.1087
128.3275
127.5698
127.2057
127.1060
126.7420
126.0944
124.1647
63.1705
57.8563
Br
[rel]
20
10
0
-10
140
120
100
80
60
[ppm]

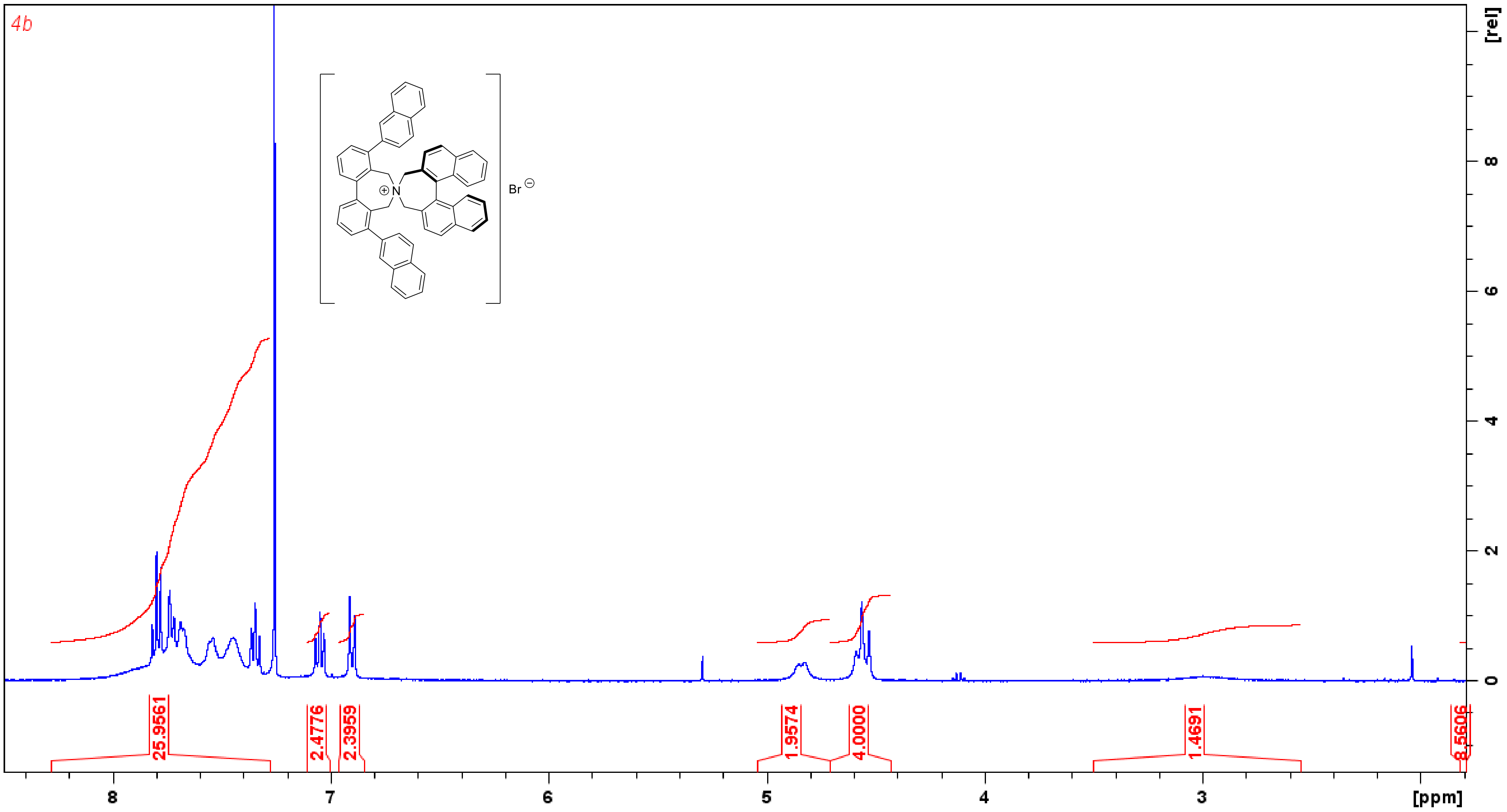
4b
Br
[rel]
25.9561
2.4776
2.3959
1.9574
4.0000
1.4691
8.5606
8
7
6
5
4
3
[ppm]

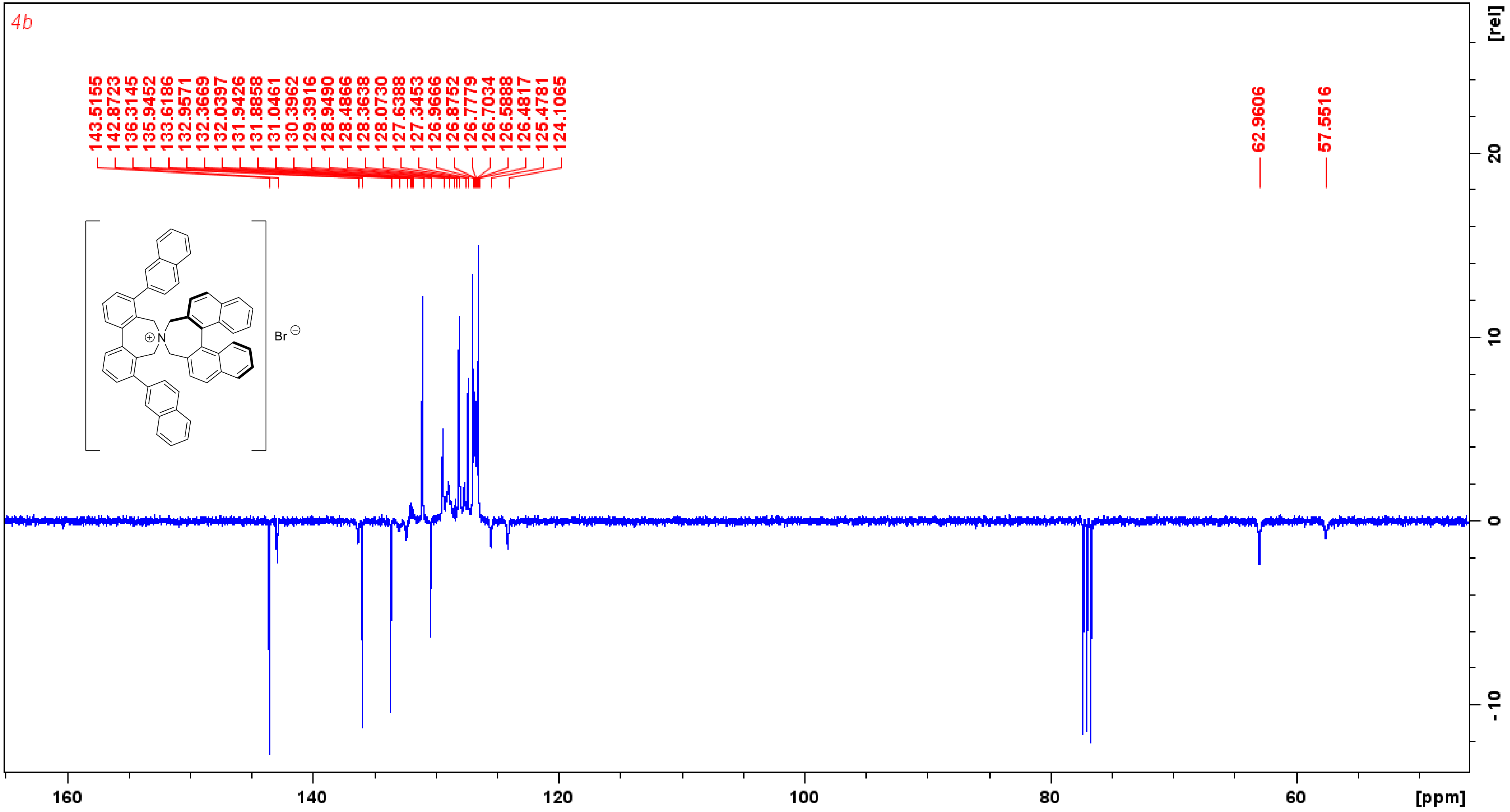
4b
143.5155
142.8723
136.3145
135.9452
133.6186
132.9571
132.3669
132.0397
131.9426
131.8858
131.0461
130.3962
129.3916
128.9490
128.4866
128.3638
128.0730
127.6388
127.3453
126.9666
126.8752
126.7779
126.7034
126.5888
126.4817
125.4781
124.1065
62.9606
57.5516
Br
N
[rel]
20
10
0
-10
160
140
120
100
80
60
[ppm]

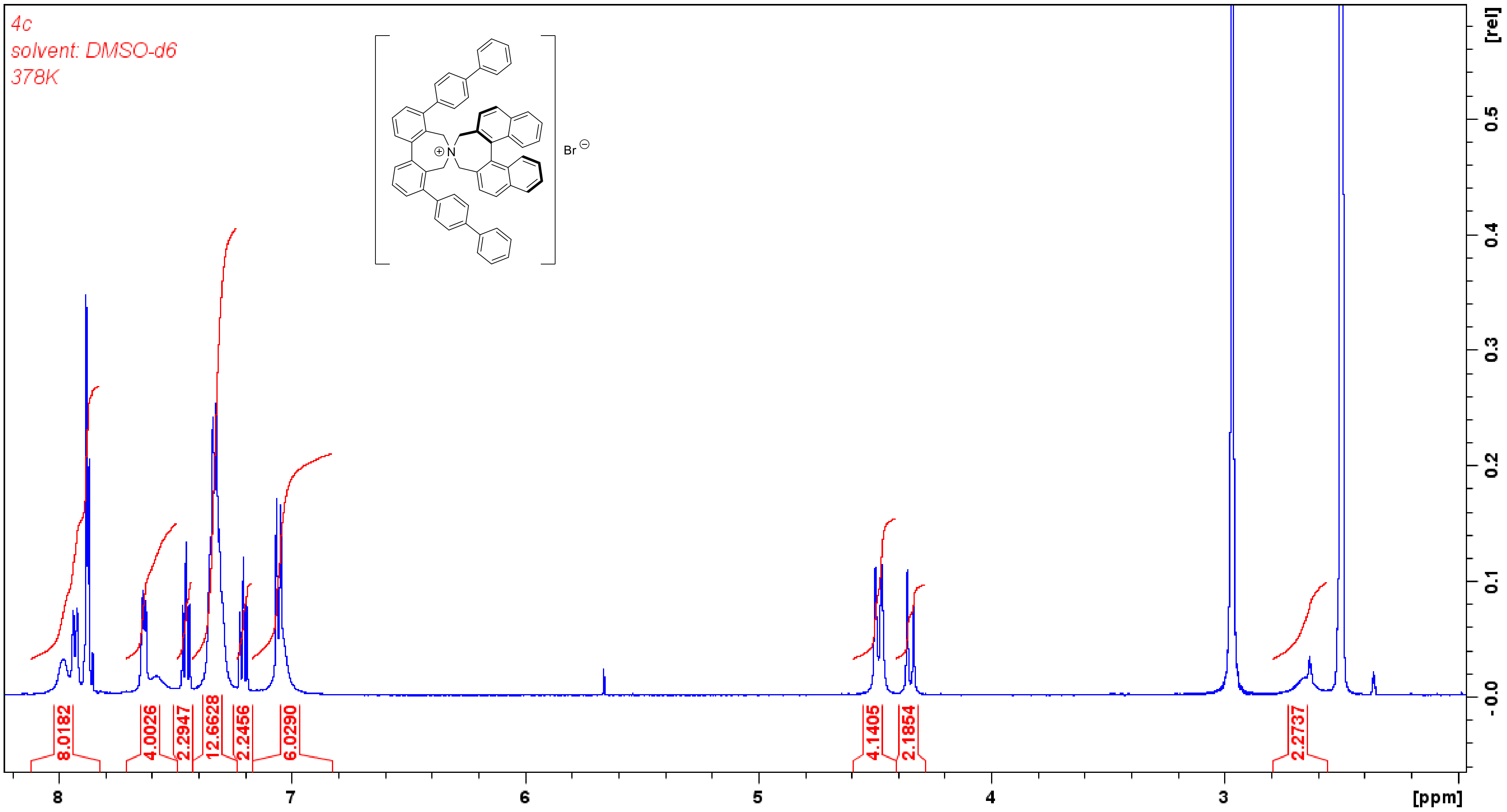

4c
solvent: DMSO-d6
378K
Br
N
8.0182
4.0026
2.2947
12.6628
2.2456
6.0290
4.1405
2.1854
2.2737
8
7
6
5
4
3
[ppm]
[rel]
0.5
0.4
0.3
0.2
0.1
-0.0

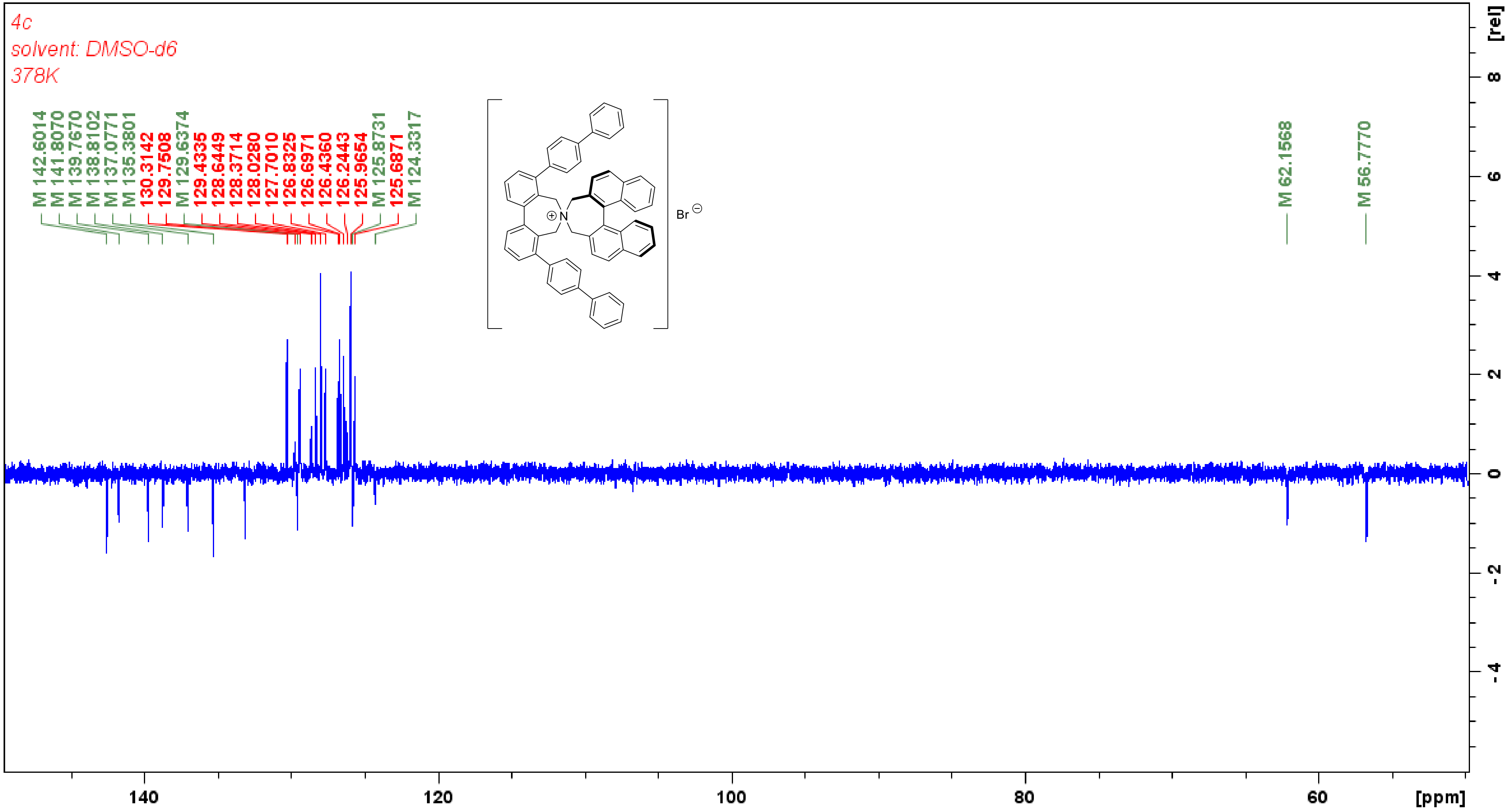
4c
solvent: DMSO-d6
378K
M 142.6014
M 141.8070
M 139.7670
M 138.8102
M 137.0771
M 135.3801
130.3142
129.7508
M 129.6374
129.4335
128.6449
128.3714
128.0280
127.7010
126.8325
126.6971
126.4360
126.2443
125.9654
M 125.8731
125.6871
M 124.3317
M 62.1568
M 56.7770
Br
[rel]
[ppm]

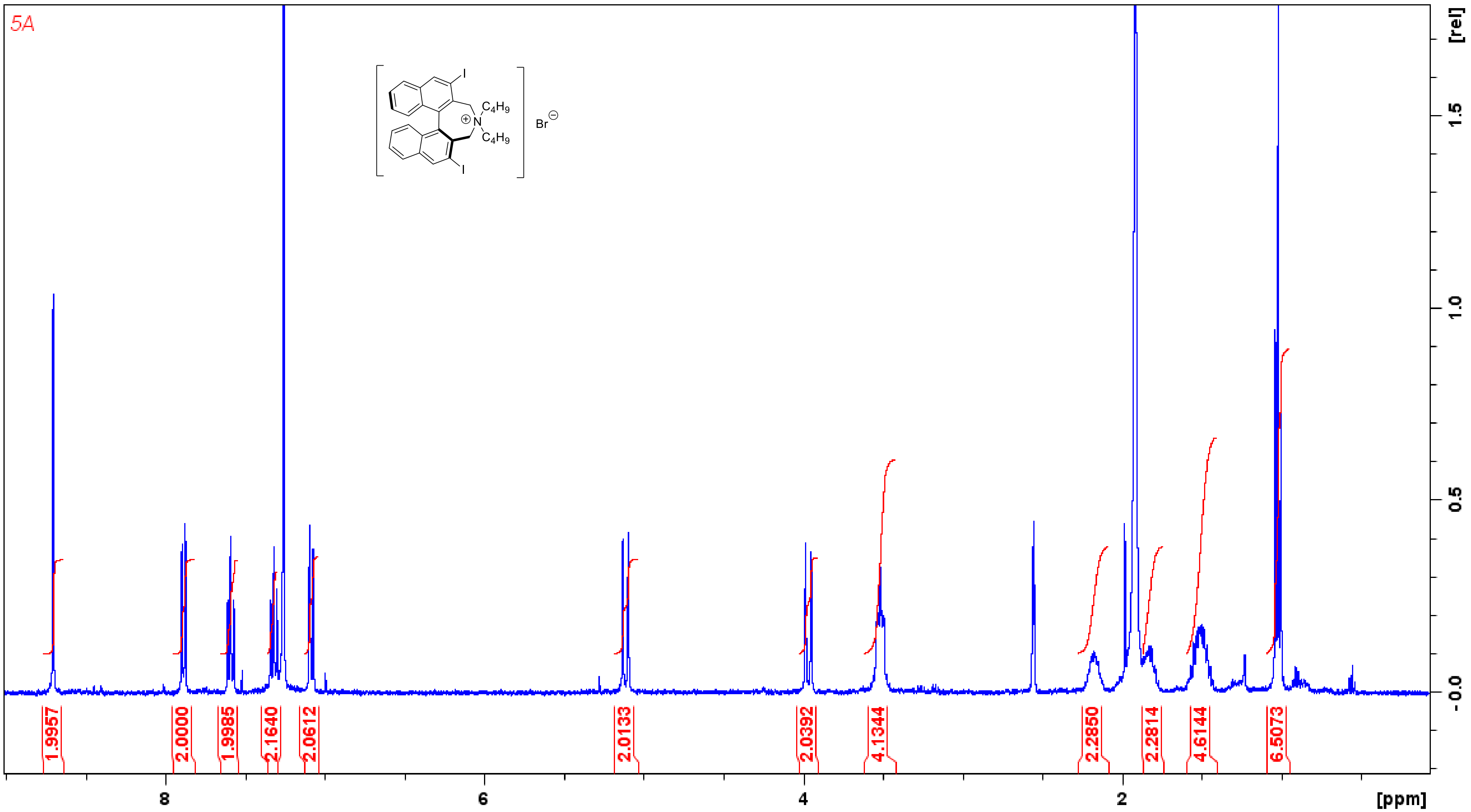
5A
I
C4H9
N
C4H9
I
Br
1.9957
2.0000
1.9985
2.1640
2.0612
2.0133
2.0392
4.1344
2.2850
2.2814
4.6144
6.5073
8
6
4
2
[ppm]
[rel]
1.5
1.0
0.5
-0.0

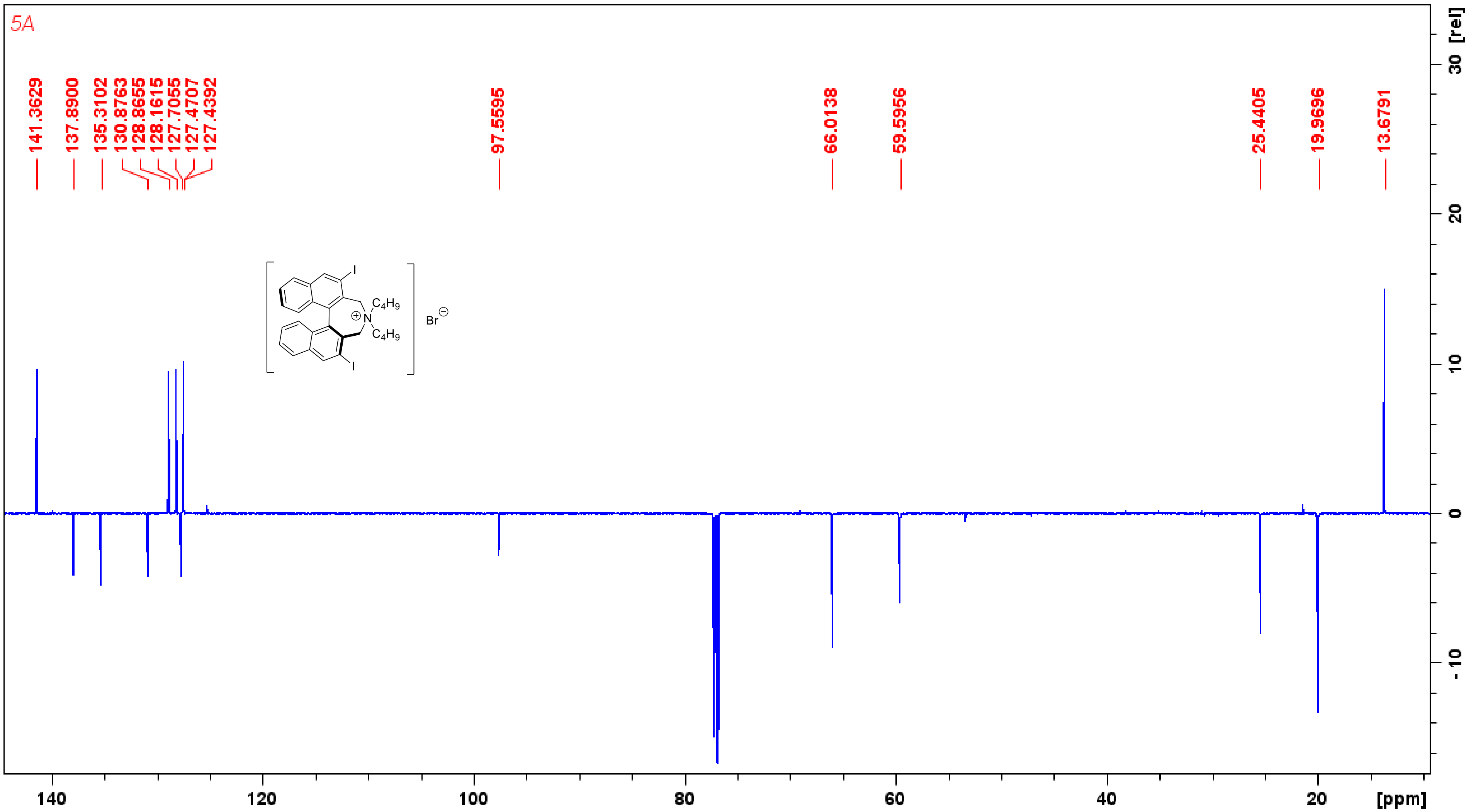
5A
141.3629
137.8900
135.3102
130.8763
128.8655
128.1615
127.7055
127.4707
127.4392
97.5595
66.0138
59.5956
25.4405
19.9696
13.6791
I
C4H9
N
C4H9
I
Br
[rel]
[ppm]

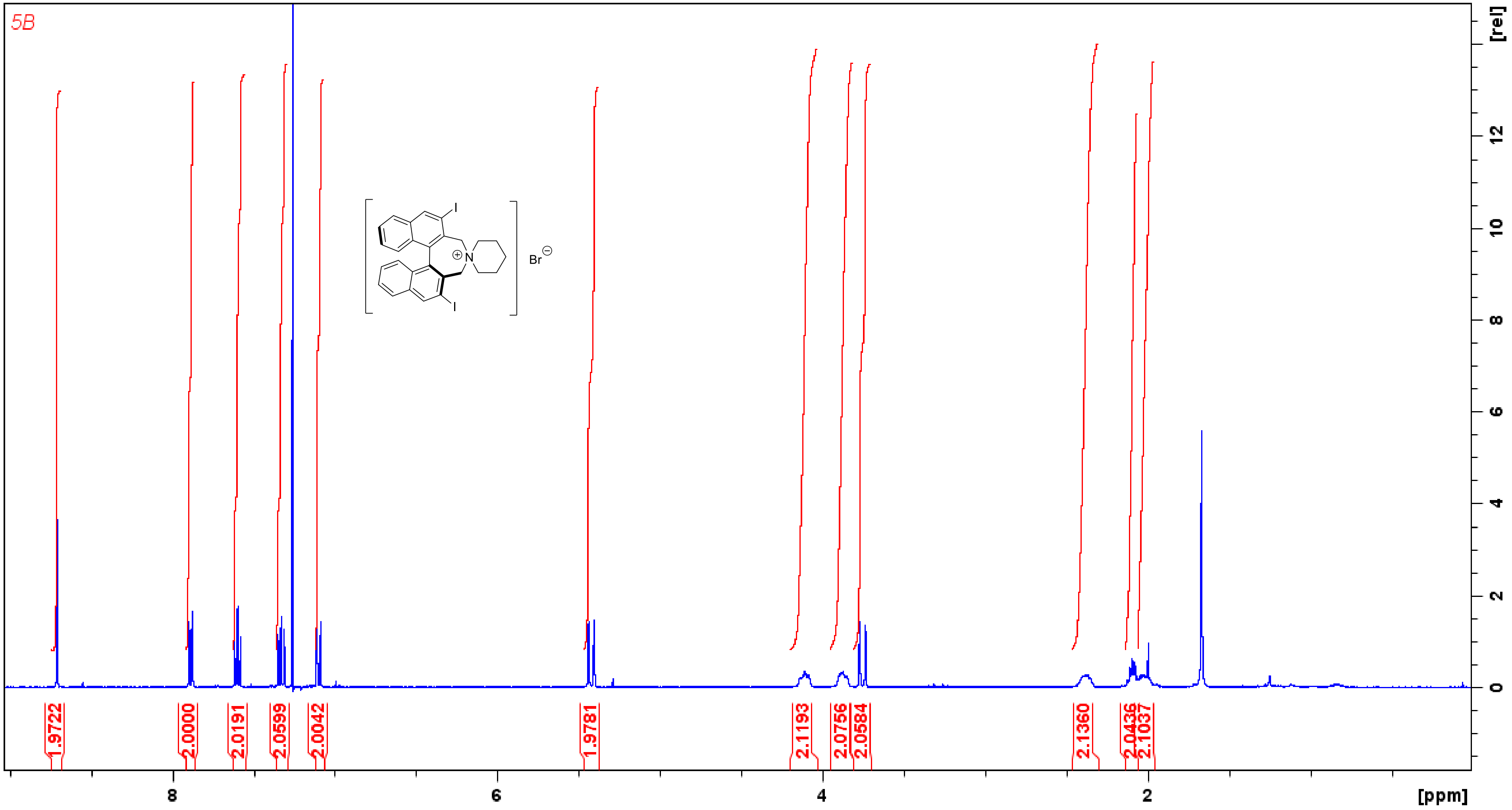
5B
[rel]
0
2
4
6
8
10
12
Br
I
I
N
1.9722
2.0000
2.0191
2.0599
2.0042
1.9781
2.1193
2.0756
2.0584
2.1360
2.0436
2.1037
8
6
4
2
[ppm]

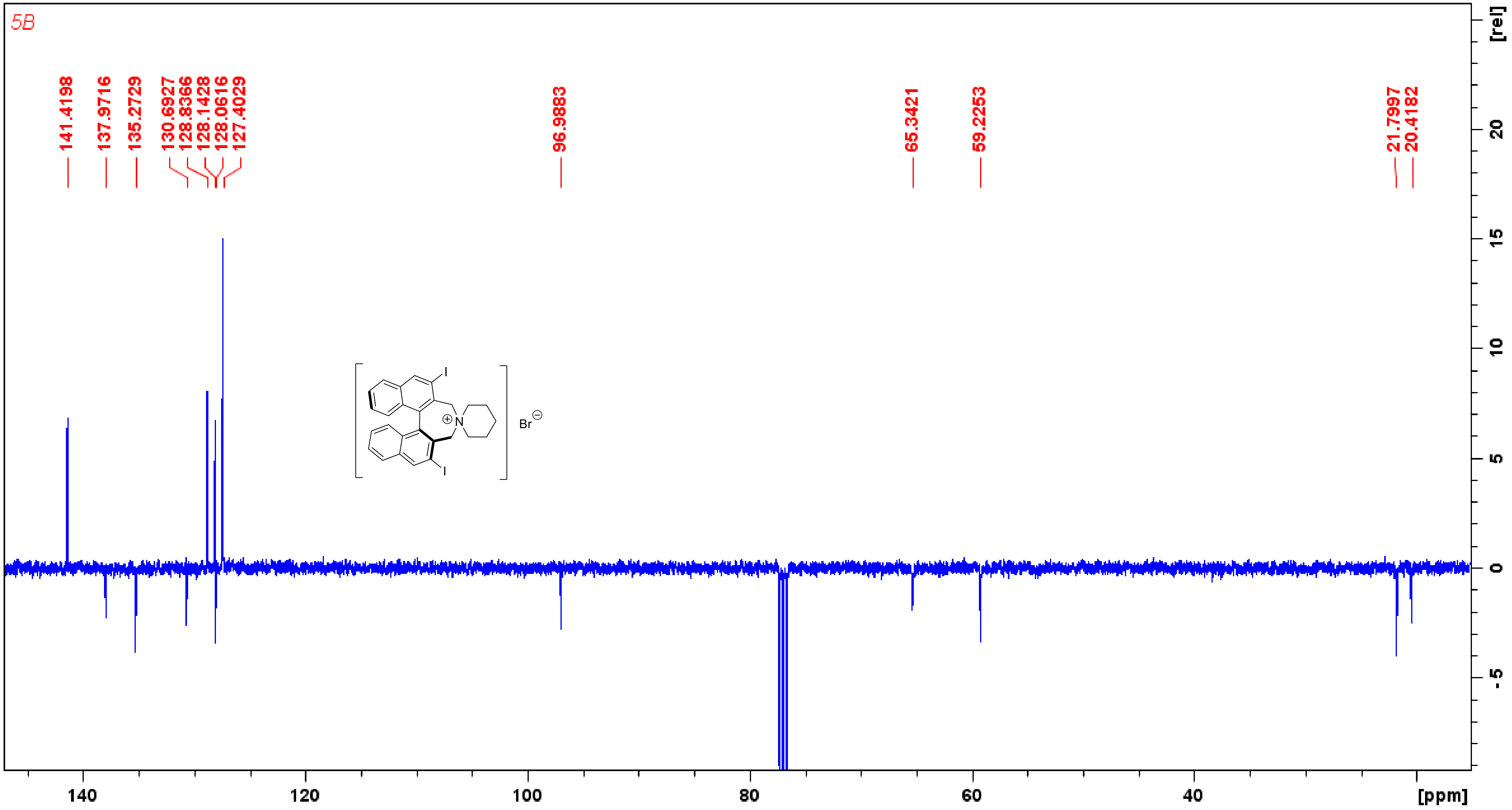
5B
141.4198
137.9716
135.2729
130.6927
128.8366
128.1428
128.0616
127.4029
96.9883
65.3421
59.2253
21.7997
20.4182
I
N
Br
I
140
120
100
80
60
40
[ppm]
[rel]
20
15
10
5
0
-5

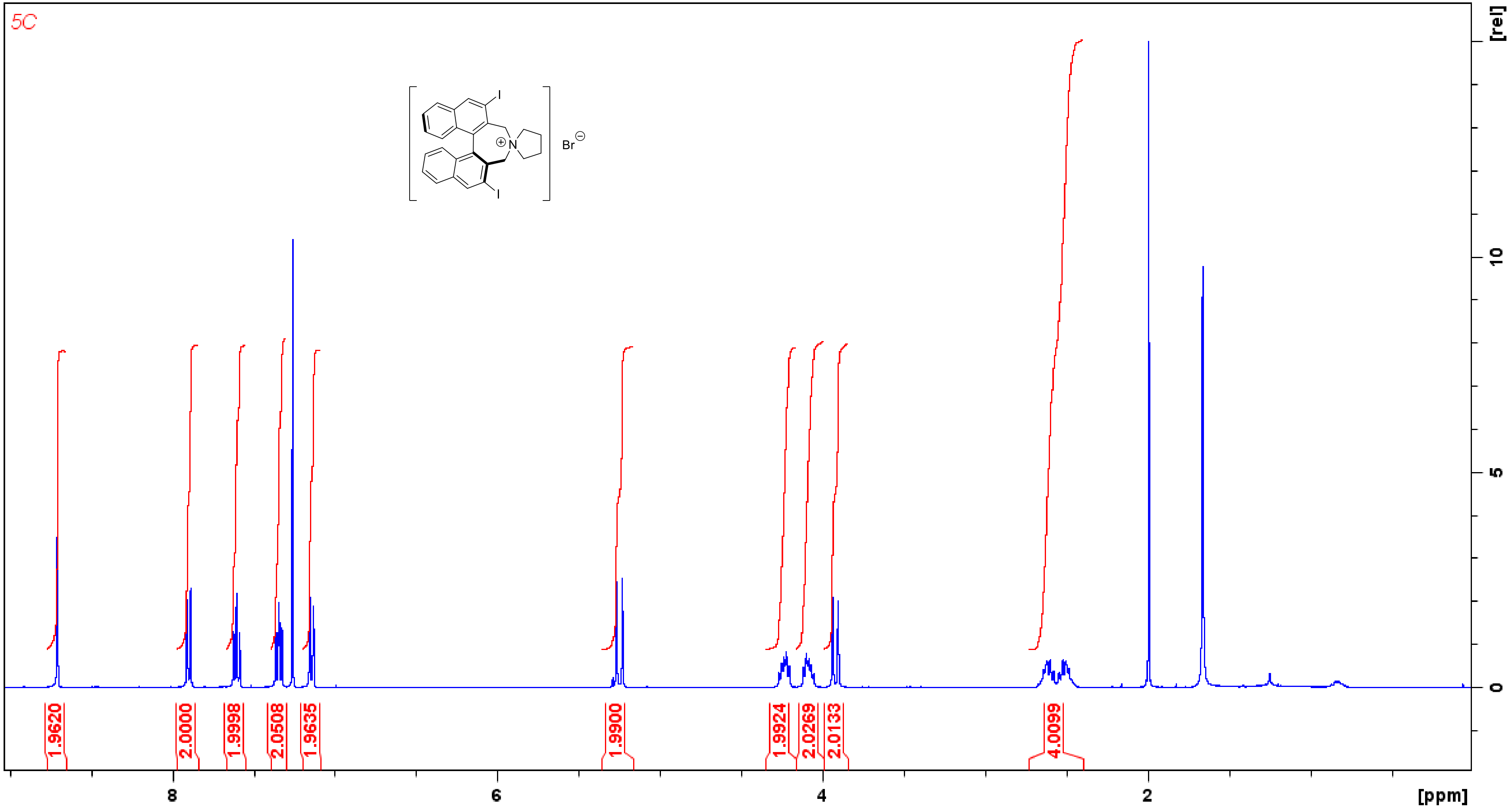
5C
I
N
Br
I
[rel]
10
5
0
1.9620
2.0000
1.9998
2.0508
1.9635
1.9900
1.9924
2.0269
2.0133
4.0099
8
6
4
2
[ppm]

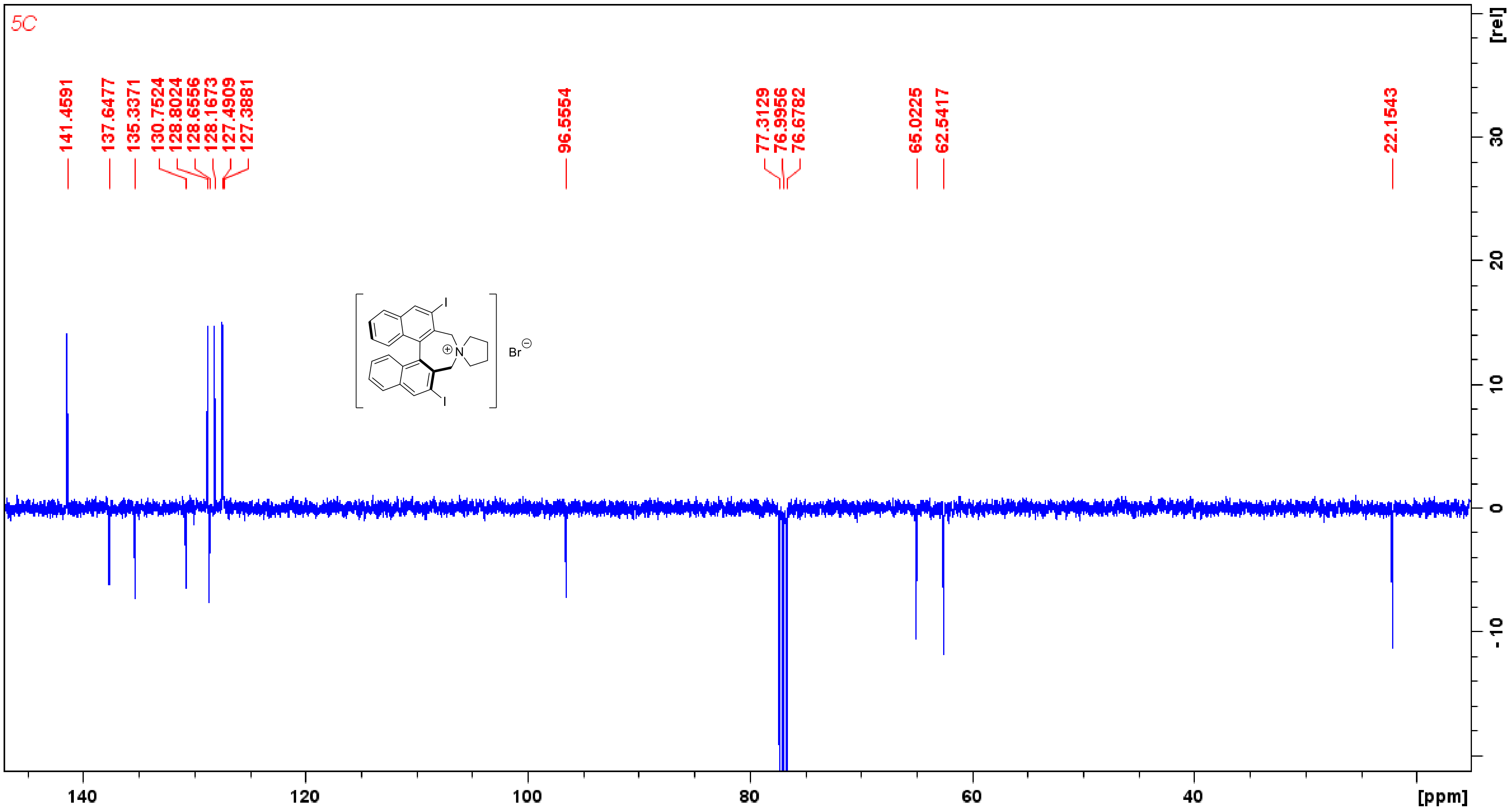

5C
141.4591
137.6477
135.3371
130.7524
128.8024
128.6556
128.1673
127.4909
127.3881
96.5554
77.3129
76.9956
76.6782
65.0225
62.5417
22.1543
I
N
Br
140
120
100
80
60
40
[ppm]
[rel]
30
20
10
0
-10

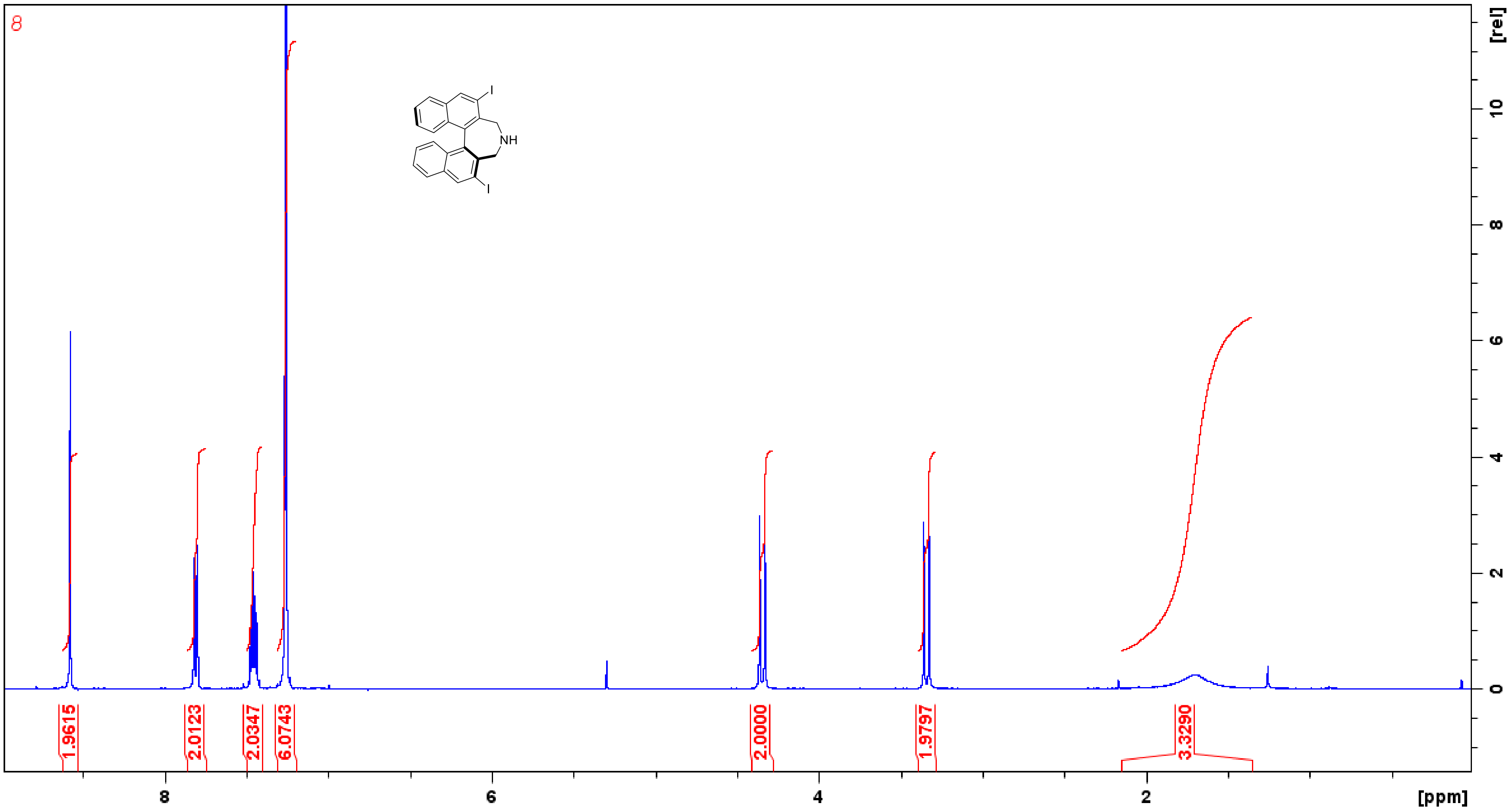
8
I
NH
I
[rel]
10
8
6
4
2
0
1.9615
2.0123
2.0347
6.0743
2.0000
1.9797
3.3290
8
6
4
2
[ppm]

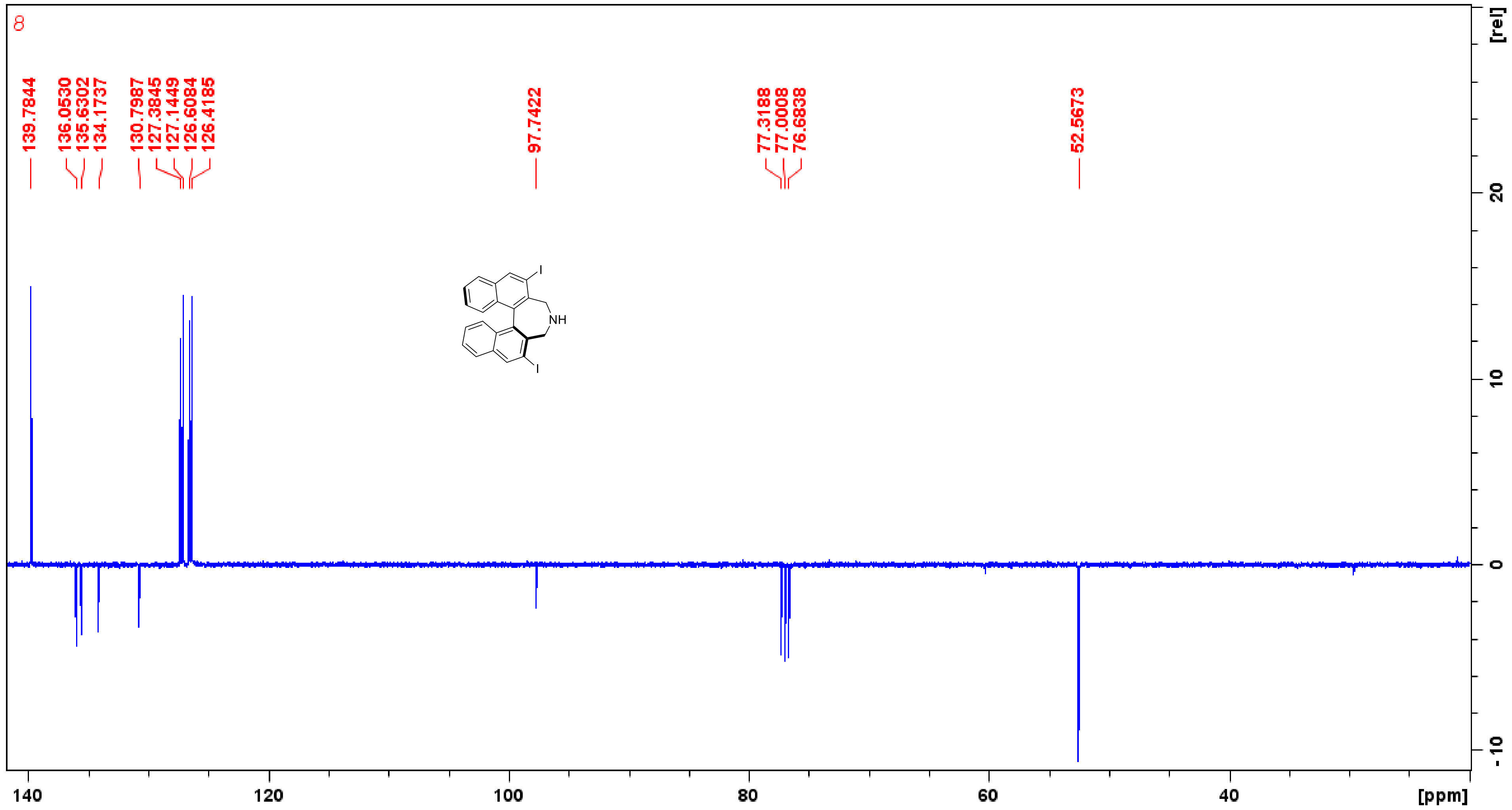
8
139.7844
136.0530
135.6302
134.1737
130.7987
127.3845
127.1449
126.6084
126.4185
97.7422
77.3188
77.0008
76.6838
52.5673
NH
I
I
[rel]
20
10
0
-10
140
120
100
80
60
40
[ppm]

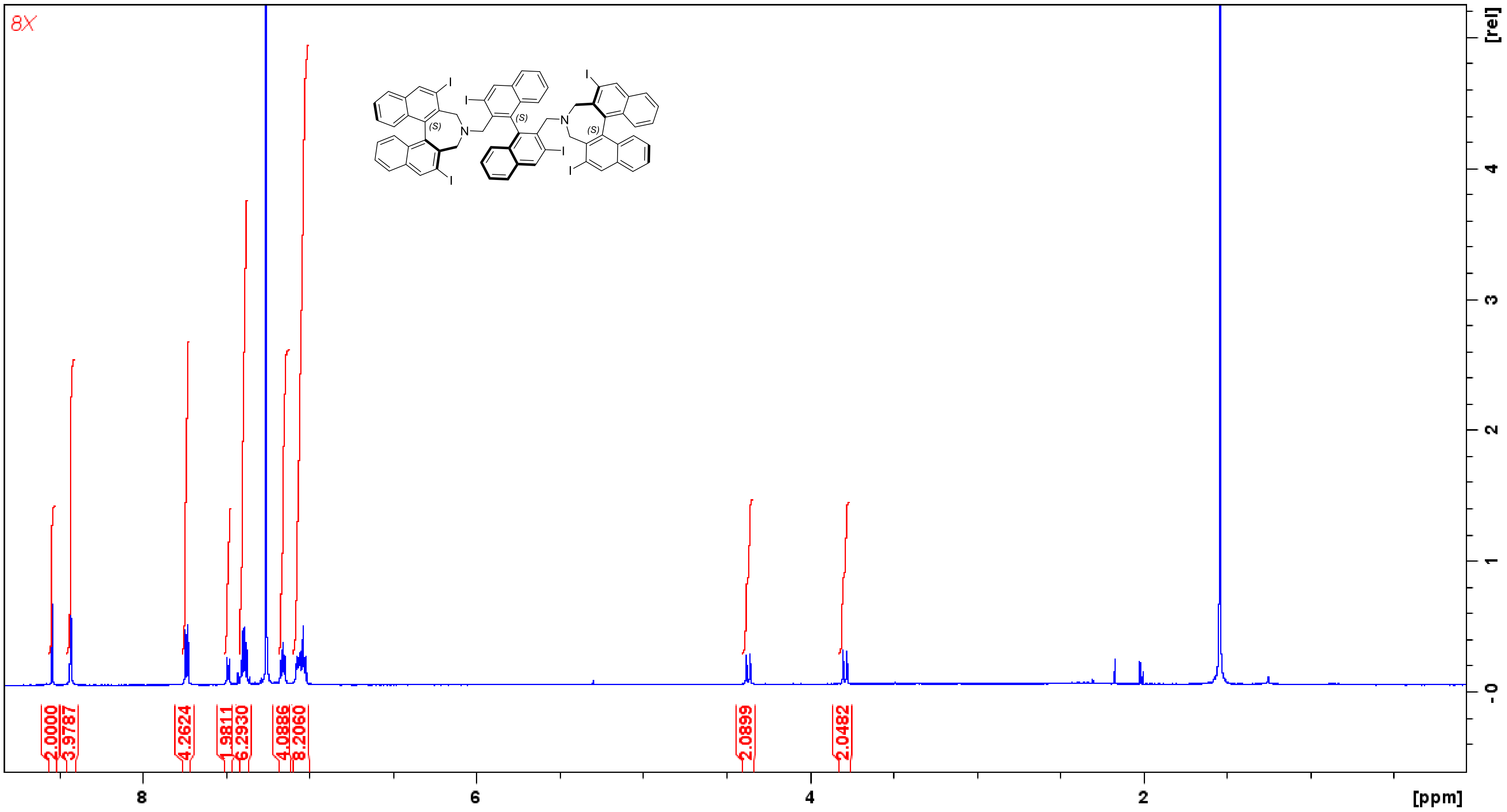
8X
2.0000
3.9787
4.2624
1.9811
6.2930
4.0886
8.2060
2.0899
2.0482
8
6
4
2
[ppm]
[rel]
(S)
N
I

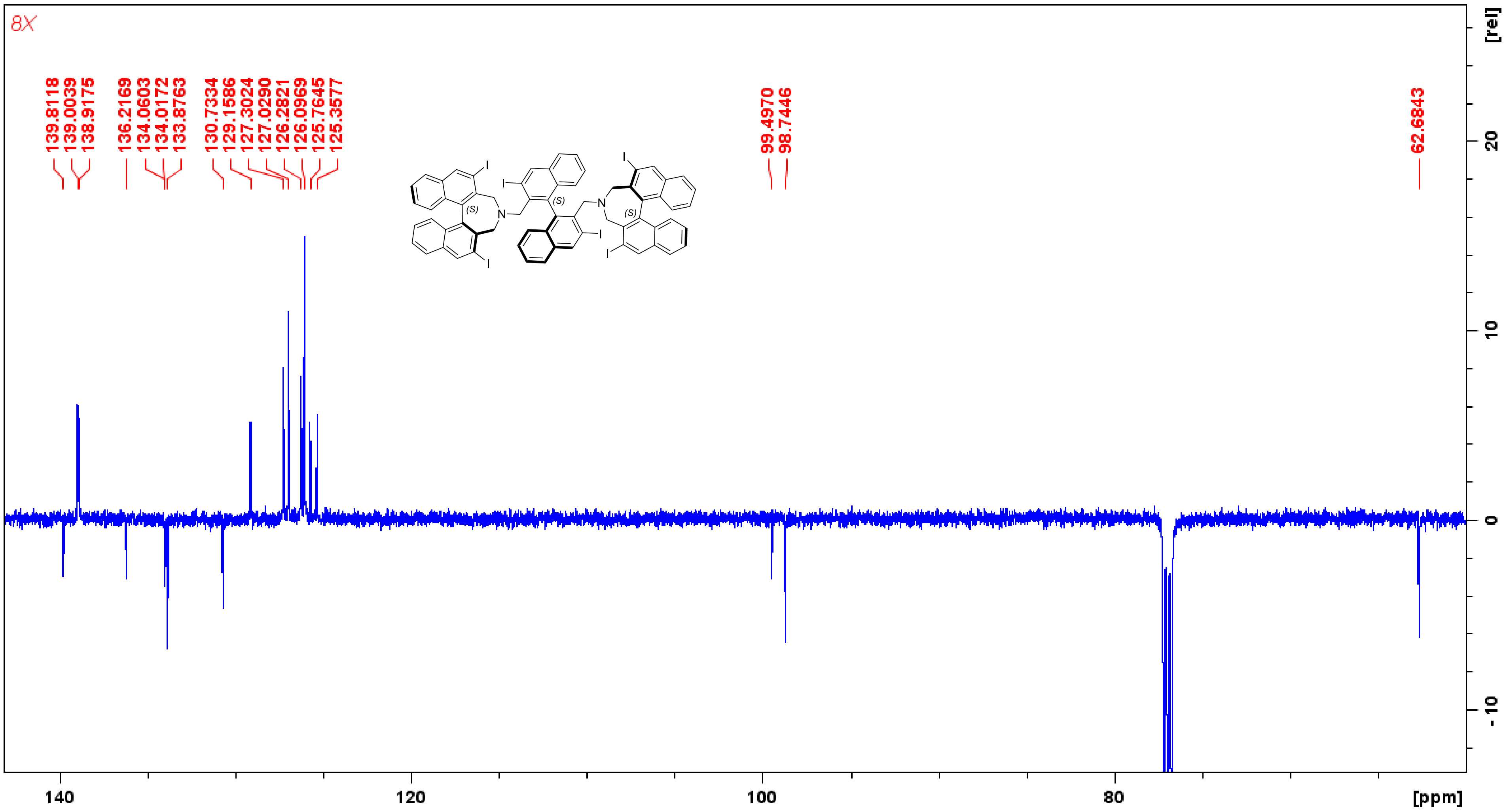
8X
139.8118
139.0039
138.9175
136.2169
134.0603
134.0172
133.8763
130.7334
129.1586
127.3024
127.0290
126.2821
126.0969
125.7645
125.3577
99.4970
98.7446
62.6843
[rel]
20
10
0
-10
140
120
100
80
[ppm]

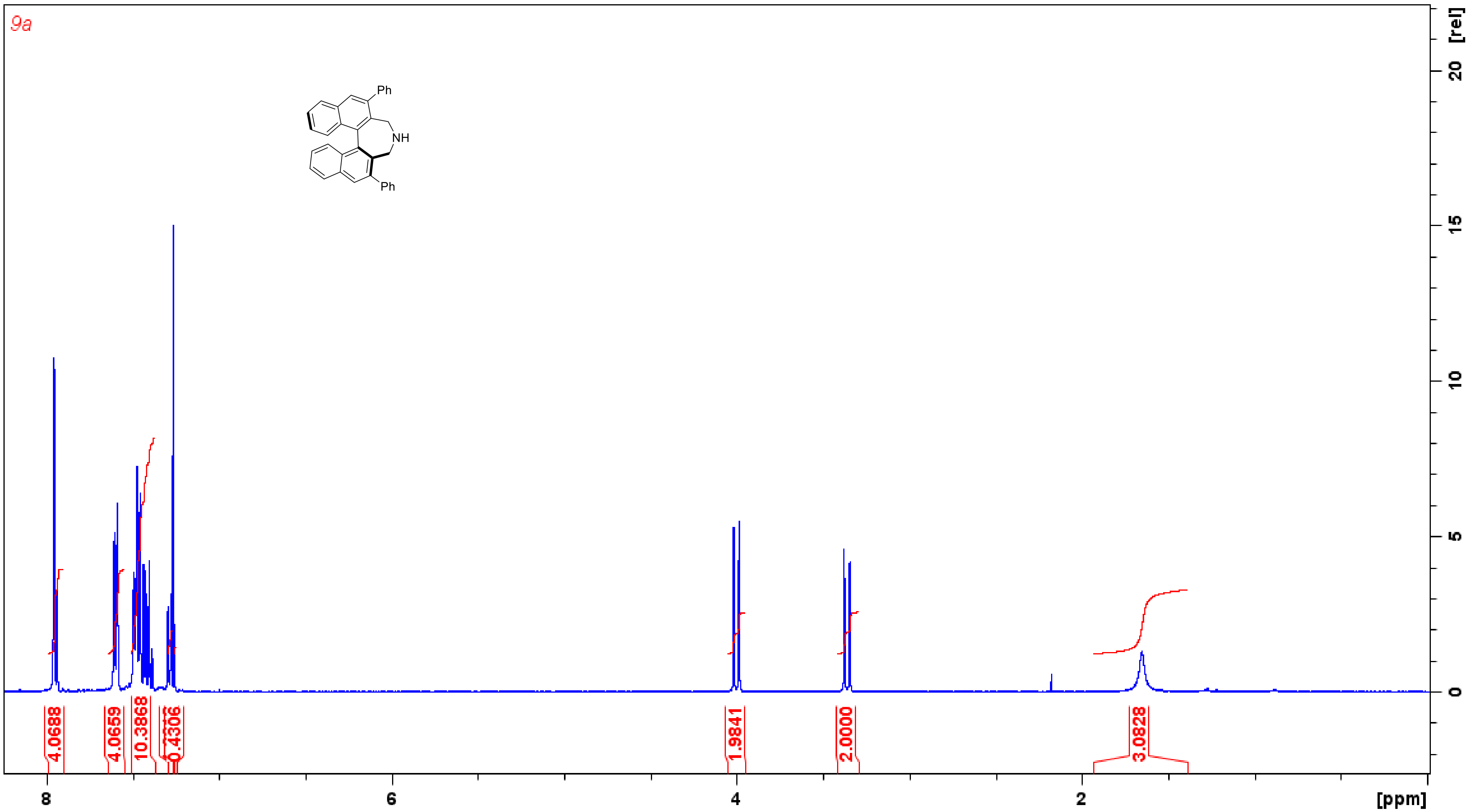
9a
Ph
NH
Ph
4.0688
4.0659
10.3868
0.4306
1.9841
2.0000
3.0828
8
6
4
2
[ppm]
0
5
10
15
20
[rel]

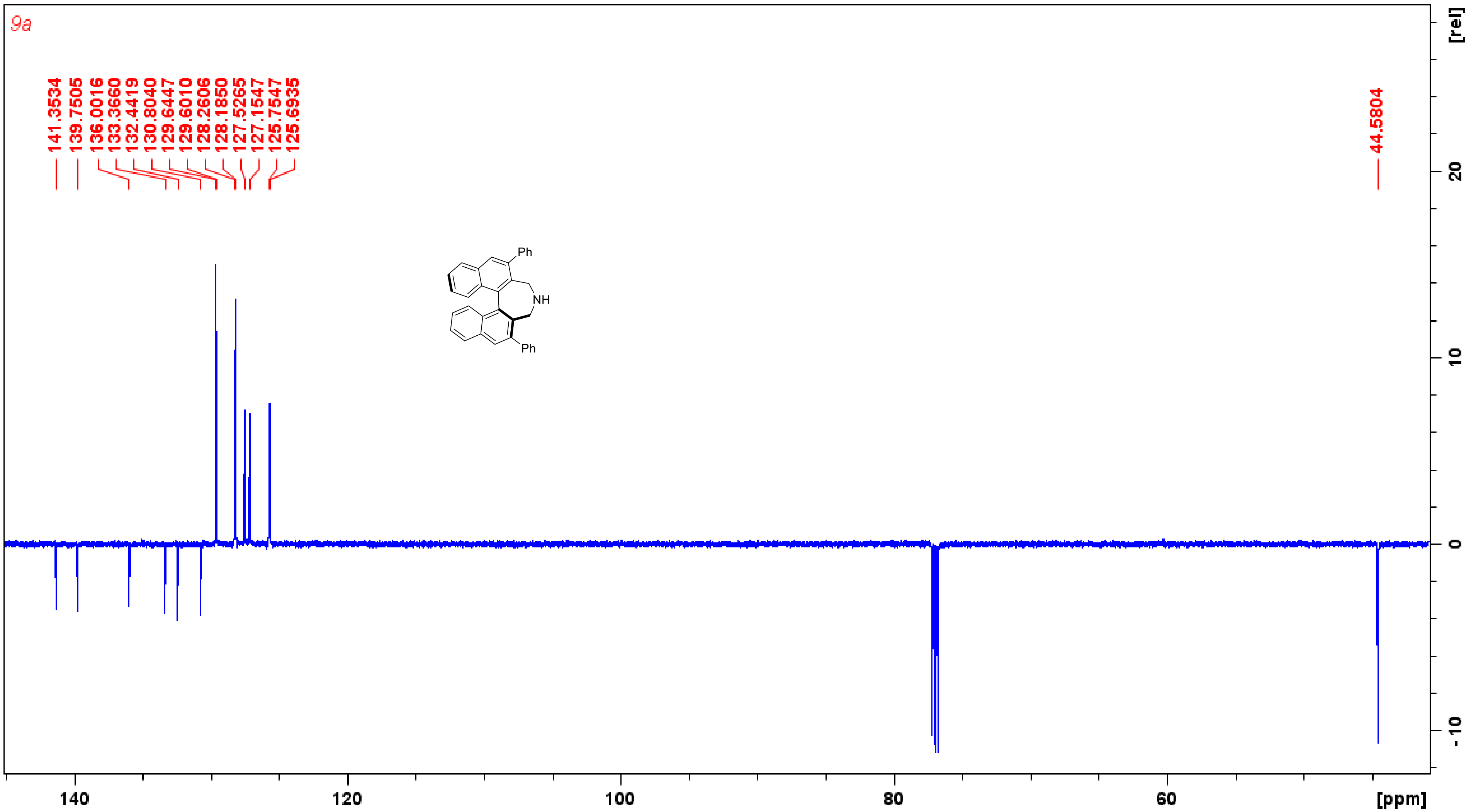
9a
141.3534
139.7505
136.0016
133.3660
132.4419
130.8040
129.6447
129.6010
128.2606
128.1850
127.5265
127.1547
125.7547
125.6935
44.5804
Ph
NH
Ph
[rel]
20
10
0
-10
140
120
100
80
60
[ppm]

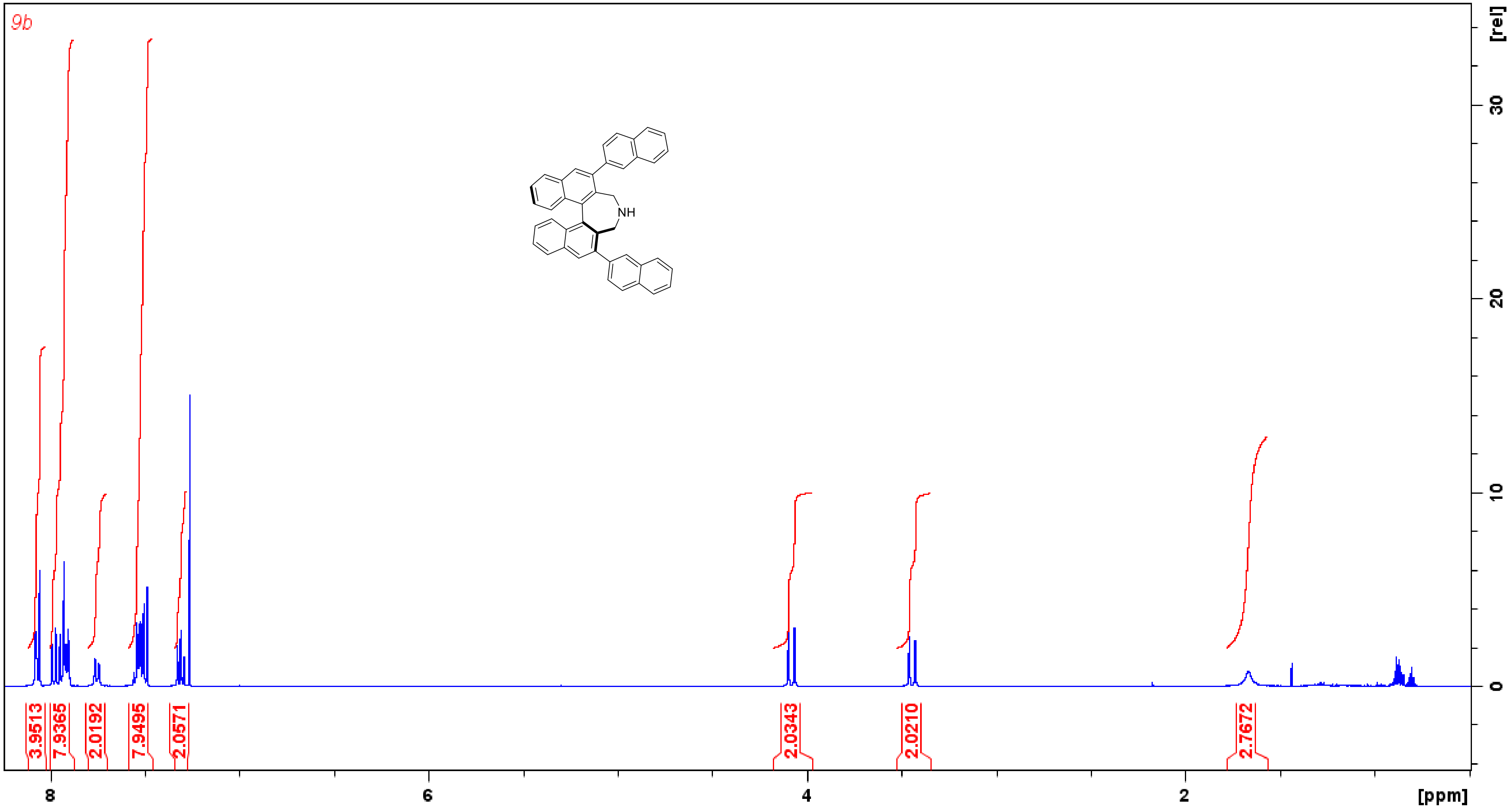
9b
NH
3.9513
7.9365
2.0192
7.9495
2.0571
2.0343
2.0210
2.7672
8
6
4
2
[ppm]
[rel]
30
20
10
0

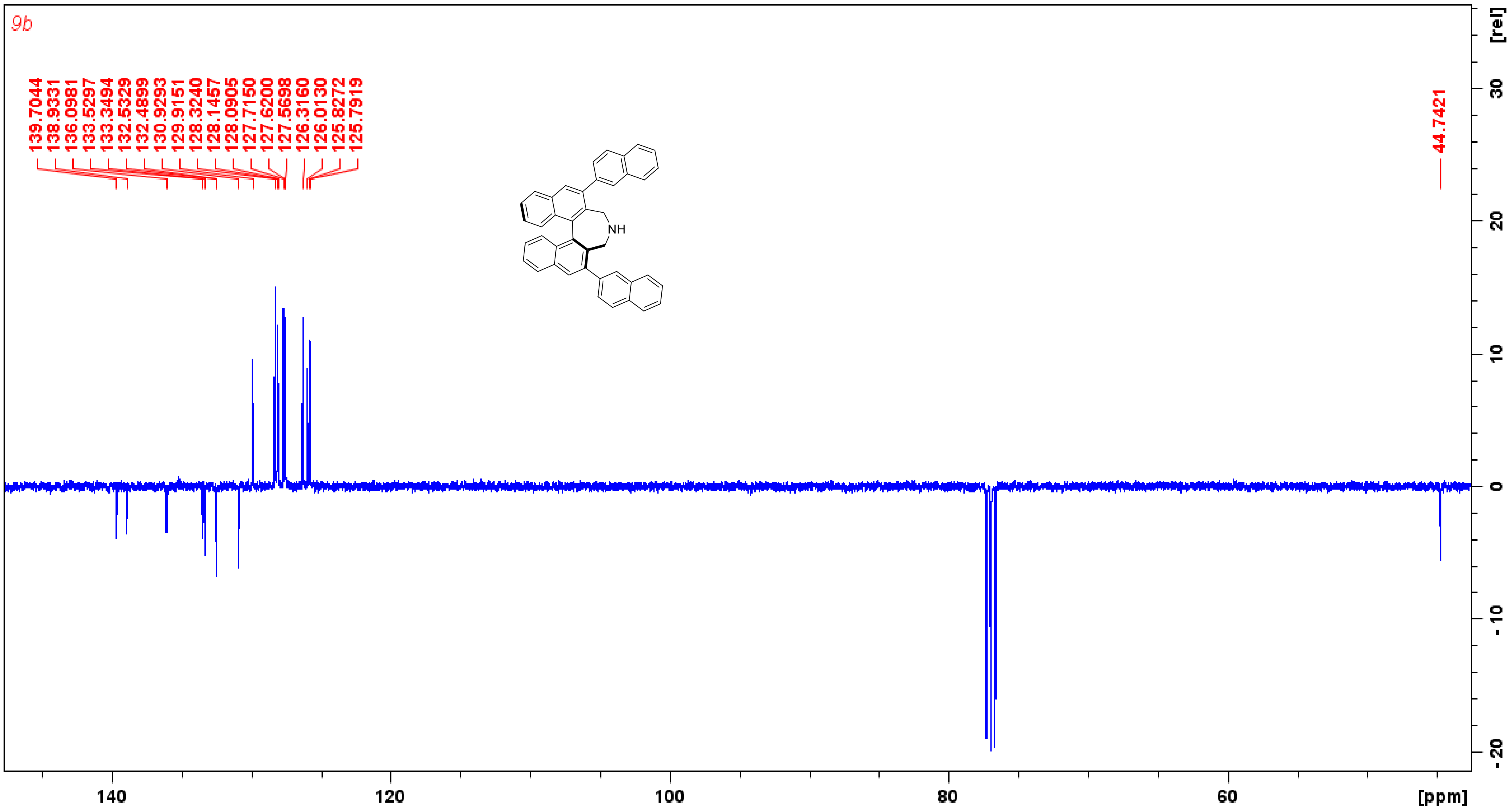
9b
139.7044
138.9331
136.0981
133.5297
133.3494
132.5329
132.4899
130.9293
129.9151
128.3240
128.1457
128.0905
127.7150
127.6200
127.5698
126.3160
126.0130
125.8272
125.7919
44.7421
NH
[rel]
30
20
10
0
-10
-20
140
120
100
80
60
[ppm]

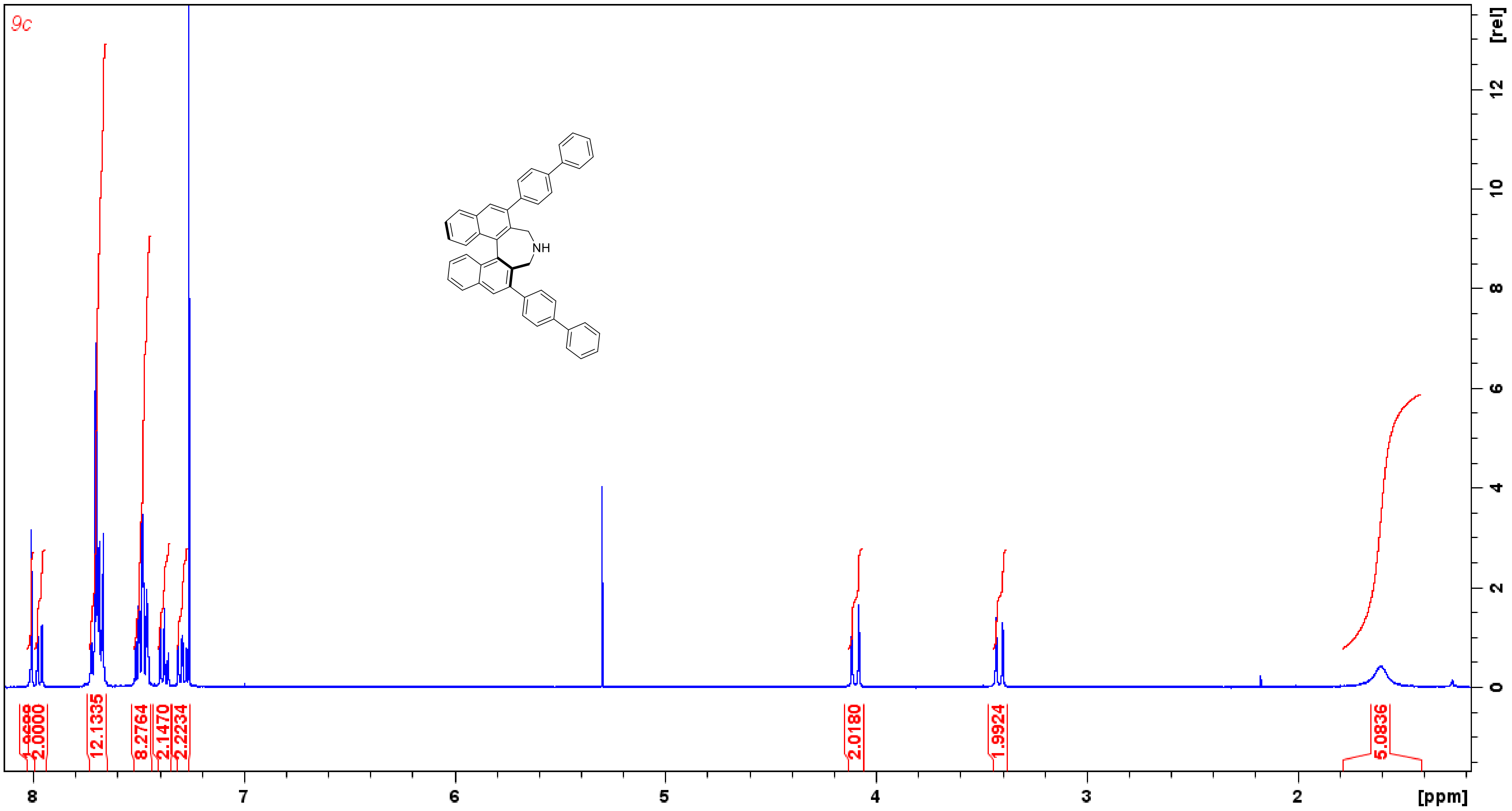
9c
NH
2.0000
12.1335
8.2764
2.1470
2.2234
2.0180
1.9924
5.0836
8
7
6
5
4
3
2
[ppm]
[rel]
0
2
4
6
8
10
12

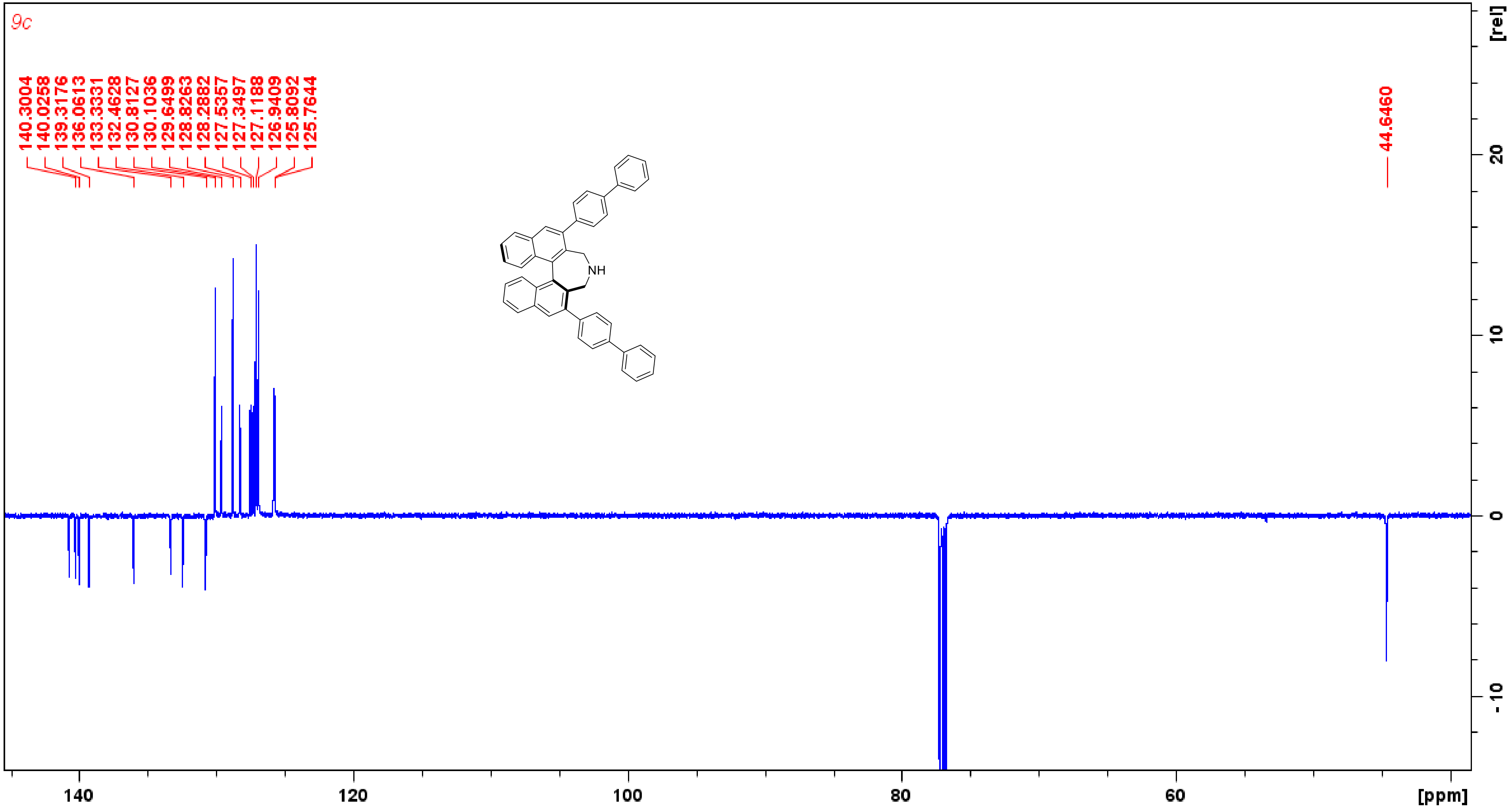
9c
140.3004
140.0258
139.3176
136.0613
133.3331
132.4628
130.8127
130.1036
129.6499
128.8263
128.2882
127.5357
127.3497
127.1188
126.9409
125.8092
125.7644
44.6460
NH
[rel]
20
10
0
-10
140
120
100
80
60
[ppm]

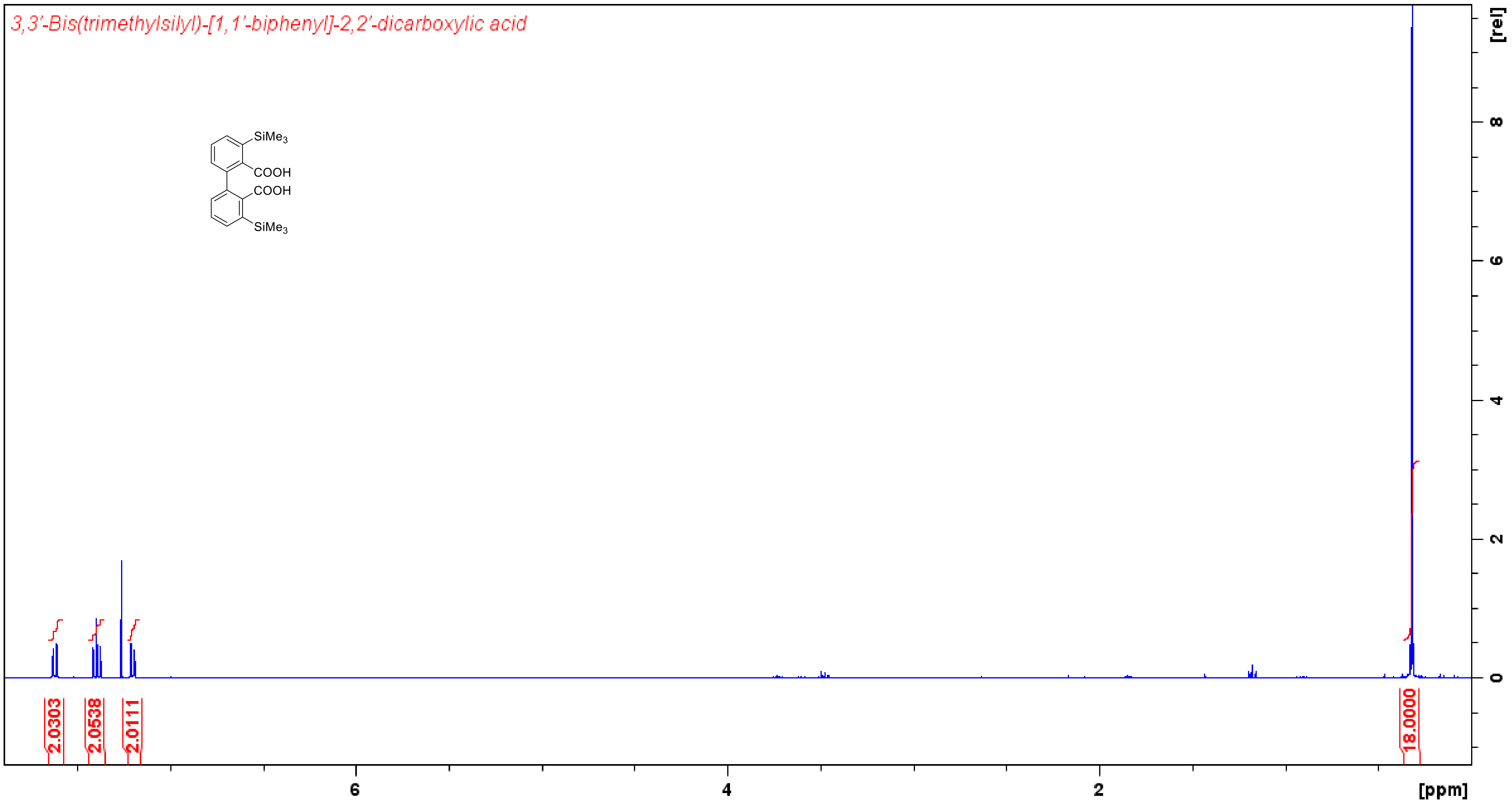
3,3'-Bis(trimethylsilyl)-[1,1'-biphenyl]-2,2'-dicarboxylic acid
SiMe3
COOH
COOH
SiMe3
2.0303
2.0538
2.0111
18.0000
[rel]
8
6
4
2
0
6
4
2
[ppm]

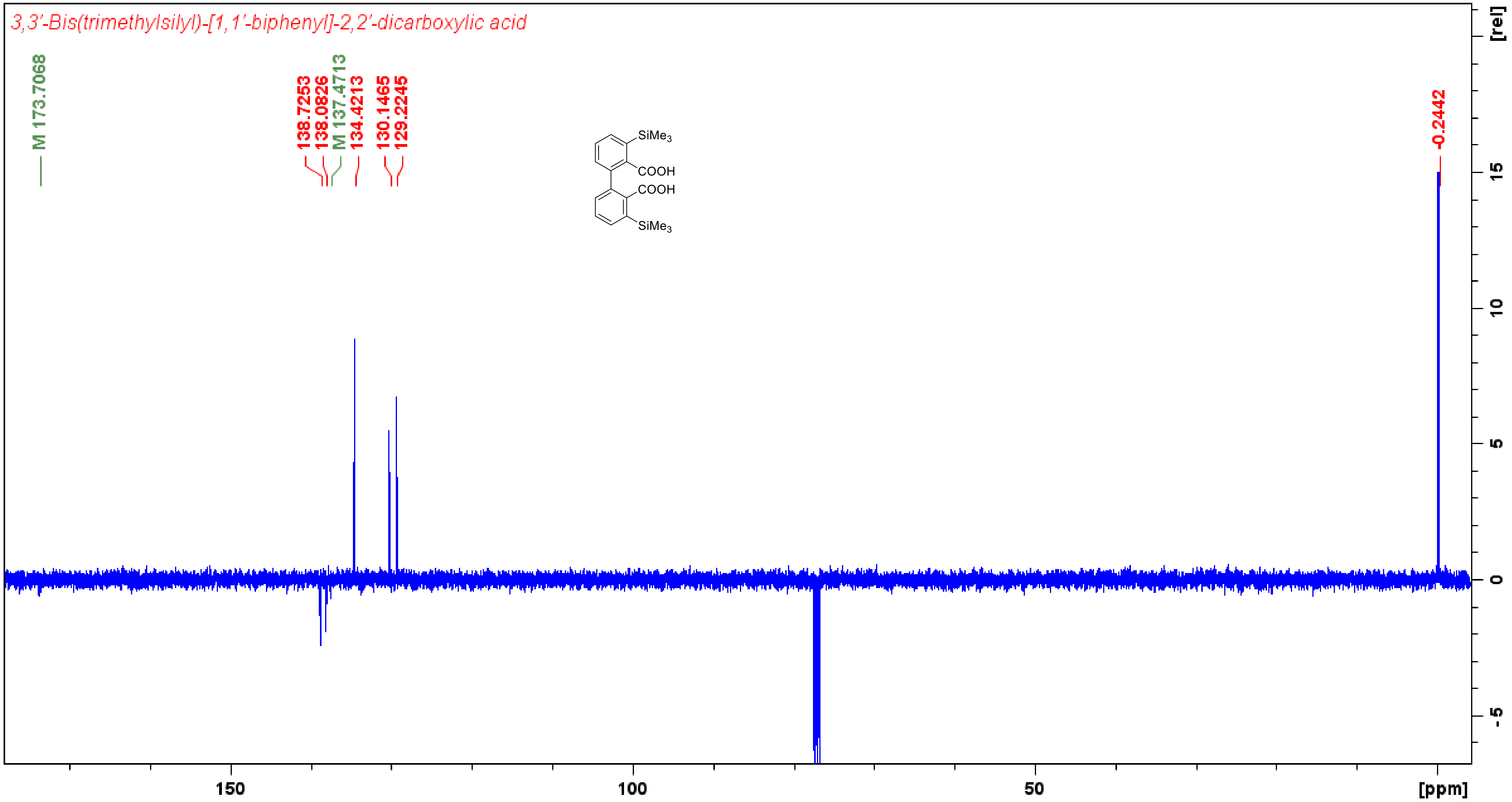
3,3'-Bis(trimethylsilyl)-[1,1'-biphenyl]-2,2'-dicarboxylic acid
M 173.7068
138.7253
138.0826
M 137.4713
134.4213
130.1465
129.2245
-0.2442
SiMe3
COOH
COOH
SiMe3
[rel]
15
10
5
0
-5
150
100
50
[ppm]

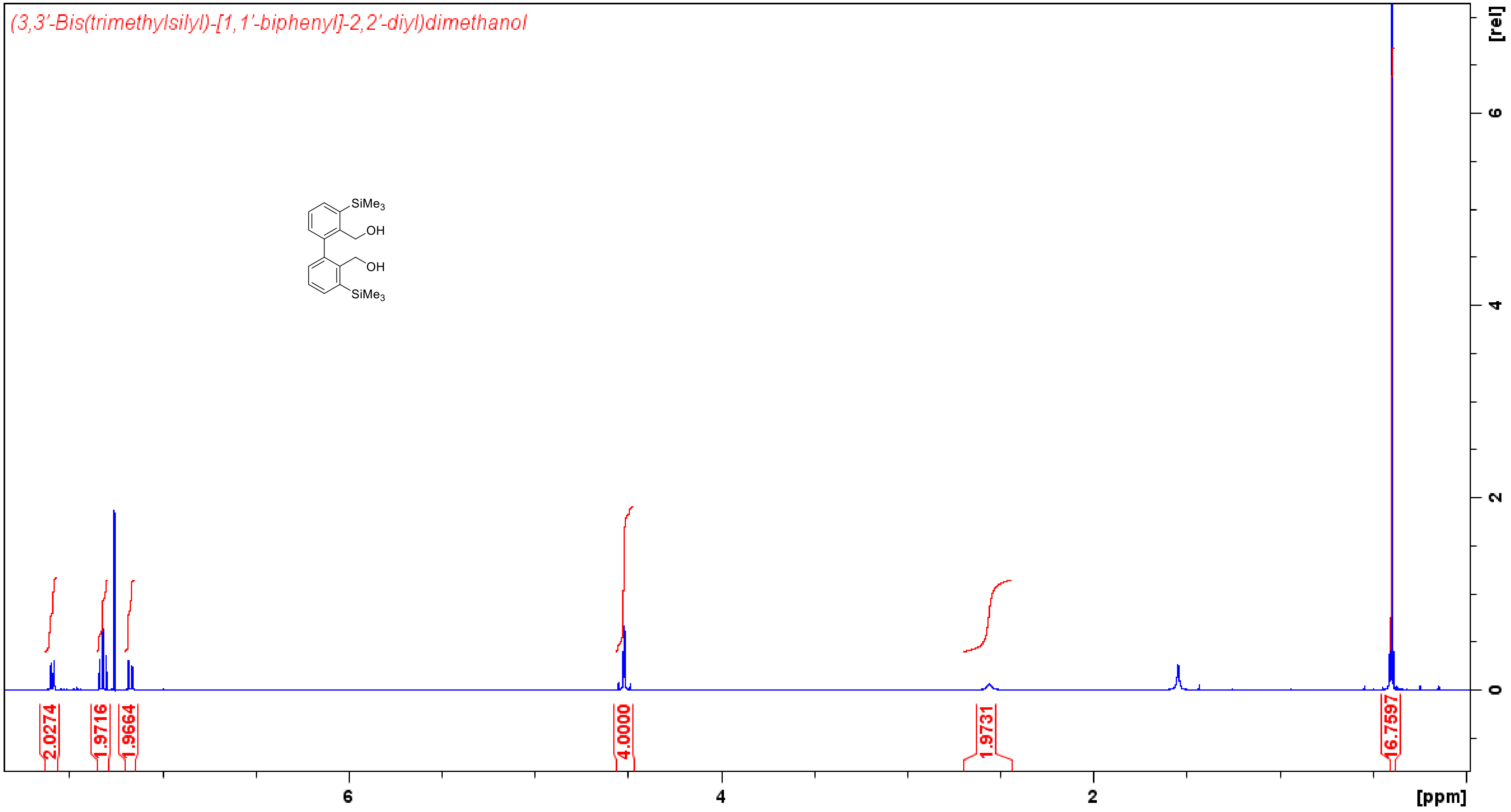
(3,3'-Bis(trimethylsilyl)-[1,1'-biphenyl]-2,2'-diyl)dimethanol
SiMe3
OH
OH
SiMe3
2.0274
1.9716
1.9664
4.0000
1.9731
16.7597
6
4
2
[ppm]
[rel]
6
4
2
0

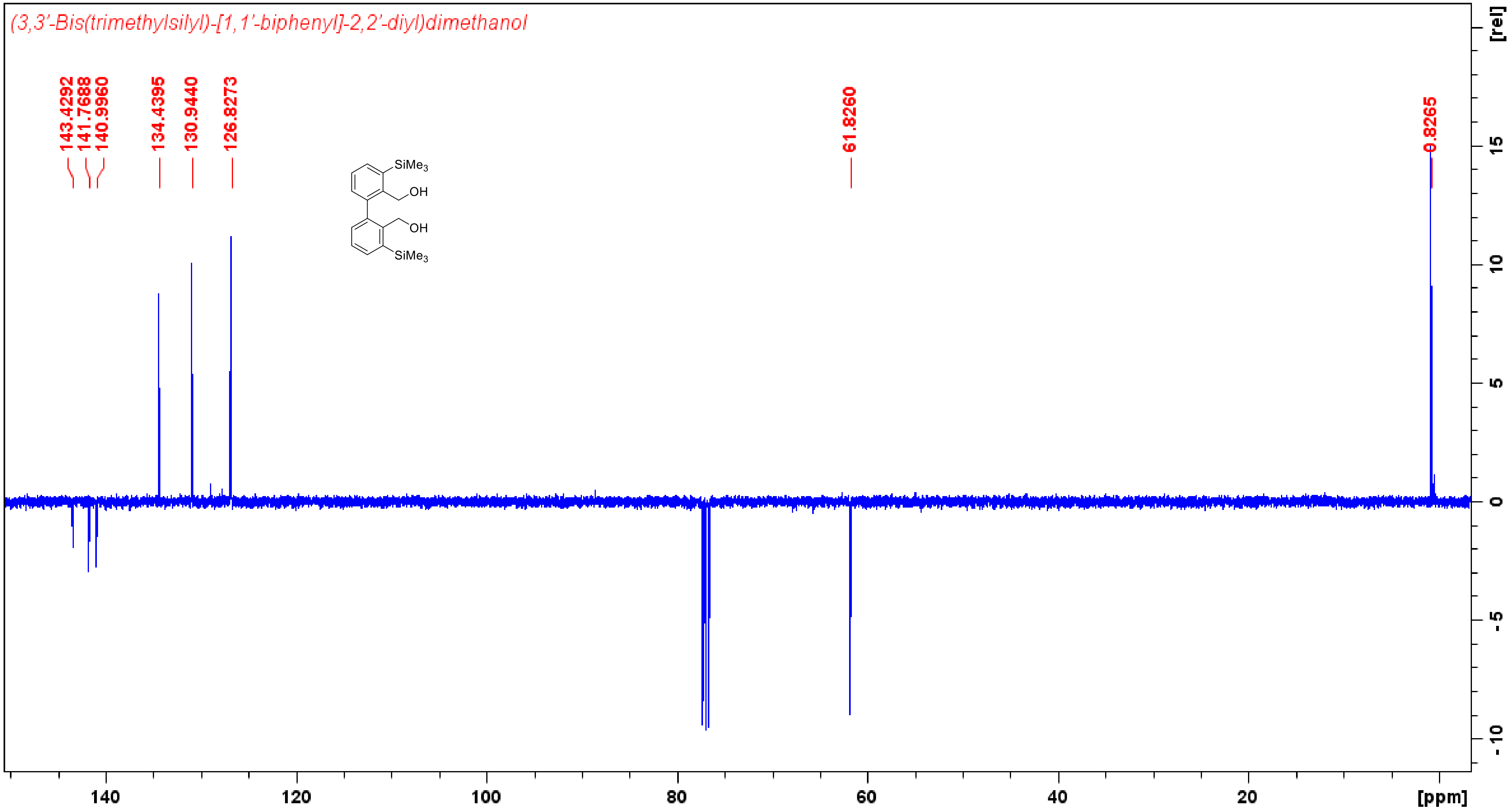

(3,3'-Bis(trimethylsilyl)-[1,1'-biphenyl]-2,2'-diyl)dimethanol
143.4292
141.7688
140.9960
134.4395
130.9440
126.8273
61.8260
0.8265
SiMe$_3$
OH
OH
SiMe$_3$
[rel]
15
10
5
0
-5
-10
140
120
100
80
60
40
20
[ppm]

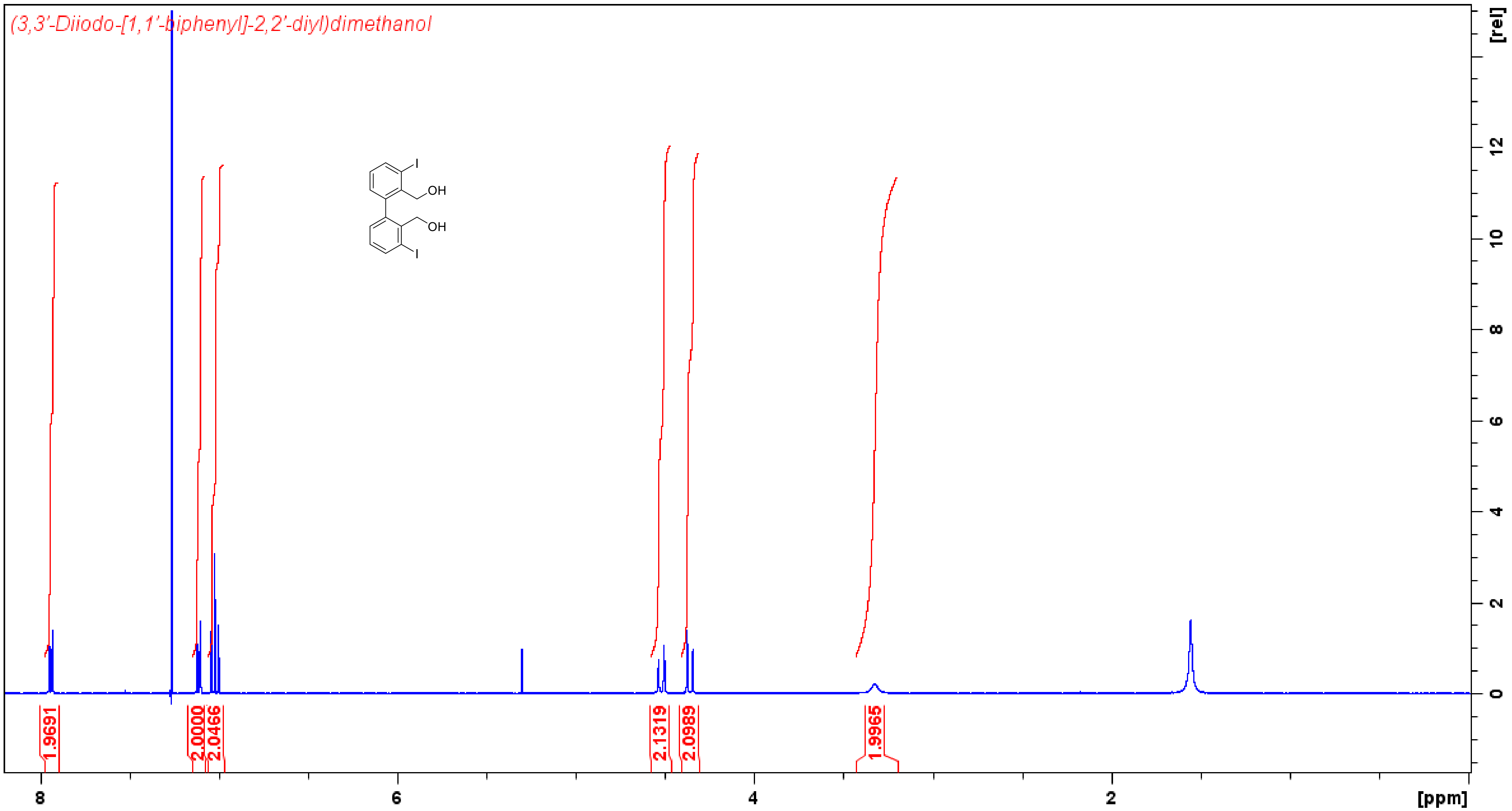

(3,3'-Diiodo-[1,1'-biphenyl]-2,2'-diyl)dimethanol
I
OH
OH
I
1.9691
2.0000
2.0466
2.1319
2.0989
1.9965
8
6
4
2
[ppm]
[rel]
0
2
4
6
8
10
12

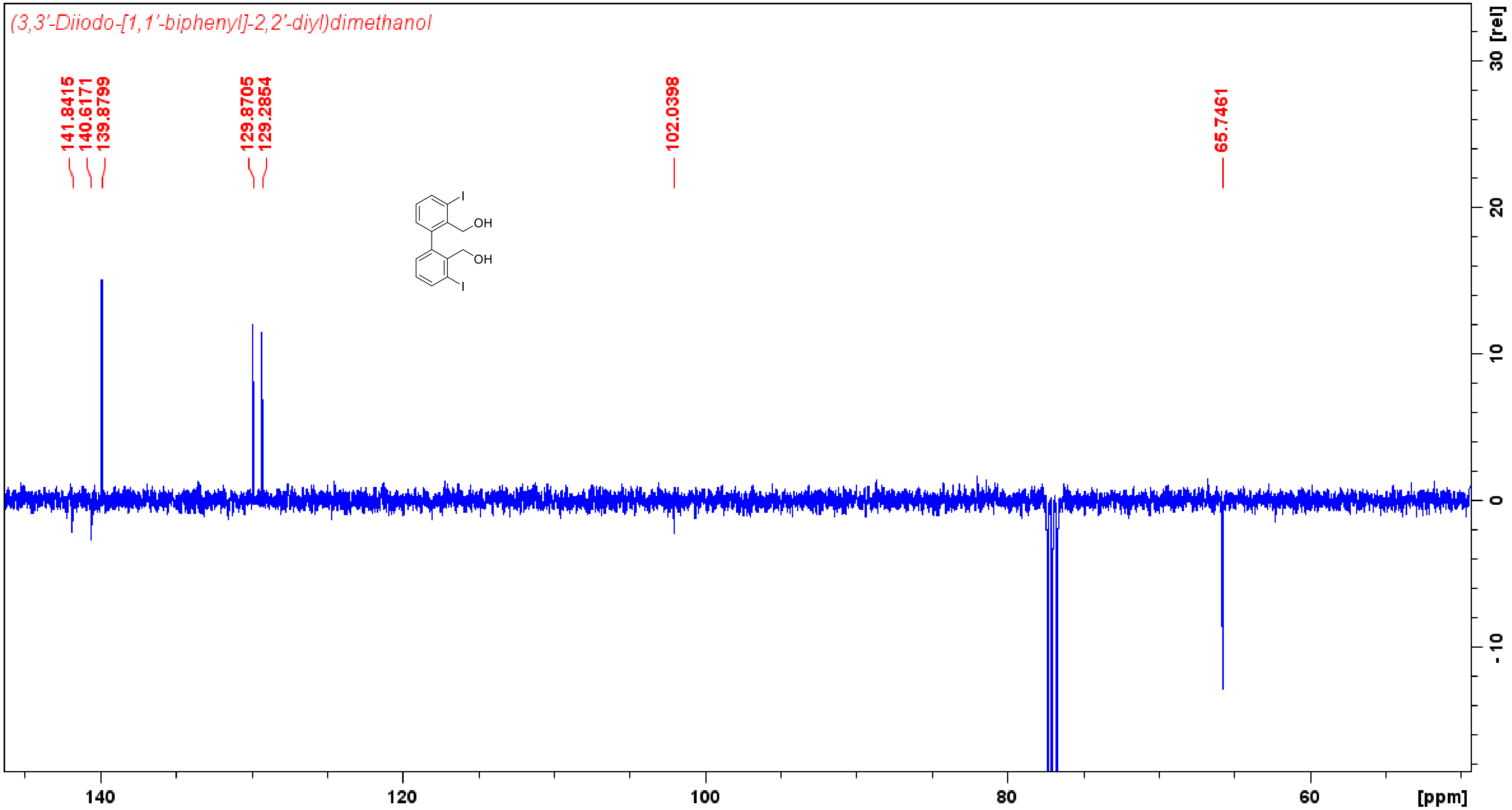
(3,3'-Diiodo-[1,1'-biphenyl]-2,2'-diyl)dimethanol
141.8415
140.6171
139.8799
129.8705
129.2854
102.0398
65.7461
I
OH
OH
I
30 [rel]
20
10
0
-10
140
120
100
80
60
[ppm]

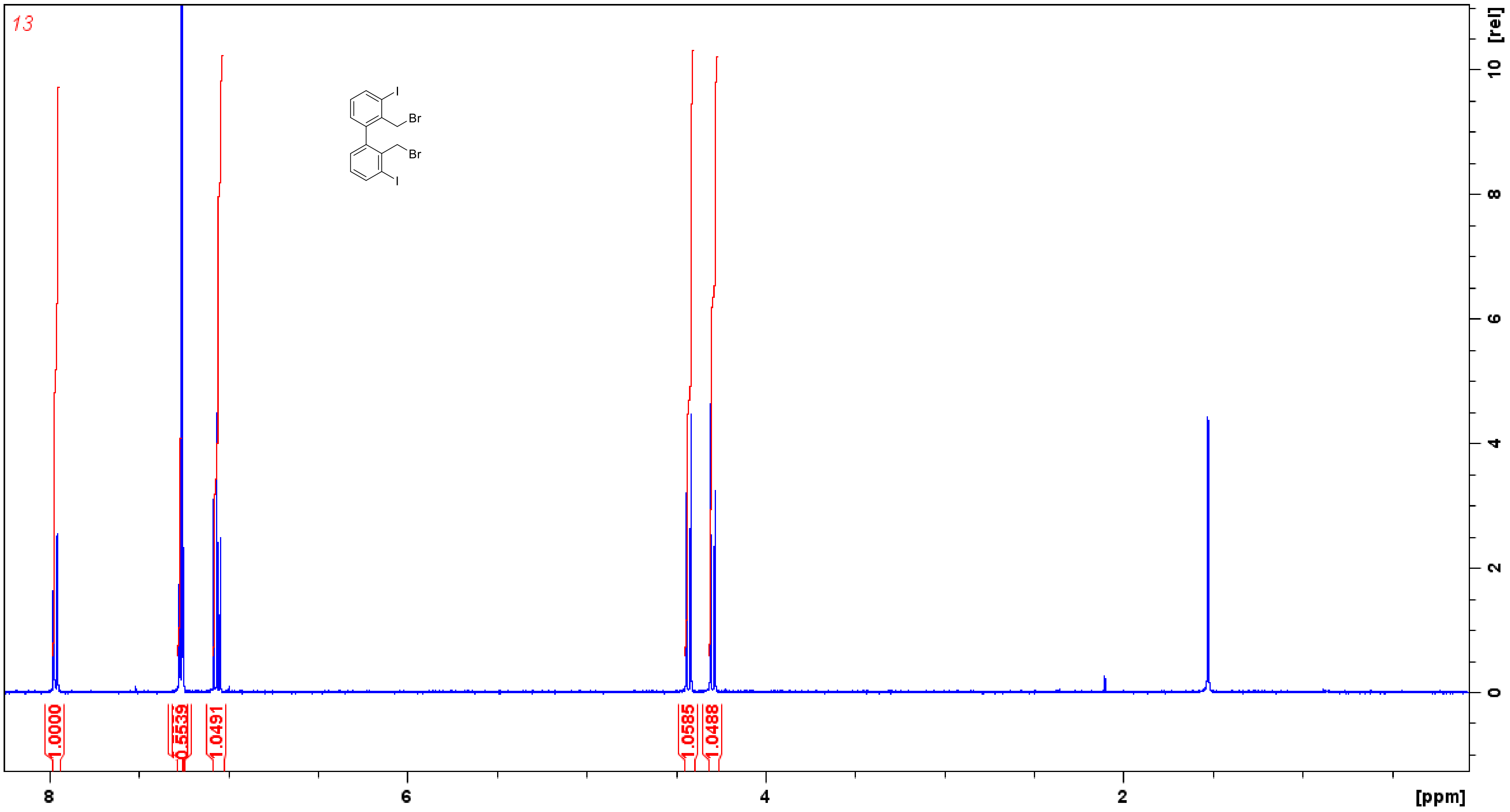
13
I
Br
Br
I
1.0000
0.5539
1.0491
1.0585
1.0488
8
6
4
2
[ppm]
10
8
6
4
2
0
[rel]

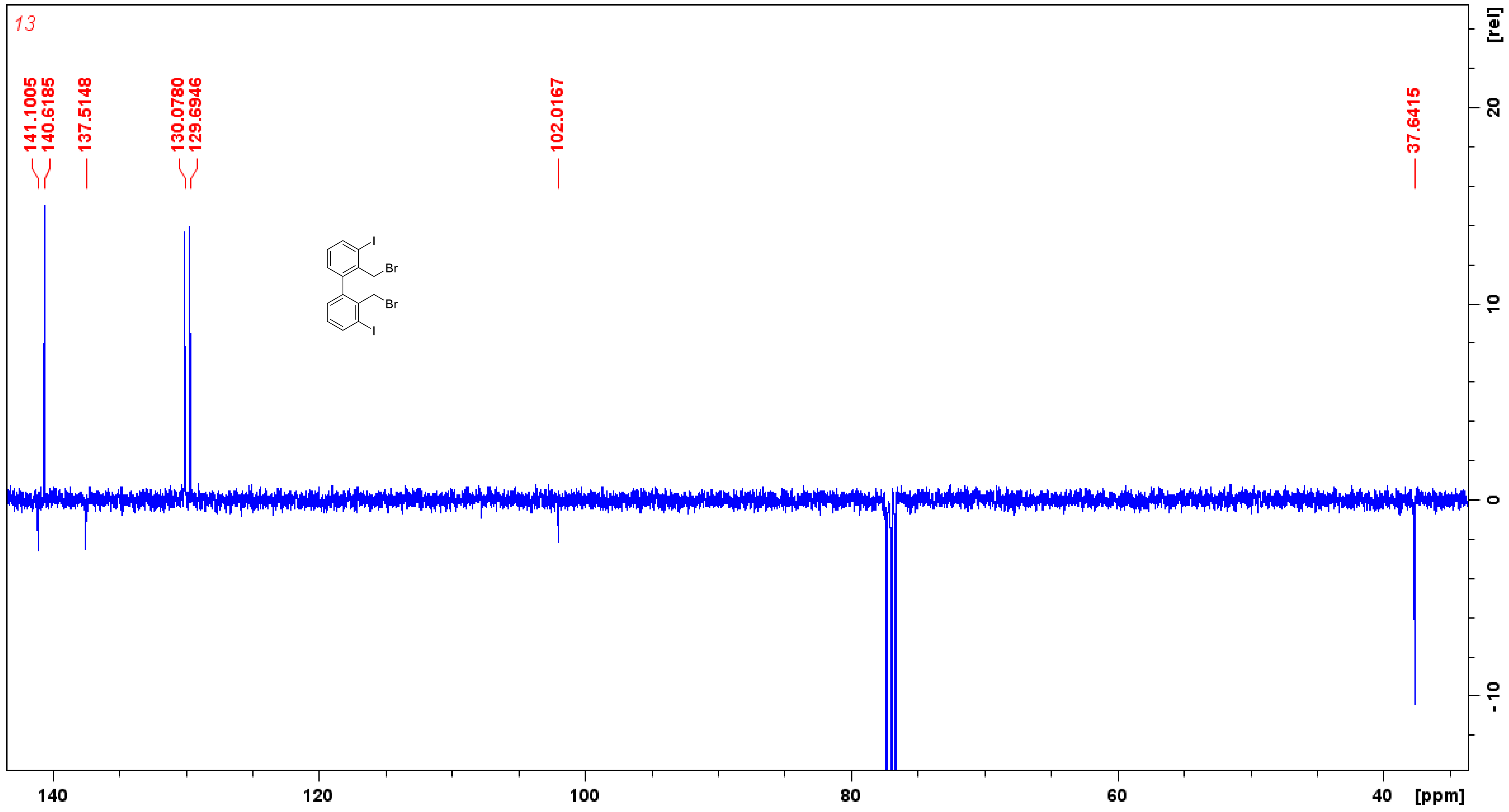
13
141.1005
140.6185
137.5148
130.0780
129.6946
102.0167
37.6415
I
Br
Br
I
[rel]
20
10
0
- 10
140
120
100
80
60
40
[ppm]

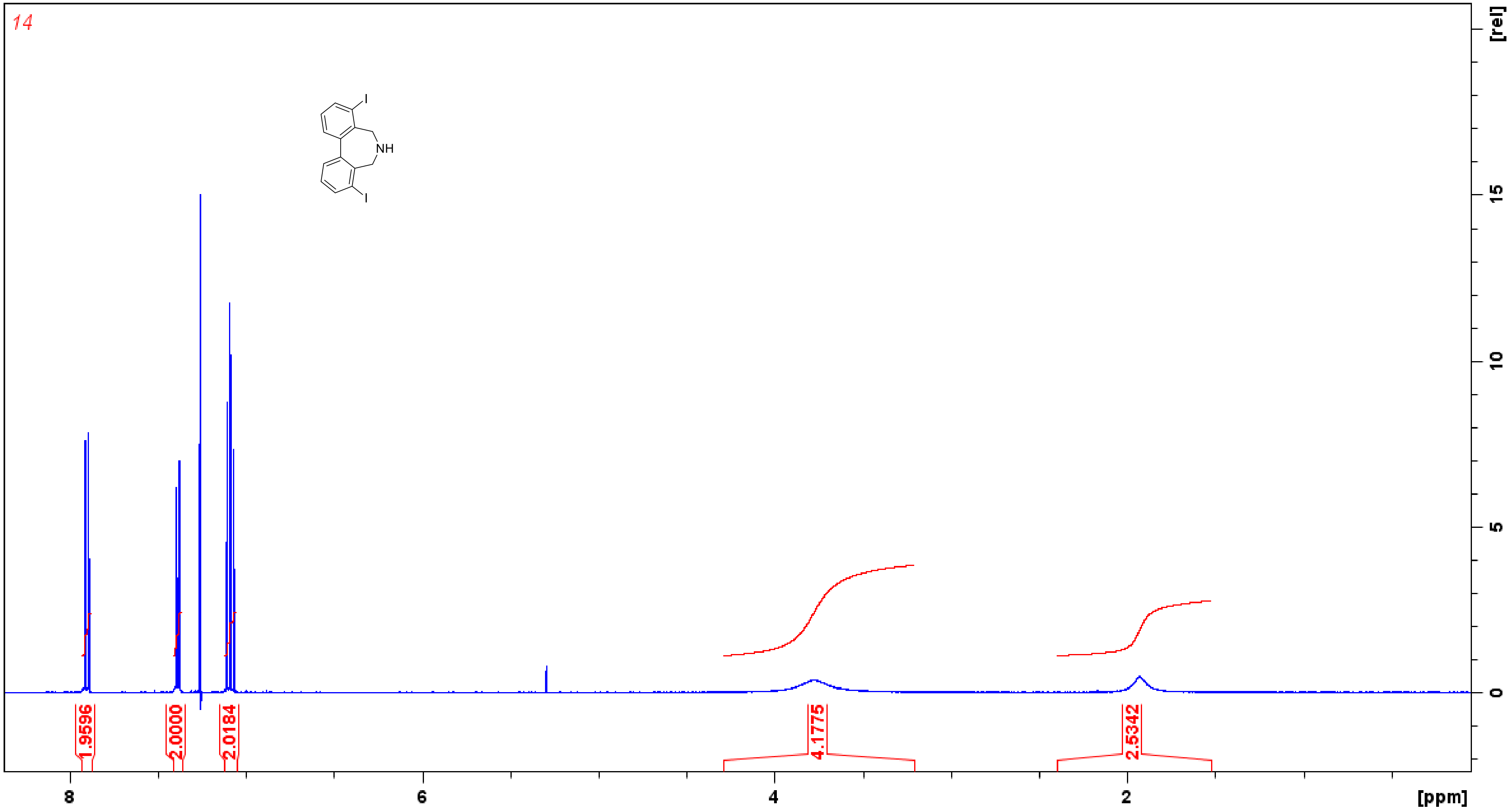
14
NH
I
I
1.9596
2.0000
2.0184
4.1775
2.5342
8
6
4
2
[ppm]
0
5
10
15
[rel]

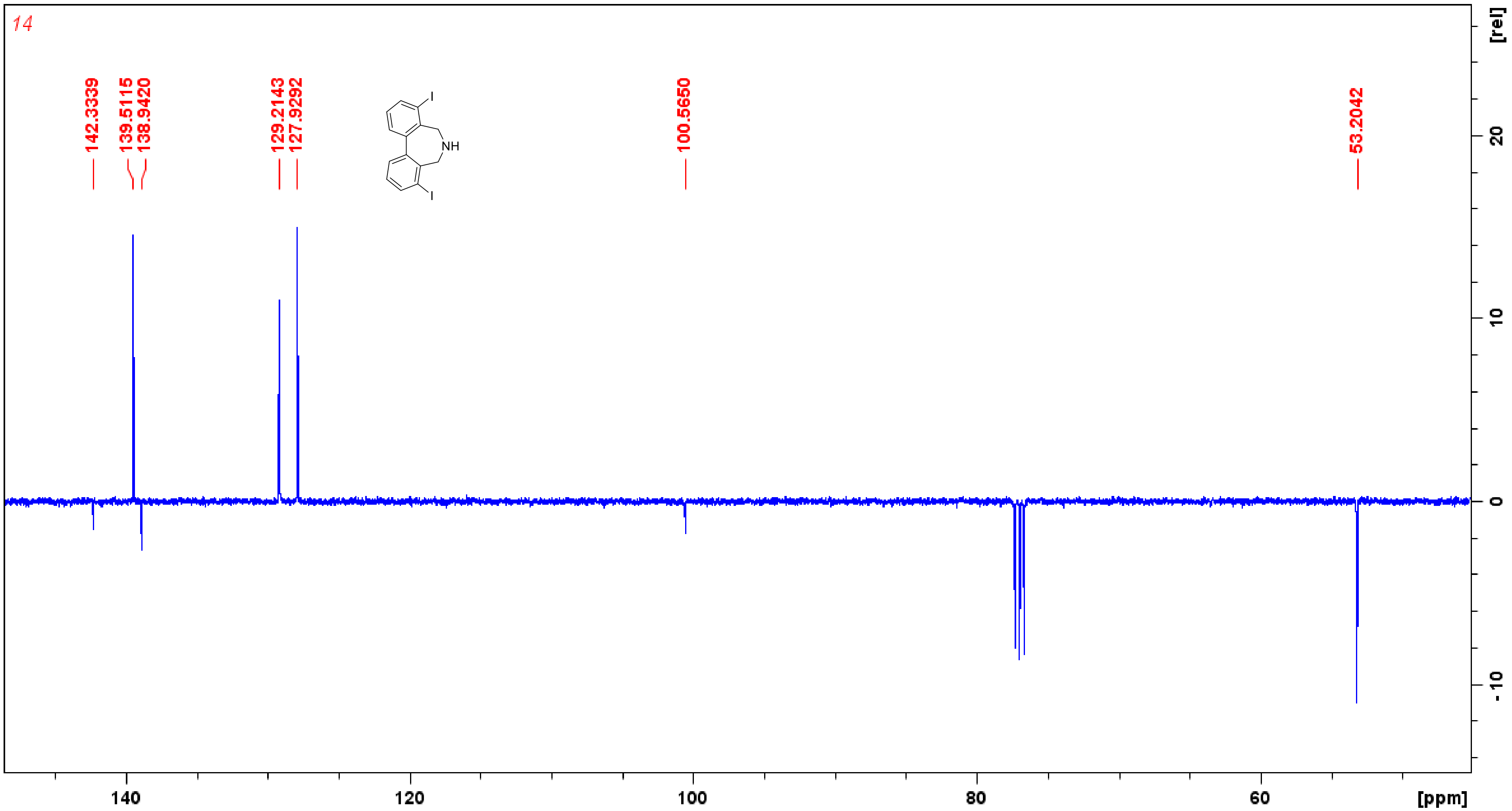
14
142.3339
139.5115
138.9420
129.2143
127.9292
100.5650
53.2042
I
NH
I
[rel]
20
10
0
-10
140
120
100
80
60
[ppm]

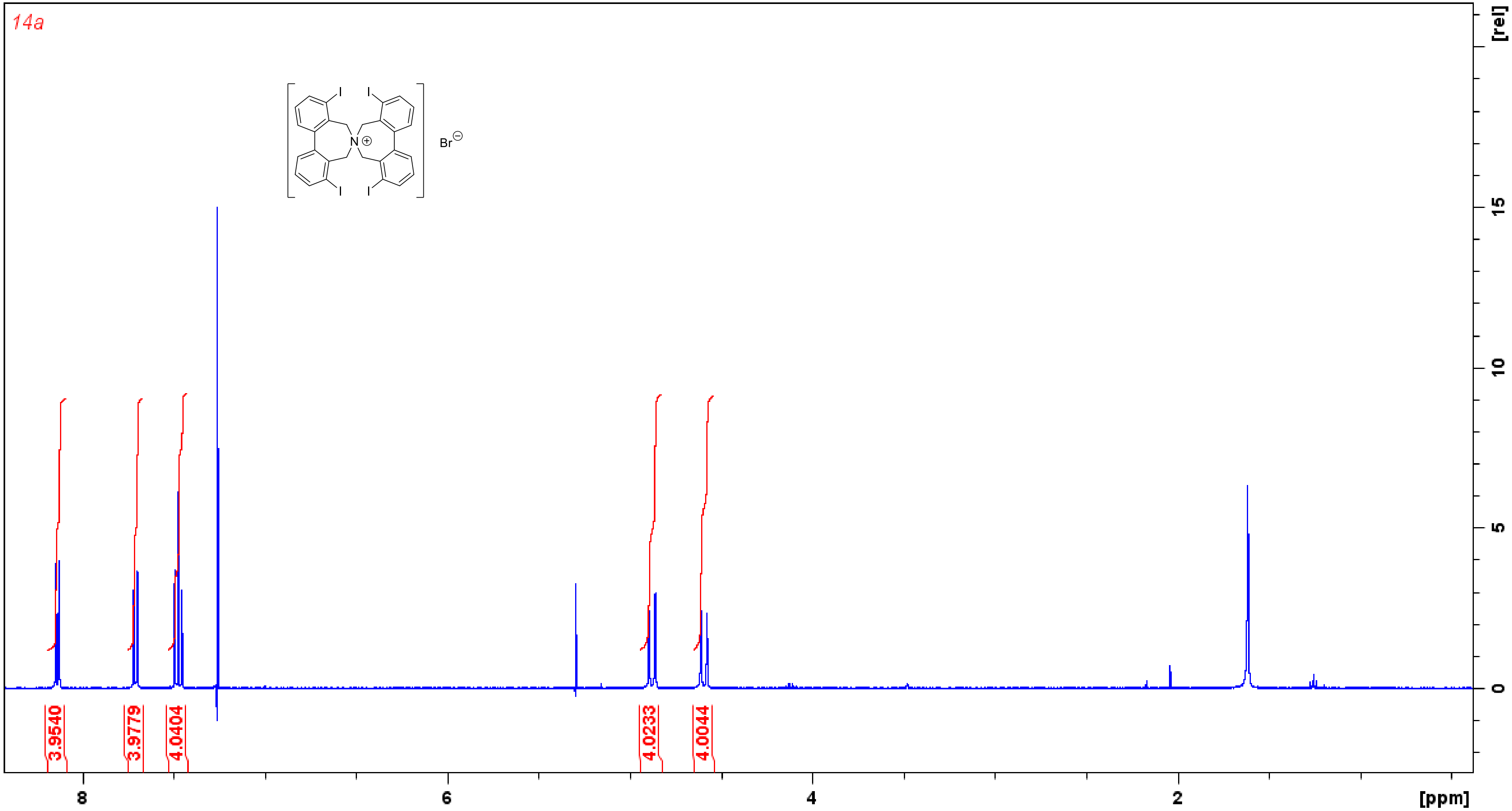
14a
I
I
I
I
N⊕
Br⊖
3.9540
3.9779
4.0404
4.0233
4.0044
8
6
4
2
[ppm]
[rel]
15
10
5
0

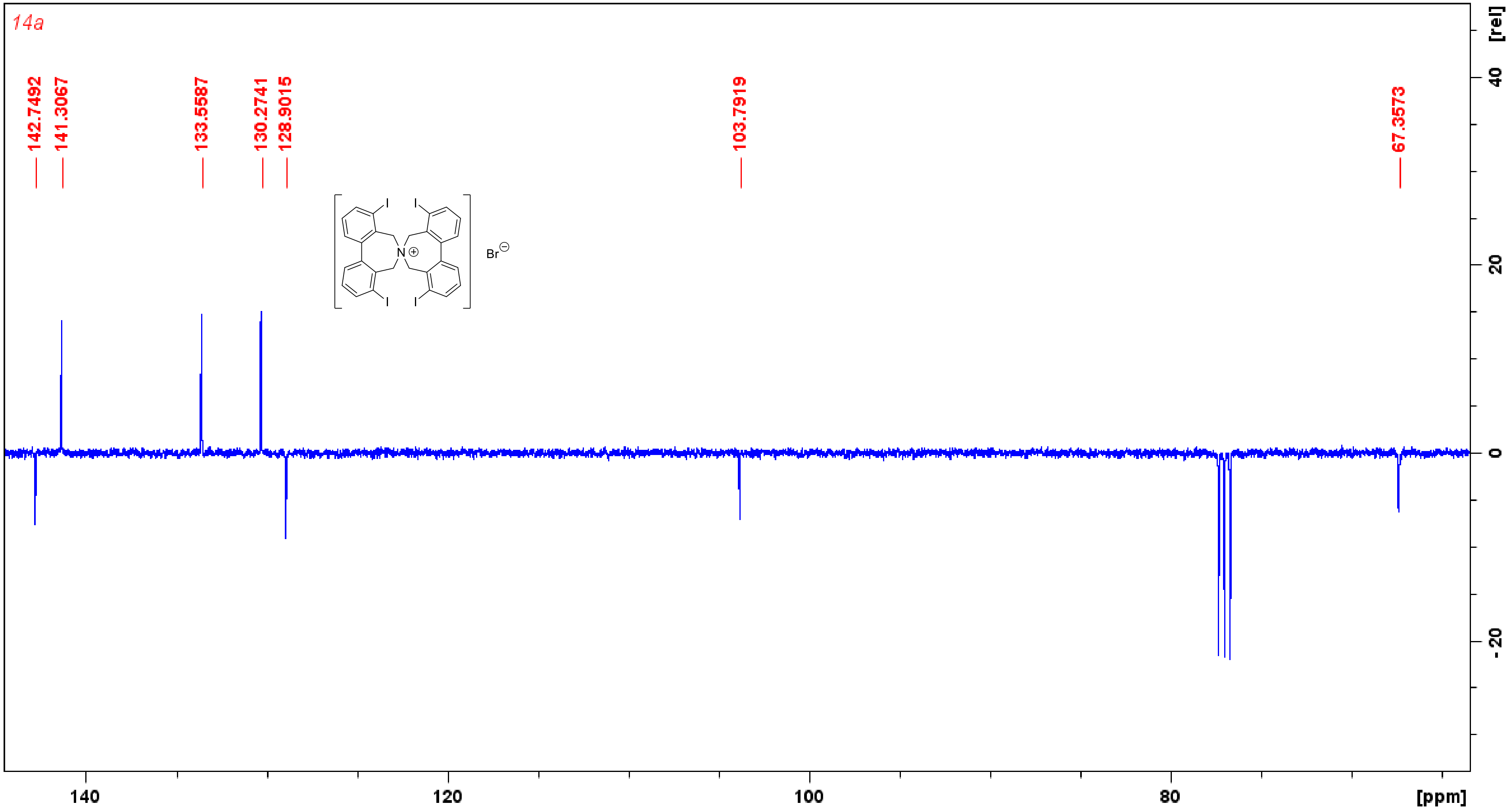
14a
142.7492
141.3067
133.5587
130.2741
128.9015
103.7919
67.3573
Br
N
I
[rel]
40
20
0
-20
140
120
100
80
[ppm]

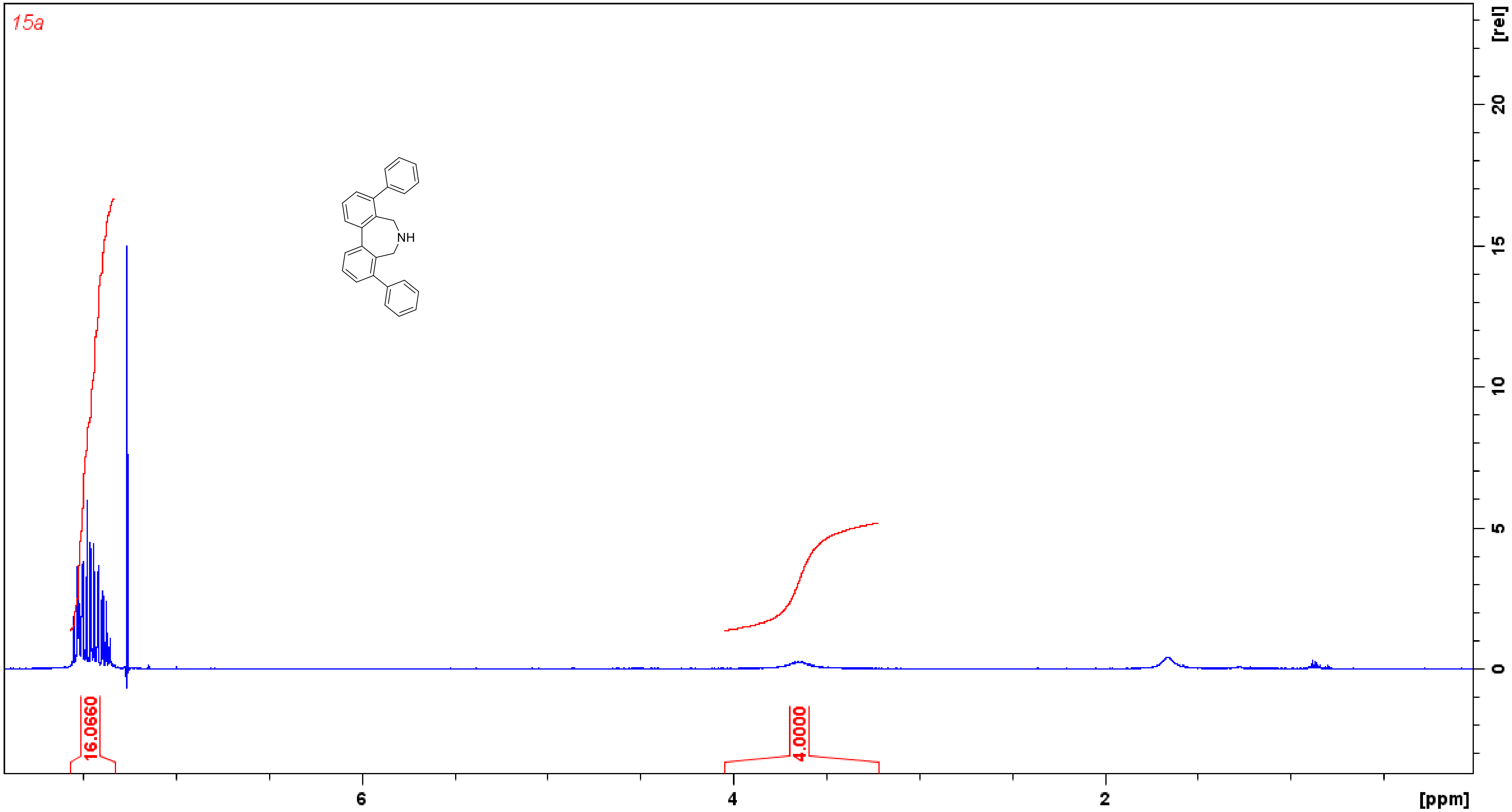
15a
NH
16.0660
4.0000
6
4
2
[ppm]
[rel]
20
15
10
5
0

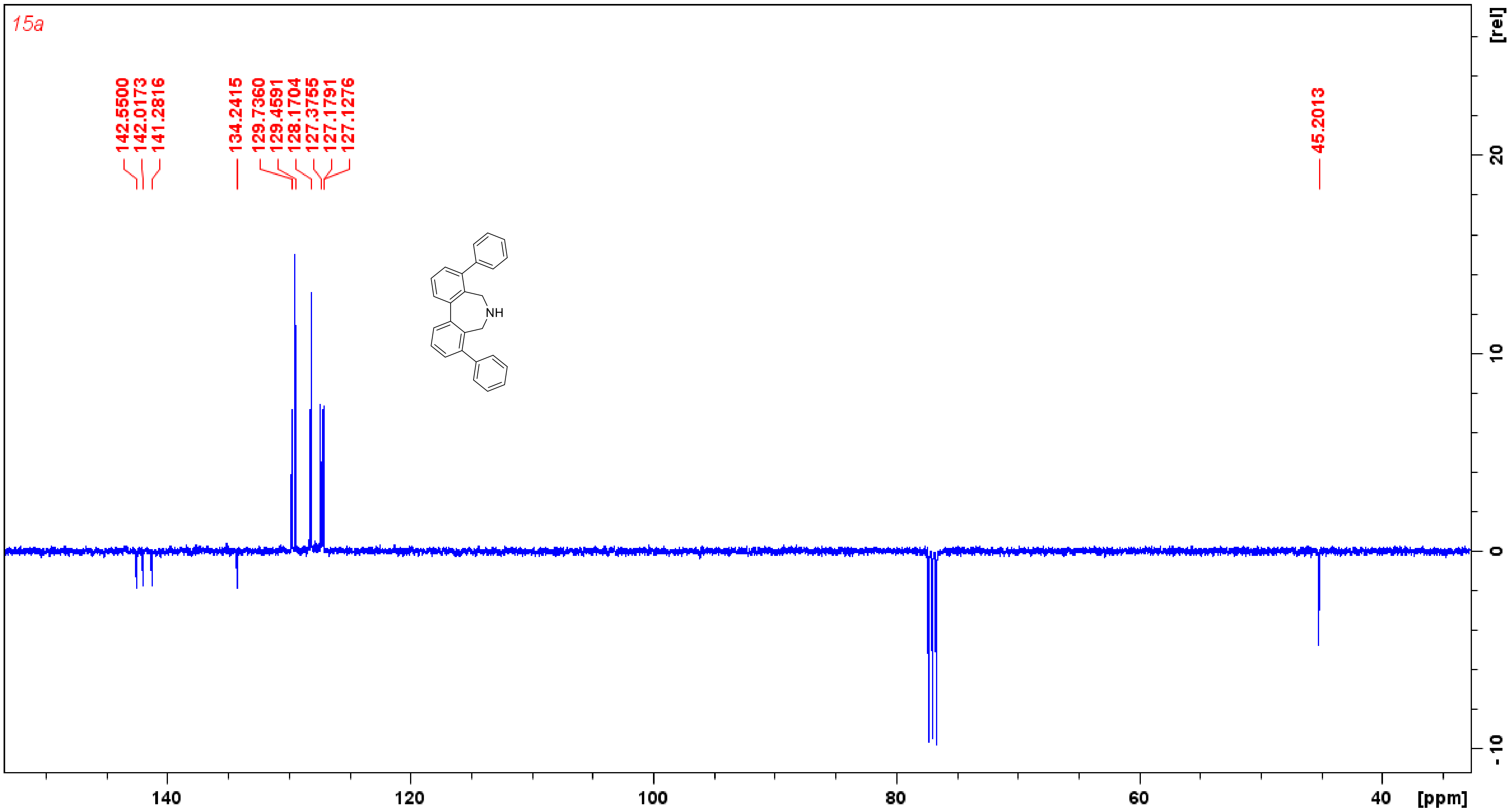
15a
142.5500
142.0173
141.2816
134.2415
129.7360
129.4591
128.1704
127.3755
127.1791
127.1276
45.2013
NH
[rel]
20
10
0
-10
140
120
100
80
60
40
[ppm]

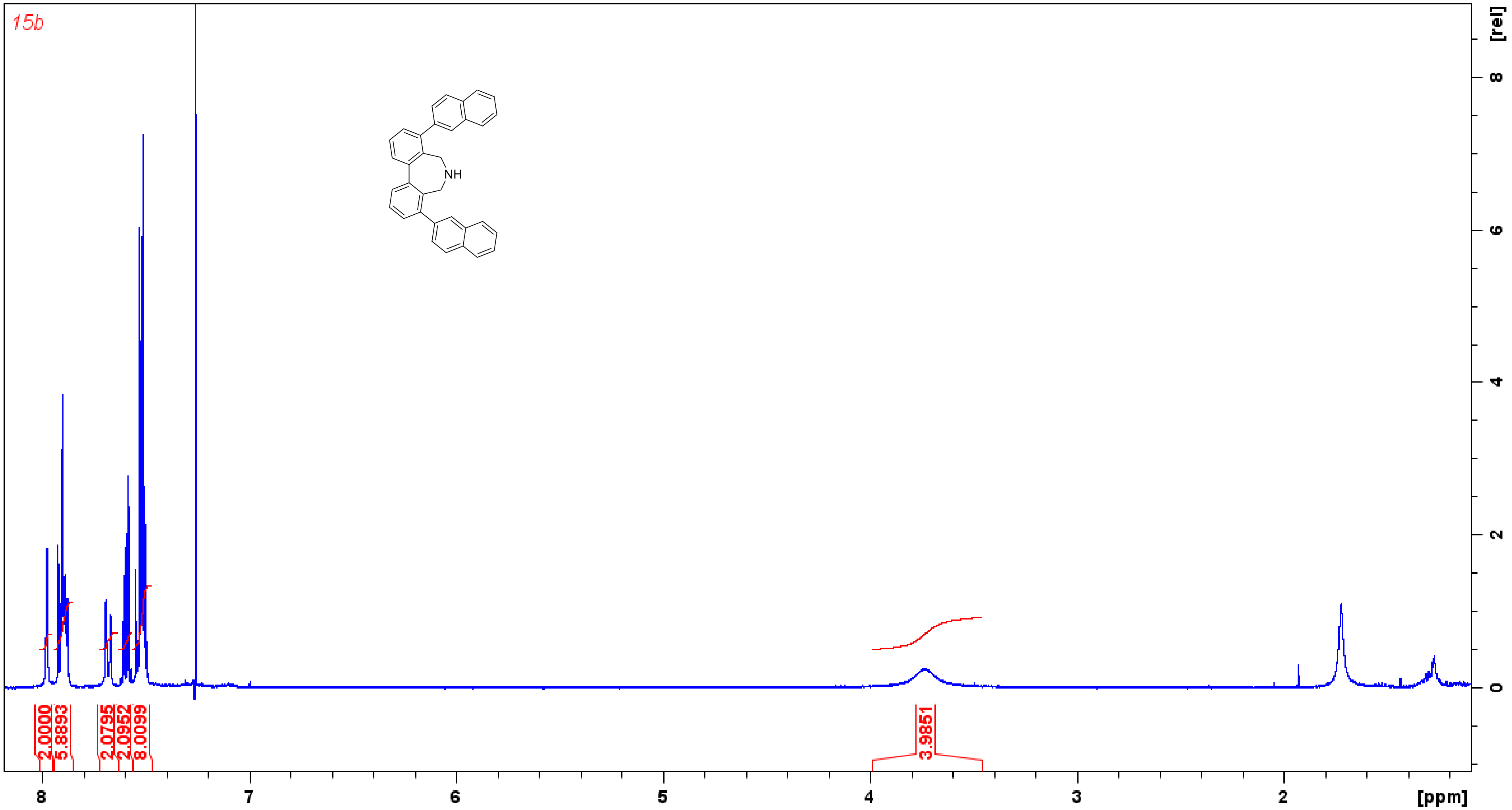
15b
NH
[rel]
8
6
4
2
0
2.0000
5.8893
2.0795
2.0952
8.0099
3.9851
8
7
6
5
4
3
2
[ppm]

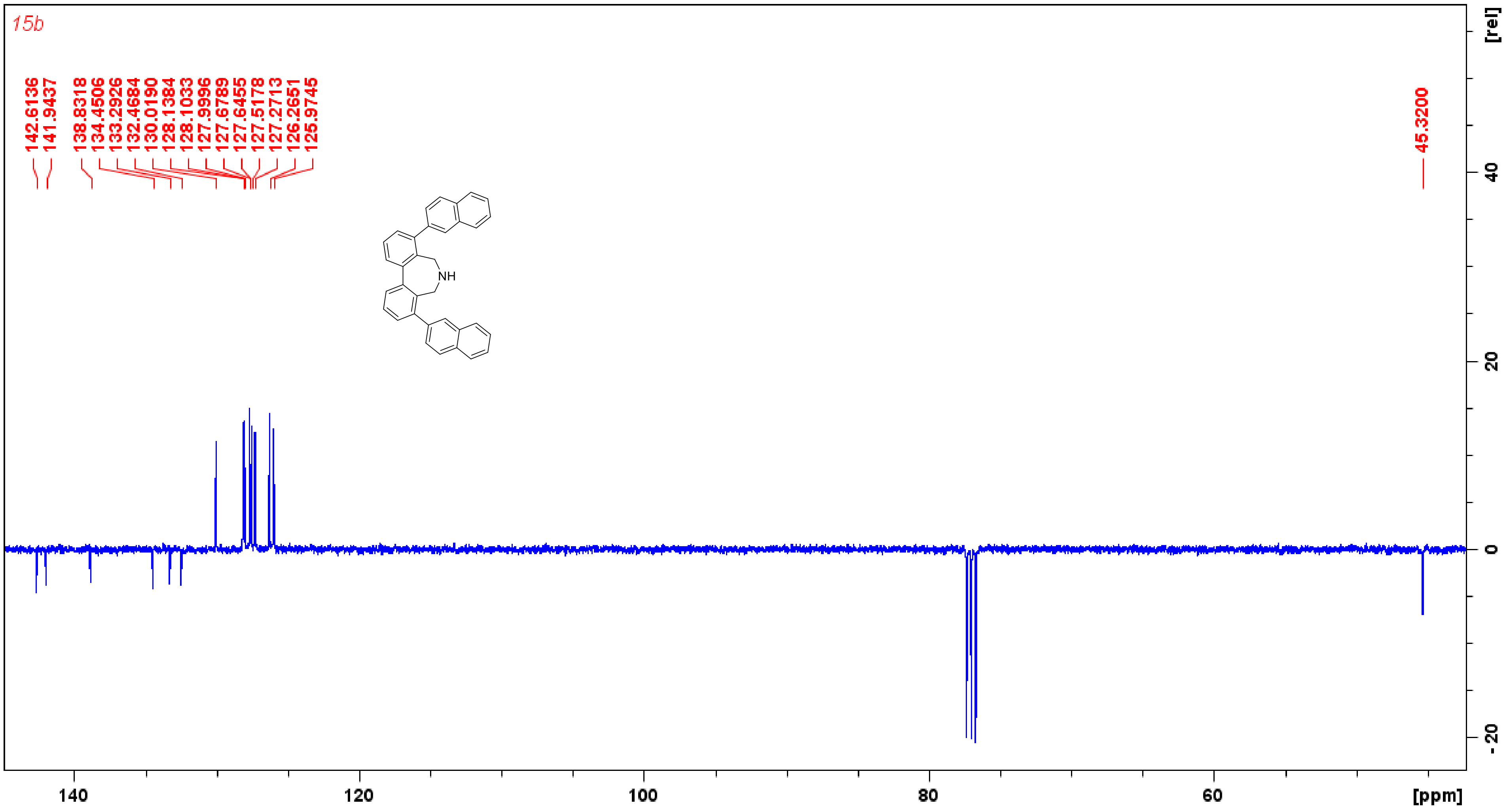
15b
142.6136
141.9437
138.8318
134.4506
133.2926
132.4684
130.0190
128.1384
128.1033
127.9996
127.6789
127.6455
127.5178
127.2713
126.2651
125.9745
45.3200
NH
[rel]
40
20
0
-20
140
120
100
80
60
[ppm]

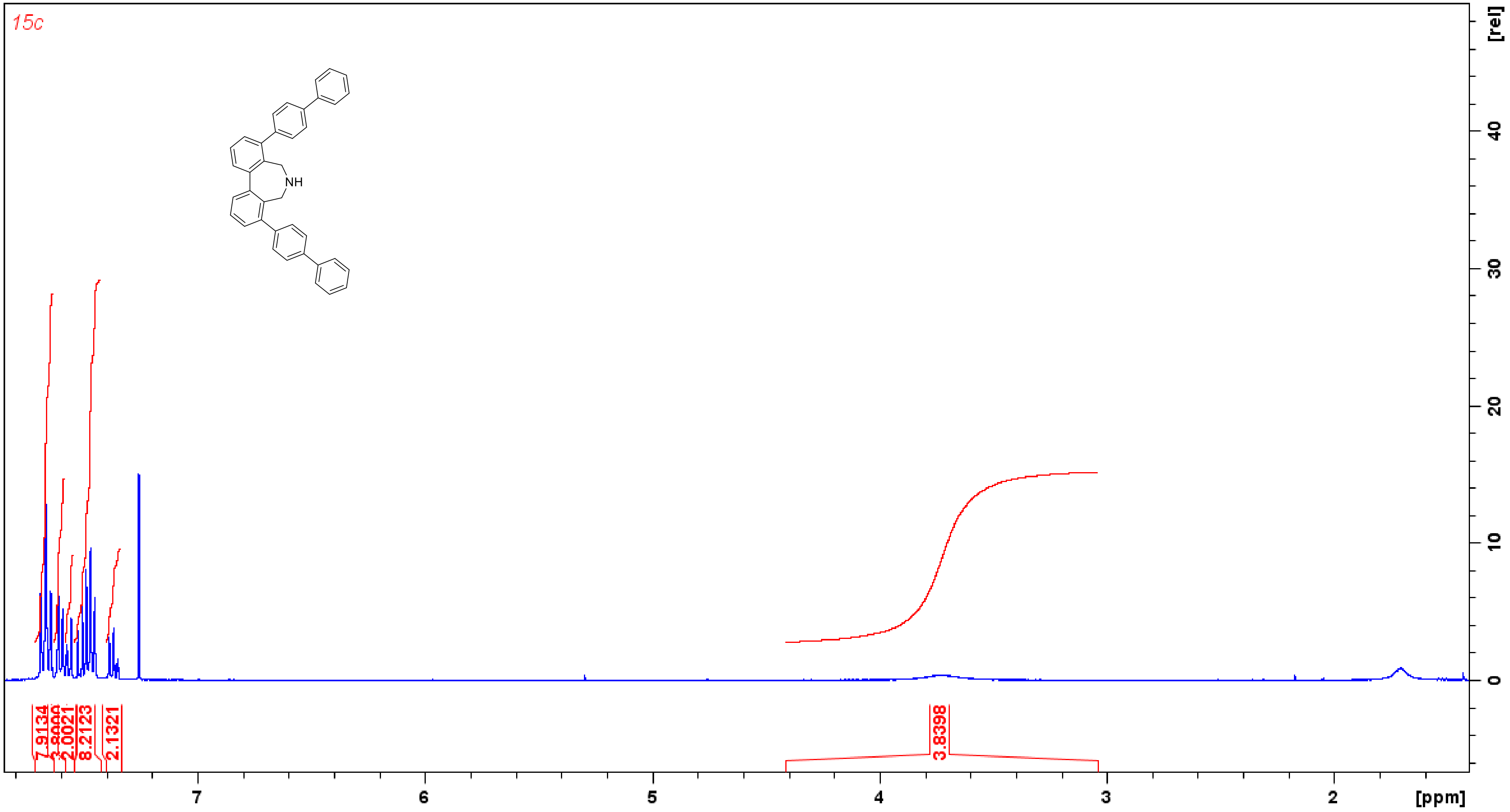
15c
NH
[rel]
40
30
20
10
0
7.9134
3.8000
2.0021
8.2123
2.1321
3.8398
7
6
5
4
3
2
[ppm]

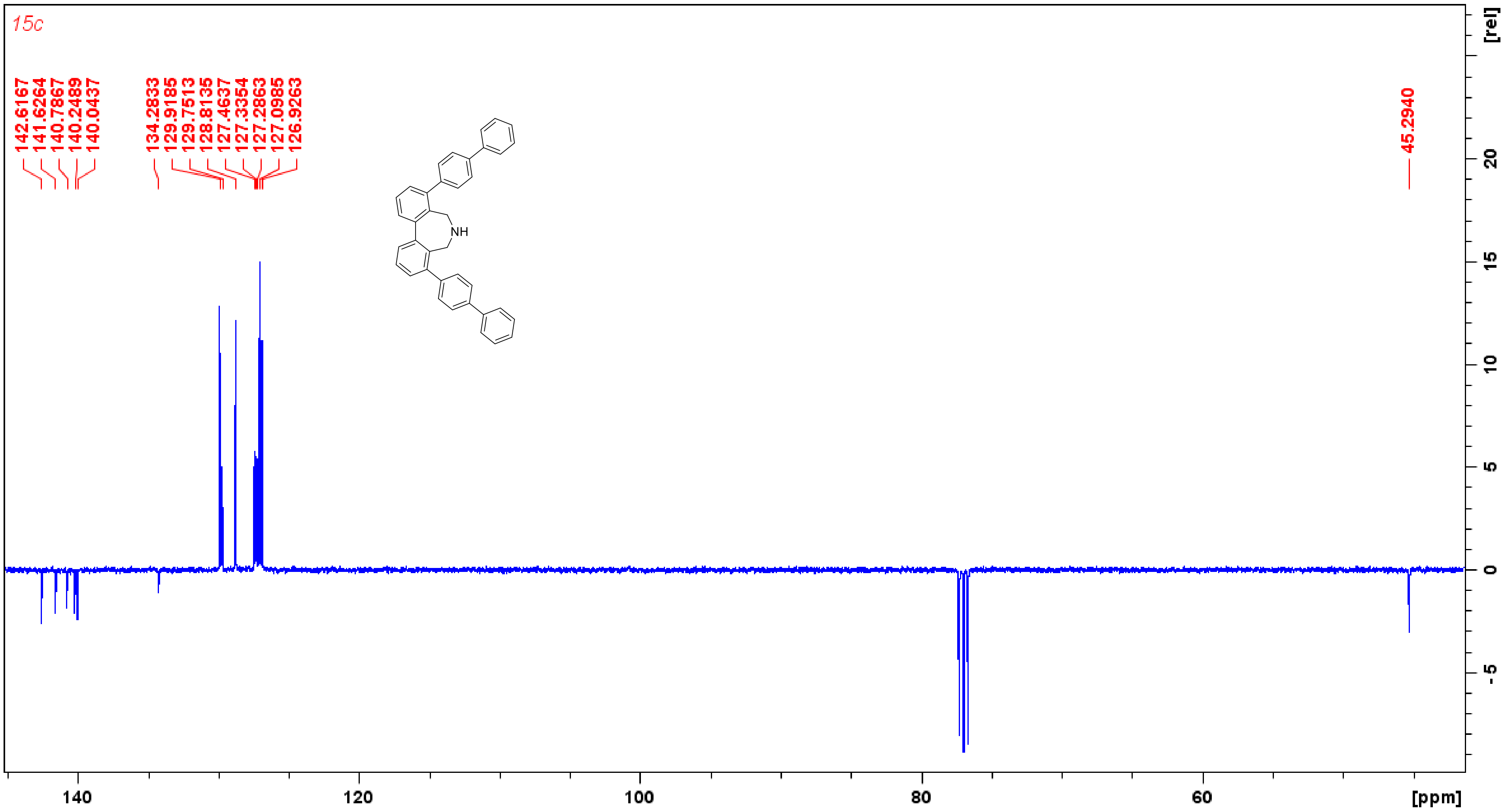
15c
142.6167
141.6264
140.7867
140.2489
140.0437
134.2833
129.9185
129.7513
128.8135
127.4637
127.3354
127.2863
127.0985
126.9263
45.2940
NH
[rel]
20
15
10
5
0
-5
140
120
100
80
60
[ppm]

in DMSO-d_6

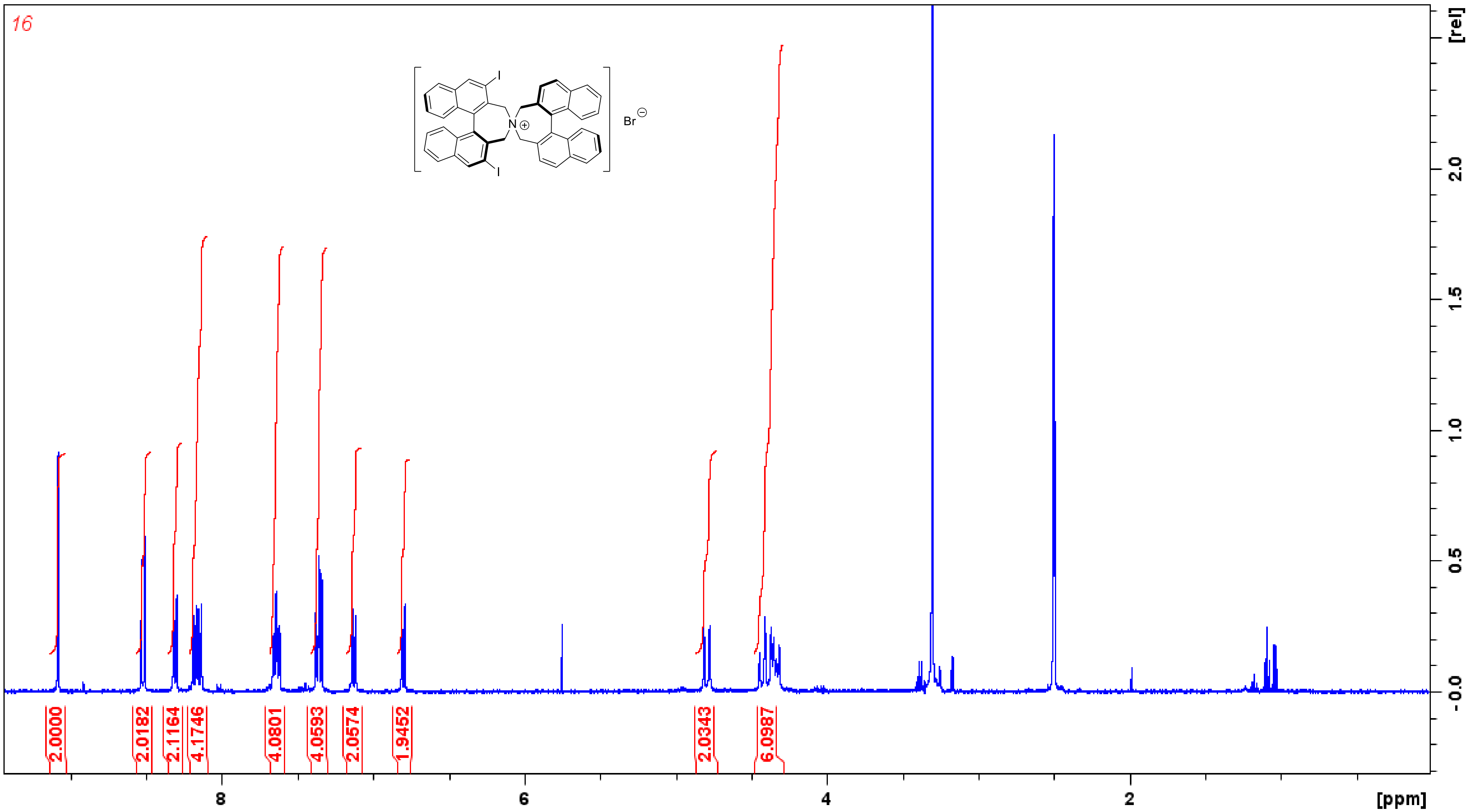
16
Br
2.0000
2.0182
2.1164
4.1746
4.0801
4.0593
2.0574
1.9452
2.0343
6.0987
[ppm]
[rel]

in DMSO-d_6

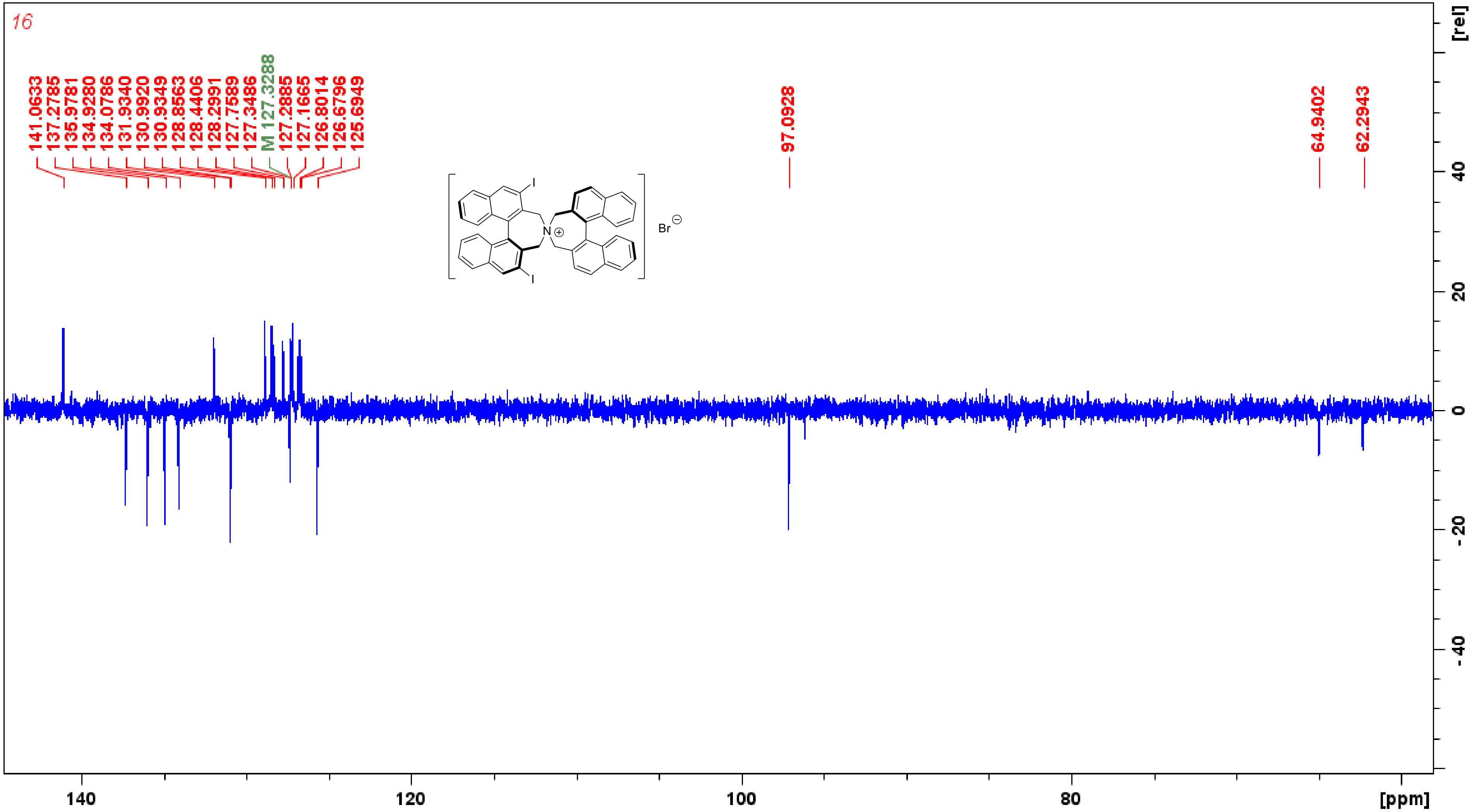
16
141.0633
137.2785
135.9781
134.9280
134.0786
131.9340
130.9920
130.9349
128.8563
128.4406
128.2991
127.7589
127.3486
M 127.3288
127.2885
127.1665
126.8014
126.6796
125.6949
97.0928
64.9402
62.2943
I
I
N
Br
[rel]
40
20
0
-20
-40
140
120
100
80
[ppm]

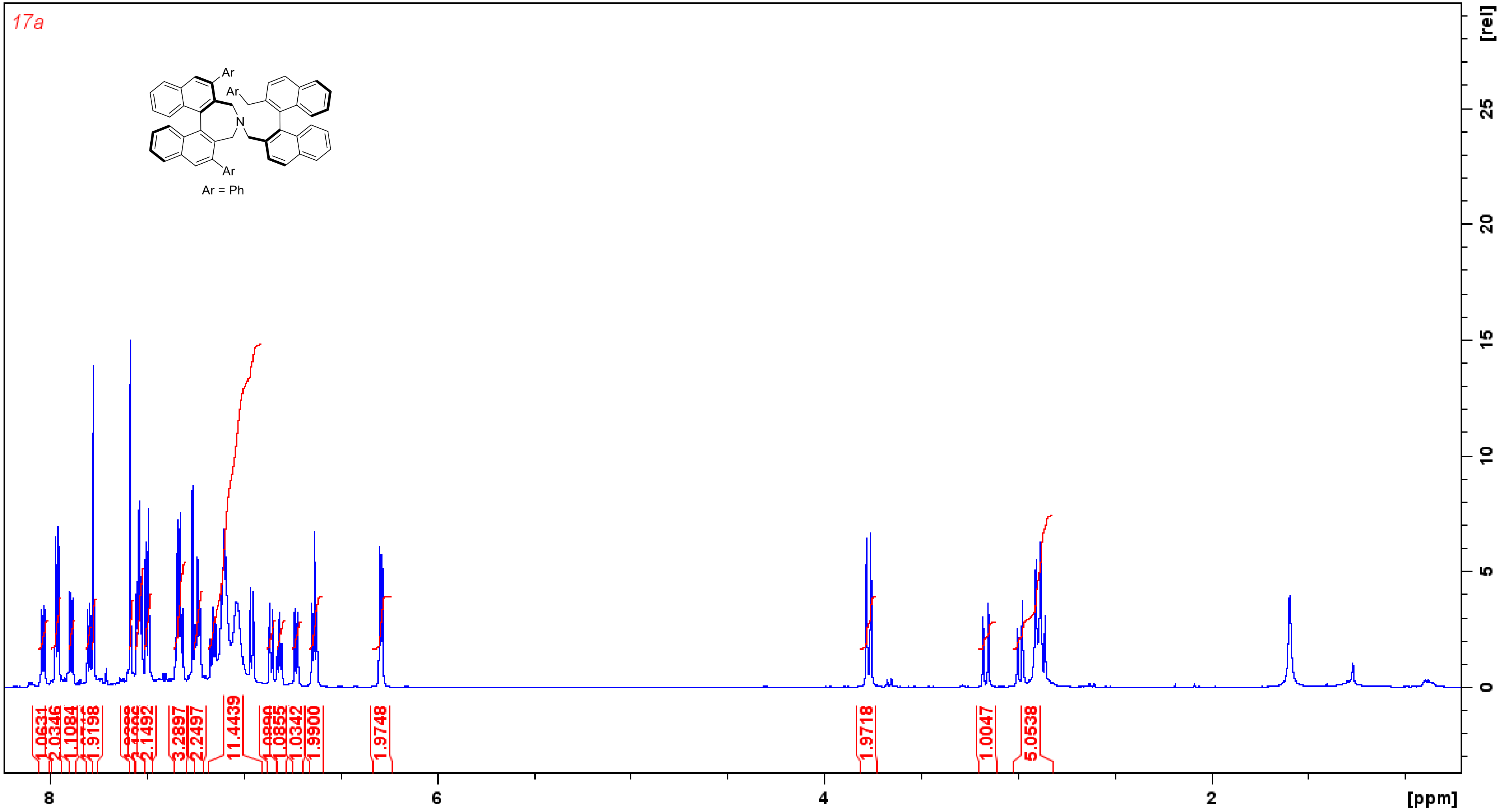
17a
Ar
Ar
N
Ar
Ar = Ph
1.0631
2.0346
1.1084
1.9198
2.1492
3.2897
2.2497
11.4439
1.0855
1.0342
1.9900
1.9748
1.9718
1.0047
5.0538
[rel]
[ppm]

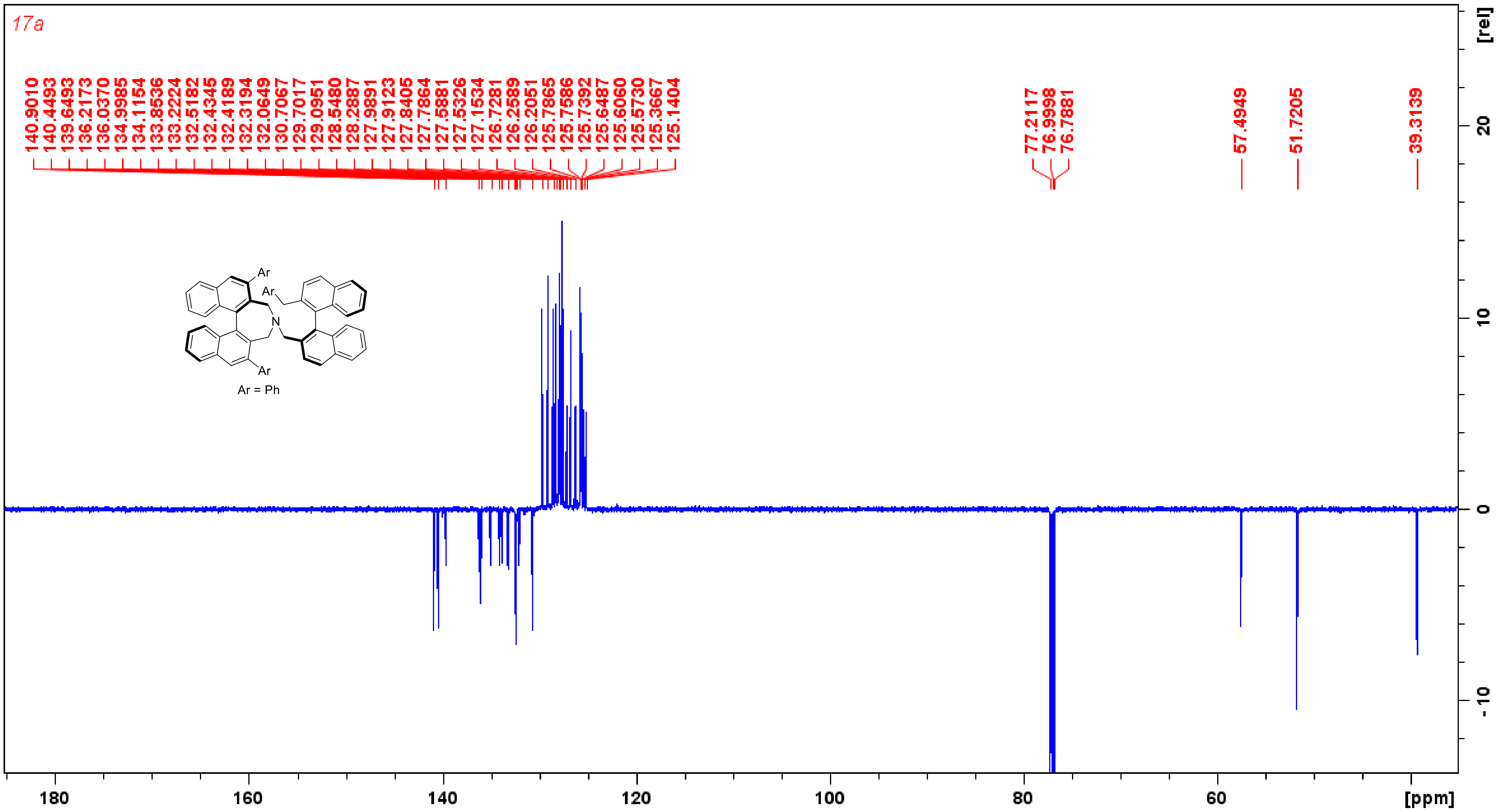
17a
140.9010
140.4493
139.6493
136.2173
136.0370
134.9985
134.1154
133.8536
133.2224
132.5182
132.4345
132.4189
132.3194
132.0649
130.7067
129.7017
129.0951
128.5480
128.2887
127.9891
127.9123
127.8405
127.7864
127.5881
127.5326
127.1534
126.7281
126.2589
126.2051
125.7865
125.7586
125.7392
125.6487
125.6060
125.5730
125.3667
125.1404
77.2117
76.9998
76.7881
57.4949
51.7205
39.3139
Ar
Ar
N
Ar
Ar = Ph
[rel]
20
10
0
-10
180
160
140
120
100
80
60
[ppm]

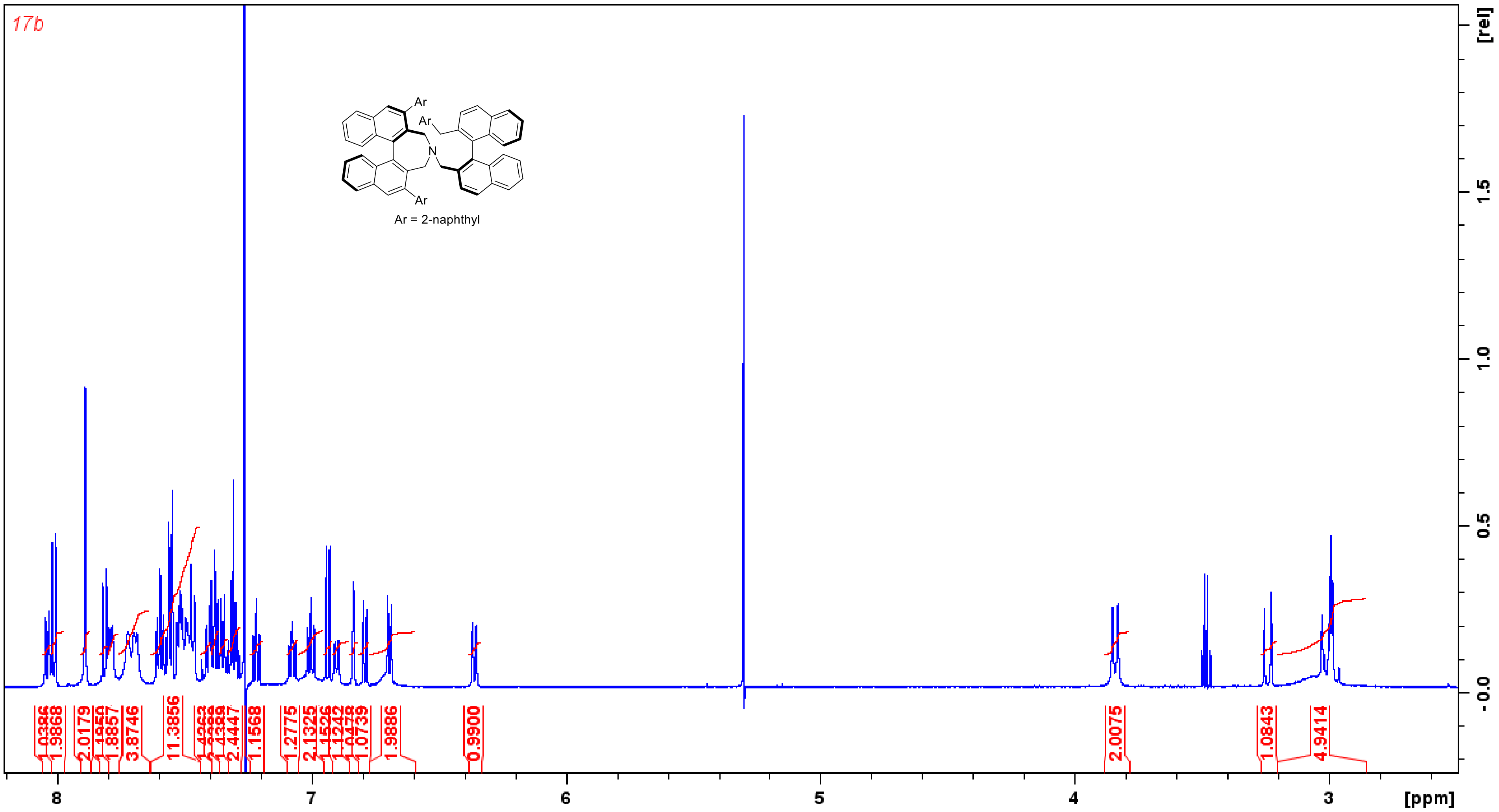
17b
Ar
Ar
N
Ar
Ar = 2-naphthyl
1.9868
2.0179
1.8857
3.8746
11.3856
2.4447
1.1568
1.2775
2.1325
1.0739
1.9886
0.9900
2.0075
1.0843
4.9414
8
7
6
5
4
3
[ppm]
0.0
0.5
1.0
1.5
[rel]

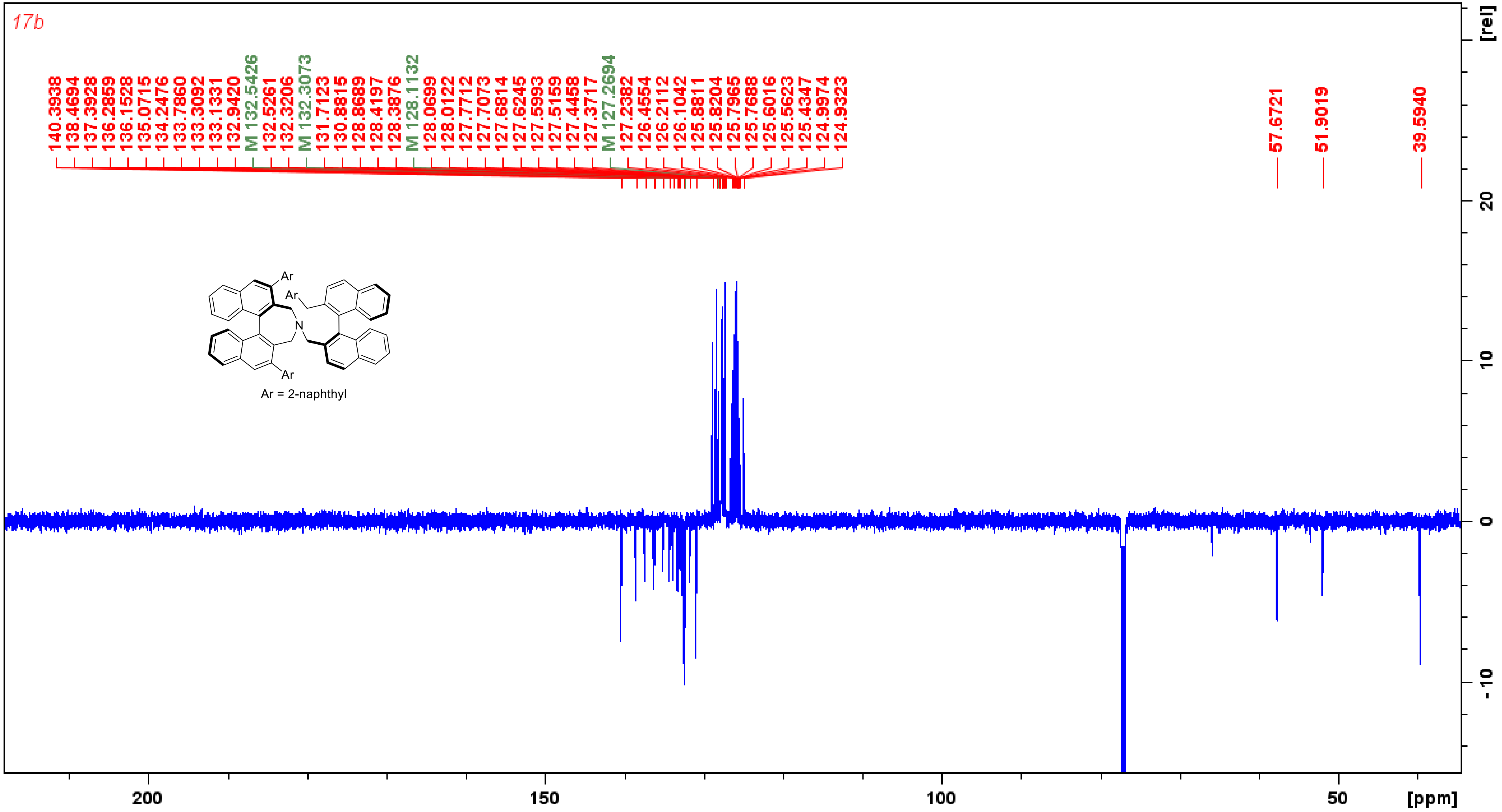
17b
Ar
Ar
N
Ar
Ar = 2-naphthyl
140.3938
138.4694
137.3928
136.2859
136.1528
135.0715
134.2476
133.7860
133.3092
133.1331
132.9420
M 132.5426
132.5261
132.3206
M 132.3073
131.7123
130.8815
128.8689
128.4197
128.3876
M 128.1132
128.0699
128.0122
127.7712
127.7073
127.6814
127.6245
127.5993
127.5159
127.4458
127.3717
M 127.2694
127.2382
126.4554
126.2112
126.1042
125.8811
125.8204
125.7965
125.7688
125.6016
125.5623
125.4347
124.9974
124.9323
57.6721
51.9019
39.5940
200
150
100
50
[ppm]
[rel]
20
10
0
-10

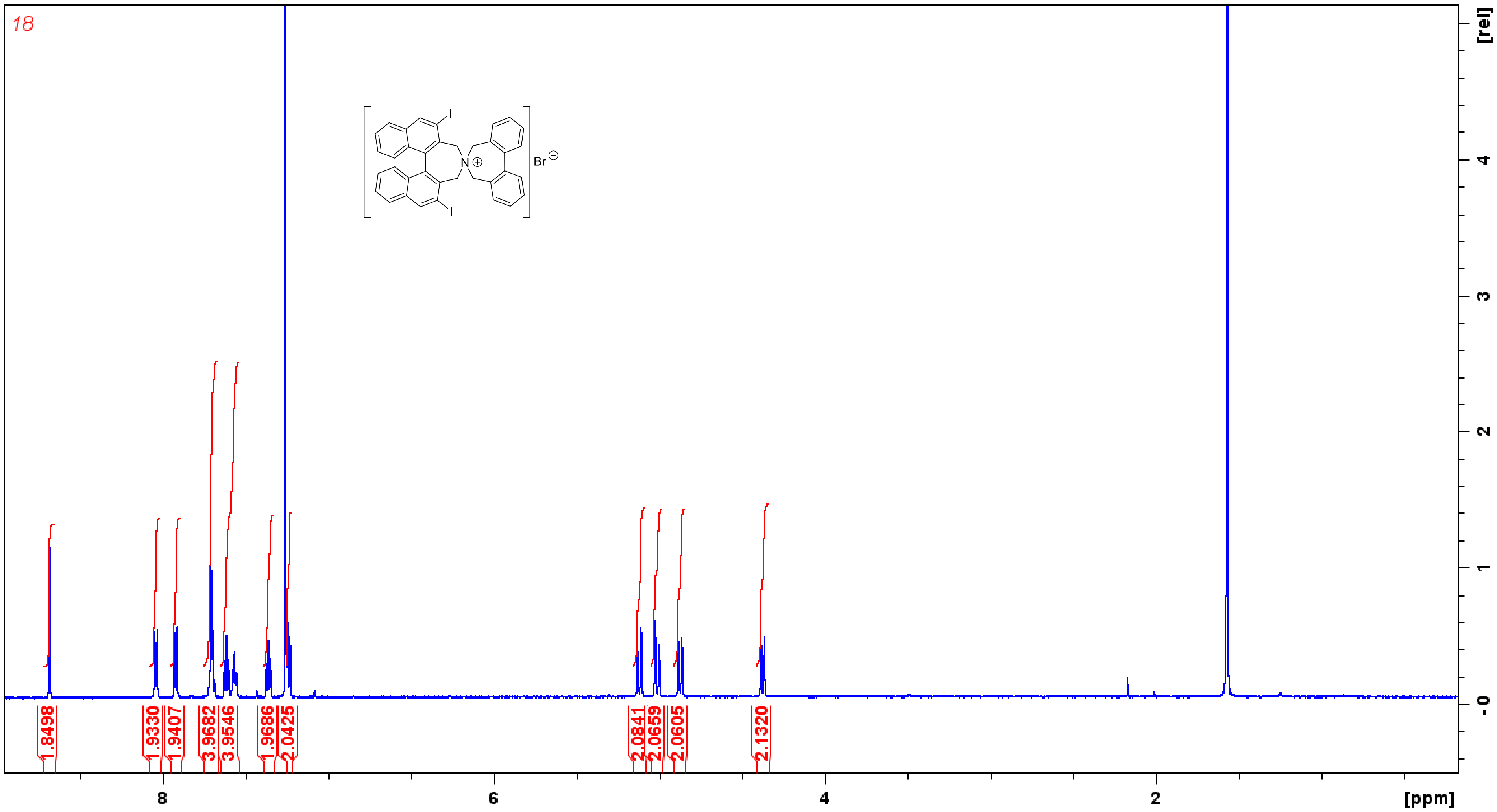
18
Br
N
I
I
1.8498
1.9330
1.9407
3.9682
3.9546
1.9686
2.0425
2.0841
2.0659
2.0605
2.1320
8
6
4
2
[ppm]
[rel]
0
1
2
3
4

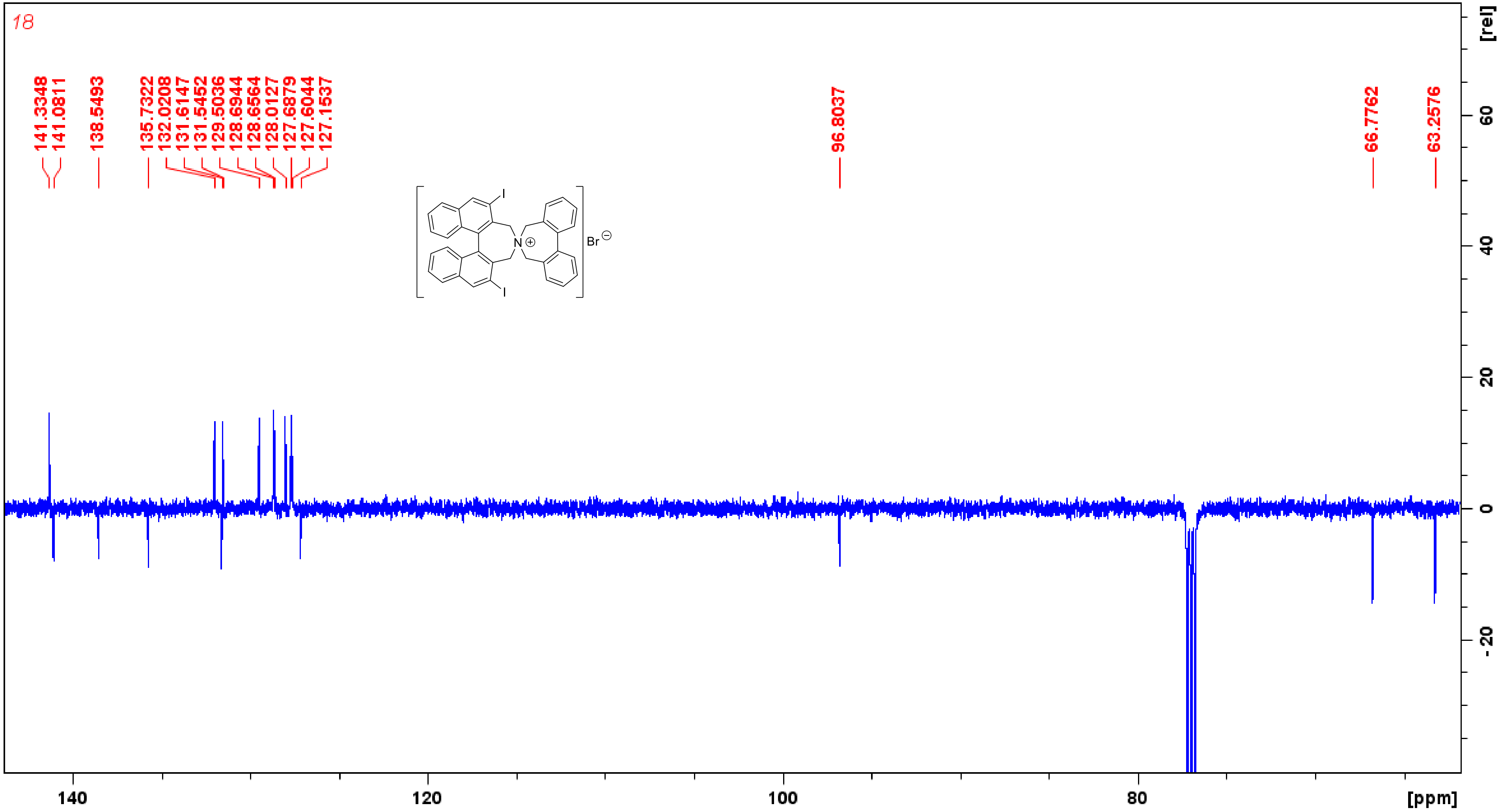
18
141.3348
141.0811
138.5493
135.7322
132.0208
131.6147
131.5452
129.5036
128.6944
128.6564
128.0127
127.6879
127.6044
127.1537
96.8037
66.7762
63.2576
I
I
N
Br
[rel]
60
40
20
0
-20
140
120
100
80
[ppm]

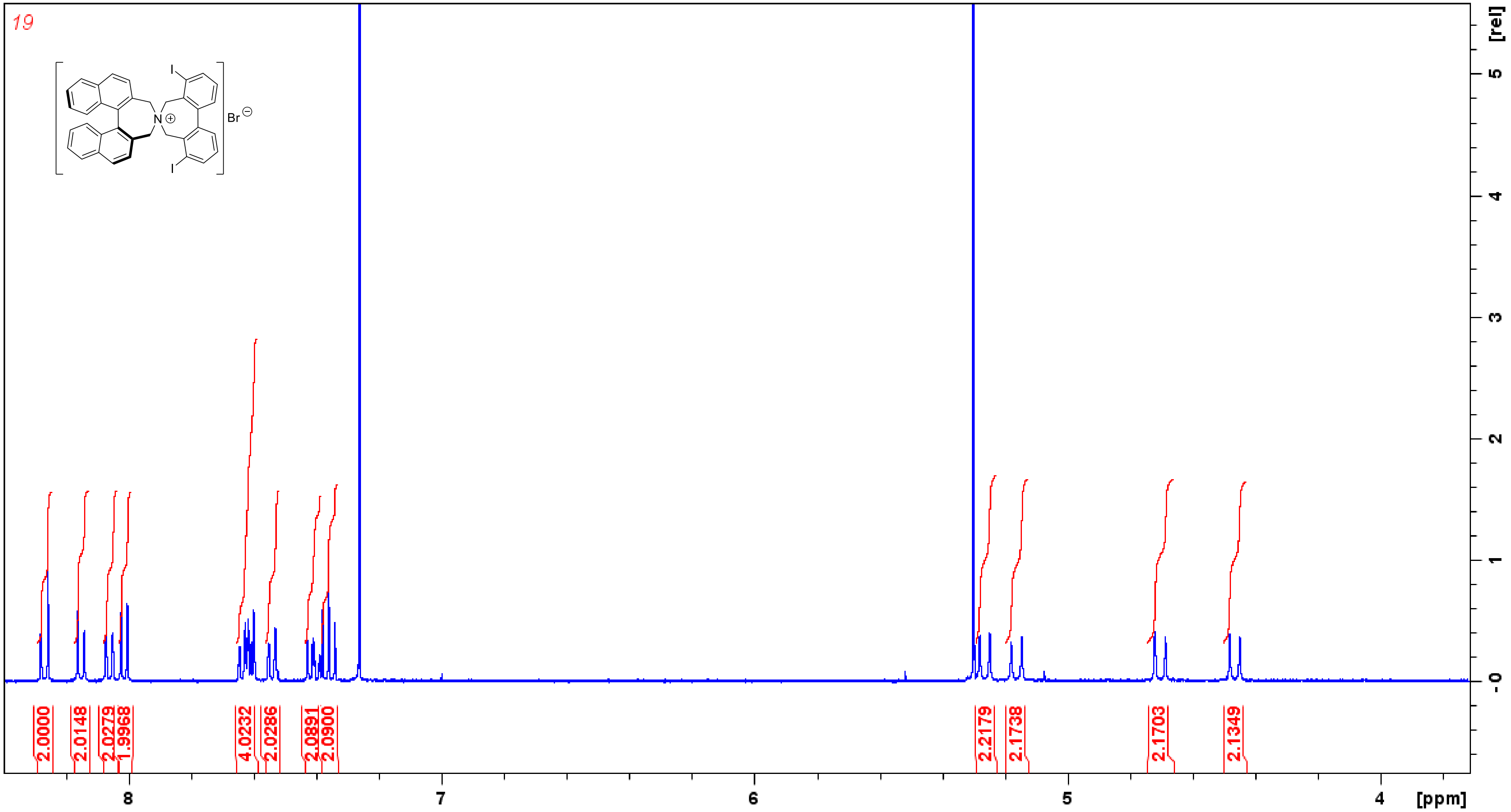
19
Br
N
I
I
[rel]
5
4
3
2
1
-0
2.0000
2.0148
2.0279
1.9968
4.0232
2.0286
2.0891
2.0900
2.2179
2.1738
2.1703
2.1349
8
7
6
5
4
[ppm]

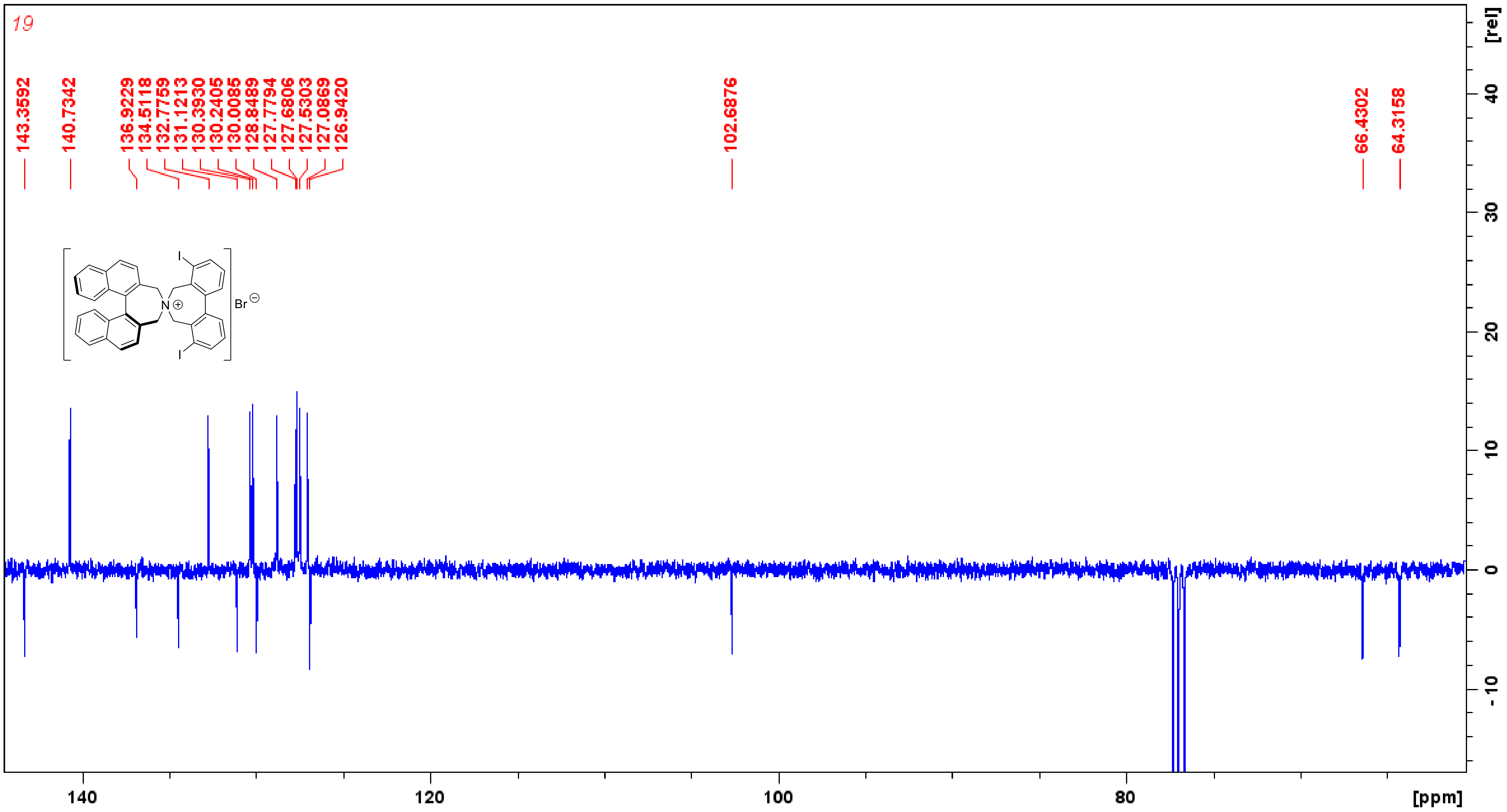
19
143.3592
140.7342
136.9229
134.5118
132.7759
131.1213
130.3930
130.2405
130.0085
128.8489
127.7794
127.6806
127.5303
127.0869
126.9420
102.6876
66.4302
64.3158
N
Br
I
I
140
120
100
80
[ppm]
[rel]
40
30
20
10
0
-10

X-ray Analysis

Experimental data and CCDC-Codes can be found in Table S2. Crystal data, data collection parameters, and structure refinement details are given in Tables S3 to S10. Crystal structures visualized in Figure S1 to S4.

Table S2: Experimental parameter and CCDC-Code.

**Sample**	**Machine**	**Source**	**Temp.**	**Detector Distance**	**Time/ Frame**	**#Frames**	**Frame width**	**CCDC**
	Bruker		[K]	[mm]	[s]		[°]	
**3a**	D8/ Kryoflex	Mo	100	40	100	735	0.55	1825002
**8**	D8/ Kryoflex	Mo	100	35	6.4	3372	0.40	1825003
**16**	D8/ Oxford	Mo	100	50	15	3532	0.35	1825004
**17b**	D8/ Kryoflex	Cu	100	34	50	1830	0.70	1825005

(*S,R**)-2',6'-Diphenyl-3',5,5',7-tetrahydrospiro[dibenzo[c,e]azepine-6,4'-dinaphtho[2,1-c:1',2'-e]azepin]-6-ium bromide (**3a**)

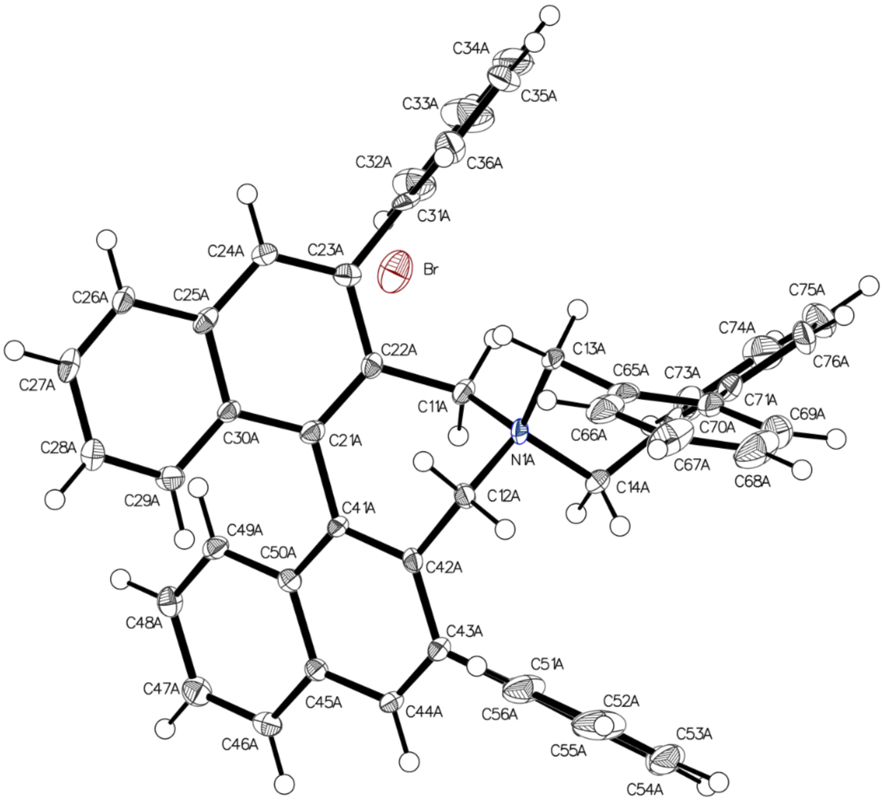

Figure S1: Crystal structure of **3a**, drawn with 50% displacement ellipsoids. The asymmetric unit is built up by 1 and 2*1/2 independent molecules of **3a**. The 2*1/2 molecules, one counter ion and $CHCl_3$ molecules are omitted for clarity. All three moieties form the same chiral arrangement. The centrosymmetric space group forces the inverse chiral form. Four voids with each 451.2 Å³ (9.6% of unit cell) had to be excluded from refinement. The corresponding value of electrons is 109.5 each. We could not find satisfactory positions for solvent atoms.

Table S3: Sample and crystal data of **3a**.

**Chemical formula**	$C_{50}H_{38}BrCl_6N$	**Crystal system**	monoclinic	
**Formula weight [g/mol]**	945.42	**Space group**	*C*2/*c*	
**Temperature [K]**	100	**Z**	16	
**Measurement method**	\f and \w scans	**Volume [Å³]**	18676.7(11)	
**Radiation (Wavelength [Å])**	MoKα (λ = 0.71073)	**Unit cell dimensions [Å] and [°]**	32.9747(11)	90
**Crystal size / [mm³]**	0.118 × 0.03 × 0.019		33.7652(11)	119.5218(14)
**Crystal habit**	clear colourless needle		19.2774(7)	90
**Density (calculated) / [g/cm³]**	1.345	**Absorption coefficient / [mm⁻¹]**	1.258	
**Abs. correction Tmin**	0.6858	**Abs. correction Tmax**	0.7452	
**Abs. correction type**	multiscan	**F(000) [e⁻]**	7712	

Table S4: Data collection and structure refinement of **3a**.

<table>
<tr><td>Index ranges</td><td>-39 ≤ h ≤ 39, -40 ≤ k ≤ 40, -23 ≤ l ≤ 23</td><td>Theta range for data collection [°]</td><td colspan="2">4.426 to 50.784</td></tr>
<tr><td>Reflections number</td><td>134422</td><td>Data / restraints / parameters</td><td colspan="2">17153/0/1049</td></tr>
<tr><td>Refinement method</td><td>Least squares</td><td rowspan="2">Final R indices</td><td>all data</td><td>R1 = 0.0870, wR2 = 0.1394</td></tr>
<tr><td>Function minimized</td><td>Σ w(F_o^2 - F_c^2)^2</td><td>I>2σ(I)</td><td>R1 = 0.0529, wR2 = 0.1249</td></tr>
<tr><td>Goodness-of-fit on F^2</td><td>1.035</td><td rowspan="2">Weighting scheme</td><td colspan="2">w=1/[$\sigma^2(F_o^2)+(0.0583P)^2+67.0953P$]</td></tr>
<tr><td>Largest diff. peak and hole [e Å^{-3}]</td><td>1.46/-1.15</td><td colspan="2">where P=($F_o^2+2F_c^2$)/3</td></tr>
</table>

2,6-Diiodo-4,5-dihydro-3*H*-dinaphtho[2,1-c:1',2'-e]azepine (**8**)

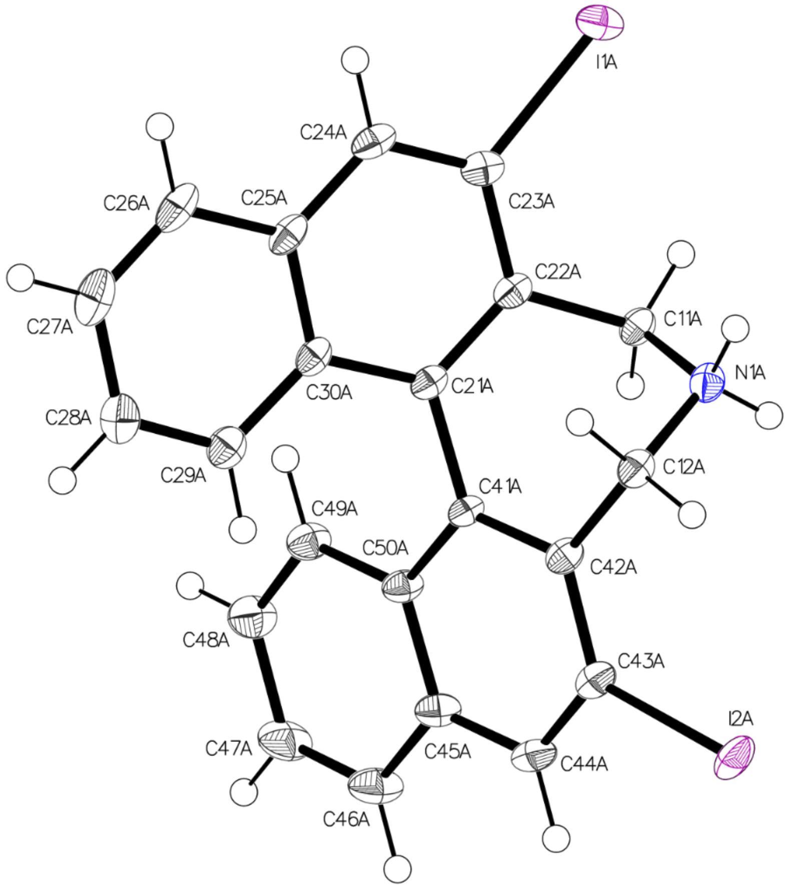

Figure S2: Crystal structure of **8**, drawn with 50% displacement ellipsoids. The asymmetric unit is built up by 2 independent molecules of **8**. Counter Ion, $CHCl_3$ and second independent moiety omitted for clarity. The two moieties form different chiral arrangements. Anyhow the chiral space group is proofed by Flack Parameter = 0.000(2).

Table S5: Sample and crystal data of **8**.

**Chemical formula**	$C_{24}H_{18}BrCl_6I_2N$	**Crystal system**	monoclinic	
**Formula weight [g/mol]**	866.8	**Space group**	*P21*	
**Temperature [K]**	100	**Z**	4	
**Measurement method**	\f and \w scans	**Volume [Å^3]**	2927.7(3)	
**Radiation (Wavelength [Å])**	MoKα (λ = 0.71073)	**Unit cell dimensions [Å] and [°]**	11.8178(7)	90
**Crystal size / [mm^3]**	0.253 × 0.217 × 0.204		17.2708(10)	94.2551(19)
**Crystal habit**	clear colourless block		14.3841(8)	90
**Density (calculated) / [g/cm^3]**	1.967	**Absorption coefficient / [mm^{-1}]**	4.076	
**Abs. correction Tmin**	0.6217	**Abs. correction Tmax**	0.746	
**Abs. correction type**	multiscan	**F(000) [e$^-$]**	1648	

Table S6: Data collection and structure refinement of **8**.

**Index ranges**	$-16 \leq h \leq 16$, $-24 \leq k \leq 24$, $-20 \leq l \leq 20$	**Theta range for data collection [°]**	3.456 to 60.186	
**Reflections number**	185276	**Data / restraints / parameters**	17236/1/629	
**Refinement method**	Least squares	**Final R indices**	all data	R1 = 0.0229, wR2 = 0.0496
**Function minimized**	Σ w(F_o^2 - F_c^2)^2		I>2σ(I)	R1 = 0.0215, wR2 = 0.0491
**Goodness-of-fit on F^2**	1.048	**Weighting scheme**	w=1/[σ^2(F_o^2)+(0.0231P)^2+2.2240P]	
**Largest diff. peak and hole [e Å^{-3}]**	1.09/-0.68		where P=(F_o^2+2F_c^2)/3	

(S,S)-2,6-Diiodo-3,3',5,5'-tetrahydro-4,4'-*spiro*bi[dinaphtho[2,1-c:1',2'-e]azepin]-4-ium bromide (**16**)

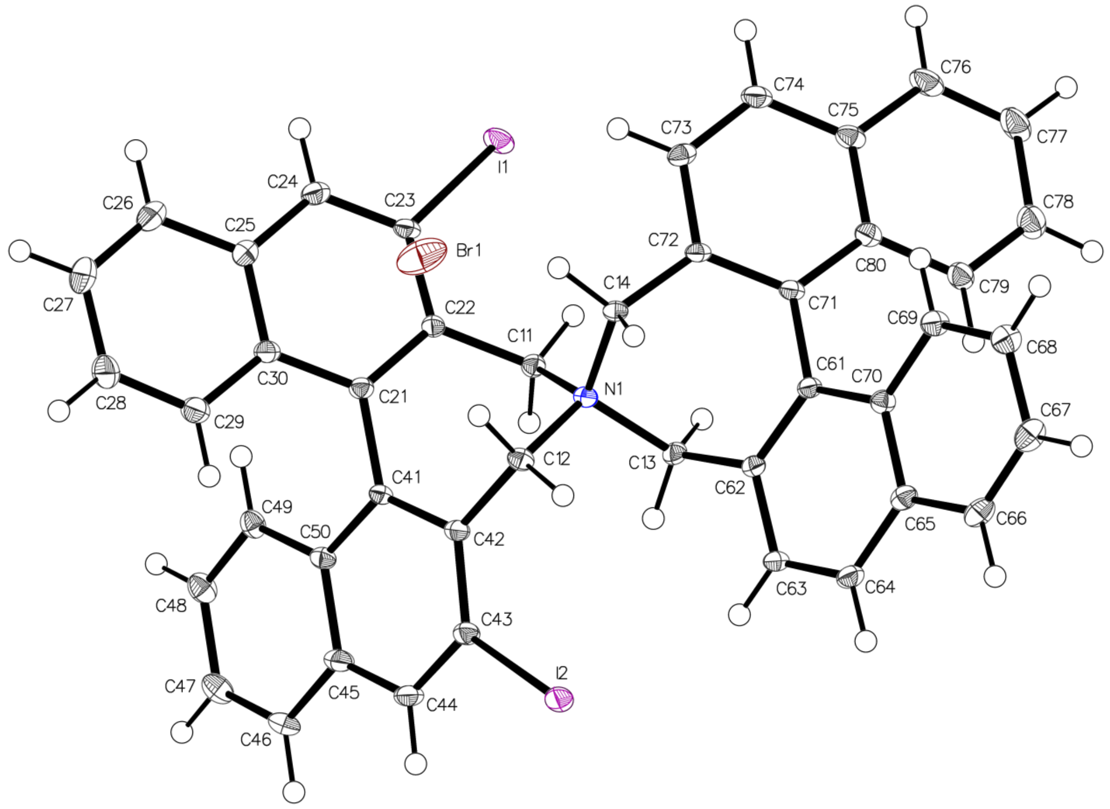

Figure S3: Asymmetric unit of **16**, drawn with 50% displacement ellipsoids. $CHCl_3$ omitted for clarity. The chiral space group is proofed by Flack Parameter = 0.059(3).

Table S7: Sample and crystal data of **16**.

**Chemical formula**	$C_{47}H_{36}BrCl_6I_2N$	**Crystal system**	orthorhombic	
**Formula weight [g/mol]**	1161.18	**Space group**	*P212121*	
**Temperature [K]**	100	**Z**	4	
**Measurement method**	\f and \w scans	**Volume [Å³]**	4402.1(5)	
**Radiation (Wavelength [Å])**	MoKα (λ = 0.71073)	**Unit cell dimensions [Å] and [°]**	8.9851(5)	90
**Crystal size / [mm³]**	0.161 × 0.152 × 0.048		11.0959(7)	90
**Crystal habit**	clear colourless block		44.154(3)	90
**Density (calculated) / [g/cm³]**	1.752	**Absorption coefficient / [mm^{-1}]**	2.736	
**Abs. correction Tmin**	0.6645	**Abs. correction Tmax**	0.747	
**Abs. correction type**	multiscan	**F(000) [e^{-}]**	2272	

Table 8: Data collection and structure refinement of **16**.

**Index ranges**	$-14 \leq h \leq 14$, $-18 \leq k \leq 18$, $-72 \leq l \leq 69$	**Theta range for data collection [°]**	4.598 to 71.546	
**Reflections number**	168354	**Data / restraints / parameters**	20447/0/523	
**Refinement method**	Least squares	**Final R indices**	all data	R1 = 0.0360, wR2 = 0.0736
**Function minimized**	$\Sigma\ w(F_o^2 - F_c^2)^2$		$I>2\sigma(I)$	R1 = 0.0318, wR2 = 0.0721
**Goodness-of-fit on F^2**	1.085	**Weighting scheme**	$w=1/[\sigma^2(F_o^2)+(0.0296P)^2+5.3285P]$	
**Largest diff. peak and hole [e Å^{-3}]**	1.42/-2.32		where $P=(F_o^2+2F_c^2)/3$	

(R,S)*-2,6-Di(naphthalen-2-yl)-4-(((S,R*)-2'-(naphthalen-2-ylmethyl)-[1,1'-binaphthalen]-2-yl)methyl)-4,5-dihydro-3*H*-dinaphtho[2,1-c:1',2'-e]azepine (**17b**)

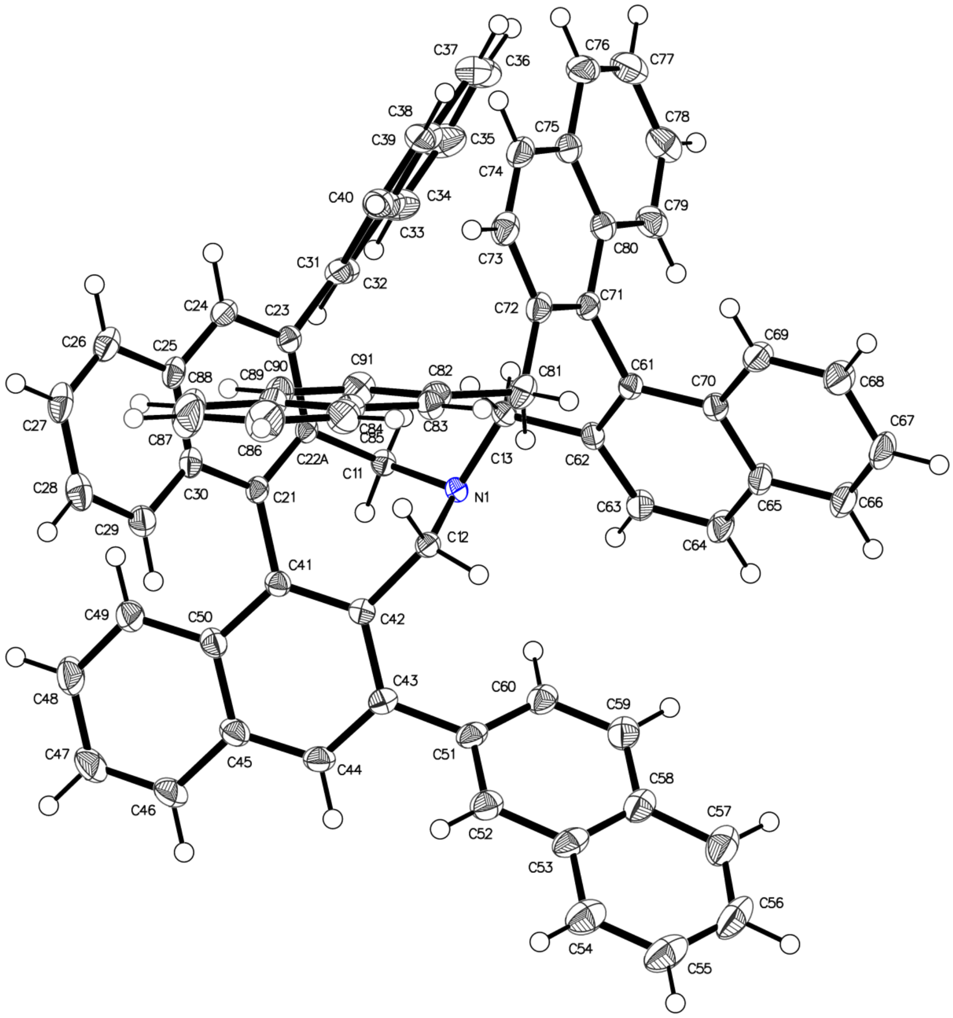

Figure S4: Asymmetric unit of **17b**, drawn with 50% displacement ellipsoids. CH_2Cl_2 omitted for clarity.

Table S9: Sample and crystal data of **17b**.

**Chemical formula**	$C_{77}H_{57}Cl_6N$	**Crystal system**	triclinic	
**Formula weight [g/mol]**	1208.93	**Space group**	*P-1*	
**Temperature [K]**	100	**Z**	2	
**Measurement method**	\f and \w scans	**Volume [Å³]**	3019.4(5)	
**Radiation (Wavelength [Å])**	CuKα (λ = 1.54178)	**Unit cell dimensions [Å] and [°]**	12.3238(10)	71.502(2)
**Crystal size / [mm³]**	0.259 × 0.198 × 0.098		13.5521(11)	86.172(4)
**Crystal habit**	clear colourless block		19.1746(19)	84.204(3)
**Density (calculated) / [g/cm³]**	1.33	**Absorption coefficient / [mm⁻¹]**	2.952	
**Abs. correction Tmin**	0.6112	**Abs. correction Tmax**	0.7536	
**Abs. correction type**	multiscan	**F(000) [e⁻]**	1256	

Table S10: Data collection and structure refinement of **17b**.

**Index ranges**	$-15 \leq h \leq 13$, $-16 \leq k \leq 16$, $-23 \leq l \leq 23$	**Theta range for data collection [°]**	6.902 to 146.862	
**Reflections number**	31469	**Data / restraints / parameters**	11696/15/766	
**Refinement method**	Least squares	**Final R indices**	all data	R1 = 0.0750, wR2 = 0.1912
**Function minimized**	Σ w(F_o^2 - F_c^2)^2		I>2σ(I)	R1 = 0.0707, wR2 = 0.1869
**Goodness-of-fit on F²**	1.084	**Weighting scheme**	w=1/[$\sigma^2(F_o^2)+(0.0876P)^2+5.0094P$]	
**Largest diff. peak and hole [e Å⁻³]**	1.30/-1.38		where P=($F_o^2+2F_c^2$)/3	

References

1 Kanoh, S.; Hongoh, Y.; Motoi, M.; Suda, H. Convenient Optical Resolution of Axially Chiral 1,1′-Binaphthyl-2,2′-dicarboxylic Acid. *Bull. Chem. Soc. Jpn.*, **1988**, *61*, 1032–1034.

2 Oi, S.; Matsuzaka, Y.; Yamashita, J.; Miyano, S. A Practical Method for Optical Resolution of 1,1′-Binaphthyl-2,2′-dicarboxylic Acid via 1-Phenylethylamides. *Bull. Chem. Soc. Jpn.* **1989**, *62*, 956–957.

3 Seki, M.; Yamada, S.; Kuroda, K.; Imashiro, R.; Shimizu, T. A Practical Synthesis of C_2-Symmetric Chiral Binaphthyl Ketone Catalyst. *Synthesis* **2000**, 1677–1680.

4 Cai, D.; Hughes, D.L.; Verhoeven, T.R.; Reider, P.J. Simple and Efficient Resolution of 1,1′-Bi-2-naphthol. *Tetrahedron Lett.* **1995**, *36*, 7991–7994.

5 Hu, Q.-S.; Vitharana, D.; Pu, L. An Efficient and Practical Direct Resolution of Racemic 1,1′-Bi-2-naphthol to Both of Its Pure Enantiomers. *Tetrahedron: Asymm.* **1995**, *6*, 2123–2126.

6 Hughes, D.L. Resolution of 1,1′-Bi-2-naphthol; (R)-(+)- and (S)-(-)-2,2′-Bis(diphenylphosphino)-1,1′-binaphthyl (BINAP). *Org. Synth.* **2014**, *91*, 1–11.

7 Rohde, V.H.G.; Müller, M.F.; Oestreich, M. Intramolecularly Sulfur-Stabilized Silicon Cations with Chiral Binaphthyl Backbones: Synthesis of Three Different Motifs and Their Application in Enantioselective Diels−Alder Reactions. *Organometallics* **2015**, *34*, 3358–3373.

8 Schafer, A.G.; Wieting, J.M.; Fisher, T.J.; Mattson, A. E.: Chiral Silanediols in Anion-Binding Catalysis. *Angew. Chem. Int. Ed.* **2013**, *52*, 11321–11324.

9 Sun, X.; Li, W.; Zhou, L.; Zhang, X. Matching and Mismatching Effects of Hybrid Chiral Biaxial Bisphosphine Ligands in Enantioselective Hydrogenation of Ketoesters. *Chem. Eur. J.* **2009**, *15*, 7302–7305.

10 Ikunaka, M.; Maruoka, K.; Okuda, Y.; Ooi, T. A Scalable Synthesis of (*R*)-3,5-Dihydro-4*H*-dinaphth[2,1-c:1′2′-e]azepine. *Org. Process Research & Development* **2003**, *7*, 644–648.

11 Ooi, T.; Kameda, M.; Maruoka, K. Design of *N*-Spiro C_2-Symmetric Chiral Quaternary Ammonium Bromides as Novel Chiral Phase-Transfer Catalysts: Synthesis and Application to Practical Asymmetric Synthesis of α Amino Acids. *J. Am. Chem. Soc.* **2003**, *125*, 5139–5151.

12 Miyano, S.; Okada, S.; Suzuki, T.; Handa, S.; Hashimoto, H. Practical Synthesis of 1,1′-Binaphthyl-2-carboxylic Acids via Side Chain Oxidation of 2-Methyl-1,1′-binaphthyls. *Bull. Chem. Soc. Jpn.* **1986**, *59*, 2044–2046.

13 Hayashi,T.; Hayashizaki, K., Kiyoi, T.; Ito, Y. Asymmetric Synthesis Catalyzed by Chiral Ferrocenylphosphine-Transition-Metal Complexes. 6. Practical Asymmetric Synthesis of 1, 1′-Binaphthyls via Asymmetric Cross-Coupling with a Chiral [(Alkoxyalkyl)ferrocenyl]mono-phosphine/Nickel Catalyst. *J. Am. Chem. Soc.* **1988**, *110*, 8153–8156.

14 Uozumi, Y.; Matsuura, Y.; Suzuka, T.; Arakawa, T.; Yamada, Y.M.A. Palladium-Catalyzed Asymmetric Suzuki–Miyaura Cross Coupling with Homochiral Phosphine Ligands Having Tetrahydro-1*H*-imidazo[1,5-a]indole Backbone. *Synthesis* **2017**, *49*, 59–68.

15 Lesieur, M.; Slawin, A.M.Z.; Cazin, C.S.J. $[Pd(\mu\text{-}Cl)Cl(IPr^*)]_2$: a highly hindered pre-catalystfor the synthesis of tetra-*ortho*-substituted biaryls via Grignard reagent cross-coupling. *Org. Biomol. Chem.* **2014**, *12*, 5586–5589.

16 Li, G.-Q.; Yamamoto, Y.; Miyaura, N. Synthesis of Tetra-*ortho*-Substituted Biaryls Using Aryltriolborates. *Synlett*, **2011**, 1769–1773.

17 Genov, M.; Almorin, A.; Espinet, P. Efficient Synthesis of Chiral 1,1′- Binaphthalenes by the Asymmetric Suzuki–Miyaura Reaction: Dramatic Synthetic Improvement by Simple Purification of Naphthylboronic Acids. *Chem. Eur. J.* **2006**, *12*, 9346–9352.

18 Ohta, T.; Ito, M.; Inagaki, K.; Takaya, H. A convenient synthesis of optically pure dimethyl 1,1'-binaphthalene-2,2'-dicarboxylate from 1,1'-binaphthalene-2,2'-diol. *Tetrahedron Lett.* **1993**, *34*, 1615–1616.

19 Egami, H.; Sato, K.; Asada, J.; Kawato, Y.; Hamashima, Y. Concise synthesis of binaphthol-derived chiral dicarboxylic acids. *Tetrahedron* **2015**, *71*, 6384–5388.

20 Konishi, H.; Hoshino, F.; Manabe, K. Practical Synthesis of Axially Chiral Dicarboxylates via Pd-Catalyzed External-CO-Free Carbonylation. *Chem. Pharm. Bull.* **2016**, *64*, 1438–1441

21 Lewin, A.H.; Zovko, M.J.; Rosewater, W.H.; Cohen, T. The use of complexing agents in the activation of copper for the Ullmann biaryl synthesis. *Chem. Commun.* **1967**, 80–81.

22 Podgorsek, A.; Stavber, S.; Zupan, M.; Iskra, J. Environmentally benign electrophilic and radical bromination 'on water': H_2O_2–HBr system versus N-bromosuccinimide *Tetrahedron* **2009**, *65*, 4429–4439.

23 Vautravers, N.R.; Regent, D.D.; Breit, B. Inter- and intramolecular hydroacylation of alkenes employing a bifunctional catalyst system. *Chem. Commun*. **2011**, *47*, 6635–6637.

24 Wommack, A.J.; Kingsbury, J.S. Synthesis of Acyclic Ketones by Catalytic, Bidirectional Homologation of Formaldehyde with Nonstabilized Diazoalkanes. Application of a Chiral Diazomethyl(pyrrolidine) in Total Syntheses of Erythroxylon Alkaloids, *J. Org. Chem.* **2013**, *78*, 10573–10587.

25 Andrus, M.B.; Asgari, D.; Sclafani, J.A. Efficient Synthesis of 1,1′-Binaphthyl and 2,2′-Bi-o-tolyl-2,2′-bis(oxazoline)s and Preliminary Use for the Catalytic Asymmetric Allylic Oxidation of Cyclohexene *J. Org. Chem.* **1997**, *62*, 9365–9368.

26 Krätzschmar, F.; Kassel, M.; Delony, D.; Breder, A. Selenium-Catalyzed C(sp^3)-H Acyloxylation: Application in the Expedient Synthesis of Isobenzofuranones. *Chem. Eur. J.* **2015**, *21*, 7030–7034.

27 Rosini, C.; Tanturli, R.; Pertici, P.; Salvadori, P. Enantioselective dihydroxylation of olefins by osmium tetroxide in the presence of an optically active 1,1′-binaphthyl diamine derivative. *Tetrahedron: Asymmetry*, **1996**, *7*, 2971–2982.

28 Widhalm, M.; Abraham, M.; Arion, V.B.; Saarsalu, S.; Maeorg, U. A modular approach to a new class of phosphinohydrazones and their use in asymmetric allylic alkylation reactions. *Tetrahedron: Asymm.*, **2010**, *21*, 1971–1982.

29 Bulman Page, P.C.; Buckley, B.R.; Blacker, A. J. Iminium Salt Catalysts for Asymmetric Epoxidation: The First High Enantioselectivities. *Org. Lett.* **2004** *6*, 1543–1546.

30 Shen, H.-C.; Tang, J.-M.; Chang, H.-K.; Yang, C.-W.; Liu, R.-S. Short and Efficient Synthesis of Coronene Derivatives via Ruthenium-Catalyzed Benzannulation Protocol. *J. Org. Chem.* **2005**, *70*, 10113–10116.

31 Bulman Page, P.C.; Buckley, B.R.; Farah, M.M.; Blacker, A. J. Binaphthalene-Derived Iminium Salt Catalysts for Highly Enantioselective Asymmetric Epoxidation. *Eur. J. Org. Chem.* **2009**, 3413–3426.

32 Furutani, T.; Hatsuda, M.; Imashiro, R.; Seki, M. Facile synthesis of enantiopure 1,1′-binaphthyl-2,2′-dicarboxylic acid via lipase-catalyzed kinetic resolution. *Tetrahedron: Asymm.* **1999**, *10*, 4763–4768.

33 Schlosser, M.; Bailly, F. Embedding an Allylmetal Dimer in a Chiral Cavity: The Unprecedented Stereoselectivity of a Twofold Wittig [1,2]-Rearrangement. *J. Am. Chem. Soc.* **2006**, *128*, 16042–16043.

34 Widhalm, M.; Pacar, M., unpublished.
